# Chiral Lewis acid-bonded picolinaldehyde enables enantiodivergent carbonyl catalysis in the Mannich/condensation reaction of glycine ester†

**DOI:** 10.1039/d0sc07052a

**Published:** 2021-01-26

**Authors:** Xia Zhong, Ziwei Zhong, Zhikun Wu, Zhen Ye, Yuxiang Feng, Shunxi Dong, Xiaohua Liu, Qian Peng, Xiaoming Feng

**Affiliations:** Key Laboratory of Green Chemistry & Technology, Ministry of Education, College of Chemistry, Sichuan University Chengdu 610064 P. R. China liuxh@scu.edu.cn xmfeng@scu.edu.cn http://www.scu.edu.cn/chem_asl/; State Key Laboratory of Elemento-Organic Chemistry, College of Chemistry, Nankai University 94 Weijin Road Tianjin 300071 P. R. China qpeng@nankai.edu.cn

## Abstract

A new strategy of asymmetric carbonyl catalysis *via* a chiral Lewis acid-bonded aldehyde has been developed for the direct Mannich/condensation cascade reaction of glycine ester with aromatic aldimines. The co-catalytic system of 2-picolinaldehyde and chiral Yb^III^-*N*,*N*′-dioxides was identified to be efficient under mild conditions, providing a series of trisubstituted imidazolidines in moderate to good yields with high diastereo- and enantioselectivities. Enantiodivergent synthesis was achieved *via* changing the sub-structures of the chiral ligands. The reaction could be carried out in a three-component version involving glycine ester, aldehydes, and anilines with equally good results. Based on control experiments, the X-ray crystal structure study and theoretical calculations, a possible dual-activation mechanism and stereo-control modes were provided to elucidate carbonyl catalysis and enantiodivergence.

## Abstract 2

The catalytic asymmetric Mannich/condensation of glycine ester with aldimines was achieved by merging chiral *N*,*N*′-dioxide/Yb^III^ complex Lewis acid catalysis/carbonyl catalysis under mild condition.

## Graphical abstract

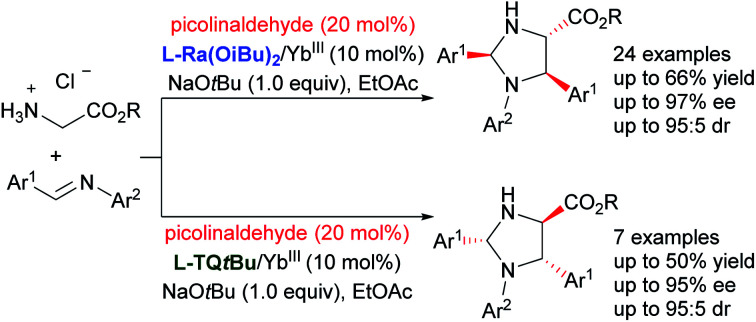

## Introduction

**Scheme 1:**
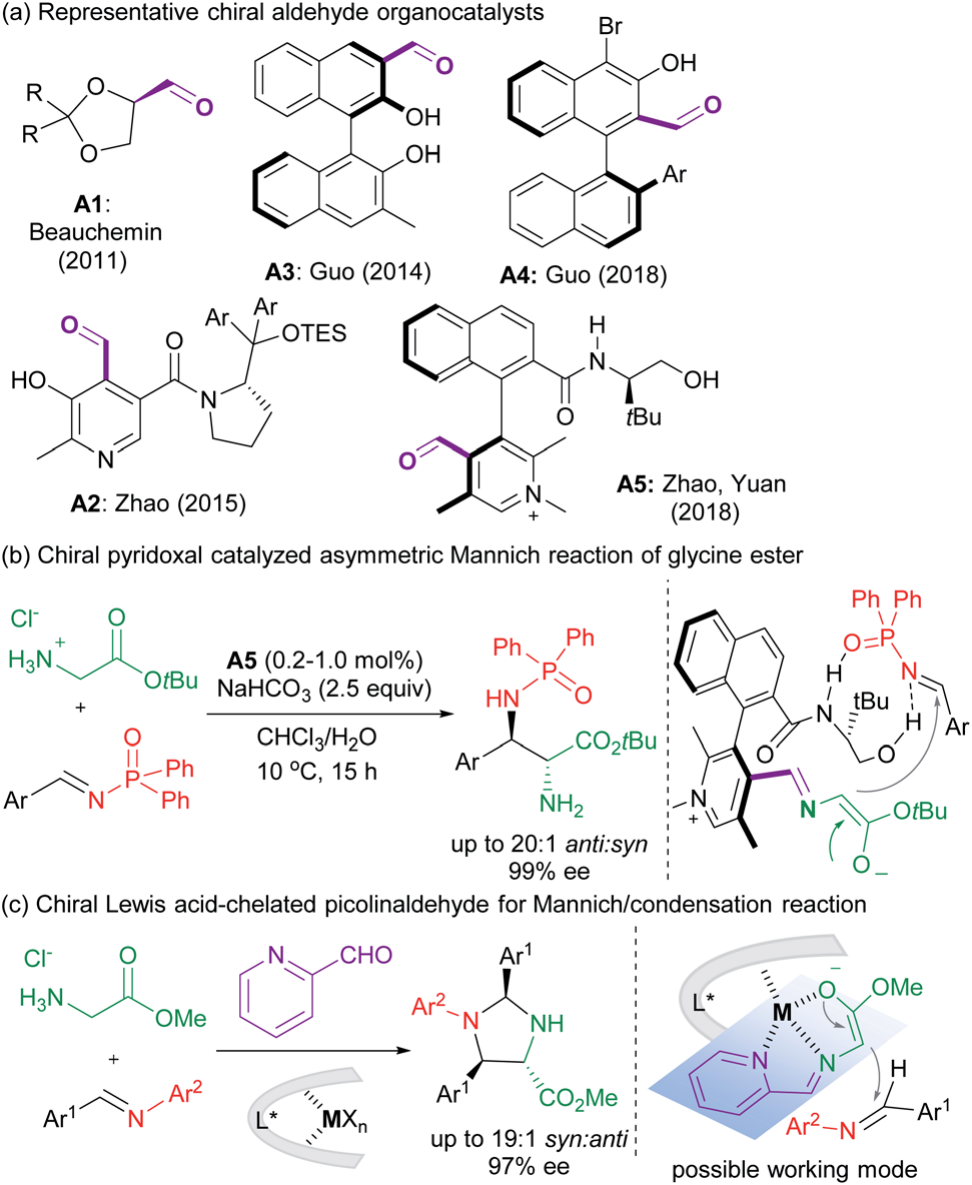
Carbonyl catalysts and selected examples of α-functionalization of glycine esters.

Asymmetric carbonyl catalysis,^1^ inspired by enzyme catalysis,^2^ represents a type of useful organo-covalent-activation method *via* formation of various species, such as hemiacetal,^3^ or imine,^4^ as well as dioxirane from chiral ketone for epoxidation.^5^ Substantial efforts in developing aldehyde-based chiral organocatalysts have been made after pioneering reports by Kuzuhara,^6^ Breslow and co-workers.^7^ Excellent contributions have been documented in recent years by Beauchemin,^8^ Guo,^9^ Zhao and Yuan^10^*et al.* (Scheme 1a), and the reactions involve Cope-type hydroamination,^8^ alkylation,^9*d*^ transamination,^10*a*^ and addition reactions of glycine esters.^9*a*,*b*,10*b*^ For instance, chiral *N*-quaternized pyridoxal analogue **A5** containing a chiral axis and a carbon stereogenic center allowed a biomimetic asymmetric Mannich reaction of *tert*-butyl glycine ester and *N*-diphenylphosphinyl imines (Scheme 1b).^10*b*^ Cooperative imine/hydrogen-bond bifunctional activation guaranteed high activity and stereoselectivity. On top of that, the dual-catalysis by Lewis acid-bonded picolinaldehyde has been explored by Aron's group in the synthesis of chiral amino acids.^11^ They found that the metal ion chelated quinonoid intermediate facilitated racemization and Tsuji–Trost allylation, and enantiomerically enriched amino acids could be obtained in concert with Alacase^11*a*^ or a chiral palladium catalyst.^11*b*^

These studies, together with the ingenious imine-based transient directing group strategy in transition metal-catalyzed C–H activation,^12^ intrigue us to develop chiral Lewis acid-bonded aldehyde as a new route for asymmetric carbonyl catalysis in α-functionalization of glycine esters,^13^ which might compensate for the design and synthesis of chiral aldehyde organocatalysts *de novo*. Our research group focused on chiral Lewis acid catalysts of *N*,*N*′-dioxides which are capable of a number of asymmetric transformations due to their easy preparation and structural modification.^14,15^ It is anticipated that the stereo-environment created by the chiral Lewis acid could deliver to the bonded-aldehyde for asymmetric carbonyl catalysis, for instance, the asymmetric nucleophilic addition of the azomethine ylides, generated from picolinaldehyde and glycine ester (Scheme 1c). The study by Wang's group reported the utilization of metallated azomethine ylides in [3 + 2] cycloadditions with a variety of electron deficient alkenes.^16^ The improved reactivity and efficient enantiocontrol from metal-bonded amino esters shed light on our new chiral imine-bonded activation method for the Mannich-type reaction of glycine ester. Herein, we wish to disclose a new co-catalytic system of 2-picolinaldehyde and a chiral Yb^III^ complex of *N*,*N*′-dioxides,^17^ which was optimized to be efficient to catalyze the diastereo- and enantioselective Mannich/condensation cascade reaction of glycine ester with aromatic aldimines under mild conditions (Scheme 1c). Various enantioenriched imidazolidines were provided in good yields with high stereoselectivities, even in the three-component version with glycine ester, aldehydes and alinines. Interestingly, changing the sub-structure of Feng *N*,*N*′-dioxides enabled enantiodivergent synthesis.^18^ Two different working modes were provided to give a rationale for carbonyl catalysis and enantiodivergence on the basis of the X-ray crystal structures of chiral Yb(iii) complexes and DFT calculations.

## Results and discussion

**Fig. 1:**
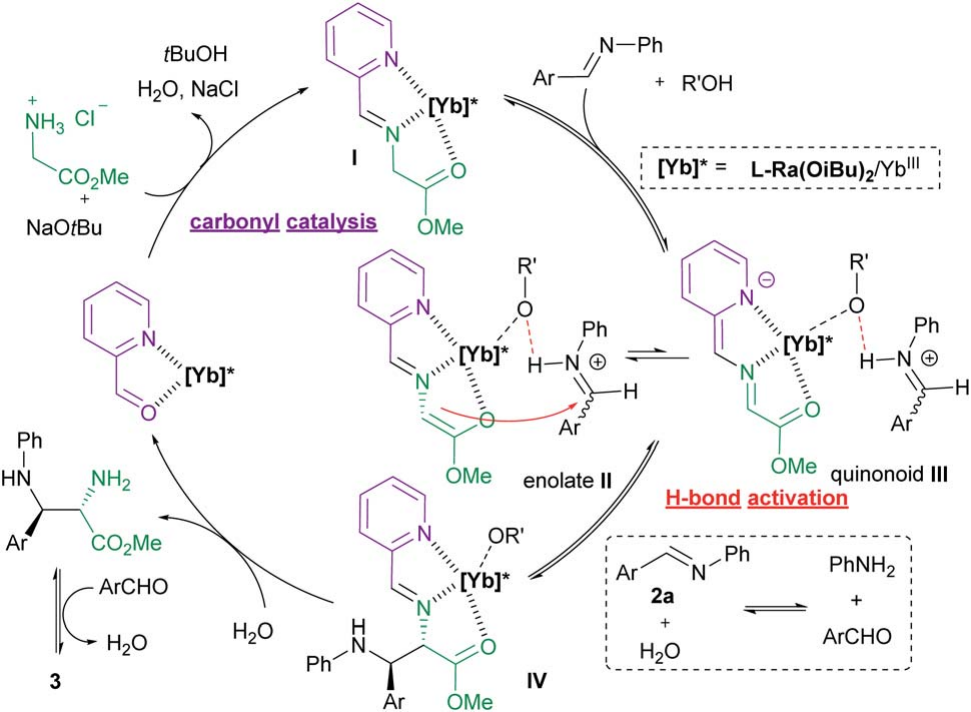
Proposed catalytic cycle.

Initially, methyl glycine ester hydrochloride (**1a**) and *N*-phenyl phenylmethanimine (**2a**)^19^ were chosen as the model substrates to optimize the reaction conditions. In the preliminary screening, the chiral complex of Yb(OTf)_3_ with Feng *N*,*N*′-dioxide **L-Ra(OiBu)2** was employed as the Lewis acid catalyst. NaO*t*Bu (1.0 equivalent) was used as the base to generate methyl glycine ester from **1a***in situ*, and 2-picolinaldehyde **C1** (10 mol%) was loaded as the carbonyl co-catalyst. The reaction took place smoothly in EtOAc at 35 °C, affording imidazolidine **3a** as the final product in 40% yield, 93 : 7 dr, and 95% ee after 24 hours (Table 1, entry 1), which is generated from the Mannich reaction of aldimine **2a** with glycine ester, following condensation with benzaldehyde (see Fig. 1 for details). The comparison experiments in the absence of 2-picolinaldehyde or in the presence of other aldehydes confirmed that both pyridine and aldehyde groups were indispensable. No reaction occurred without the addition of 2-picolinaldehyde (entry 2) or in the presence of benzaldehyde **C2** (entry 3), or isonicotinaldehyde **C3** (entry 4). Although the addition of 2-formylpyridine *N*-oxide **C4** benefitted the generation of imidazolidine **3a**, the enantioselectivity dropped dramatically (entry 5, 9% ee *vs.* 95% ee). We rationalized that the stronger chelated 2-picolinaldehyde and the resultant azomethine ylide were involved in initiating the Mannich reaction. We also identified other critical parameters for this asymmetric transformation (see ESI Tables S1–S8† for details). Subsequent investigation of Feng *N*,*N*′-dioxides with different chiral backbones indicated that the (*S*)-2-pipecolic acid-derived **L-Pi(OiBu)2** exhibited comparable activity with slightly lower stereoselectivity, while L-perindopril-derived **L-Pe(OiBu)2** showed lower activity with similar stereoselectivity (entries 6 and 7). Notably, the introduction of (*S*)-1,2,3,4-tetrahydroisoquinoline-3-carboxylic acid-derived *N*,*N*′-dioxide ligand **L-TQtBu**, led to the enantiodivergent synthesis of the corresponding product *ent*-**3a** in 34% yield, and 86 : 14 dr with 87% ee (entry 8). With the increase of the amount of co-catalyst **C1** to 20 mol%, the product was isolated in a slightly higher yield with comparable stereoselectivity (entry 9, 44% yield, 93 : 7 dr, 95% ee). Besides, the addition of EtOH as a protonic additive (entry 10), and increasing the amount of aldimine **2a** (entry 11) resulted in the formation of **3a** with 62% yield, 95 : 5 dr and 97% ee. Under such conditions, the product *ent-***3a** could be afforded in 50% yield, 88 : 12 dr and 95% ee by the use of ligand **L-TQtBu** instead (entry 12). Due to the occurence of several side reactions, the attempt to improve the yield of the product was unsuccessful (see the ESI† and control experiments below for details).

**Table 1:** Optimization of reaction conditions^a^

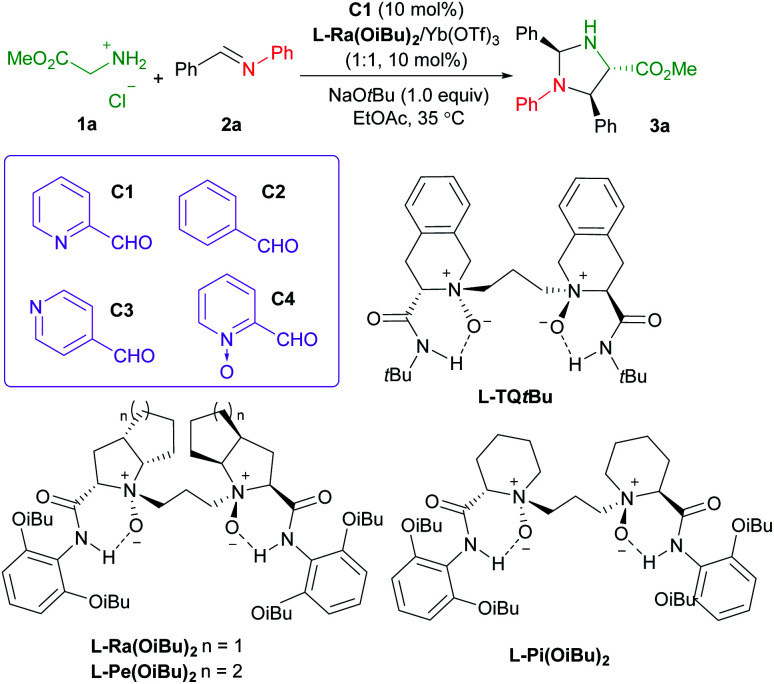				
Entry	Variation	Yield^b^ (%)	dr^c^	ee^c^ (%)
1	None	40	93 : 7	95(+)
2	Without **C1**	N.R.	—	—
3	**C2**	N.R.	—	—
4	**C3**	N.R.	—	—
5	**C4**	45	81 : 19	9(+)
6	**L-Pi(OiBu)2**	43	90 : 10	91(+)
7	**L-Pe(OiBu)2**	34	92 : 8	97(+)
8	**L-TQtBu**	34	86 : 14	87(−)
9	**C1** (20 mol%)	44	93 : 7	95(+)
10^d^	**C1** (20 mol%), EtOH	46	94 : 6	97(+)
11^d^^,^^e^	**C1** (20 mol%), EtOH	62	95 : 5	97(+)
12^d^^,^^e^	**L-TQtBu**, **C1** (20 mol%)	50	88 : 12	95(−)

a Unless otherwise noted; all reactions were carried out with **1a** (0.10 mmol), **2a** (2.0 equiv.), NaO*t*Bu (1.0 equiv.), carbonyl catalyst (10 mol%) and ligand/Yb(OTf)_3_ (1 : 1, 10 mol%) in EtOAc (0.17 M) at 35 °C for 24 hours. N.R. = no reaction.

b Isolated yield of **3a** based on **1a**.

c Determined by HPLC on a chiral stationary phase.

d EtOH (8.0 equiv.).

e **2a** (3.0 equiv.) for 48 hours.

With the optimized reaction conditions established (Table 1, entry 11), the generality of the aldimines was explored (Table 2, conditions A). Firstly, a series of aromatic aldehyde-derived imines were investigated (**3b–3p**). Regardless of the electronic nature or steric hindrance of the substituents on the phenyl ring (Ar^1^), the imidazolidine products **3b–3k** were obtained in 31–66% yields with excellent stereoselectivities (82 : 18–95 : 5 dr, 73–97% ee). Aldimine derived from 3,4-dimethylbenzaldehyde was tolerated (**3l**, 39% yield, 93 : 7 dr and 90% ee). The reaction of aldimines derived from 2-naphthaldehyde or heteroaryl aldehydes proceeded well, affording **3m–3p** with comparative results (50–56% yields, 90 : 10–94 : 6 dr, 94–97% ee). The following screening of the *N*-aryl substituent (Ar^2^) of aldimines showed that *para*-substituted aniline based aldimine had a limited effect on the reaction (**3q–3t**, 35–46% yields, 90 : 10–94 : 6 dr, 95–97% ee). The 4-methoxyaniline derived one resulted in a high enantioselectivity with a decreased yield (**3r**, 35% yield, 92 : 8 dr, 95% ee). It should be noted that for several products, the use of the ligand **L-Pi(OiBu)2** or **L-Pe(OiBu)2** instead was necessary to get higher yields without the change of stereo-preference. The absolute configuration of the product **3n** was determined to be (2*S*,4*S*,5*S*)-isomer by X-ray single crystal analysis.^20^ The stereochemistry of other products was assigned by comparing their CD spectra with those of the product **3n**.

**Table 2:** Substrate Scope of Aldimines^a^

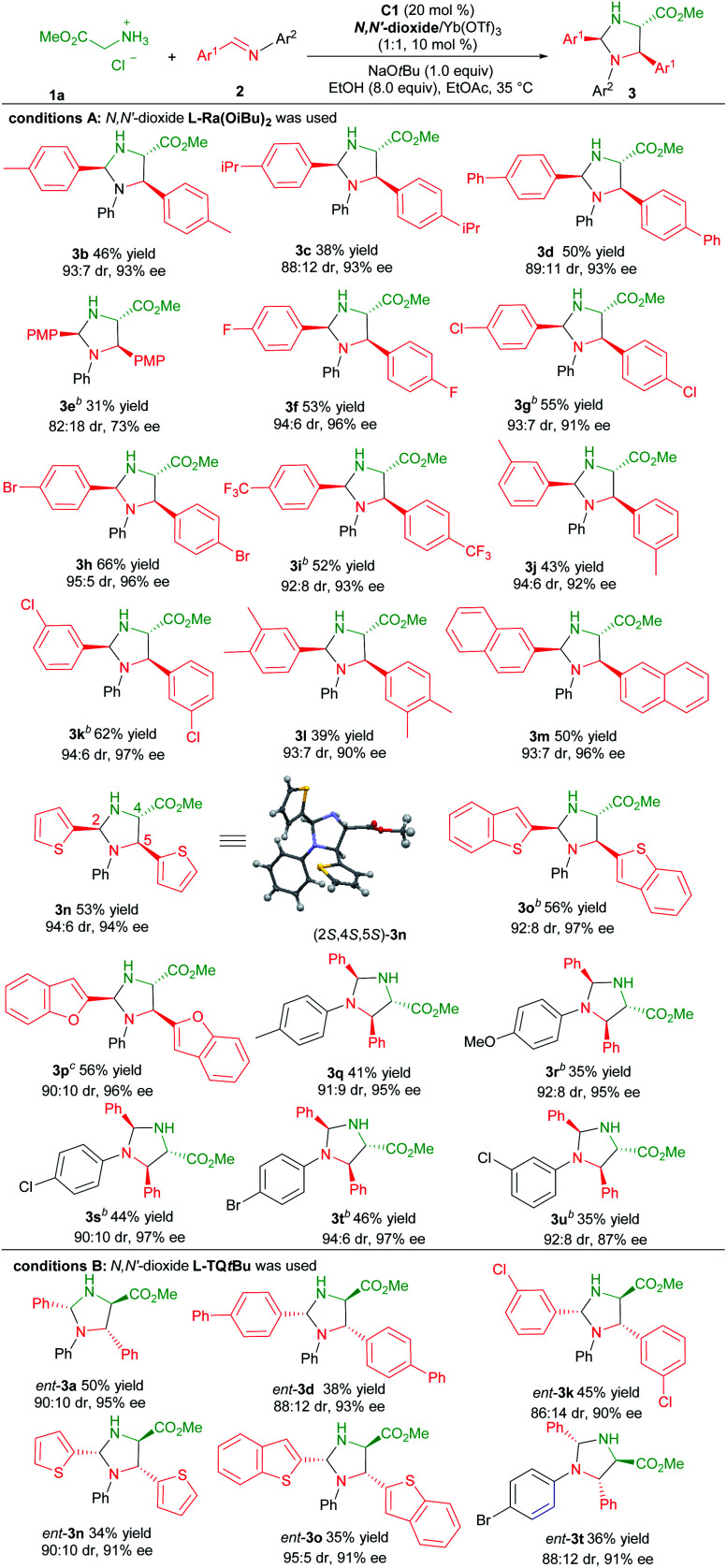

a Reactions were carried out with **1a** (0.10 mmol), **2** (3.0 equiv.), NaO*t*Bu (1.0 equiv.), **C1** (20 mol%), EtOH (8.0 equiv.) and **L**/Yb(OTf)_3_ (1 : 1, 10 mol%) in EtOAc (0.17 M) at 35 °C for 48 hours. The isolated yield of **3** based on **1a**. The dr value was detected by ^1^H NMR analysis, and the ee value was determined by HPLC on a chiral stationary phase.

b **L-Pi(OiBu)2** was used as the ligand.

c **L-Pe(OiBu)2** was used as the ligand.

Next, we proceeded to prepare the antipodes of the product **3** by the employment of the chiral **L-TQtBu**/Yb(OTf)_3_ complex as the Lewis acid catalyst. Representative aldimines were examined and the results are depicted in Table 2, condition B. Generally, these reactions occurred smoothly to deliver the corresponding products *ent*-**3** with high enantio- and diastereoselectivity in slightly diminished yields (34–50% yields, 86 : 14–95 : 5 dr, 90–95% ee).

In order to improve the practicability of the reaction, we investigated the three-component synthesis of imidazolidines, from glycine ester, aldehyde and aniline. As shown in Table 3, the three-component reaction was accomplished under slightly modified conditions (see ESI Table S9† for details), and an array of aldehydes **4** and anilines **5** were surveyed. In comparison with the two-component reaction system, similar results were given (39–55% yields, 88 : 12–94 : 6 dr, 88–97% ee). By using this protocol, the product **3v** was yielded in moderate yield with good diastereo- and enantioselectivity (Table 3, entry 6), which is not possible from the two-component reaction of *N-m*-tolyl substituted aldimine due to the separation and purification problem of aldimine. Unfortunately, aliphatic amines and aldehydes were not suitable in the current system.

**Table 3:** Three-component reaction^a^

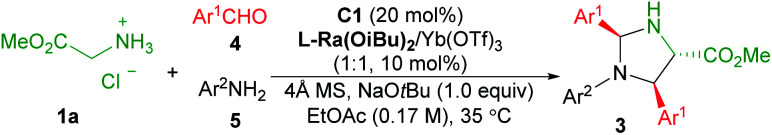				
Entry	Ar^1^/Ar^2^	Yield (%)	dr	ee (%)
1	Ph/Ph (**3a**)	55	93 : 7	96
2	4-MeC_6_H_4_/Ph (**3b**)	40	88 : 12	88
3^b^	4-ClC_6_H_4_/Ph (**3g**)	53	94 : 6	91
4	Ph/4-MeC_6_H_4_ (**3q**)	39	94 : 6	93
5^b^	Ph/4-ClC_6_H_4_ (**3s**)	44	94 : 6	97
6^c^	Ph/3-MeC_6_H_4_ (**3v**)	41	92 : 8	87

a Reactions were carried out with **1a** (0.10 mmol), **4** (3.0 equiv.), **5** (3.0 equiv.), NaO*t*Bu (0.10 mmol), **C1** (20 mol%), 4 Å MS (20.0 mg), and Yb(OTf)_3_/**L-Ra(OiBu)2** (1 : 1, 10 mol%) in EtOAc (0.17 M) at 35 °C for 48 hours. The isolated yield of **3** based on **1a**. The dr value was detected by ^1^H NMR analysis and the ee value was determined by HPLC on a chiral stationary phase.

b With **L-Pi(OiBu)2**.

c With **L-Pe(OiBu)2**.

**Scheme 2:**
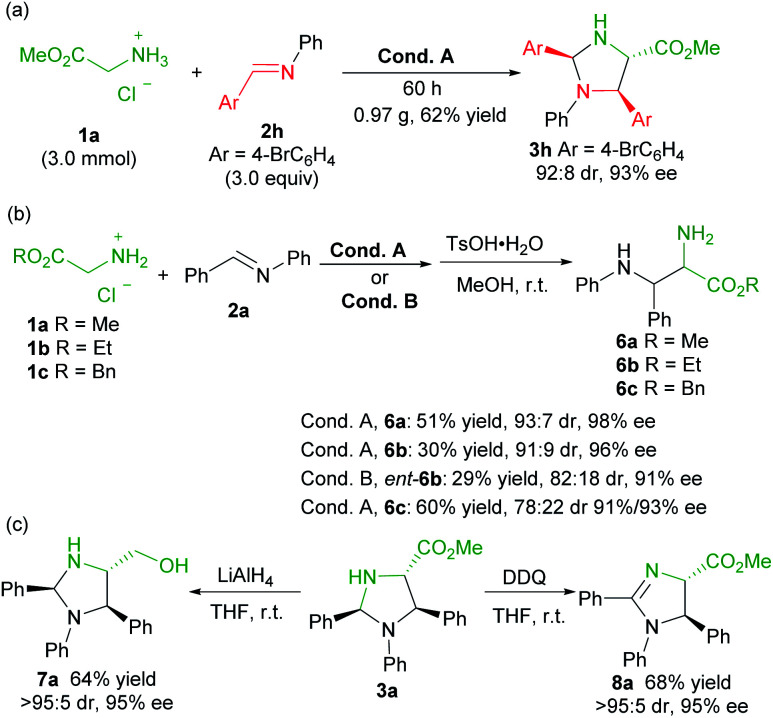
Gram-scale synthesis and transformations.

To evaluate the synthetic potential of the catalytic system, a scale-up preparation of imidazolidine **3h** was carried out (Scheme 2a). Upon treatment of 3.0 mmol of **1a** with 9.0 mmol of **2h** under the optimized reaction condition A, the desired product **3h** was obtained in 62% yield (0.97 g), with 92 : 8 dr and 93% ee after 60 hours. Furthermore (Scheme 2b), the product **3a** was successfully converted into the chiral vicinal diamine product **6a***via* TsOH-mediated hydrolysis in 51% yield, 93 : 7 dr and 98% ee after two steps. The change of the ester group had an obvious effect on the transformation, and ethyl glycine ester **1b** led to a reduced yield (**6b**, 30% yield), and benzyl glycine ester **1c** provided a lower diastereoselectivity (**6c**, 78 : 22 dr, 91%/93% ee). Similarly, *ent*-**6b** diamine was obtained in comparable yield when **L-TQtBu** was used (29%, 82 : 18 dr, 91% ee). Noteworthily, the *syn*-diamine derivatives **6** were dominant in the current case (Scheme 2b), which is different from the chiral pyridoxal analogue **A5** based catalytic system.^10*b*^ Reduction of the ester to a primary alcohol led to the imidazolidine derivative **7a** in good yield with maintained stereoselectivity. In addition, chiral 2-imdazoline **8a** was produced by oxidation with DDQ (Scheme 2c).

**Scheme 3:**
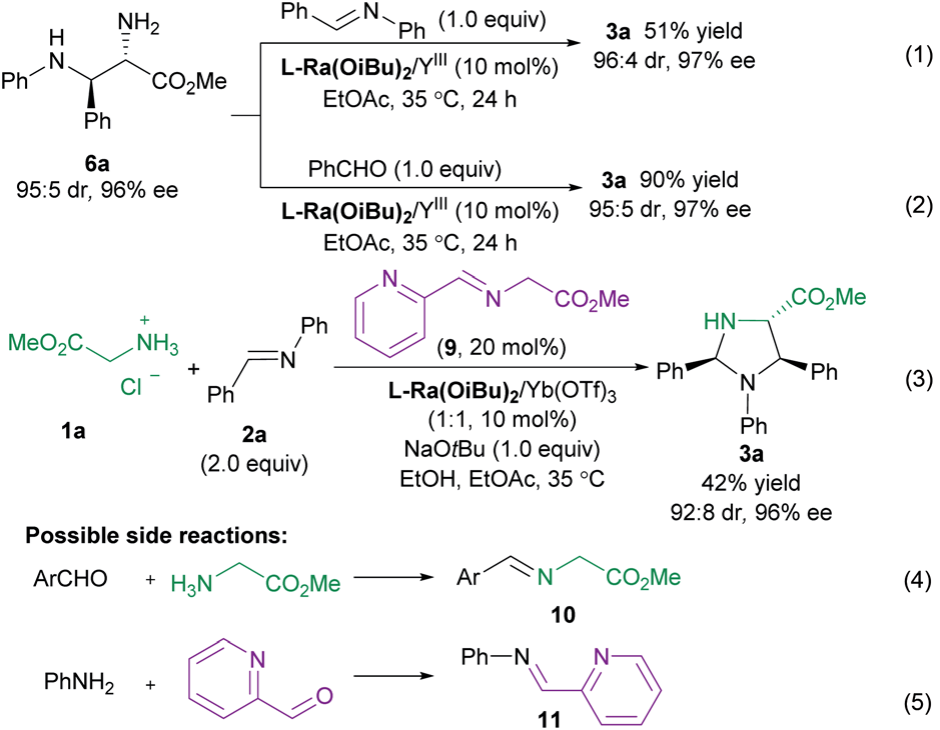
Control experiments.

To get insight into the mechanism of the reaction, a series of control experiments were conducted (Scheme 3). Treatment of chiral diamine **6a** with aldimine **2a** in the presence of the **L-Ra(OiBu)2**/Yb(OTf)_3_ complex delivered the imidazolidine **3a** in 51% yield with maintained diastereoselectivity and enantioselectivity (eqn (1)). In contrast, a yield of 90% was obtained if benzaldehyde was used for the formation of imidazolidine **3a** (eqn (2)). These results clearly indicated that the final imidazolidine product **3a** was generated *via* a stepwise Mannich/condensation cascade reaction with aldehyde released from the decomposition of aldimine **2a**. Therefore, this reaction was different from the previous direct [3 + 2] cycloaddition of azomethine ylides with aldimines.^21^ When the Schiff base **9** of 2-picolinaldehyde **C1** and methyl glycine ester was used instead of 2-picolinaldehyde, the product **3a** was isolated in 42% yield with 92 : 8 dr and 96% ee (eqn (3)). It confirmed that 2-picolinaldehyde probably serves as the carbonyl catalyst to generate the Schiff base intermediate for the subsequent addition reaction. According to HRMS analysis of the reaction mixture, Schiff base **10** and picolinaldehyde-derived imine **11** might exist in the reaction system (eqn (4) and (5)). However, these species were unreactive under the current reaction conditions (see ESI page S24–S27 for detail†). These side reactions along with the instability of products were partly responsible for the moderate yields of the titled process.

The influence of the concentration of each component on the reaction rate was detected from operando IR profiles (see ESI page S19–S22 for details†). The kinetic study showed that the initial rate of the reaction was first-order depending on the chiral **L-Ra(OiBu)2**/Yb(OTf)_3_ complex, glycine ester, 2-picolinaldehyde **C1** and aldimine **2**, indicating that these species are involved in the rate-determining process. Furthermore, the HRMS spectra of the mixture of **L-Ra(OiBu)2**, Yb(OTf)_3_, **C1**, **1a**, and NaO*t*Bu (1 : 1 : 1 : 1 : 1) in MeOH exhibited the ion peak at *m*/*z* 1470.4521, referring to [**L-Ra(OiBu)2** + Yb^3+^ + 2OTf^−^ + **9**]^+^ species (calculated *m*/*z* 1470.4516). It confirmed that chiral Lewis acid bonded 2-picolinaldehyde condenses with methyl glycine ester to generate the metal-chelated Schiff base **I** (Fig. 1).

Based on the aforementioned results, a plausible catalytic cycle is rationalized for the current reaction (Fig. 1). Initially, chiral ytterbium-bonded picolinaldehyde reacts with glycine ester to yield chiral cation bonded Schiff base species **I**. In view of the large and variable coordination number of the ytterbium ion, the tridentate coordination of Schiff base **9** and another anion is anticipated to enhance the stability and reactivity of the corresponding enolate species, as well as the quinonoid tautomer. Then, H-bond activated aldimine species undergoes the Mannich reaction *via* the enolate intermediate **II** to afford the intermediate **IV**. Then, hydrolysis of the intermediate **IV** produces the chiral diamine **6**, regenerating the active catalytic species **Ligand**/Yb^III^ bonded-picolinaldehyde. Eventually, the final product imidazolidine **3** was yielded by the condensation of vicinal diamine and aldehyde.

**Fig. 2:**
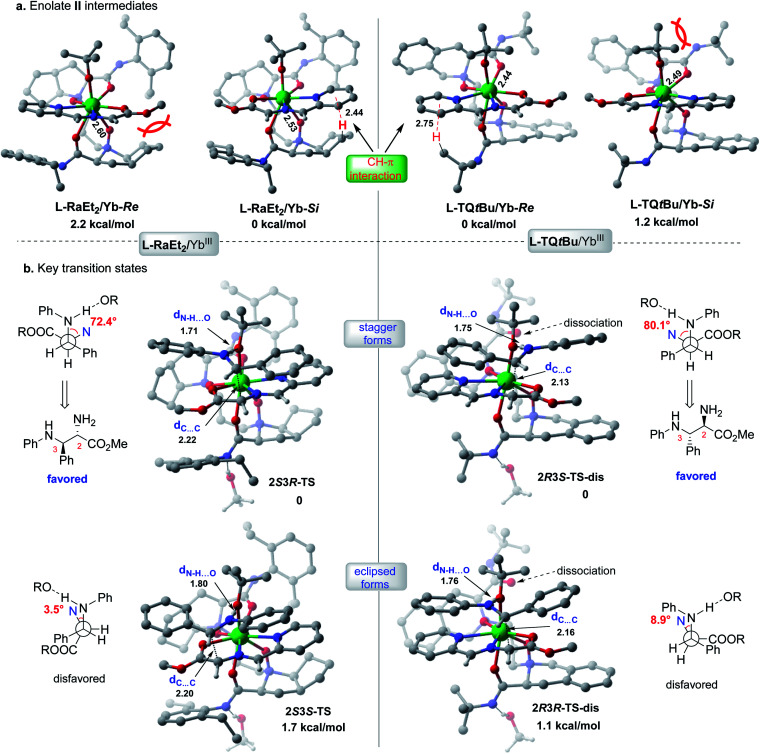
Calculated stereo-models of transition states and intermediates for **L-RaEt2/YbIII** or **L-TQtBu/YbIII** complexes.

The X-ray crystal structures of the chiral Lewis acid complexes **L-RaEt2**/Yb(OTf)_3_ and **L-TQtBu**/Yb(OTf)_3_ (ref. 22) provide interesting spatial information (see ESI Fig. S2 and S3† for full pictures), which might account for the enantiodivergent outcomes. To unveil the different stereochemical control of **L-RaEt2** (ref. 23) and **L-TQtBu** in the formation of chiral intermediate **IV**, preliminary density functional theory (DFT) calculations were performed at the SMD-B3LYP-D3/MWB59/6-311+G(d,p) level of theory. Due to different thermodynamic stabilities, ^−^O*t*Bu is more favorable to coordinate with Yb^III^ for about −10.2 kcal mol^−1^ rather than the triflate anion. Moreover, based on the binding orientation of the tridentate Schiff base **9** in different Yb^III^-chiral *N*,*N*′-dioxides complexes, ^−^O*t*Bu always locates at the *Si*-face or *Re*-face site of the Schiff base for **L-RaEt2/**Yb^III^ or **L-TQtBu/**Yb^III^, respectively, which would block their opposite sites by substrate/ligand interactions and avoid the steric hindrance of the bulky ligand (bicycle or *tert*-butyl amide subunits). As shown in **L-RaEt2/**Yb^III^ of Fig. 2a, the steric effect of the bicycle ring on the ligand would lead to relatively weak coordination 2.60 Å between ligands O^N^ and Yb^III^ in **L-RaEt2/Yb-Re**, and thus its thermodynamic free energy is 2.2 kcal mol^−1^ higher than that of **L-RaEt2/Yb-Si**. On the other hand, the *tert*-butyl amide in **L-TQtBu/**Yb^III^ displays major steric hindrance with ^−^O*t*Bu that results in unfavorable coordination in **L-TQtBu/Yb-Si**.

Further discussion will depend on the most favorable Yb^III^/chiral *N*,*N*′-dioxide complexes with the Schiff base substrate and ^−^O*t*Bu ligand (Fig. 2b). To control the stereoselectivity, an efficient inner-sphere hydrogen bond (around 1.7 Å) between the coordinated ^−^O*t*Bu and protonated aldimine would drive the nucleophilic attacking to form stable transition states in the following Mannich reaction, compared to the outer-sphere hydrogen bond interaction that affords 6.1 kcal mol^−1^ free energy higher in the transition state **2S3R-TS_1** (see ESI Table S10†). For both the **L-RaEt2/**Yb^III^ and **L-TQtBu/**Yb^III^ complexes, the stagger forms of transition states were favored *via Si*–*Si* (**2S3R-TS**) and *Re*–*Re* (**2R3S-TS-dis**) facial selectivity, resulting in (2*S*,3*R*)-**6a** and (2*R*,3*S*)-**6a** respectively, which are in agreement with experimental observations (Fig. 2b). In contrast, the eclipsed forms of transition states *via Si*–*Re* and *Re*–*Si* facial attacking were calculated to be about 1–2 kcal mol^−1^ energy higher than those of the most stable ones, indicating the important substrate interactions between the Schiff base and protonated aldimine under each chiral space, which account for the diastereoselectivity. Furthermore, the transient ligand dissociation of the **L-TQtBu**/Yb^III^ complex at one carbonyl group of amide was inspected in the most stable transition states **2R3S-TS-dis** and **2R3R-TS-dis***via Re*–*Re* and *Re*–*Si* facial selectivities, respectively, and the relative free energies are slightly favorable to the full coordinated transition states (see ESI Fig. S5–S7†), suggesting that the large steric hindrance of transition states in this ligand would reduce the coordination number also supported by the crystal structure of **L-TQtBu**/Yb(OTf)_3_. However, the calculated ligand dissociation can be recovered when the protonated aldimine is released from the Yb^III^ center displaying dynamic coordination.

## Conclusions

In summary, a new asymmetric carbonyl-catalysis strategy was developed for the Mannich/condensation of glycine ester with aldimine under mild conditions. The chiral *N*,*N*′-dioxide/Yb(OTf)_3_ complex bonded aldehyde enabled carbonyl activation of glycine ester for α-addition transformation. This protocol provided facile and feasible access to a variety of synthetically useful chiral imidazolidine derivatives in moderate yields (up to 66% yield), and excellent diastereo- and enantioselectivities (up to 95 : 5 dr, 97% ee). The reaction could be performed in either two- or three-component versions. Interestingly, enantiodivergent synthesis was accessible by modulating the sub-structure of the Feng *N*,*N*′-dioxide ligand in connection with a multi-coordinated ytterbium metal ion. Theoretical calculations suggested that the steric hindrance and CH–π interaction between the substrate and ligand were responsible for this reversal. And the inner-sphere hydrogen bond and the stagger model of reaction substrates enable stabilization of key transition states leading to high stereoselectivity. Further application of this co-catalytic system in other reactions is under investigation.

## Conflicts of interest

There are no conflicts to declare.

## Supplementary Material

SC-012-D0SC07052A-s001

SC-012-D0SC07052A-s002
