# Supplementary material for: Chiral Lewis acid-bonded picolinaldehyde enables enantiodivergent carbonyl catalysis in the Mannich/condensation reaction of glycine ester
Source: Chem Sci. 2021 Jan 26;12(12):4353–60. doi: 10.1039/d0sc07052a (PMC8179594; doi:10.1039/d0sc07052a)
Supplement: SC-012-D0SC07052A-s001 [file SC-012-D0SC07052A-s001.pdf]

Electronic Supplementary Material (ESI) for Chemical Science.
This journal is © The Royal Society of Chemistry 2021

Chiral Lewis Acid-Bonded Picolinaldehyde Enables Enantiodivergent Carbonyl Catalysis in Mannich/Condensation Reaction of Glycine Ester

Xia Zhong, Ziwei Zhong, Zhikun Wu, Zhen Ye, Yuxiang Feng, Shunxi Dong, Xiaohua Liu, * Qian Peng * and Xiaoming Feng *

Table of Contents

(A) General Information 3

(B) Characterization of *N,N′*-Dioxide Ligands 3

(C) General Procedure for the Catalytic Asymmetric Cascade Mannich/condensation Reaction 3

(D) Optimization of Conditions 4

(E) X-ray Crystal Structure of Product **3n** and *N,N′*-Dioxide/Yb(OTf)$_3$ Complexes 11

(F) Experimental Procedure for the Scale-up Reaction and Transformations of the Products 15

(G) Procedure for React IR Experiment for Kinetic Studies 19

(H) Control Experiments and HRMS Analysis 22

(I) IR Analysis and Deuterium Labeling Experiment 28

(J) HRMS Analysis 30

(K) Computational Details 30

(L) References 55

(M) The Analytical and Spectral Characterization Data of the Products 56

(N) Copies of NMR Spectra 75

(O) Copies of CD Spectra in CH_2Cl_2 123

(A) General Information

CH_2Cl_2, CH_2ClCH_2Cl, EtOAc, MeOAc, *n*PrOAc, MeCN were freshly distilled from CaH_2 prior to use; THF, toluene, Et_2O were freshly distilled from sodium metal prior to use; alcohols were freshly distilled from magnesium metal/I_2 prior to use; aldehydes and amines were distilled or recrystallized to use. ^{1}H NMR spectra were recorded on bruker ASCEND^{TM} operating at 400 MHz. The chemical shifts were recorded in ppm relative to tetramethylsilane and with the solvent resonance as the internal standard. Data were reported as follows: chemical shift, multiplicity (s = singlet, d = doublet, t = triplet, m = multiplet, q = quartet), coupling constants (Hz), integration. ^{13}C{^{1}H} NMR data were collected bruker ASCEND^{TM} operating at 101 MHz with complete proton decoupling. ^{19}F{^{1}H} NMR was collected bruker ASCEND^{TM} operating at 376 M Hz with complete proton decoupling. Metal salts obtained from commercial sources were used without further purification. Enantiomeric excesses were determined by HPLC analysis on Daicel Chiralcel IA/IB/IC/IF/IG/IH/ASH in comparison with the authentic racemates. Optical rotations were reported as follows: $[\alpha]^{T}_{D}$ = (*c* = g/100 mL, in solvent, unless otherwise noted, λ = 436 nm). HRMS was recorded on Thermo Q-Exactive Focus (FTMS+c ESI) and data were reported as (m/z). The chiral *N,N′*-dioxide ligands were synthesized by the same procedure in the literature.[1] The imine compounds were prepared according to the literature.[2] Amino acid ester hydrochlorides were commercial reagent and recrystallized from MeOH/CH_2Cl_2. Unless noted, other commercial reagents were used without further purification.

(B) Characterization of *N,N′*-Dioxide Ligands

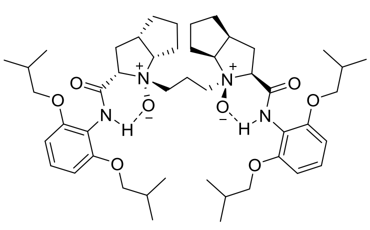

L-Ra(O^{i}Bu)$_2$

L-Ra(OiBu)$_2$ White solid; R_f = 0.3 (ethyl acetate/methanol = 10/1). $[\alpha]^{27}_{D}$ **=** −24.3 (*c* = 0.54, in tetrahydrofuran, λ = 365 nm); **m.p.:** 107.6 – 116.8 ºC; **^{1}H NMR** (400 MHz, $CDCl_3$) δ 12.37 (s, 2H), 7.12 – 7.05 (m, 2H), 6.52 – 6.46 (m, 4H), 3.99 – 3.92 (m, 2H), 3.85 – 3.78 (m, 2H), 3.71 – 3.61 (m, 10H), 3.25 – 3.14 (m, 2H), 2.79 – 2.66 (m, 4H), 2.64 – 2.53 (m, 4H), 2.47 – 2.35 (m, 2H), 2.05 – 1.96 (m, 6H), 1.76 – 1.57 (m, 8H), 0.97 (d, *J* = 6.8, 1.3 Hz, 24H) ppm; **^{13}C{^{1}H} NMR** (101 MHz, $CDCl_3$) δ 165.3, 155.0, 127.2, 114.0, 104.7, 83.4, 81.1, 74.7, 65.7, 42.5, 34.9, 32.3, 28.4, 27.9, 27.0, 19.4 ppm; **IR (neat) ν (cm^{-1})**: 2953, 2359, 1680, 1592, 1527, 1459, 1393, 1257, 1097, 952, 752, 720, 650; **HRMS** (ESI-FT) calcd for $C_{47}H_{73}N_4O_8^+$ ([M]+H$^+$) = 821.5423, found 821.5416.

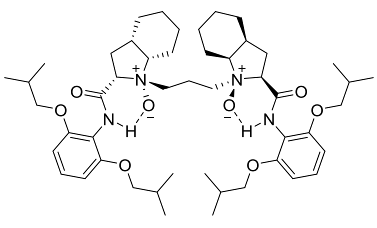

L-Pe(O^{i}Bu)$_2$

L-Pe(OiBu)$_2$ White solid; R_f = 0.3 (ethyl acetate/methanol = 10/1). $[\alpha]^{23}_{D}$ = −18.4 (*c* = 0.32, in tetrahydrofuran, λ = 365 nm); **m.p.:** 89.0 – 97.8 ºC; **^{1}H NMR** (400 MHz, $CDCl_3$) δ12.67 (s, 1H), 7.10 – 7.05 (m, 2H), 6.52 – 6.49 (m, 4H), 3.72 (d, *J* = 6.4 Hz, 8H), 3.66 – 3.55 (m, 4H), 3.41 – 3.30 (m, 2H), 2.63 – 2.54 (m, 2H), 2.44 – 2.29 (m, 4H), 2.24 – 2.13 (m, 4H), 2.07 – 2.01 (m, 4H), 1.84 – 1.76 (m, 4H), 1.70 – 1.60 (m Hz, 4H), 1.28 – 1.22 (m, 4H), 1.00 (dd, *J* = 6.8, 1.6 Hz, 24H), 0.96 – 0.92 (m, 4H) ppm; **^{13}C{^{1}H} NMR** (101 MHz, $CDCl_3$) δ 166.5, 154.9, 127.0, 114.1, 104.7, 74.9, 74.0, 63.9, 34.5, 33.9, 28.4, 28.3, 21.1, 20.7, 20.1, 19.8, 19.4, 19.3 ppm; **IR (neat) ν (cm^{-1})**: 2954, 2336, 1682, 1596, 1529, 1464, 1392, 1255, 1102, 719, 594; **HRMS** (ESI-FT) calcd for $C_{49}H_{77}N_4O_8^+$ ([M]+H$^+$) = 849.5736, found 849.5745.

(C) General Procedure for the Catalytic Asymmetric Cascade Mannich/condensation Reaction

1 Typical procedure for the asymmetric cascade Mannich/condensation reaction

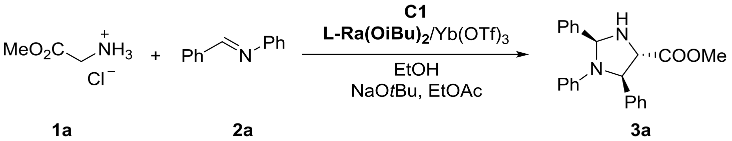

1a **2a** **3a**

Procedure A: The reaction was conducted with $Yb(OTf)_3$ (6.2 mg, 10 mol%), **L-Ra(OiBu)$_2$** (8.2 mg, 10 mol%), NaO*t*Bu (9.6 mg, 0.10 mmol), methyl glycine hydrochloride **1a** (12.6 mg, 0.10 mmol), and EtOH (5.0 μL). Picolinaldehyde (**C1**, 2.0 μL, 20 mol%) and EtOAc (0.60 mL) were added in succession. The mixture was stirred at 35 °C for 15 min, and imine **2a** (54.0 mg, 0.30 mmol) was added at 35 °C. The resulting mixture was stirred at 35 °C for 48 hours. The reaction mixture was subjected to column chromatography on silica gel and eluted with petroleum ether/ethyl acetate (10/1 to 6/1, v/v) to afford the corresponding product **3a**.

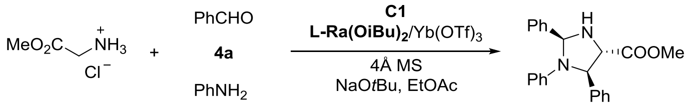

1a **4a** **5a** **3a**

Procedure B: The reaction was conducted with $Yb(OTf)_3$ (6.2 mg, 10 mol%), **L-Ra(OiBu)$_2$** (8.2 mg, 10 mol%), NaO*t*Bu (9.6 mg, 0.10 mmol), methyl glycine hydrochloride **1a** (12.6 mg, 0.10 mmol), and 4Å MS (20 mg). Picolinaldehyde (**C1**, 2.0 μL, 20 mol%) and EtOAc (0.60 mL) were added in succession. The mixture was stirred at 35 °C for 15 min, and aldehyde **4** (0.30 mmol), amine **5** (0.30 mmol) were added at 35 °C. The resulting mixture was stirred at 35 °C for 48 hours. The reaction mixture was subjected to column chromatography on silica gel and eluted with petroleum ether/ethyl acetate (10/1 to 6/1, v/v) to afford the corresponding product **3a**.

2 Typical procedure for the racemic product.

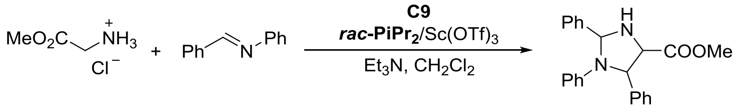

1a **2a** ***rac*-3a**

Procedure A: The reaction was conducted with $Sc(OTf)_3$ (4.9 mg, 10 mol%), ***rac*-PiPr$_2$** (6.5 mg, 10 mol%), Et_3N (15.5 μL, 0.11 mmol), and methyl glycine hydrochloride **1a** (12.6 mg, 0.10 mmol). CH_2Cl_2 (0.60 mL) and pyridoxal hydrochloride (**C9**, 2.0 mg, 10 mol%) were added in succession. The mixture was stirred at 35 °C for 15 min, and imine **2a** (36.2 mg, 0.20 mmol) was added at 35 °C. The resulting mixture was stirred at 35 °C for 24 hours. The reaction mixture was subjected to column chromatography on silica gel and eluted with petroleum ether/ethyl acetate (10/1 to 6/1, v/v) to afford the corresponding product ***rac*-3a**.

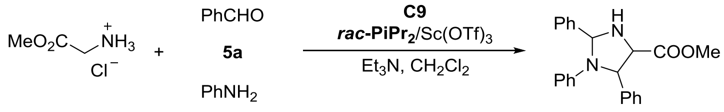

1a **6a** ***rac*-3a**

Procedure B: The reaction was conducted with $Sc(OTf)_3$ (4.9 mg, 10 mol%), ***rac*-PiPr$_2$** (6.5 mg, 10 mol%), Et_3N (15.5 μL, 0.11 mmol), and methyl glycine hydrochloride **1a** (12.6 mg, 0.10 mmol). CH_2Cl_2 (0.60 mL) and pyridoxal hydrochloride (**C9**, 2.0 mg, 10 mol%) were added in succession. The mixture was stirred at 35 °C for 15 min, and aldehyde **5a** (20.0 μL, 0.20 mmol), amine **6a** (18.0 μL, 0.20 mmol) were added at 35 °C. The resulting mixture was stirred at 35 °C for 24 hours. The reaction mixture was subjected to column chromatography on silica gel and eluted with petroleum ether/ethyl acetate (10/1 to 6/1, v/v) to afford the corresponding product ***rac*-3a**.

(D) Optimization of Conditions

Table S1. Screening of carbonyl catalyst[a]

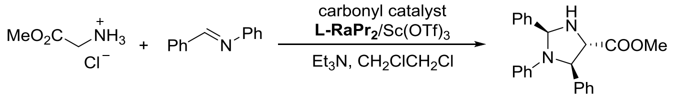

1a **2a** **3a**

carbonyl catalyst:

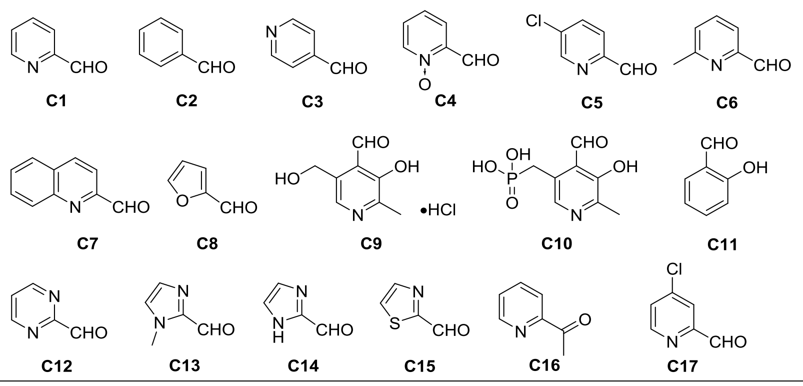

entry	carbonyl catalyst	yield (%)[b]	dr[c]	ee (%)[c]
1	**C1**	10	78:22	23/49
2	**C2**	N.R.	--	--
3	**C3**	N.R.	--	--

4	**C4**	19	75:25	5/racemic
5	**C5**	6	72:28	20/37
6	**C6**	3	76:24	racemic
7	**C7**	13	84:16	racemic
8[d]	**C8**	N.R.	--	--
9	**C9**	24	66:34	11/9
10	**C10**	14	66:34	racemic
11	**C11**	N.R.	--	--
12	**--**	N.R.	--	--

[a] Unless otherwise noted, all reactions were carried out with **1a** (0.10 mmol), **2a** (0.20 mmol), Et_3N (0.10 mmol), carbonyl catalyst (10 mol%) and **L-RaPr$_2$**/$Sc(OTf)_3$ (1:1, 10 mol%) in CH_2ClCH_2Cl (0.60 mL) at 35 °C for 24 h. [b] Isolated yield of **3a** based on **1a**. [c] Determined by HPLC on a chiral stationary phase. [d] Et_3N (0.11 mmol) was used. [e] The reactions were carried out with **1a** (0.10 mmol), **2a** (0.20 mmol), NaO*t*Bu (0.10 mmol), carbonyl catalyst (10 mol%) and **L-Ra(OiBu)$_2$**/$Yb(OTf)_3$ (1:1, 10 mol%) in EtOAc (0.60 mL) at 35 °C for 24 h.

Table S2. Screening of metal salts[a]

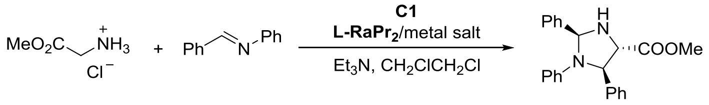

entry	metal salt	yield (%)[b]	dr[c]	ee (%)[c]
1	$Mg(OTf)_2$	2	68:32	7/20
2	$Sc(OTf)_3$	8	78:22	23/49
3	$Fe(OTf)_3$	5	57:43	racemic
4	$Ni(OTf)_2$	< 2	66:34	racemic
5	$Cu(OTf)_2$	4	52:48	racemic
6	$Zn(OTf)_2$	14	53:47	racemic
7	$Y(OTf)_3$	34	71:29	48/35
8	$La(OTf)_3$	28	63:37	58/21
9	$Nd(OTf)_3$	16	65:35	55/63
10	$Sm(OTf)_3$	20	67:33	60/57
11	$Eu(OTf)_3$	28	64:36	56/57
12	$Gd(OTf)_3$	36	70:30	54/39
13	$Dy(OTf)_3$	18	64:36	54/52
14	$Ho(OTf)_3$	41	69:31	43/35
15	$Er(OTf)_3$	29	65:35	47/46
16	$Tm(OTf)_3$	18	75:25	23/37
**17**	**$Yb(OTf)_3$**	**31**	**72:28**	**59/35**
18	$Lu(OTf)_3$	26	69:31	40/37
19[d]	$Cu(OTf)_2$	N.R.	--	--
20[d]	$Zn(OTf)_2$	15	68:32	racemic
21[d]	$Sc(OTf)_3$	13	68:32	27/21
22[d]	$Y(OTf)_3$	3	80:20	96

23[d]	$Dy(OTf)_3$	7	81:19	96
24[d]	$Lu(OTf)_3$	9	81:19	94

[a] Unless otherwise noted, all reactions were carried out with **1a** (0.10 mmol), **2a** (0.20 mmol), Et_3N (0.10 mmol), **C1** (10 mol%) and **L-RaPr$_2$**/metal salt (1:1, 10 mol%) in CH_2ClCH_2Cl (0.60 mL) at 35 °C for 24 h. [b] Isolated yield of **3a** based on **1a**. [c] Determined by HPLC on a chiral stationary phase. [d] The reactions were carried out with **1a** (0.10 mmol), **2a** (0.20 mmol), NaO*t*Bu (0.10 mmol), **C16** (20 mol%) and **L-Ra(OiBu)$_2$**/ metal salt (1:1, 10 mol%) in EtOAc (0.60 mL) at 35 °C for 24 h.

Table S3. Screening of ligands[a]

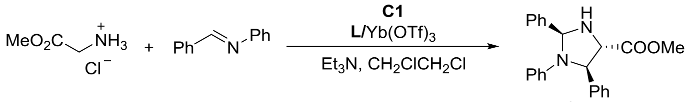

1a **2a** **3a**

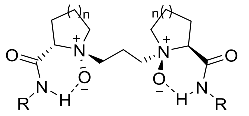

L-PrPr$_2$: R = 2,6-iPr$_2$C$_6$H$_3$, n = 1
L-Pr(OiBu)$_2$: R = 2,6-(iBuO)$_2$C$_6$H$_3$, n = 1
L-PiPr$_2$: R = 2,6-iPr$_2$C$_6$H$_3$, n = 2
L-Pi(OiBu)$_2$: R = 2,6-(iBuO)$_2$C$_6$H$_3$, n = 2

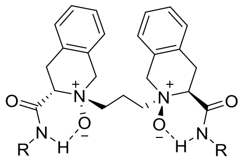

L-TQ*t*Bu: R = *t*Bu
L-TQ*s*EPh: R = (*S*)-2-phenylerhyl
L-TQAd: R = 1-adamantyl
L-TQPh: R = C$_6$H$_5$

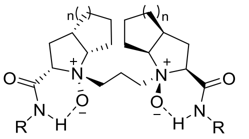

L-RaPr$_2$: R = 2,6-iPr$_2$C$_6$H$_3$, n = 1
L-RaEt$_2$: R = 2,6-Et$_2$C$_6$H$_3$, n = 1
L-RaEt$_2$Me: R = 2,6-Et$_2$-4-MeC$_6$H$_2$, n = 1
L-Ra(OiBu)$_2$: R = 2,6-(iBuO)$_2$C$_6$H$_3$, n = 1
L-RaCy: R = C$_6$H$_{11}$, n = 1
L-RaAd: R = 1-adamantyl, n = 1
L-RaBn: R = CH$_2$C$_6$H$_5$, n = 1
L-RaCHPh$_2$: R = CH(C$_6$H$_5$)$_2$, n = 1
L-PePr$_2$: R = 2,6-iPr$_2$C$_6$H$_3$, n = 2
L-Pe(OiBu)$_2$: R = 2,6-(iBuO)$_2$C$_6$H$_3$, n = 2

entry	L	yield (%)[b]	dr[c]	ee (%)[c]
1	**L-PrPr$_2$**	32	64:36	4/3
2	**L-PiPr$_2$**	36	72:28	53/29
3	**L-Pi(OiBu)$_2$**	39	77:23	73/61
**4**	**L-TQ*t*Bu**	**43**	**75:25**	**−80/−75**
5	**L-RaPr$_2$**	31	72:28	59/35
6	**L-RaEt$_2$**	33	66:34	67/59
7	**L-RaEt$_2$Me**	25	72:28	75/71
**8**	**L-Ra(OiBu)$_2$**	**43**	**74:26**	**81/76**
9	**L-RaCy**	5	66:34	63/70
10	**L-RaAd**	< 2	64:36	38/33
11	**L-RaBn**	13	70:30	76/74
12	**L-RaCHPh$_2$**	16	73:27	81/79
13	**L-PePr$_2$**	22	71:29	21/25
14	**L-TQ*s*EPh**	23	64:36	−74/−75
15	**L-TQAd**	24	68:32	−75/−90
16	**L-TQPh**	29	75:25	−59/−69
17[d]	**L-Pi(OiBu)$_2$**	9	89:11	88
18[d]	**L-Pe(OiBu)$_2$**	4	83:17	71
19[d]	**L-RaCHPh$_2$**	24	78:22	69/60

20[d]	**L-RaPr$_2$**	10	73:27	37/37
21[d]	**L-RaEt$_2$**	3	78:22	71/79

[a] Unless otherwise noted, all reactions were carried out with **1a** (0.10 mmol), **2a** (0.20 mmol), Et_3N (0.10 mmol), **C1** (10 mol%) and **L**/Yb(OTf)$_3$ (1:1, 10 mol%) in CH_2ClCH_2Cl (0.60 mL) at 35 °C for 24 h. [b] Isolated yield of **3a** based on **1a**. [c] Determined by HPLC on a chiral stationary phase. [d] The reactions were carried out with **1a** (0.10 mmol), **2a** (0.20 mmol), NaO*t*Bu (0.10 mmol), **C16** (20 mol%) and **L**/ Yb(OTf)$_3$ (1:1, 10 mol%) in EtOAc (0.60 mL) at 35 °C for 24 h.

Table S4. Screening of solvent[a]

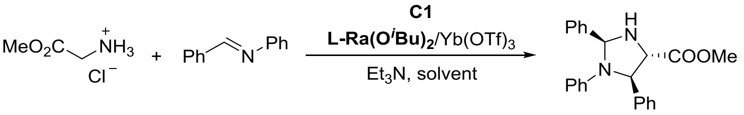

1a **2a** **3a**

entry	solvent	yield (%)[b]	dr[c]	ee (%)[c]
1	CH_2ClCH_2Cl	43	74:26	81/76
2	THF	42	68:32	90/89
3	MeOH	55	71:29	84/71
4	MeCN	38	61:39	27/27
5	Et_2O	32	75:25	86/90
6	toluene	34	72:28	92/87
7	MeOAc	44	73:27	82/71
**8**	**EtOAc**	**40**	**72:28**	**93/92**
9	^{n}PrOAc	39	75:25	91/85
10[d]	toluene	4	83:17	74
11[d]	MeOH	N.R.	--	--
12[d]	CH_2Cl_2	7	90:10	90
13[d]	^{n}PrOAc	20	88:12	91

[a] Unless otherwise noted, all reactions were carried out with **1a** (0.10 mmol), **2a** (0.20 mmol), Et_3N (0.10 mmol), **C1** (10 mol%) and **L-Ra(OiBu)$_2$**/Yb(OTf)$_3$ (1:1, 10 mol%) in solvent (0.60 mL) at 35 °C for 24 h. [b] Isolated yield of **3a** based on **1a**. [c] Determined by HPLC on a chiral stationary phase. [d] The reactions were carried out with **1a** (0.10 mmol), **2a** (0.20 mmol), NaO*t*Bu (0.10 mmol), **C16** (20 mol%) and **L-Pi(OiBu)$_2$**/Yb(OTf)$_3$ (1:1, 10 mol%) in solvent (0.60 mL) at 35 °C for 24 h.

Table S5. Screening of bases[a]

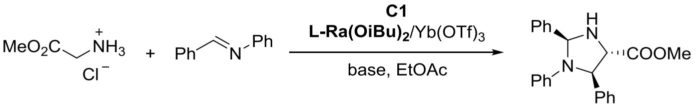

1a **2a** **3a**

entry	base	yield (%)[b]	dr[c]	ee (%)[c]
1	Et_3N	40	72:28	93/92
2	*n*Pr$_3$N	34	70:30	57/59
3	iPr$_2$NH	30	70:30	91/91
4	DMAP	34	77:23	73/80
5	DBU	5	71:29	49/55
6	DABCO	7	78:22	95/85
7	TMDEA	19	78:22	95/89
8	*n*Bu$_4$NBr	4	58:42	65/47

9	Na_2CO_3	19	88:12	93
10	Na_3PO_4	8	68:32	79/80
11	NaOH	46	83:17	96/95
12	NaOAc	32	81:19	72/24
13	NaOMe	33	83:17	91/99
14	NaOEt	39	82:18	95/99
**15**	**NaO*t*Bu**	**40**	**93:7**	**95/91**
16	LiO*t*Bu	37	77:23	43/49
17	KO*t*Bu	11	93:7	94
18	--	N.R.		
19[d]	NaO*t*Bu	30	77:23	68/65
20[e]	NaO*t*Bu	37	94:6	96
21[f]	NaO*t*Bu	4	95:5	96
22[g]	NaO*t*Bu	43	90:10	91
23[h]	NaO*t*Bu	41	90:10	70
23[i]	NaO*t*Bu	36	92:8	97
**24[j]**	**NaO*t*Bu**	**34**	**86:14**	**−87/−90**
25[k]	NaO*t*Bu	42	88:12	92/90
26[l]	NaO*t*Bu	7	70:30	racemic
27[m]	Et_3N	N.R.	--	--
28[m]	DMAP	N.R.	--	--
29[m]	Na_2CO_3	N.R.	--	--

[a] Unless otherwise noted, all reactions were carried out with **1a** (0.10 mmol), **2a** (0.20 mmol), base (0.10 mmol), **C1** (10 mol%) and **L-Ra(OiBu)$_2$**/Yb(OTf)$_3$ (1:1, 10 mol%) in EtOAc (0.60 mL) at 35 °C for 24 h. [b] Isolated yield of **3a** based on **1a**. [c] Determined by HPLC on a chiral stationary phase. [d] NaO*t*Bu (0.05 mmol) was used. [e] NaO*t*Bu (0.11 mmol) was used. [f] NaO*t*Bu (0.15 mmol) was used. [g] **L-Pi(OiBu)$_2$** was used instead of **L-Ra(OiBu)$_2$**. [h] **L-Pr(OiBu)$_2$** was used instead of **L-Ra(OiBu)$_2$**. [i] **L-Pe(OiBu)$_2$** was used instead of **L-Ra(OiBu)$_2$**. [j] **L-TQ*t*Bu** was used instead of **L-Ra(OiBu)$_2$**. [k] **L-RaEt$_2$** was used instead of **L-Ra(OiBu)$_2$**. [l] Ligand was absented. [m] The reactions were carried out with **1a** (0.10 mmol), **2a** (0.20 mmol), base (0.10 mmol), **C16** (20 mol%) and **L-Pi(OiBu)$_2$**/Yb(OTf)$_3$ (1:1, 10 mol%) in EtOAc (0.60 mL) at 35 °C for 24 h.

Table S6. Screening of the amount of C1[a]

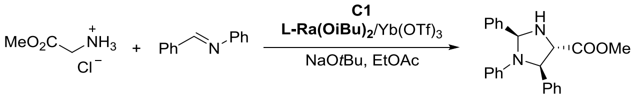

1a **2a** **3a**

entry	amount of **C1** (mol%)	yield (%)[b]	dr[c]	ee (%)[c]
1	0	N.R.		
2	5	10	81:19	77/30
3	10	40	93:7	95
**4**	**20**	**44**	**93:7**	**95/97**
5	50	30	91:9	93
6	100	3	65:35	race/race
7[d]	20	21	91:9	96
8[e]	20	47	83:17	93/87

9[f]	20	35	93:7	96
10[g]	20	43	91:9	96

[a] Unless otherwise noted, all reactions were carried out with **1a** (0.10 mmol), **2a** (0.20 mmol), NaO*t*Bu (0.10 mmol), **C1**, and **L-Ra(OiBu)$_2$**/Yb(OTf)$_3$ (1:1, 10 mol%) in EtOAc (0.60 mL) at 35 °C for 24 h. [b] Isolated yield of **3a** based on **1a**. [c] Determined by HPLC on a chiral stationary phase. [d] EtOAc (1.0 mL) was used. [e] EtOAc (0.20 mL) was used. [f] The reaction was carried out at 30 °C. [g] The reaction was carried out at 40 °C.

Table S7. Screening of additives[a]

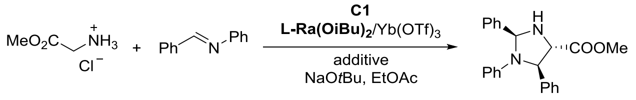

1a **2a** **3a**

entry	additive	yield (%)[b]	dr[c]	ee (%)[c]
1	10 µL (24.0 equiv) MeOH	40	89:11	97
2	10 µL (16.0 equiv) EtOH	44	93:7	96
3	10 µL (10.0 equiv) *t*BuOH	42	90:10	98
4	10 µL (55.0 equiv) H_2O	37	86:14	97
5	10 mol% PhCOOH	39	93:7	97
6	1.0 equiv PhCHO	48	84:16	96/90
7	1.0 equiv $PhNH_2$	39	88:12	94
8	30 mol% $NaBAr^F_4$	51	84:16	78/74
9	30 mol% $LiNTf_2$	56	82:18	87/79
10	30 mg 3Å MS	38	84:16	91/80
11	30 mg 4Å MS	49	93:7	96
12	30 mg 5Å MS	46	88:12	93
13	10 mg 4Å MS	52	91:9	96
14	20 mg 4Å MS	51	93:7	96
15	100 mg 4Å MS	45	93:7	97
**16**	**5 µL (8.0 equiv) EtOH**	**46**	**94:6**	**97/97**
17	50 µL (80.0 equiv) EtOH	44	93:7	97
18[d]	5 µL (8.0 equiv) EtOH	48	93:7	97
19[e]	5 µL (8.0 equiv) EtOH	50	93:7	96
**20[d,f]**	**5 µL (8.0 equiv) EtOH**	**62**	**95:5**	**97/99**
21[e,f]	5 µL (8.0 equiv) EtOH	61	92:8	95

[a] Unless otherwise noted, all reactions were carried out with **1a** (0.10 mmol), **2a** (0.20 mmol), NaO*t*Bu (0.10 mmol), **C1** (20 mol%), additive and **L-Ra(O*i*Bu)$_2$**/Yb(OTf)$_3$ (1:1, 10 mol%) in EtOAc (0.60 mL) at 35 °C for 24 h. [b] Isolated yield of **3a** based on **1a**. [c] Determined by HPLC on a chiral stationary phase. [d] **2a** (0.30 mmol) was used. [e] **2a** (0.40 mmol) was used. [f] The reaction was carried out for 48 h.

Table S8. Re-screening of carbonyl catalyst[a]

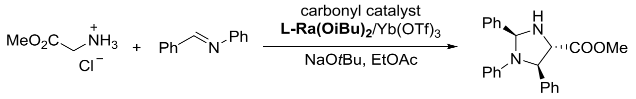

1a **2a** **3a**

entry	additive	yield (%)[b]	dr[c]	ee (%)[c]
1	**C1**	40	93:7	95

2	**C2**	N.R.	--	--
3	**C3**	N.R.	--	--
4	**C4**	45	81:19	9
5	**C5**	45	85:15	94
6	**C6**	11	92:8	90
7	**C9**	27	75:25	31/20
8	**C12**	10	93:7	97
9	**C13**	22	90:10	83
10	**C14**	14	90:10	91
11	**C15**	3	95:5	95
12	**C16**	8	84:16	94
13	**C17**	44	83:17	94

[a] Unless otherwise noted, all reactions were carried out with **1a** (0.10 mmol), **2a** (0.20 mmol), NaO*t*Bu (0.10 mmol), carbonyl catalyst (10 mol%) and **L-Ra(OiBu)$_2$**/Yb(OTf)$_3$ (1:1, 10 mol%) in EtOAc (0.60 mL) at 35 °C for 24 h. [b] Isolated yield of **3a** based on **1a**. [c] Determined by HPLC on a chiral stationary phase.

Table S9. Screening of three-component reaction[a]

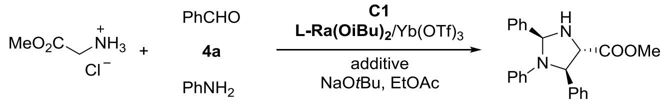

1a **5a** **3a**

entry	additive	amount of **4a** (mmol)	amount of **5a** (mmol)	yield (%)[b]	dr[c]	ee (%)[c]
1	20 mg 4Å MS	0.20	0.20	42	95:5	97
2	20 mg 4Å MS	0.30	0.30	45	94:6	97
3	20 mg 4Å MS	0.20	0.30	39	95:5	97
4	20 mg 4Å MS	0.30	0.20	48	86:14	97
5	20 mg 4Å MS	0.60	0.30	51	80:20	95
6	5 μL (8.0 equiv) EtOH	0.20	0.20	31	93:7	96
**7[d]**	**20 mg 4Å MS**	**0.30**	**0.30**	**55**	**95:5**	**96/97**

[a] Unless otherwise noted, all reactions were carried out with **1a** (0.10 mmol), **4a** (x mmol), **5a** (x mmol), NaO*t*Bu (0.10 mmol), **C1** (20 mol%), additive and **L-Ra(OiBu)$_2$**/Yb(OTf)$_3$ (1:1, 10 mol%) in EtOAc (0.60 mL) at 35 ºC for 24 h. [b] Isolated yield of **3a** based on **1a**. [c] Determined by HPLC on a chiral stationary phase. [d] The reaction was carried out for 48 h.

Table S10. Unsuccessful examples

1. Failed examples

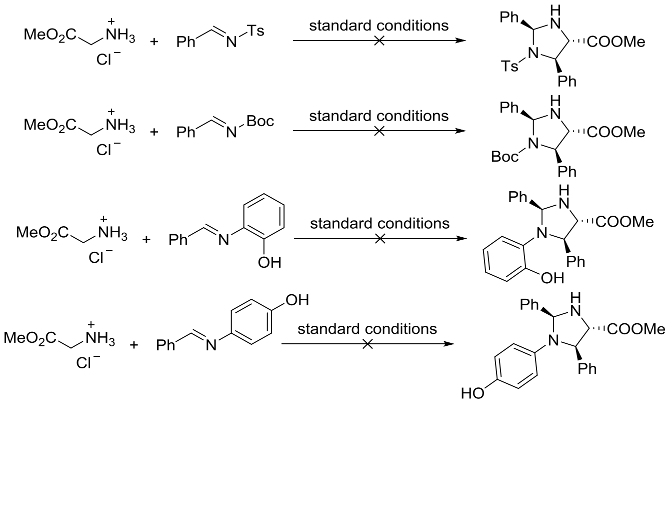

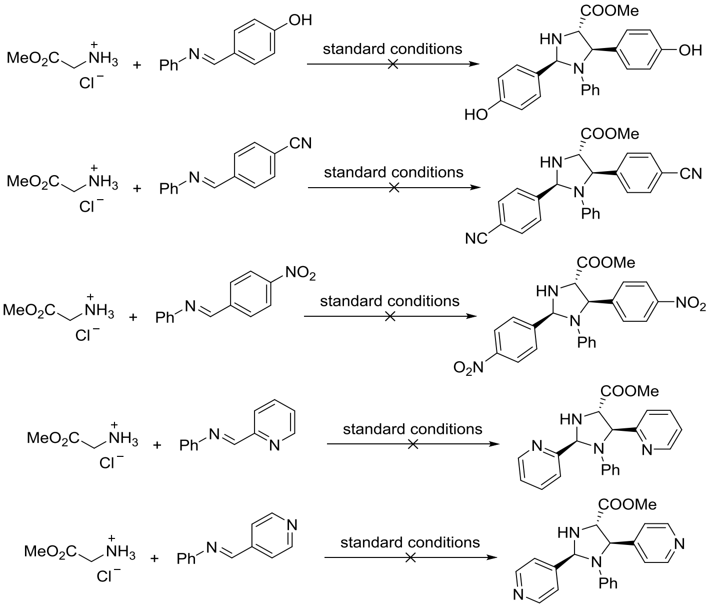

Alkyl imines were ineffective caused the alkyl imines don't have conjugation stabilization. Thus they are relatively unstable as compared to aromatic imines and easily hydrolyzed under the reaction condition.[3]

2. Racemic examples

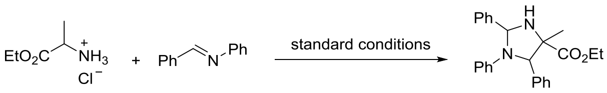

3ala
13% yield, >95:5 dr, racemic

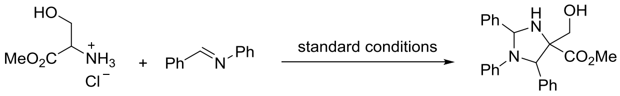

3ser
13% yield, 86:14 dr, racemic

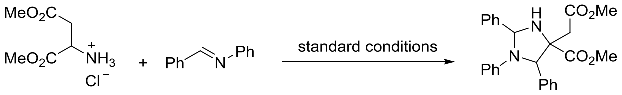

3asp
11% yield, 94:6 dr, racemic

(E) X-ray Crystal Structure of Product 3n and *N,N′*-Dioxide/Yb(OTf)$_3$ Complexes

The colourless and block-shape crystals were selected and mounted for the single-crystal X-ray diffraction. The data set was collected by a Bruker D8 Venture Photon II at 170 K equipped with micro-focus Cu radiation source (K_α = 1.54178Å). Applied with face-indexed numerical absorption correction, the structure solution was solved and refinement was processed by SHELXTL (version 6.14) program package. The structure was analyzed by ADDSYM routine implemented in PLATON suite and no higher symmetry was suggested.

The crystal of product **3n** was obtained in the solvents of dichloromethane and *n*-hexane. CCDC 1983265 contains the supplementary crystallographic data for this paper. These data can be obtained free of charge from The Cambridge Crystallographic Data Center via https://www.ccdc.cam.ac.uk/.

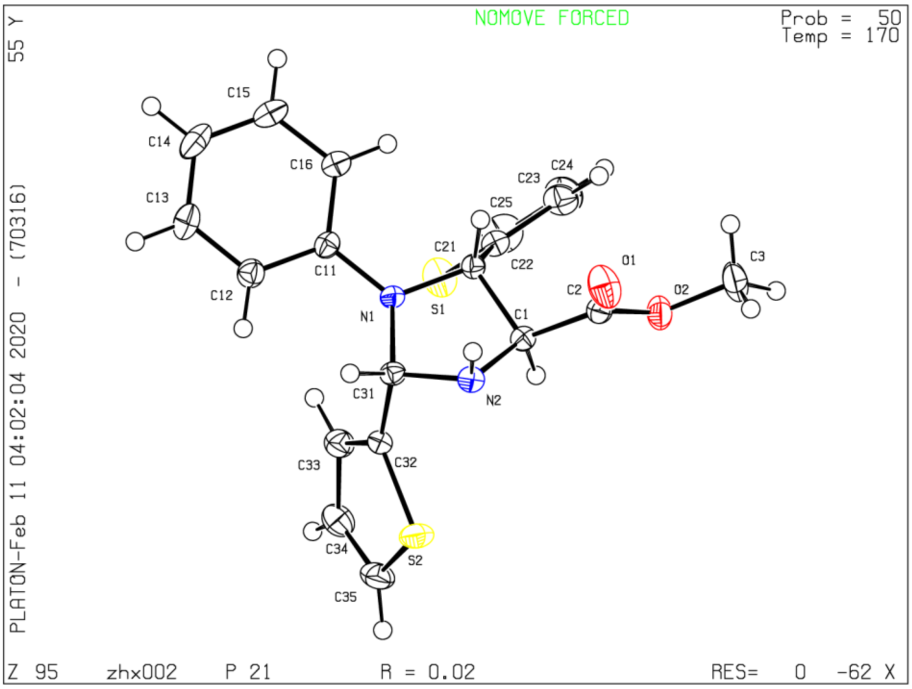

Figure S1. X-ray Crystal Structure of Product **3n**

Crystallographic Data for $C_{19}H_{18}N_2O_2S_2$.

Formula	$C_{19}H_{18}N_2O_2S_2$
Formula mass (amu)	370.47
Space group	P 21
a (Å)	9.6009 (2)
b (Å)	10.3983 (2)
c (Å)	9.9867 (2)
α (deg)	90
β (deg)	115.222 (2)
γ (deg)	90
V (Å^3)	901.95 (3)
Z	2
λ (Å)	1.54178
T (K)	170 K
r_{calcd} (g cm^{-3})	1.364
m (mm^{-1})	2.797
Transmission factors	0.686, 0.832
$2q_{max}$ (deg)	74.738
No. of unique data, including $F_o^2 < 0$	3635
No. of unique data, with $F_o^2 > 2s(F_o^2)$	3574
No. of variables	231

$R(F)$ for $F_o^2 > 2s(F_o^2)$ [a]	0.0236
$R_w(F_o^2)$ [b]	0.0607
Goodness of fit	1.038

[a] $R(F) = \sum||F_o| - |F_c|| / \sum|F_o|$. [b] $R_w(F_o^2) = [\sum[w(F_o^2 - F_c^2)^2] / \sum wF_o^4]^{1/2}$; $w^{-1} = [\sigma^2(F_o^2) + (Ap)^2 + Bp]$, where $p = [\max(F_o^2,0) + 2F_c^2] / 3$.

The crystal of **L-RaEt$_2$**/Yb(OTf)$_3$ was obtained in the solvents of MeOH and Et_2O. CCDC 2011003 contains the supplementary crystallographic data for this paper. These data can be obtained free of charge from The Cambridge Crystallographic Data Center via https://www.ccdc.cam.ac.uk/.

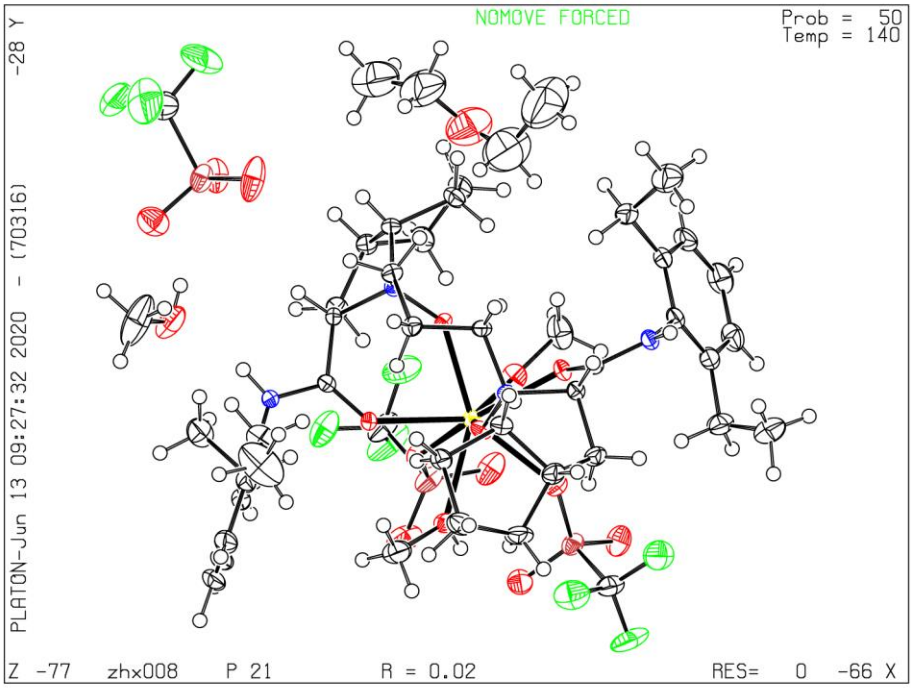

Square-antiprism geometry

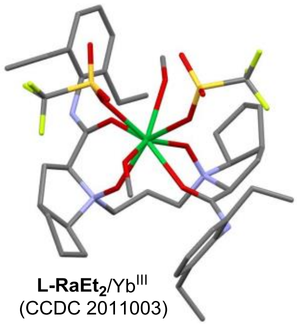

L-RaEt$_2$/Yb^{III}
(CCDC 2011003)

Figure S2. X-ray Crystal Structure of **L-RaEt$_2$**/Yb(OTf)$_3$ Complex

Crystallographic Data for $C_{43}H_{64}F_6N_4O_{12}S_2Yb$.

Formula	$C_{43}H_{64}F_6N_4O_{12}S_2Yb$
Formula mass (amu)	1435.37
Space group	P 21
a (Å)	11.9789 (5)
b (Å)	20.7474 (9)

c (Å)	12.9099 (5)
α (deg)	90
β (deg)	106.293 (1)
γ (deg)	90
V (Å^3)	3079.7 (2)
Z	2
λ (Å)	1.54178
T (K)	140 K
r_{calcd} (g cm^{-3})	1.548
m (mm^{-1})	4.592
Transmission factors	0.767,1.000
2q_{max} (deg)	80.757
No. of unique data, including $F_o^2 < 0$	13247
No. of unique data, with $F_o^2 > 2s(F_o^2)$	13218
No. of variables	778
$R(F)$ for $F_o^2 > 2s(F_o^2)$ [a]	0.0217
$R_w(F_o^2)$ [b]	0.0560
Goodness of fit	1.053

[a] $R(F) = \sum||F_o| - |F_c|| / \sum|F_o|$. [b] $R_w(F_o^2) = [\sum[w(F_o^2 - F_c^2)^2] / \sum wF_o^4]^{1/2}$; $w^{-1} = [\sigma^2(F_o^2) + (Ap)^2 + Bp]$, where $p = [\max(F_o^2,0) + 2F_c^2] / 3$.

The crystal of **L-TQ*t*Bu**/Yb(OTf)$_3$ was obtained in the solvents of THF/EtOAc and n-Hexane. CCDC 2011004 contains the supplementary crystallographic data for this paper. These data can be obtained free of charge from The Cambridge Crystallographic Data Center via https://www.ccdc.cam.ac.uk/.

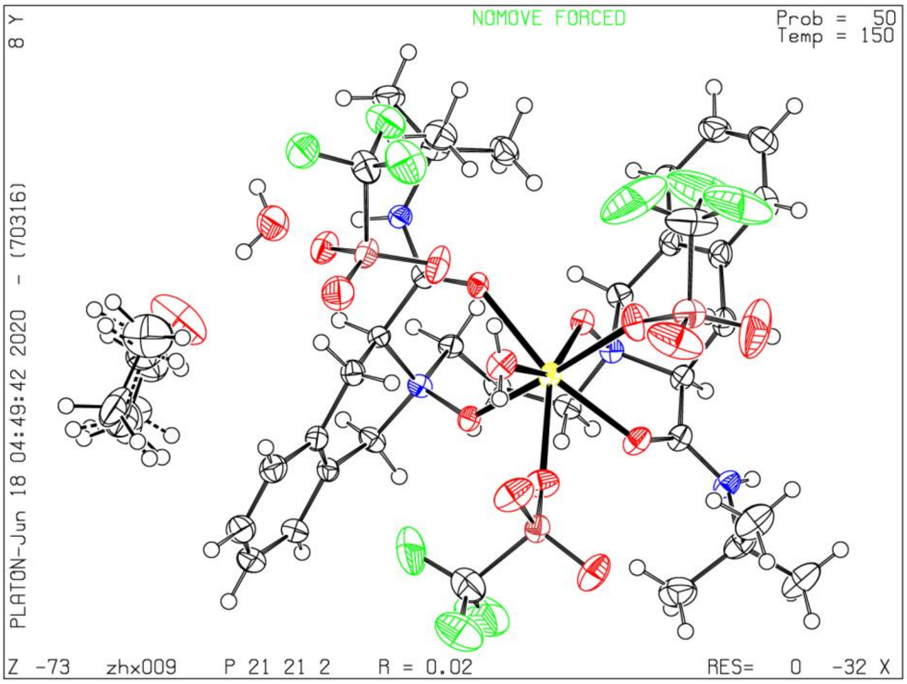

Pentagonal bipyramid geometry

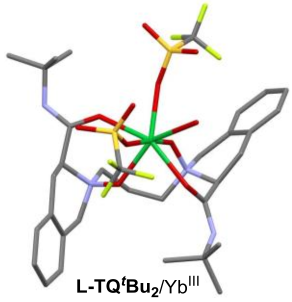

L-TQ^{t}Bu$_2$/Yb^{III}
(CCDC 2011004)

Figure S3. X-ray Crystal Structure of **L-TQ*t*Bu**/Yb(OTf)$_3$ Complex

Crystallographic Data for $C_{38}H_{56}F_9N_4O_{16}S_3Yb$.

Formula	$C_{38}H_{56}F_9N_4O_{16}S_3Yb$
Formula mass (amu)	1265.08
Space group	P 21 21 2
a (Å)	23.0690(11)
b (Å)	24.5839(12)
c (Å)	9.5501(4)
α (deg)	90
β (deg)	90
γ (deg)	90
V (Å^3)	5416.1(4)
Z	4
λ (Å)	0.71073
T (K)	150 K
r_{calcd} (g cm^{-3})	1.551
m (mm^{-1})	1.937
Transmission factors	0.353,0.616
2q_{max} (deg)	27.636
No. of unique data, including $F_o^2 < 0$	12456
No. of unique data, with $F_o^2 > 2s(F_o^2)$	12275
No. of variables	704
$R(F)$ for $F_o^2 > 2s(F_o^2)$ [a]	0.0215
$R_w(F_o^2)$ [b]	0.0572
Goodness of fit	1.125

[a]$R(F) = \sum||F_o| - |F_c|| / \sum|F_o|$. [b]$R_w(F_o^2) = [\sum[w(F_o^2 - F_c^2)^2] / \sum wF_o^4]^{1/2}$; $w^{-1} = [\sigma^2(F_o^2) + (Ap)^2 + Bp]$, where $p = [\max(F_o^2,0) + 2F_c^2] / 3$.

(F) Experimental Procedure for the Scale-up Reaction and Transformations of the Products

1 Scale-up experiment

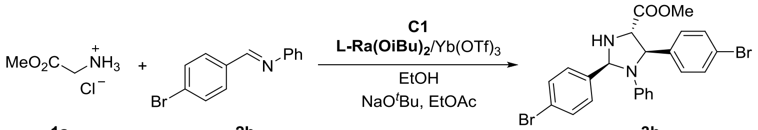

1a **2h** **3h**

The reaction was conducted with methyl glycine hydrochloride **1a** (378.0 mg, 3.0 mmol), $Yb(OTf)_3$ (186.0 mg, 10 mol%), **L-Ra(OiBu)$_2$** (246.0 mg, 10 mol%), NaO*t*Bu (288.0 mg, 3.0 mmol) and EtOH (150.0 μL, 8.0 equiv). Picolinaldehyde (**C1**, 60.0 μL 20 mol%) and EtOAc (18.0 mL) were added in succession. The mixture was stirred at 35 °C for 30 min, and imine **2h** (2340.0 mg, 9.0 mmol) was added at 35 °C, the resulting mixture was stirred at 35 °C for 60 hours. The reaction mixture was subjected to column chromatography on silica gel to afford the product **3h** (PET/EtOAc = 10/1 to PET/EtOAc = 5/1 as eluent) as pale yellow oil (0.9725 g, 62% yield, 92:8 dr, 93% ee). The dr value was as determined by ^{1}H NMR analysis and the ee value was determined by high-performance liquid chromatography (HPLC) with chiralcel Daicel Chiralcel IF column.

2 Experimental procedures for further transformations of product 3a.

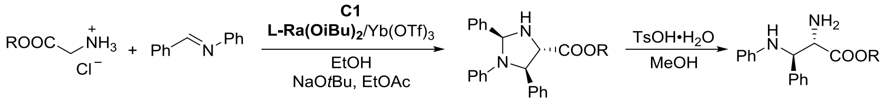

1 **2a** **3** **6**

The reaction was conducted with $Yb(OTf)_3$ (6.2 mg, 10 mol%), **L-Ra(OiBu)$_2$** (8.2 mg, 10 mol%), NaO*t*Bu (9.6 mg, 0.10 mmol), methyl glycine hydrochloride **1a** (12.6 mg, 0.10 mmol), and EtOH (5.0 μL, 8.0 equiv). Picolinaldehyde (**C1**, 2.0 μL 20 mol%) and EtOAc (0.60 mL) were added in succession. The mixture was stirred at 35 °C for 15 min, and imine **2a** (54.0 mg, 0.30 mmol) was added at 35 °C. The resulting mixture was stirred at 35 °C for 48 hours. The reaction mixture was subjected to column chromatography on silica gel to afford the crude product **3a**. The product **3a** was dissolved in 3.0 mL of methanol at room temperature followed by the addition of $TsOH{\cdot}H_2O$ (38.0 mg, 0.20 mmol). The reaction mixture was stirred for 8 h and neutralized the mixture by saturated Na_2CO_3 solution. Then the mixture was partitioned between ethyl acetate and water, then the organic layer was washed with brine, dried over $MgSO_4$, filtered and evaporated in vacuo than the organic solvent was removed and then the residue was purified by column chromatography on silica gel and eluted with petroleum ether/ethyl acetate (4/1 to 1/1, v/v) to afford the corresponding product **6a**.

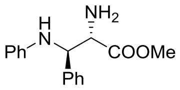

6a

Methyl (2*S*,3*R*)-2-amino-3-phenyl-3-(phenylamino)propanoate (6a): 51% yield over two step, White solid; **m.p.**: 93.6 – 97.0; R_f = 0.4 (petroleum ether/ethyl acetate = 1/1); Dissolved in iPrOH for HPLC; **HPLC** (Chiralcel IG, hexane/iPrOH = 90/10, flow rate 1.0 mL/min, λ = 254 nm); major isomer: t_r (major) = 13.38 min, t_r (minor) = 14.45 min, ee = 98%; minor isomer: t_r (major) = 15.25 min. dr = 93:7 determined by ^{1}H NMR. $[\alpha]^{24}_D$ = +138.2 (*c* = 0.15, in tetrahydrofuran). **^{1}H NMR (400 MHz, CDCl$_3$)** δ 7.38 – 7.24 (m, 6H), 7.10 – 7.03 (m, 2H), 6.66 – 6.61 (m, 1H), 6.55 – 6.51 (m, 2H), 5.11 (s, 1H), 4.88 (s, 1H), 3.94 (d, *J* = 3.6 Hz, 1H), 3.70 (s, 3H) ppm; **^{13}C{^{1}H} NMR (101 MHz, CDCl$_3$)** δ 173.2, 146.8, 140.1, 129.1, 128.7, 127.5, 126.9, 117.4, 113.8, 59.6, 59.1, 52.4 ppm; **IR (neat)** **ν (cm^{-1})**: 3389, 3027, 1737, 1603, 1506, 1453, 1434, 1229, 993, 752, 704; **HRMS** (ESI-FT) calcd for $C_{16}H_{19}N_2O_2^+$ ($[M]+H^+$) = 271.1441, found 271.1436.

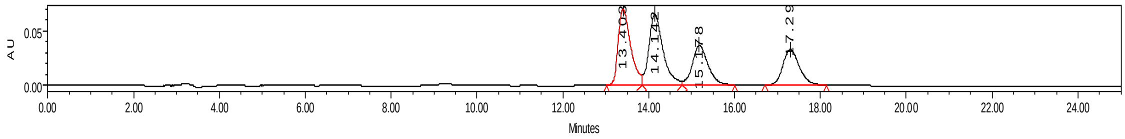

	Retention Time	Area	% Area
1	13.403	1421789	29.99
2	14.142	1505233	31.75
3	15.178	929616	19.61
4	17.299	884658	18.66

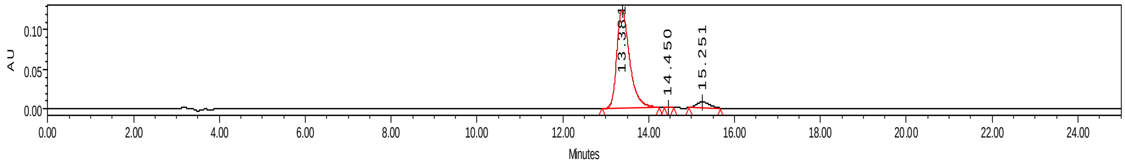

	Retention Time	Area	% Area
1	13.381	2571988	94.21
2	14.450	878	0.03
3	15.251	157306	5.76

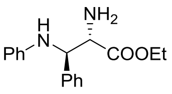

6b

Ethyl (2*S*,3*R*)-2-amino-3-phenyl-3-(phenylamino)propanoate (6b): Procedure C: 30% yield over two step, White solid; **m.p.**: 74.0 – 78.7; R_f = 0.4 (petroleum ether/ethyl acetate = 1/1); Dissolved in iPrOH for HPLC; **HPLC** (Chiralcel IH, hexane/iPrOH = 90/10, flow rate 1.0 mL/min, λ = 254 nm); major isomer: t_r (major) = 11.76 min, t_r (minor) = 16.39 min, ee = 96%; minor isomer: t_r (major) = 14.42 min. dr = 91:9 determined by ^{1}H NMR. $[\alpha]^{27}_{D}$ = +59.7 (*c* = 0.30, in tetrahydrofuran). **^{1}H NMR (400 MHz, CDCl$_3$)** δ 7.31 – 7.14 (m, 6H), 7.01 – 6.96 (m, 2H), 6.57 – 6.52 (m, 1H), 6.48 – 6.44 (m, 2H), 5.01 (s, 1H), 4.79 – 4.77 (m, 1H), 4.07 (q, *J* = 7.2 Hz, 2H), 3.83 (d, *J* = 4.0 Hz, 1H), 1.09 (t, *J* = 7.1 Hz, 3H) ppm; **^{13}C{^{1}H} NMR (101 MHz, CDCl$_3$)** δ 172.8, 147.0, 140.2, 129.0, 128.7, 127.4, 126.9, 117.4, 113.7, 61.4, 59.6, 59.3, 14.1 ppm; **IR (neat) ν (cm^{-1})**: 3360, 2932, 1731, 1599, 1502, 1453, 1345, 1259, 1195, 1061, 751, 697; **HRMS** (ESI-FT) calcd for $C_{17}H_{21}N_2O_2^+$ ([M]+H$^+$) = 285.1598, found 285.1598.

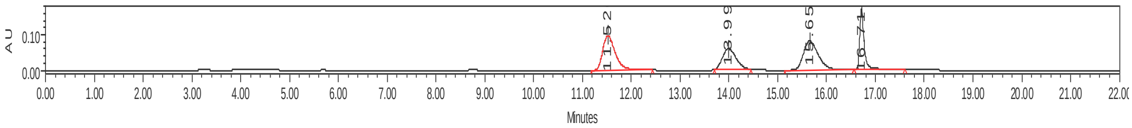

	Retention Time	Area	% Area
1	11.520	1749632	30.38
2	13.990	1126999	19.57
3	15.654	1758762	30.54
4	16.715	1124420	19.52

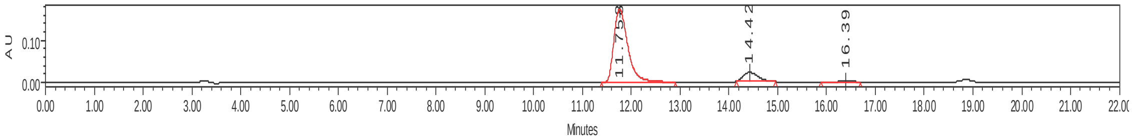

	Retention Time	Area	% Area
1	11.758	3461663	87.56
2	14.423	456104	11.54
3	16.393	35843	0.91

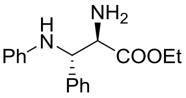

(2*R*,3*S*)-**6b**

Ethyl (2*R*,3*S*)-2-amino-3-phenyl-3-(phenylamino)propanoate [(2*R*,3*S*)-6b]: Procedure C: 29% yield over two step, R_f = 0.4 (petroleum ether/ethyl acetate = 1/1); Dissolved in iPrOH for HPLC; **HPLC** (Chiralcel IH, hexane/iPrOH = 90/10, flow rate 1.0 mL/min, λ = 254 nm); major isomer: t_r (major) = 15.62 min, t_r (minor) = 11.62 min, ee = 91%; minor isomer: t_r (major) = 16.94 min, t_r (minor) = 14.12 min, ee = 91%. dr = 82:18 determined by ^{1}H NMR. $[\alpha]^{29}_{D}$ = –58.1 (*c* = 0.24, in tetrahydrofuran). **^{1}H NMR (400 MHz, CDCl$_3$)** δ 7.46 – 7.15 (m, 6H), 7.08 – 7.03 (m, 2H), 6.64 – 6.59 (m, 1H), 6.54 – 6.51 (m, 2H), 5.08 (s, 1H), 4.87 – 4.84 (m, 1H), 4.13 (q, *J* = 6.8 Hz, 2H), 3.90 (d, *J* = 3.6 Hz, 1H), 1.16 (t, *J* = 7.2 Hz, 3H) ppm; **^{13}C{^{1}H} NMR (101 MHz, CDCl$_3$)** δ 172.8, 146.9, 140.2, 129.0, 128.7, 127.4, 126.9, 117.4, 113.7, 61.4, 59.6, 59.3, 14.1 ppm.

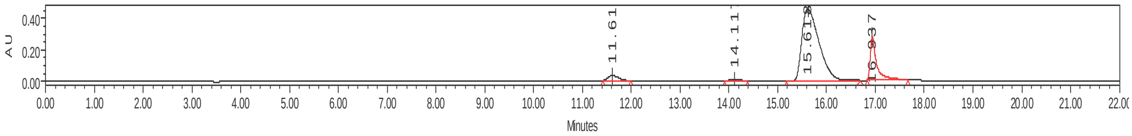

	Retention Time	Area	% Area
1	11.615	543218	3.72
2	14.117	141431	0.97
3	15.618	11418949	78.19
4	16.937	2499682	17.12

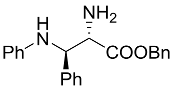

6c

Benzyl (2*S*,3*R*)-2-amino-3-phenyl-3-(phenylamino)propanoate (6c): Procedure C: 60% yield over two step, White solid; **m.p.**: 108.3 – 114.7; R_f = 0.4 (petroleum ether/ethyl acetate = 1/1); Dissolved in iPrOH for HPLC; **HPLC** (Chiralcel IH, hexane/iPrOH = 90/10, flow rate 1.0 mL/min, λ = 254 nm); major isomer: t_r (major) = 12.35

min, t_r (minor) = 14.01 min, ee = 91%; minor isomer: t_r (major) = 15.05 min, t_r (minor) = 18.51 min, ee = 93%. dr = 78:22 determined by ^{1}H NMR. $[\alpha]^{27}_D$ = +11.1 (*c* = 0.32, in tetrahydrofuran). **^{1}H NMR (400 MHz, $CDCl_3$)** δ 7.32 – 7.12 (m, 12H), 7.03 – 6.98 (m, 2H), 6.60 – 6.50 m, 1H), 6.48 – 6.42 (m, 2H), 5.15 – 4.98 (m, 3H), 4.86 – 4.82 (m, 1H), 3.92 (d, *J* = 3.6 Hz, 1H) ppm; **^{13}C{^{1}H} NMR (101 MHz, $CDCl_3$)** δ 172.6, 146.8, 140.0, 135.3, 129.1, 128.7, 128.5, 128.3, 128.1, 127.5, 126.9, 117.5, 113.8, 67.1, 59.6, 59.1. ppm; **IR (neat) ν (cm^{-1})**: 3380, 2924, 2853, 1736, 1602, 1504, 1454, 1180, 1028, 749, 696; **HRMS** (ESI-FT) calcd for $C_{22}H_{23}N_2O_2^+$ ([M]+H$^+$) = 347.1754, found 347.1758.

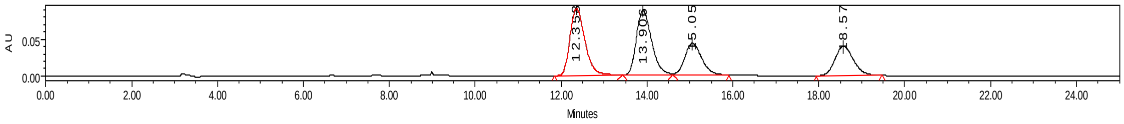

	Retention Time	Area	% Area
1	12.358	2226367	32.61
2	13.906	2210088	32.37
3	15.057	1194210	17.49
4	18.573	1196723	17.53

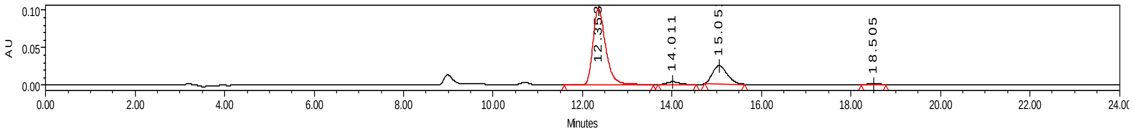

	Retention Time	Area	% Area
1	12.353	2017618	75.33
2	14.011	82932	3.10
3	15.051	553537	20.67
4	18.505	24266	0.91

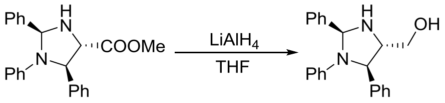

3a
93:7 dr, 97% ee

7a

To a solution of **3a** (35.7 mg, 0.10 mmol, dr value was determined by ^{1}H NMR) in THF (1.0 mL) was added $LiAlH_4$ (11 mg, 0.30 mmol) at 0 ºC. After stirring for 60 minutes at 0 ºC, the reaction mixture was allowed to warm to room temperature for an additional 2 h. Then, the reaction mixture was diluted with Et_2O and cooled to 0 ºC. H_2O (10.0 μL), 15% NaOH (aq. 10.0 μL) and H_2O (30.0 μL) were added, the reaction mixture was allowed to warm to room temperature to stir for 15 minutes. The mixture was dried over Mg_2SO_4, filtered and evaporated in vacuo, and was subjected to column chromatography on silica gel and eluted with petroleum ether/ethyl acetate (5/1 to 2/1, v/v).

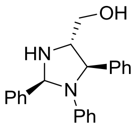

7a

[(2*S*,4*S*,5*R*)-1,2,5-Triphenylimidazolidin-4-yl]methanol (7a): 64% yield, Colorless oil; R_f = 0.3 (petroleum ether/ethyl acetate = 2/1); Dissolved in iPrOH for HPLC; **HPLC** (Chiralcel ASH, hexane/iPrOH = 90/10, flow rate 1.0 mL/min, λ = 254 nm) major isomer: t_r (major) = 12.94 min, t_r (minor) = 18.44 min, ee = 95%. dr = 96:4 determined by ^{1}H NMR. $[\alpha]^{24}_D$ = –41.2 (*c* = 0.25, in tetrahydrofuran). **^{1}H NMR (400 MHz, $CDCl_3$)** δ 7.70 – 7.66 (m, 2H), 7.55 – 7.50 (m, 2H), 7.45 – 7.23 (m, 7H), 7.11 – 7.03 (m, 2H), 6.74 – 6.69 (m, 1H), 6.51 – 6.47 (m, 2H), 5.52 (s, 1H), 4.50 (d, *J* = 4.4 Hz, 1H), 3.75 (dd, *J* = 11.1, 4.8 Hz, 1H), 3.66 (dd, *J* = 11.2, 7.6 Hz, 1H), 3.44 – 3.38 (m, 1H), 2.39 (s, 1H) ppm; **^{13}C{^{1}H} NMR (101 MHz, $CDCl_3$)** δ 147.2, 142.8, 141.0, 128.9, 128.8, 128.8, 128.3, 127.4, 126.9, 126.1, 118.2, 114.6, 78.9, 68.1, 67.3, 61.2 ppm; **IR (neat) ν (cm^{-1})**: 3310, 2925, 1599, 1501, 1453, 1332, 1029, 996, 751, 695; **HRMS** (ESI-FT) calcd for $C_{22}H_{23}N_2O^+$ ([M]+H$^+$) = 331.1805, found 331.1805.

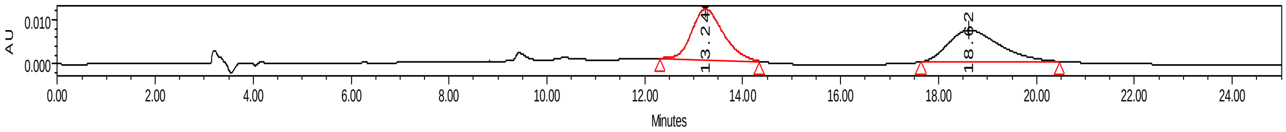

	Retention Time	Area	% Area
1	13.240	511482	49.63

2	18.622	519100	50.37

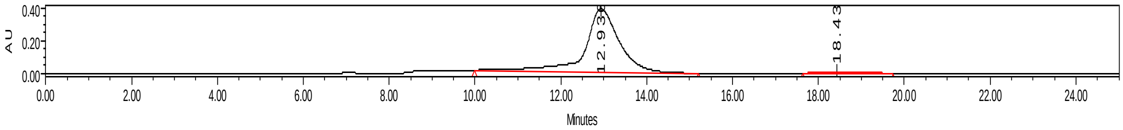

	Retention Time	Area	% Area
1	12.936	21737505	97.58
2	18.435	540004	2.42

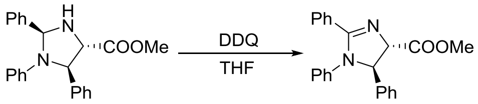

3a
93:7 dr, 97% ee

8a

To a solution of **3a** (17.9 mg, 0.05 mmol, dr value was determined by ^{1}H NMR) in THF (1.5 mL) was added DDQ (11.4 mg, 0.05 mmol) at room temperature. The reaction mixture was stirred at room temperature for 10 min. The mixture was partitioned between ethyl acetate and water, then the organic layer was washed with brine, dried over $MgSO_4$, filtered and evaporated in vacuo, and was subjected to column chromatography on silica gel and eluted with petroleum ether/ethyl acetate (4/1 to 1/1, v/v).

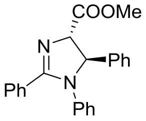

8a

Methyl (4*S*,5*R*)-1,2,5-triphenyl-4,5-dihydro-1H-imidazole-4-carboxylate (8a): 68% yield, Colorless oil; R_f = 0.4 (petroleum ether/ethyl acetate = 1/1); Dissolved in iPrOH for HPLC; **HPLC** (Chiralcel IF, hexane/iPrOH = 90/10, flow rate 1.0 mL/min, λ = 254 nm) major isomer: t_r (major) = 20.36 min, t_r (minor) = 15.18 min, ee = 95%. dr > 96:4 determined by ^{1}H NMR. $[\alpha]^{24}_D$ = –600.0 (*c* = 0.18, in tetrahydrofuran). **^{1}H NMR (400 MHz, CDCl$_3$)** δ 7.66 – 7.61 (m, 2H), 7.46 – 7.37 (m, 5H), 7.33 – 7.26 (m, 3H), 7.10 – 6.96 (m, 3H), 6.80 – 6.72 (m, 2H), 5.29 (d, *J* = 6.8 Hz, 1H), 4.72 (d, *J* = 6.8 Hz, 1H), 3.83 (s, 3H) ppm; **^{13}C{^{1}H} NMR (101 MHz, CDCl$_3$)** δ 172.1, 164.6, 142.4, 142.1, 130.4, 130.3, 129.3, 129.0, 128.8, 128.1, 128.1, 126.7, 125.1, 124.5, 76.6, 71.8, 52.6 ppm; **IR (neat) ν (cm^{-1})**: 2951, 1740, 1594, 1570, 1495, 1451, 1384, 1227, 1033, 765, 698; **HRMS** (ESI-FT) calcd for $C_{23}H_{21}N_2O_2^+$ ([M]+H$^+$) = 357.1598, found 357.1594.

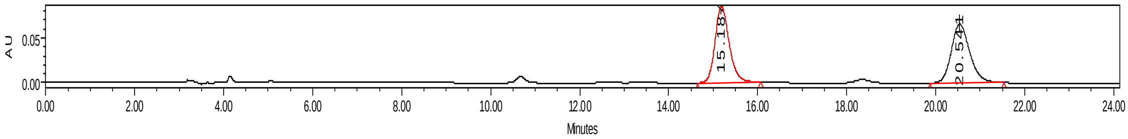

	Retention Time	Area	% Area
1	15.184	1904639	50.01
2	20.541	1904067	49.99

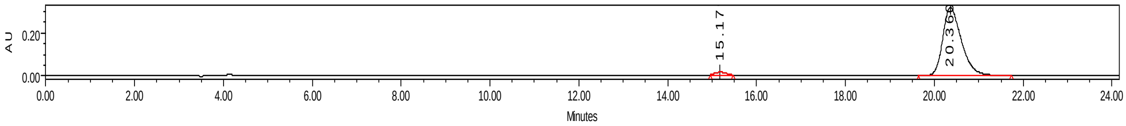

	Retention Time	Area	% Area
1	15.179	230205	2.40
2	20.360	9355023	97.60

(G) Procedure for React IR Experiment for Kinetic Studies

Kinetic analyses were performed using in situ attenuated total reflectance Fouriertransform infrared (ATR FTIR) spectroscopy to track the formation of product **3g** under synthetically relevant conditions. A Mettler Toledo ReactIR 15 was treated as main experiment equipment. All of the kinetic experiments on each plot were performed using a single batch of reagents.

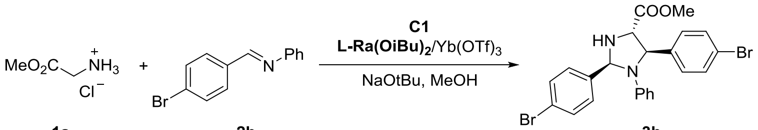

1a **2h** **3h**

A three-necked reaction tube was charge with methyl glycine hydrochloride **1a** (0.5 mmol), $Yb(OTf)_3$ (10 mol%), **L-Ra(OiBu)$_2$** (10 mol%), NaO*t*Bu (0.5 mmol). Picolinaldehyde (**C1**, 20 mol%) and MeOH (3.5 mL) were added in succession. The mixture was stirred at 35 °C for 30 min, and imine **2h** (1.25 mmol) was added at 35 °C and the IR data collection was started. The React IR spectra were recorded over the process of the reaction. ConcIRT Spectrum of the **3h** with peak at 1502 cm-1 was selected to acquire kinetic data.

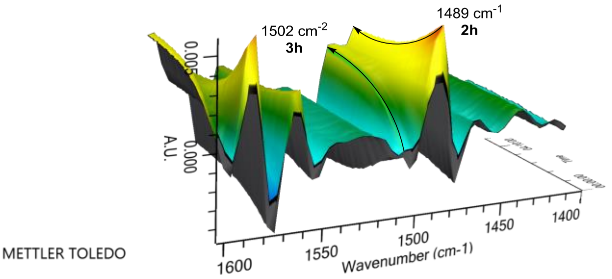

Figure S4. 3D ATR-FTIR Profile of the Recorded over the Reaction of **2h** and **3h**

1. Dependence of the reaction rate on concentration of L-Ra(O^{i}Bu)$_2$/Yb(OTf)$_3$ catalyst

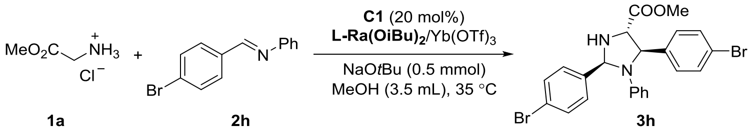

L-Ra(OiBu)$_2$/$Yb(OTf)_3$
1:1, 0.025 mmol
1:1, 0.0375 mmol
1:1, 0.05 mmol
1:1, 0.0625 mmol
1:1, 0.075 mmol

1a 0.5 mmol; **2h** 1.25 mmol; **3h**

Kinetic profiles of different initial concentration of **L-Ra(OiBu)$_2$**/$Yb(OTf)_3$ (from 0.007142857 M to 0.021428571 M), The plot of k_{obs} vs [**L-Ra(OiBu)$_2$**/$Yb(OTf)_3$] displayed a liner relationship in [**L-Ra(OiBu)$_2$**/$Yb(OTf)_3$], which indicates a first-order kinetic dependence in [**L-Ra(OiBu)$_2$**/$Yb(OTf)_3$].

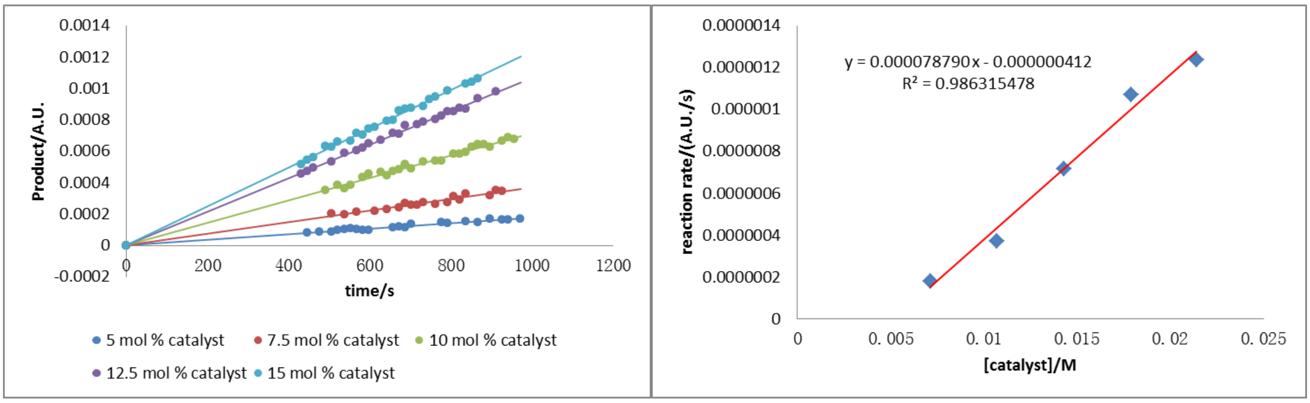

[**L-Ra(OiBu)$_2$**/$Yb(OTf)_3$]/M	reaction rate/(A.U./s)
0.007142857	0.000000178
0.010714286	0.000000369
0.014285714	0.000000717
0.017857142	0.000001068
0.021428571	0.000001235

2. Dependence of the reaction rate on concentration of picolinaldehyde (C1)

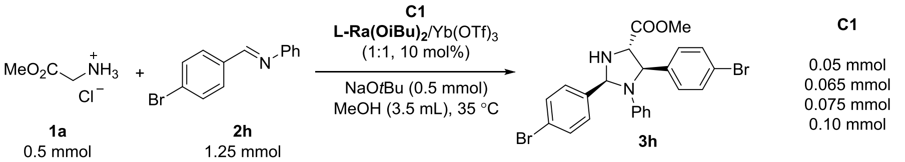

Kinetic profiles of different initial concentration of **C1** (from 0.014285714 M to 0.028571429 M), The plot of k_{obs} vs [**C1**] displayed a liner relationship in [**C1**], which indicates a first-order kinetic dependence in [**C1**].

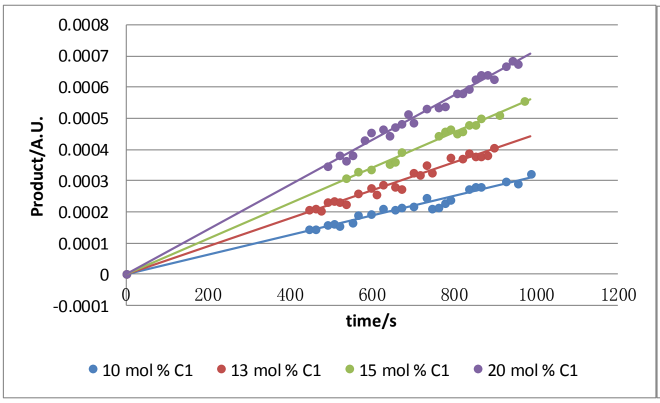

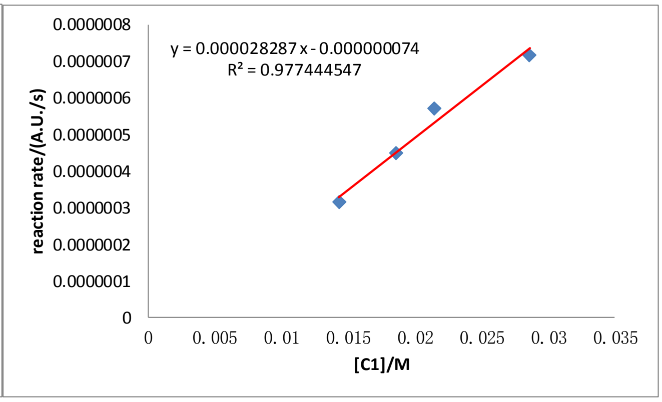

[**C1**]/M	reaction rate/(A.U./s)
0.014285714	0.000000314
0.018571429	0.000000448
0.021428571	0.000000570
0.028571429	0.000000717

3. Dependence of the reaction rate on concentration of 1a

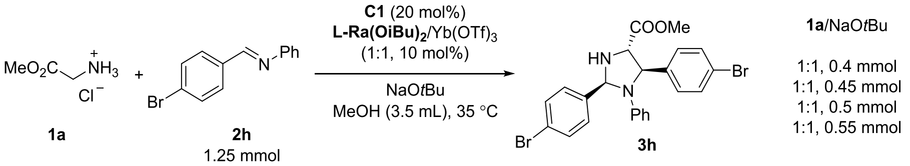

Kinetic profiles of different initial concentration of **1a** (from 0.114285714 M to 0.157142857 M), The plot of k_{obs} vs [**1a**] displayed a liner relationship in [**1a**], which indicates a first-order kinetic dependence in [**1a**].

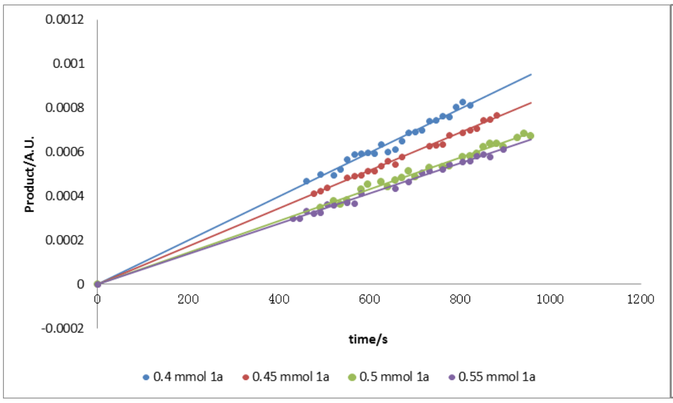

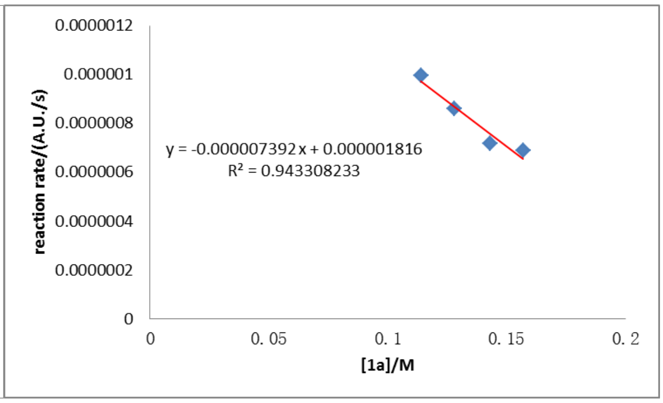

[**1a**]/M	reaction rate/(A.U./s)
0.114285714	0.000000995
0.128571429	0.000000859

0.142857143	0.000000717
0.157142857	0.000000688

4. Dependence of the reaction rate on concentration of 2h

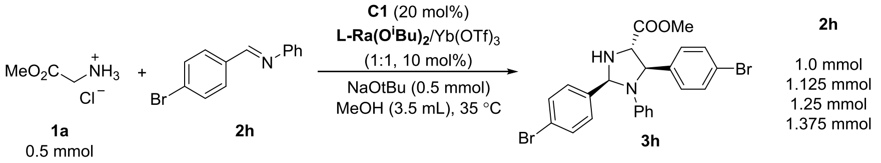

Kinetic profiles of different initial concentration of **2h** (from 0.285714286 M to 0.392857143 M), The plot of k_{obs} vs [**2h**] displayed a liner relationship in [**2h**], which indicates a first-order kinetic dependence in [**2h**].

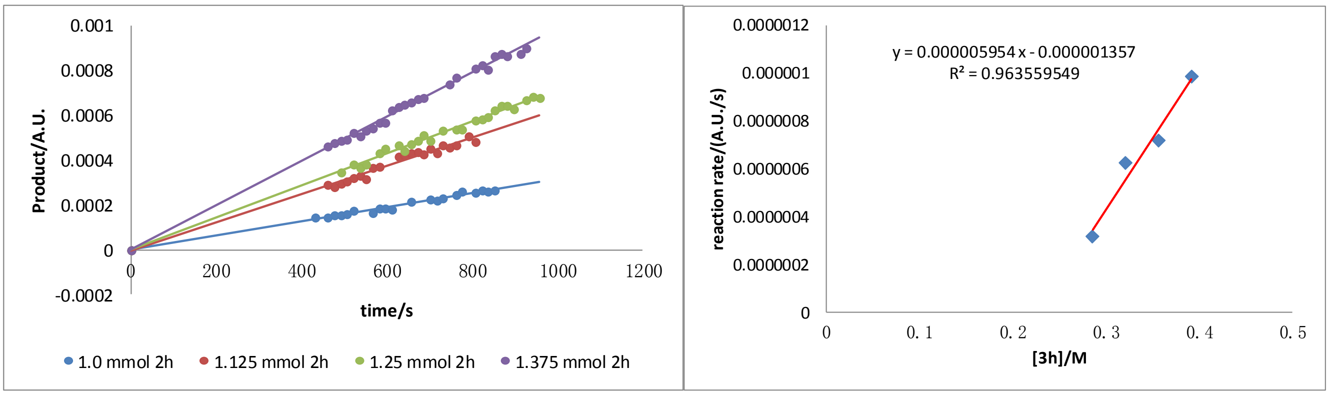

[**2h**]/M	reaction rate/(A.U./s)
0.285714286	0.000000316
0.321428571	0.000000623
0.357142857	0.000000717
0.392857143	0.000000988

(H) Control Experiments and HRMS Analysis

1. Control experiments

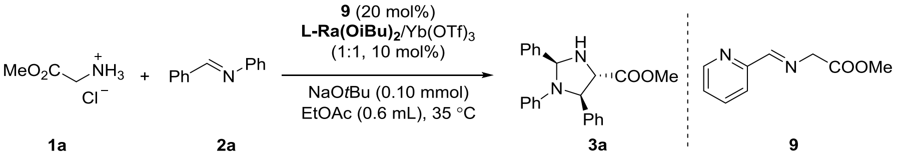

The reaction was conducted with **1a** (12.6 mg, 0.10 mmol), Yb(OTf)$_3$ (6.2 mg, 10 mol%), **L-Ra(OiBu)$_2$** (8.2 mg, 10 mol%), **9** (20 mol%), NaO^{t}Bu (9.6 mg, 0.10 mmol) and EtOAc (0.60 mL). The mixture was stirred at 35 °C for 15 min, and imine **2a** (36.0 mg, 0.20 mmol) was added at 35 °C. The resulting mixture was stirred at 35 °C for 24 hours. The reaction mixture was subjected to column chromatography on silica gel to afford the corresponding product **3a**.
The results of the reaction suggested that Schiff-base **9** was probably a key intermediate in this reaction.

entry	condition	yield (%)^b	dr^c	ee (%)^c
1	none	31	93:7	96
2	at 50 °C	41	80:20	79/80
3	with 5 μL (26.0 equiv) H_2O	39	84:16	96/91

4	with 5 μL (8.0 equiv) EtOH	42	92:8	96

[a] Unless otherwise noted, all reactions were carried out with **1a** (0.10 mmol), **2a** (0.20 mmol), NaO*t*Bu (0.10 mmol), **9**, and **L-Ra(OiBu)$_2$**/Yb(OTf)$_3$ (1:1, 10 mol%) in EtOAc (0.60 mL) at 35 °C for 24 h. [b] Isolated yield of **3a** based on **1a**. [c] Determined by HPLC on a chiral stationary phase.

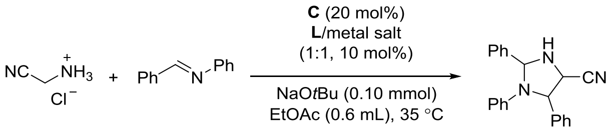

1d **2a** **3da**

The change from ester group to nitrile group performed poor results, highlighting the significant role of the carboxylate in mediating the reactivity.

entry	**Carbonyl catalyst**	**L**/metal salt	yield (%)[b]	dr[c]
1	**C1**	**L-Pi(O^{i}Bu)$_2$**/Yb(OTf)$_3$	<3	N.D.
2	**C1**	**L-Ra(O^{i}Bu)$_2$**/Yb(OTf)$_3$	<3	N.D.
3[d]	**C9**	***rac*-PiPr$_2$**/Yb(OTf)$_3$	10	85:15

[a] Unless otherwise noted, all reactions were carried out with **1d** (0.10 mmol), **2a** (0.20 mmol), NaO*t*Bu (0.10 mmol), **C** (10 mol%) and **L**/metal salt (1:1, 10 mol%) in EtOAc (0.60 mL) at 35 °C for 48 h; N.D. = no determined. [b] Isolated yield of **3da** based on **1d**. [c] Determined by ^{1}H NMR. [d] Et_3N (0.11 mmol) was used in CH_2Cl_2 (0.60 mL) for 24 h..

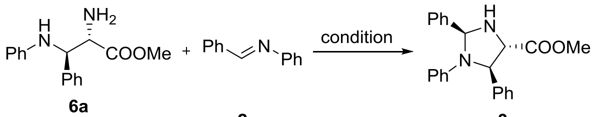

6a
95:5 dr (by HPLC)
96% ee

2a **3a**

The reaction was conducted with **6a** (27.0 mg, 0.10 mmol), imine **2a** (18.0 mg, 0.10 mmol), dissolved in EtOAc (0.60 mL). The resulting mixture was stirred at 35 °C for 24 hours. The reaction mixture was subjected to column chromatography on silica gel to afford the corresponding product **3a**.

entry	conditions	yield (%)[a]	dr[b]	ee (%)[c]
1	none	46	94:6	96
2	with 10 mol% Yb(OTf)$_3$	64	94:6	96
3	with 10 mol% **L-Ra(OiBu)$_2$**/Yb(OTf)	51	96:4	97

[a] Isolated yield of **3a** based on **1a**. [b] Determined by HPLC on a chiral stationary phase.

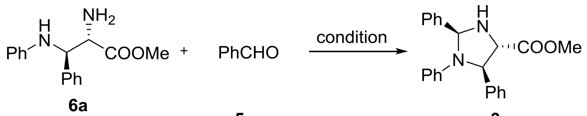

6a
95:5 dr (by HPLC)
96% ee

5a **3a**

The reaction was conducted with **6a** (27.0 mg, 0.10 mmol), benzaldehyde **5a** (10.0 μL, 0.10 mmol), dissolved in EtOAc (0.60 mL). The resulting mixture was stirred at 35 °C for 24 hours. The reaction mixture was subjected to column chromatography on silica gel to afford the corresponding product **3a**.
These results clearly indicated that imidazolidine **3a** was yielded through the condensation of initial Mannich product **6a** with benzaldehyde generated from the decomposition of imine **2a** in the catalytic reaction.

entry	condition	yield (%)[a]	dr[b]	ee (%)[b]
1	none	26	95:5	96

2	with 10 mol% $Yb(OTf)_3$	37	95:5	96
3	with 10 mol% **L-Ra(OiBu)$_2$/**Yb(OTf)	90	95:5	97

[a] Isolated yield of **3a** based on **1a**. [b] Determined by HPLC on a chiral stationary phase.

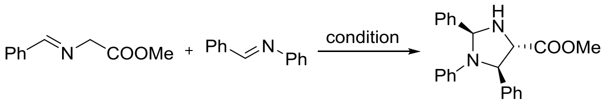

10 **2a** **3a**

The reaction was conducted with **10** (17.7 mg, 0.10 mmol), $Yb(OTf)_3$ (6.2 mg, 10 mol%), **L-Ra(OiBu)$_2$** (8.2 mg, 10 mol%) and EtOAc (0.60 mL). The mixture was stirred at 35 °C for 15 min, and imine **2a** (18.0 mg, 0.10 mmol) was added at 35 °C. The resulting mixture was stirred at 35 °C for 24 hours. The reaction mixture was subjected to column chromatography on silica gel to afford the corresponding product **3a**.
The results indicated that the [3+2] cycloaddition pathway was disfavored in this reaction condition.

entry	condition	yield (%)[a]	dr[b]	ee (%)[b]
1	none	N.R.		
2	with 1.0 equiv. NaO*t*Bu	N.R.		

[a] Isolated yield of **3a** based on **1a**. [b] Determined by HPLC on a chiral stationary phase.

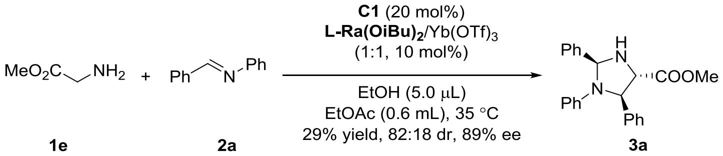

1e **2a** **3a**

The reaction was conducted with **1e** (8.9 mg, 0.10 mmol), $Yb(OTf)_3$ (6.2 mg, 10 mol%), **L-Ra(OiBu)$_2$** (8.2 mg, 10 mol%), **C1** (20 mol%), EtOH (5.0 μL, 8.0 equiv) and EtOAc (0.60 mL). The mixture was stirred at 35 °C for 15 min, and imine **2a** (36.0 mg, 0.20 mmol) was added at 35 °C. The resulting mixture was stirred at 35 °C for 24 hours. The reaction mixture was subjected to column chromatography on silica gel to afford the corresponding product **3a**.

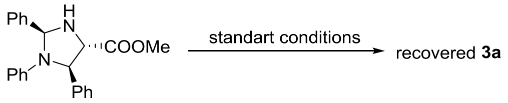

3a

The reaction was conducted with **3a** (35.6 mg, 0.10 mmol), $Yb(OTf)_3$ (6.2 mg, 10 mol%), **L-Ra(OiBu)$_2$** (8.2 mg, 10 mol%), EtOH (5.0 μL, 8.0 equiv) and EtOAc (0.60 mL). The mixture was stirred at 35 °C for 24 hours. The reaction mixture was subjected to column chromatography on silica gel to afford the recovered product **3a**.
The results indicated that the imidazolidine was instable under reaction conditions.

entry	condition	recovery (%)[a]	dr[b]	ee (%)[b]
1	none	80	94:6	97
2	with $Yb(OTf)_3$ only	67	96:4	96
3	with 1.0 equiv. NaO^{t}Bu	85	96:4	97

[a] Isolated recovery of **3a** based on **1a**. [b] Determined by HPLC on a chiral stationary phase.

2. HRMS analysis of the reaction mixture

2.1 HRMS analysis of the reaction mixture

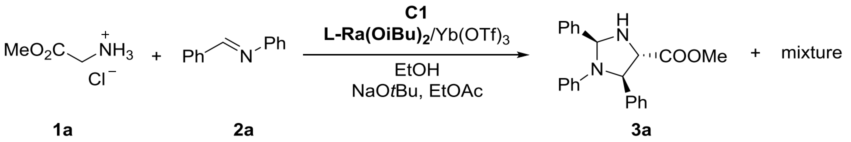

The reaction was conducted with $Yb(OTf)_3$ (6.2 mg, 10 mol%), **L-Ra(OiBu)$_2$** (8.2 mg, 10 mol%), NaO^tBu (9.6 mg, 0.10 mmol), methyl glycine hydrochloride **1a** (12.6 mg, 0.10 mmol), and EtOH (5.0 μL). Picolinaldehyde (**C1**, 20 mol%) and EtOAc (0.60 mL) were added in succession. The mixture was stirred at 35 °C for 15 min, and imine **2a** (54.0 mg, 0.30 mmol) was added at 35 °C. The resulting mixture was stirred at 35 °C for 24 hours. Finally, the solution was purified by flash filtration with a thin silica gel and the mixture was directly analyzed by HRMS.

Results:

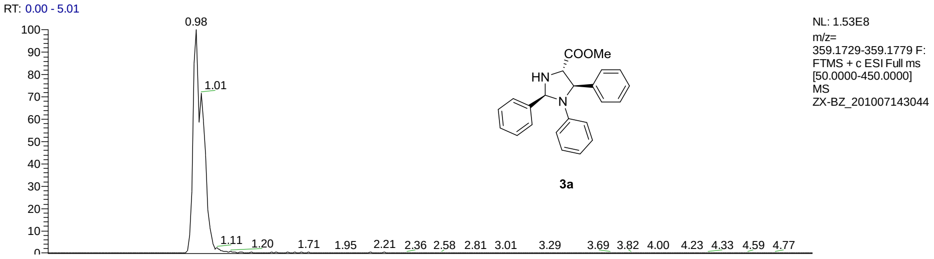

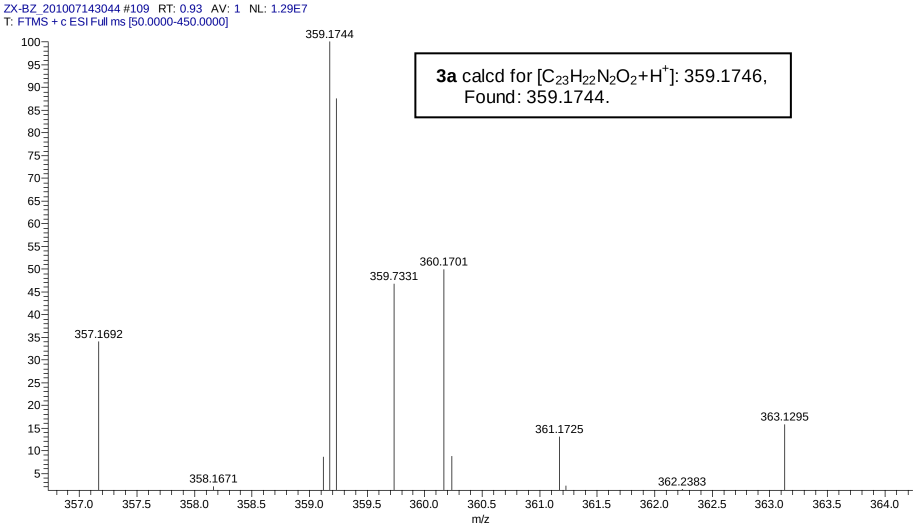

3a calcd for $[C_{23}H_{22}N_2O_2+H^+]$: 359.1746, Found: 359.1744.

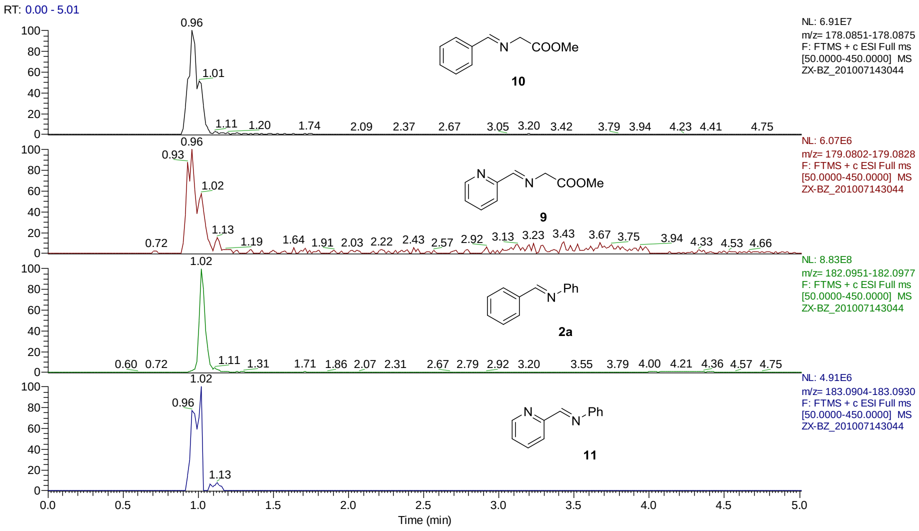

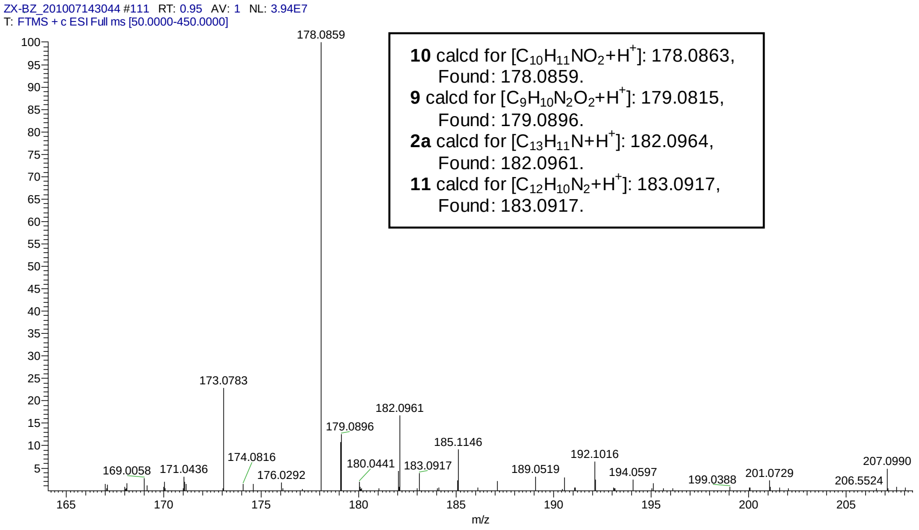

Possible side reactions:

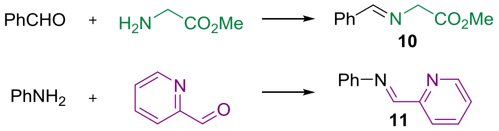

According to HRMS analysis of the reaction mixture, Schiff base **10** and picolialdehyde-derived imine **11** might exist in the reaction system. The mass balance of imines, amines and aldehydes might be the reason for low yield.

2.2 HRMS analysis of recycle product system

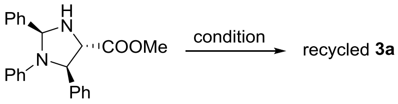

3a

The reaction was conducted with **3a** (35.6 mg, 0.10 mmol), $Yb(OTf)_3$ (6.2 mg, 10 mol%), **L-Ra(OiBu)$_2$** (8.2 mg, 10 mol%), NaO*t*Bu (9.6 mg, 0.10 mmol), EtOH (5.0 μL, 8.0 equiv) and EtOAc (0.60 mL). The mixture was stirred at 35 °C for 24 hours. T Finally, the solution was purified by flash filtration with a thin silica gel and the mixture was directly analyzed by HRMS.

Results:

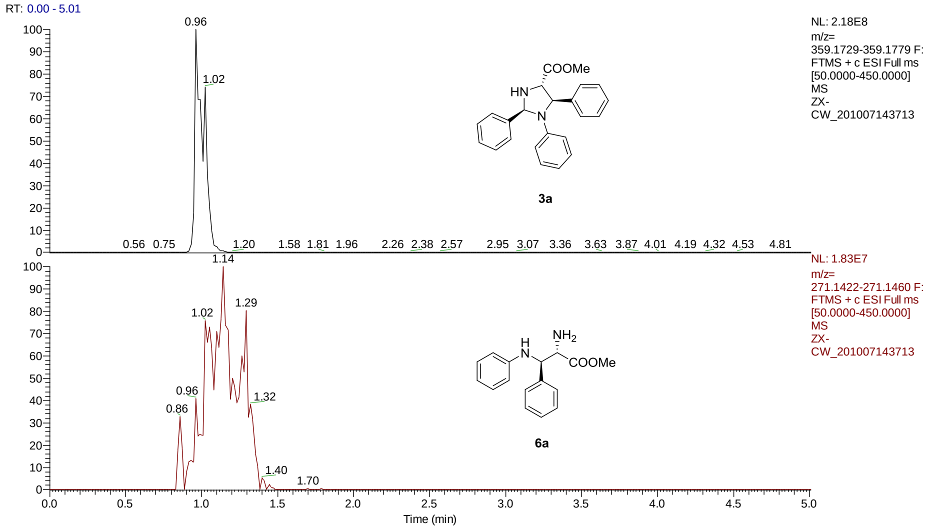

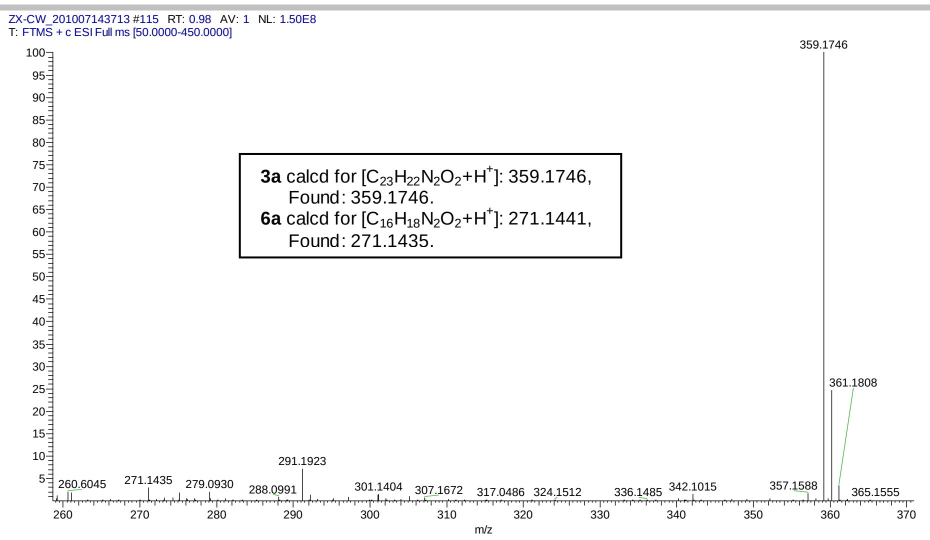

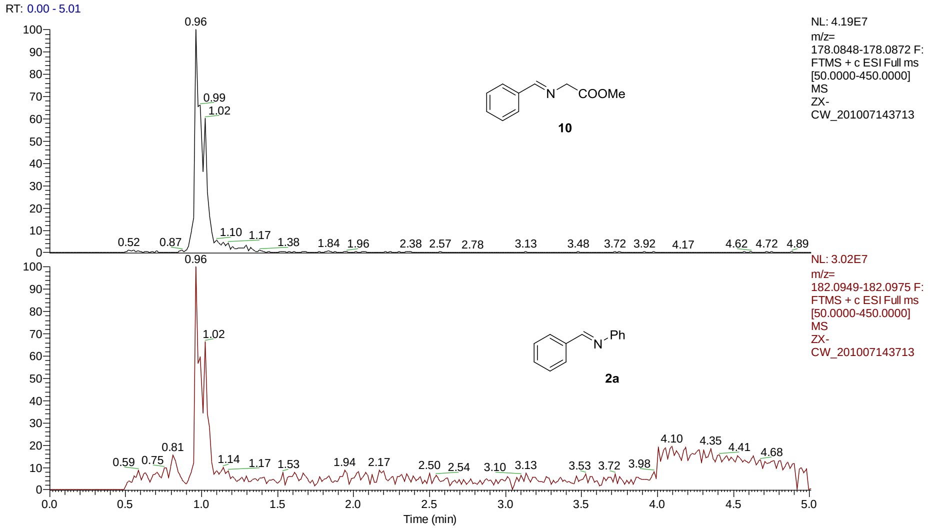

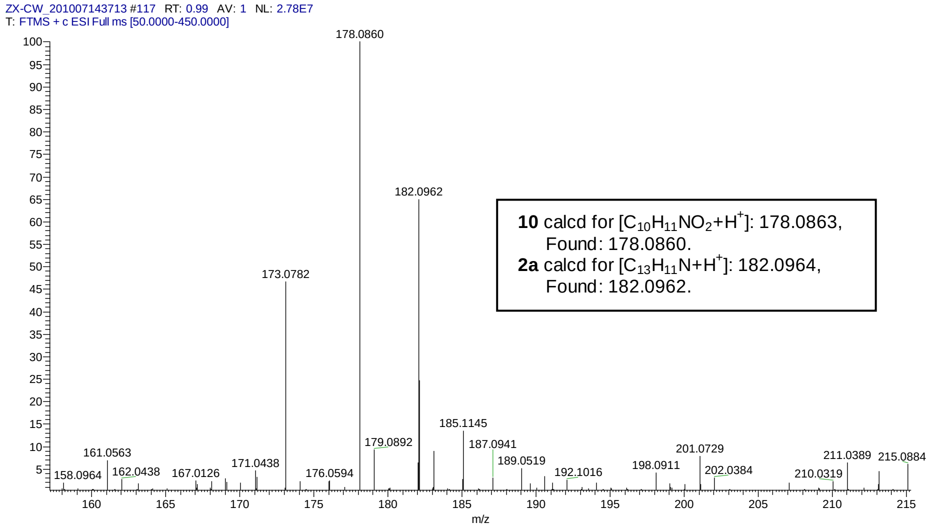

According to HRMS analysis, the product could be decomposed under the reaction and might be converted into raw material.

(I) IR Analysis and Deuterium Labeling Experiment

1. IR analysis of 9

1.1. IR spectra of **9**+Yb(OTf)$_3$ (1:1, red line) and **9** (blue line)

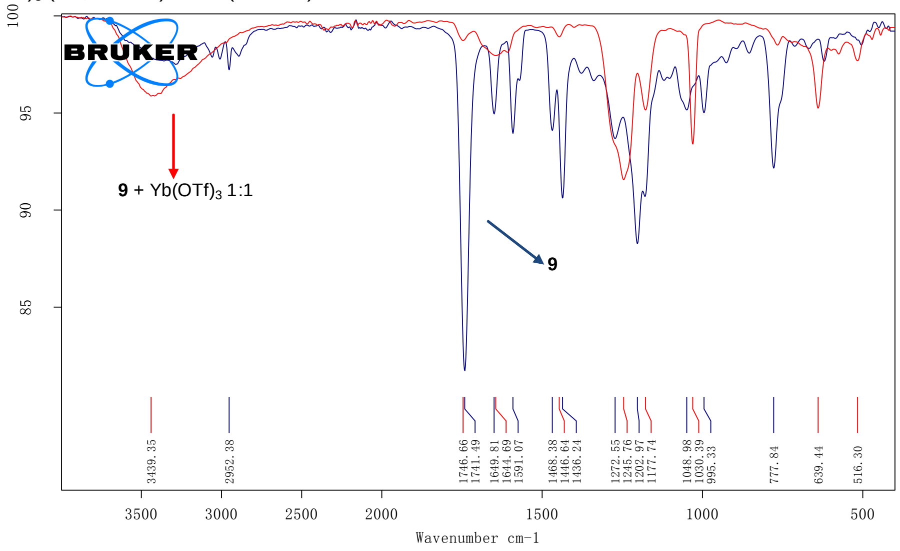

1.2. IR spectra of **9**+Yb(OTf)$_3$ (10:1, blue line) and **9** (red line)

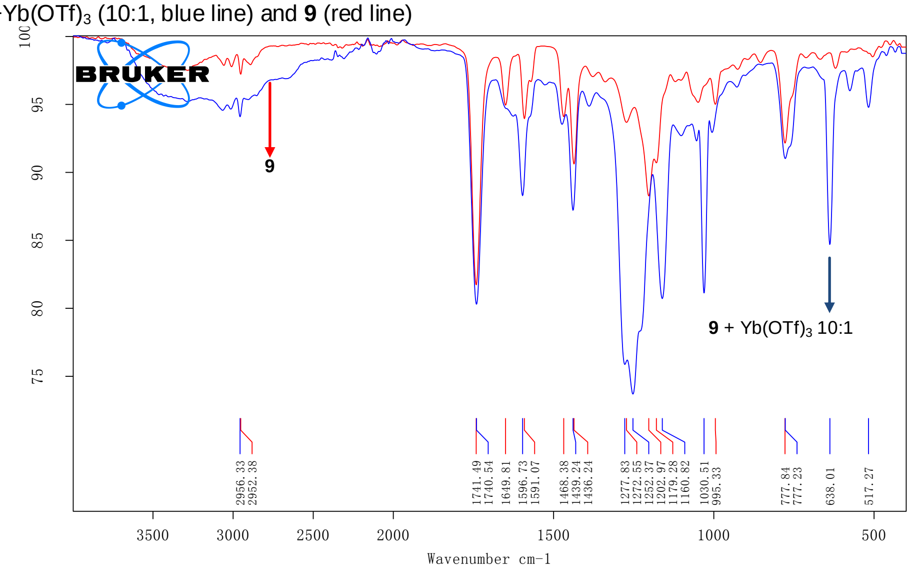

We speculated that Yb(OTf)$_3$ promote the enolization process of ester group.

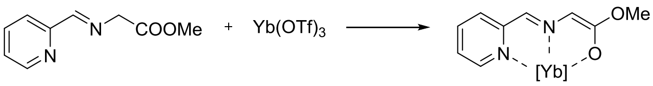

9 1.0 equiv.

2. Deuterium labeling experiment

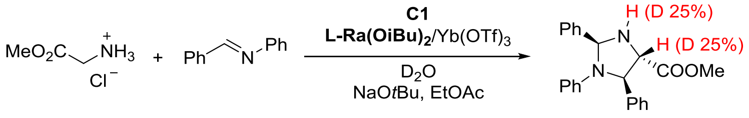

1a **2a** 30% yield, 92:8 dr, 95% ee **3a**

The reaction was conducted with Yb(OTf)$_3$ (6.2 mg, 10 mol%), **L-Ra(OiBu)$_2$** (8.2 mg, 10 mol%), NaO^{t}Bu (9.6 mg, 0.10 mmol), methyl glycine hydrochloride **1a** (12.6 mg, 0.10 mmol), and D_2O (5.0 µL). Picolinaldehyde (**C1**, 20 mol%) and EtOAc (0.60 mL) were added in succession. The mixture was stirred at 35 °C for 15 min, and imine **2a** (54.0 mg, 0.30 mmol) was added at 35 °C. The resulting mixture was stirred at 35 °C for 24 hours. The reaction mixture was subjected to column chromatography on silica gel to afford the corresponding product **3a**. The deuterium-lablled ratios the products were detected by ^{1}H NMR.

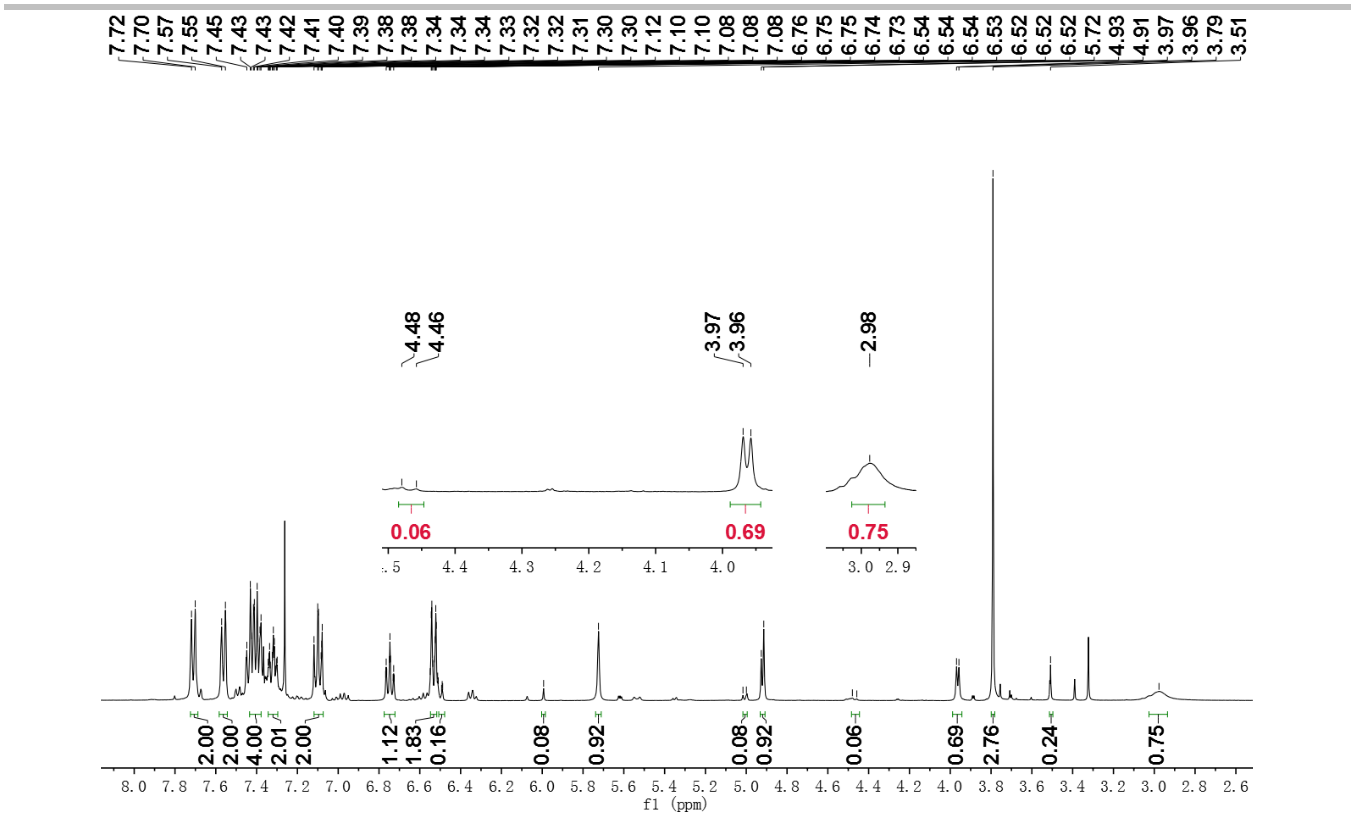
7.72
7.70
7.57
7.55
7.45
7.43
7.43
7.42
7.41
7.40
7.39
7.38
7.38
7.34
7.34
7.34
7.33
7.32
7.32
7.31
7.30
7.30
7.12
7.10
7.10
7.08
7.08
7.08
6.76
6.75
6.75
6.74
6.73
6.54
6.54
6.54
6.53
6.52
6.52
6.52
5.72
4.93
4.91
3.97
3.96
3.79
3.51
4.48
4.46
3.97
3.96
2.98
0.06
0.69
0.75
2.00
2.00
4.00
2.01
2.00
1.12
1.83
0.16
0.08
0.92
0.08
0.92
0.06
0.69
2.76
0.24
0.75
f1 (ppm)

(J) HRMS Analysis

1 The mixture of **L-Ra(OiBu)$_2$**, Yb(OTf)$_3$ (1:1) in MeOH

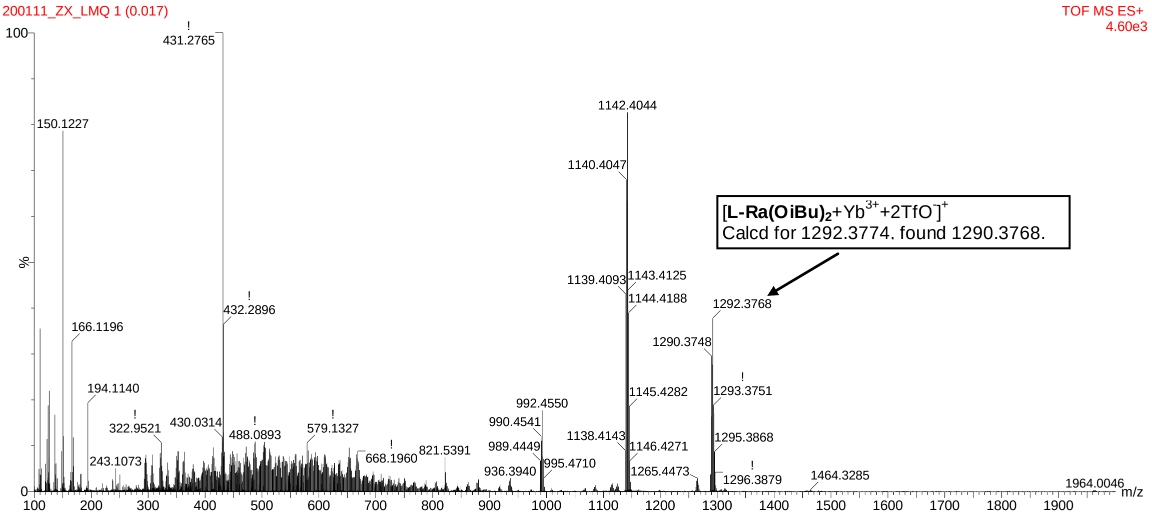

2 The mixture of **L-Ra(OiBu)$_2$**, Yb(OTf)$_3$, **C1**, **1a**, NaO*t*Bu (1:1:1:1:1) in MeOH

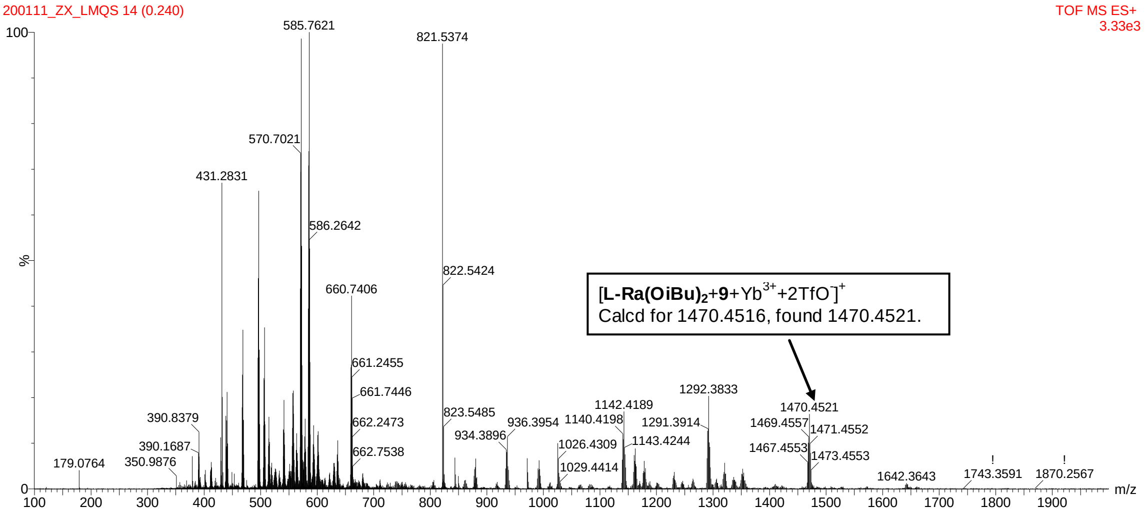

(K) Computational Details

All the geometry optimizations and related single point energy calculations were performed by Gaussian 09[4] at the B3LYP[5] level of density functional theory with consideration of the Grimme's dispersion correction[6]. For basis set, the relativistic effective core potential MWB59[7] for Ytterbium atom, and the rest atoms C, H, O, N, F and S were used in the 6-31G(d) basis set[8]. Frequency calculations on the same theoretical level were obtained to confirm local minimum structures (no imaginary frequency) or transition states (only one imaginary frequency). The single point energies were further estimated using relativistic effective core potential MWB59 for Ytterbium atom and a larger basis set 6-311+G(d,p) for all other atoms with the SMD solvation model.[9] The experimental solvent ethanol was used in our solvent correction. The CYLview software[10] were used to illustrate all the 3D structures.

Table S10. Electronic Energy and imaginary vibrational frequency of calculated structures. E: Optimized electronic energy. E_{SMD}: Single point energy with SMD model. The unit of energy and frequency is in Hartree and cm^{-1}, respectively.

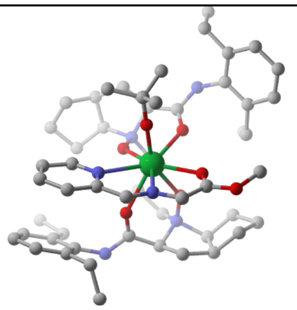

L-RaEt$_2$/Yb-*Re*

E= -2920.801507

E_{SMD}= -2921.738239

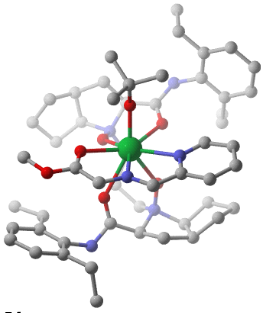

L-RaEt$_2$/Yb-*Si*

E= -2920.807476

E_{SMD}= -2921.73952

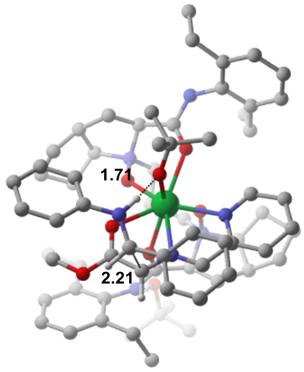

2*S*3*R*-TS

E= -3711.308938

E_{SMD}= -3712.46462

Frequency = -147.96

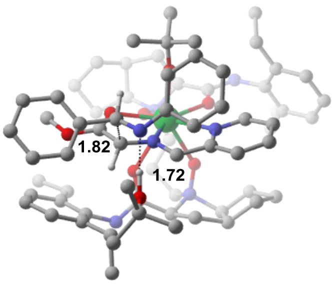

2*S*3*R*-TS_1

E= -3711.267676

E_{SMD}= -3712.443761

Frequency = -201.63

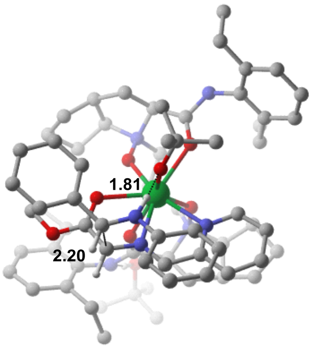

E= -3711.298452

E_{SMD}= -3712.459719

Frequency = -181.23

2*S*3*S*-TS

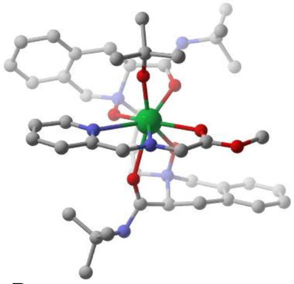

E= -2608.696608

E_{SMD}= -2609.55191

L-TQ*t*Bu/Yb-*Re*

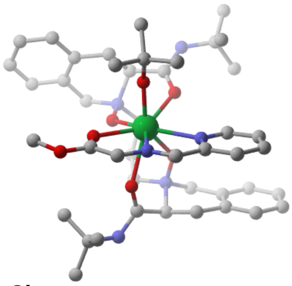

E= -2608.693725

E_{SMD}= -2609.54919

L-TQ*t*Bu/Yb-*Si*

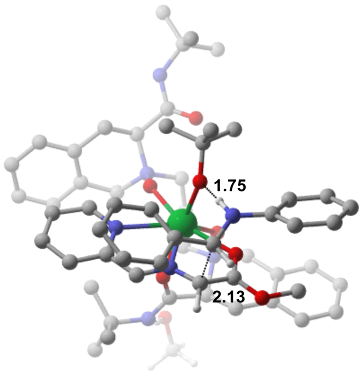

E= -3281.195274

E_{SMD}= -3282.250327

Frequency = -227.23

2*R*3*S*-TS-dis

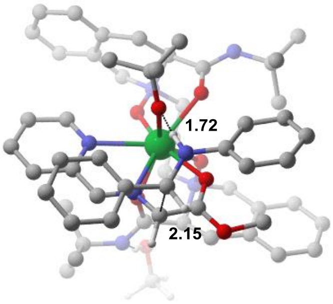

E= -3281.204233

E_{SMD}= -3282.253999

Frequency = -167.42

2*R*3*S*-TS-co

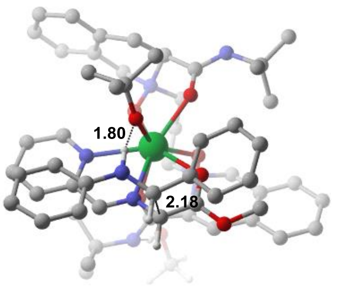

E= -3281.195765

E_{SMD}= -3281.195765

Frequency = -201.36

2*R*3*R*-TS-co

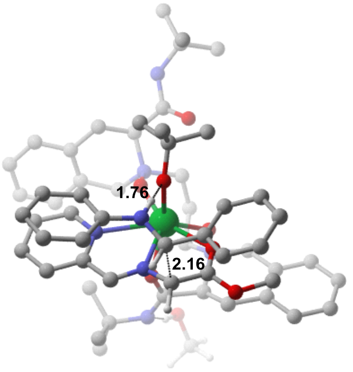

E= -3281.192563

E_{SMD}= -3282.249692

Frequency = -221.04

2*R*3*R*-TS-dis

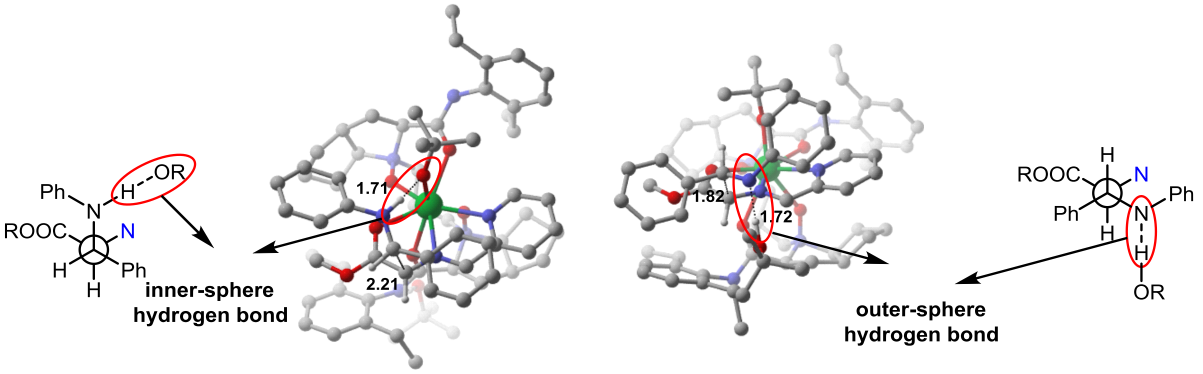

**2*S*3*R*-TS**	**2*S*3*R*-TS_1**
**0**	**6.1kcal/mol**

Figure S5. Relative free energy calculations for inner and outer-sphere hydrogen bond interaction in the transition state **2*S*3*R*-TS** by using **L-RaEt$_2$**.

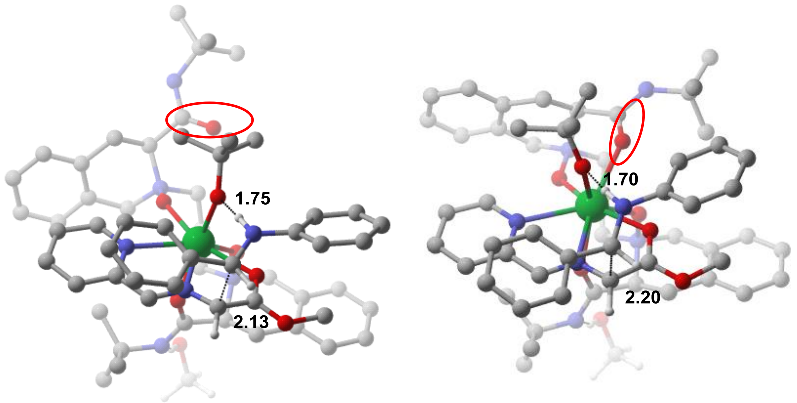

2*R*3*S*-TS-dis 0

2*R*3*S*-TS-co 0.8 kcal/mol

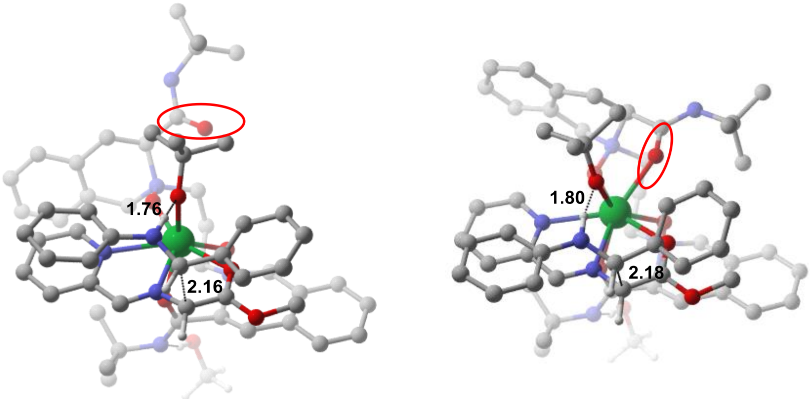

2*R*3*R*-TS-dis 1.1 kcal/mol

2*R*3*R*-TS-co 3.6kcal/mol

Figure S6. Relative free energy calculations for dissociation and coordination modes of the transition state **2*R*3*S*-TS and 2*R*3*R*-TS** by using **L-TQ^{t}Bu**.

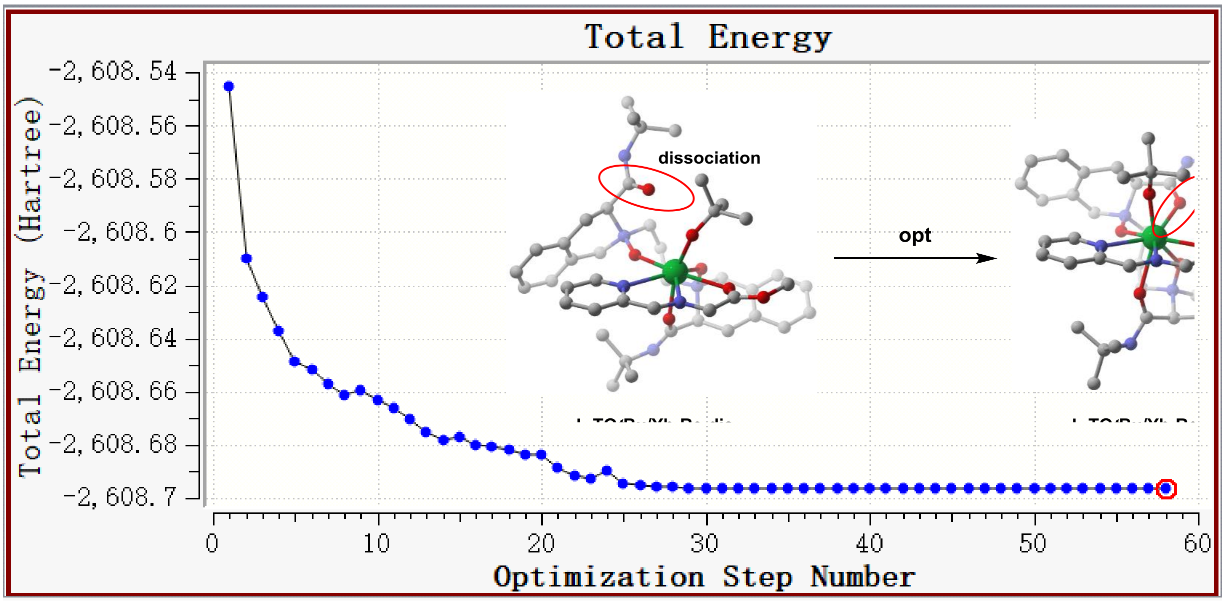

Total Energy(Hartree) curve during optimization

Figure S7. Structure optimization based on **2*R*3*S*-TS-dis** when the protonated aldimine is removed, indicating the ligand dissociation can be recovered to bind with Yb^{III} center.

Cartesian coordinates of calculated structures

L-RaEt$_2$/Yb-*Si*

Yb	-0.00733800	-0.16747400	0.56089800
O	0.14023200	-1.78747300	-0.99634600
O	-2.17065300	-0.23087000	-0.60353000
O	-0.11574800	1.38917800	-1.42860300
O	2.23474300	0.31786300	-0.48633700
N	-0.62024600	-2.12662900	-2.07656500
N	-4.11443400	-1.31276100	-1.02853400
H	-4.52793900	-2.18587700	-1.33197900
N	0.69408700	1.93255300	-2.38176400
N	4.14878300	1.32453100	-1.13857500
C	-0.57442900	-1.03899300	-3.13822700
H	-1.22191400	-0.25400700	-2.75632500
H	-0.99338100	-1.45874200	-4.05785000
C	0.82970800	-0.45439300	-3.32833700
H	1.39388500	-0.62778200	-2.41445300
H	1.35420900	-0.95205700	-4.15043500
C	0.77953500	1.04702700	-3.62542300
H	1.65787900	1.37112500	-4.19114000
H	-0.10630400	1.29748700	-4.21518200
C	-0.16422800	-3.53384800	-2.52481600
H	-0.63493300	-3.70644700	-3.49734600
C	1.36051200	-3.72518000	-2.53494300
H	1.83165300	-2.83321600	-2.12303900
H	1.74670200	-3.89327600	-3.54475800
C	1.61572000	-4.91375800	-1.57741000
H	1.54383200	-5.86385000	-2.12209100
H	2.61280800	-4.86885500	-1.12835200
C	0.48480900	-4.80467200	-0.54092000
H	0.69480900	-3.98647600	0.15520500
H	0.33619600	-5.72597500	0.03106200
C	-0.74139700	-4.44164900	-1.39998200
H	-1.14760700	-5.34706900	-1.86465200
C	-1.86725200	-3.62842900	-0.71165300
H	-2.80171800	-4.18726500	-0.60871400
H	-1.56352900	-3.26160000	0.27290700
C	-2.05570300	-2.42424200	-1.64625500
H	-2.58534000	-2.71937800	-2.56105100
C	-2.76624300	-1.22135500	-1.03311300
C	-4.96931600	-0.38304600	-0.33237900
C	-5.60980600	-0.82998800	0.83627600
C	-6.42468000	0.07553500	1.52475800
H	-6.92016300	-0.24765900	2.43614400
C	-6.58770400	1.38098300	1.06624400
H	-7.21595200	2.07524100	1.61677500
C	-5.94583500	1.79618900	-0.09992800
H	-6.08285000	2.81214600	-0.46157200
C	-5.12866300	0.92292800	-0.82804600
C	-5.44927100	-2.25228800	1.32571400
H	-5.64842300	-2.28579700	2.40187600
H	-4.40865800	-2.57023600	1.20173900
C	-6.39055000	-3.23953200	0.61055100
H	-6.25067200	-4.25647500	0.99218400
H	-6.22003400	-3.26452600	-0.47466000
H	-7.43789400	-2.95776200	0.76046100
C	-4.47815100	1.38465800	-2.11468800
H	-3.41469000	1.13079200	-2.10142800
H	-4.52790100	2.47904600	-2.15915200
C	-5.14149600	0.79905200	-3.37356900
H	-4.64879600	1.17009500	-4.27967500
H	-6.19942500	1.07674100	-3.42504000
H	-5.08244100	-0.29486800	-3.37937300
C	0.15568200	3.33488200	-2.80550800
H	0.64864400	3.55581900	-3.75704000
C	-1.37366900	3.39781800	-2.87793300
H	-1.76306600	2.48915800	-2.41402600
H	-1.74588800	3.46551100	-3.90452400
C	-1.75272900	4.61929400	-2.00776600
H	-1.71548100	5.53984800	-2.60400600
H	-2.76376100	4.53300000	-1.59673800
C	-0.67358900	4.63925100	-0.91432000
H	-0.60682500	5.59590700	-0.38777500
H	-0.88620200	3.86231000	-0.17940400
C	0.61809000	4.28244500	-1.67056200
H	1.04591400	5.18312800	-2.12467000
C	1.70316100	3.51022700	-0.88661700
H	2.59425100	4.10714600	-0.67321300
H	1.31737600	3.12479300	0.05961900
C	2.05653900	2.33636600	-1.81153400
H	2.64379600	2.69717500	-2.66411500
C	2.80137100	1.22033300	-1.09889700
C	5.03156200	0.52261300	-0.32680900
C	5.62560600	1.12961000	0.79114100
C	6.52763600	0.37158700	1.54469000

H	6.99711400	0.81795200	2.41712700
C	6.81087000	-0.94587000	1.19664600
H	7.51443900	-1.52336800	1.78945500
C	6.17916700	-1.53649800	0.10256600
H	6.39945700	-2.56998600	-0.14072100
C	5.26589900	-0.81910400	-0.67751400
C	5.30178100	2.55564300	1.18319000
H	5.50702800	2.68036400	2.25212000
H	4.22738200	2.73914000	1.05676200
C	6.10982300	3.60546900	0.39897900
H	5.85600000	4.61981900	0.72565600
H	5.92376500	3.54942100	-0.68189600
H	7.18461400	3.45768600	0.54618600
C	4.50677700	-1.45078000	-1.82834500
H	3.45461700	-1.50929200	-1.52277300
H	4.53054500	-0.76763300	-2.68864000
C	4.97875000	-2.83820200	-2.26913300
H	4.38843700	-3.18028700	-3.12561200
H	4.85410500	-3.57925800	-1.47151800
H	6.03323000	-2.83496500	-2.56575200
O	-1.21613300	-1.26813000	1.82964200
C	-0.32043400	2.71278000	2.29194800
C	-0.85606100	3.91808300	2.79991700
C	-2.17030700	4.25838400	2.51968200
C	-2.95060500	3.40000500	1.73250100
C	-2.35366000	2.24425900	1.24129300
N	-1.07676500	1.90859500	1.48507000
H	-2.59135800	5.17754400	2.91824300
H	-0.23608200	4.55251100	3.42639900
H	-3.99101300	3.60877300	1.50958700
H	-2.90495000	1.54611200	0.62367400
C	1.00695300	2.26867800	2.63402500
H	1.61447400	2.87815900	3.30659300
N	1.39915600	1.07146900	2.24228300
C	2.52105800	0.43908400	2.59456700
H	3.26758100	0.87037900	3.25775900
C	2.63050400	-0.90652300	2.14269400
O	1.76993100	-1.48580600	1.43138800
O	3.73571700	-1.55572300	2.55148600
C	3.83645300	-2.92877300	2.15641500
H	4.78713500	-3.27819400	2.56028500
H	3.00661300	-3.51176000	2.56699600
H	3.83293900	-3.01854000	1.06636600
C	-1.57997800	-1.60881300	3.14243600
C	-0.38944700	-1.38766900	4.09403700
H	0.45776100	-2.00360400	3.77621700
H	-0.07454500	-0.33929700	4.05798500
H	-0.64240500	-1.64105300	5.13082800
C	-2.76122300	-0.71914000	3.57248000
H	-3.58523800	-0.81894100	2.85842300
H	-3.12851600	-0.97648100	4.57391100
H	-2.45198600	0.33170300	3.57406800
C	-1.98652900	-3.09331500	3.17371400
H	-2.27617600	-3.41914100	4.18012000
H	-2.83330800	-3.27108600	2.50104400
H	-1.14710200	-3.71257900	2.83623600
H	4.55012100	2.13245500	-1.59821100

L-RaEt$_2$/Yb-*Re*

Yb	-0.11766100	0.10730800	0.71892900
O	0.32255600	-1.43154500	-0.97067800
O	-2.15682600	-0.27389000	-0.47667800
O	-0.21300400	1.75830000	-1.28170600
O	2.10666900	0.90598200	-0.01600100
N	-0.33036400	-1.71012000	-2.14274700
N	-3.95021500	-1.38119500	-1.30183700
H	-4.24793100	-2.19803200	-1.82002600
N	0.62419600	2.44040900	-2.11228400
N	3.96916600	1.55274800	-1.11897600
C	-0.37146300	-0.50411900	-3.05988200
H	-1.05318000	0.19395700	-2.58141400
H	-0.77720200	-0.83504200	-4.02103600
C	0.99428100	0.16884300	-3.19528700
H	1.55112100	-0.02892500	-2.28044400
H	1.56026900	-0.25247100	-4.03243500
C	0.85538600	1.67787100	-3.40873000
H	1.74337800	2.10014800	-3.88812300
H	-0.00780700	1.90499400	-4.04028100
C	0.31400000	-2.96790300	-2.76940700
H	-0.02716700	-2.98546300	-3.80818700
C	1.84259700	-3.03332000	-2.63758400
H	2.17122200	-2.24938100	-1.95305500
H	2.34607900	-2.88936800	-3.59776900
C	2.11994400	-4.42006900	-2.01103700
H	2.18303300	-5.18514700	-2.79490100
H	3.06647000	-4.43986300	-1.46085300

C	0.89808800	-4.68487800	-1.11295900
H	0.98219900	-4.12491000	-0.17665800
H	0.77814700	-5.74237500	-0.85895900
C	-0.28478400	-4.13720600	-1.93272100
H	-0.64086600	-4.90708900	-2.62609000
C	-1.47313100	-3.53926700	-1.14235300
H	-2.35929000	-4.17929600	-1.13782000
H	-1.19524900	-3.31610000	-0.11063300
C	-1.74686600	-2.21982500	-1.87174300
H	-2.18151500	-2.41880700	-2.85969600
C	-2.62093300	-1.20003800	-1.14101900
C	-4.97183800	-0.67474900	-0.57223300
C	-5.86335700	-1.45681100	0.18963800
C	-6.85357300	-0.80305300	0.92845500
H	-7.54245800	-1.39416800	1.52582200
C	-6.95161300	0.58586500	0.91718100
H	-7.71697900	1.08361800	1.50546600
C	-6.07329500	1.33410300	0.13787800
H	-6.16832200	2.41642400	0.11077800
C	-5.07352200	0.72969600	-0.63702200
C	-5.80087900	-2.97062200	0.19123900
H	-6.26789900	-3.34274800	1.10909900
H	-4.76027400	-3.31008800	0.22883300
C	-6.51827600	-3.59702500	-1.02050600
H	-6.46732600	-4.69052800	-0.98384300
H	-6.08301800	-3.27464100	-1.97710500
H	-7.57257400	-3.30283500	-1.04059000
C	-4.20280400	1.60691400	-1.51487100
H	-3.16173700	1.53368800	-1.18865000
H	-4.50384800	2.64800500	-1.35031600
C	-4.31652400	1.30569700	-3.02030300
H	-3.72877100	2.02642000	-3.59975700
H	-5.35656600	1.37271500	-3.35725300
H	-3.95253500	0.30265400	-3.26556600
C	0.04969100	3.85183300	-2.42537600
H	0.62820400	4.21516600	-3.28073200
C	-1.46367000	3.85192300	-2.66443600
H	-1.84254700	2.86021200	-2.41044000
H	-1.72513500	4.08085300	-3.70175300
C	-2.01354900	4.88437900	-1.65197400
H	-1.97832100	5.89437200	-2.07984800
H	-3.05468700	4.67933700	-1.38257700
C	-1.05102000	4.76698600	-0.45998300
H	-1.11656300	5.60519100	0.24061700
H	-1.25454900	3.83896000	0.07806300
C	0.32919900	4.65312800	-1.12965900
H	0.69590600	5.64763400	-1.40690300
C	1.42187100	3.86560000	-0.36615300
H	2.24627400	4.49199900	-0.01504700
H	1.00505200	3.33304900	0.49212300
C	1.92507100	2.85249500	-1.40057800
H	2.53656600	3.35494500	-2.15890800
C	2.66290600	1.67658500	-0.79812900
C	4.83302100	0.54475600	-0.55436000
C	5.20393600	0.63323900	0.79779600
C	6.05799500	-0.35738700	1.29538600
H	6.35356000	-0.31893500	2.33945600
C	6.52030300	-1.38589500	0.47994300
H	7.18324100	-2.14387000	0.88751000
C	6.13427900	-1.45121100	-0.85846900
H	6.50242200	-2.26122800	-1.47830200
C	5.27827300	-0.48772000	-1.40107600
C	4.73684700	1.75642700	1.69968600
H	4.96228900	1.48036700	2.73532500
H	3.64725200	1.84577900	1.65342900
C	5.39908900	3.10978300	1.38700600
H	5.04812200	3.87926400	2.08325000
H	5.16714500	3.44722100	0.37039300
H	6.48886500	3.04384200	1.47435900
C	4.82365700	-0.52564400	-2.85044000
H	3.73202900	-0.42409700	-2.87225700
H	5.20854200	0.36291000	-3.37464300
C	5.22120700	-1.77318100	-3.64364200
H	4.80596200	-1.72556900	-4.65597100
H	4.84356900	-2.68430600	-3.16658800
H	6.30802400	-1.86626700	-3.73771600
O	-1.01337800	-1.19189800	2.00167200
C	2.33740900	-0.46635600	3.00308400
C	3.36842800	-1.09151400	3.73647800
C	3.94651600	-2.25641200	3.25216600
C	3.50806800	-2.78064100	2.03123700
C	2.48560900	-2.10912200	1.36665100
N	1.89970100	-1.00341100	1.83248700
H	4.73639100	-2.74852500	3.81314700
H	3.69176900	-0.65667300	4.67784300
H	3.94496800	-3.67778800	1.60607700
H	2.10066900	-2.46163800	0.41548500
C	1.70033400	0.74153900	3.47581500

H	2.00779900	1.15289000	4.44050000
N	0.71377200	1.26774600	2.79277400
C	-0.05068000	2.30475000	3.18796200
H	0.09939100	2.82481600	4.13116800
C	-1.19729600	2.53306700	2.39937800
O	-1.45899400	1.90268200	1.32963500
O	-2.04934000	3.47756900	2.84744100
C	-3.32315500	3.54571800	2.19035000
H	-3.90884800	4.25944400	2.77153200
H	-3.21922500	3.89660800	1.15956400
H	-3.81132400	2.56663100	2.17923400
C	-1.62715400	-2.08224800	2.88070300
C	-2.99581100	-2.49732500	2.31405200
H	-3.60758400	-1.60853000	2.12834800
H	-2.85985500	-3.02210400	1.36131800
H	-3.53867700	-3.16618600	2.99327600
C	-0.72367300	-3.31811200	3.05527500
H	-0.56529600	-3.81012300	2.08690000
H	0.25594500	-3.00980000	3.43500200
H	-1.15565000	-4.05133100	3.74749200
C	-1.81721200	-1.37584000	4.23613500
H	-2.29218300	-2.02731500	4.98014800
H	-0.84352100	-1.05352100	4.62071200
H	-2.43882500	-0.48377400	4.10109200
H	4.36288900	2.19013300	-1.80004200

2*S*3*R*-TS

Yb	0.48472000	0.16611300	0.09757800
O	-0.00435000	-0.25448600	-2.06456400
O	2.25835200	-1.24807900	-0.87347300
O	-0.09108800	-2.15925900	0.52297900
O	-1.81980100	0.04842200	0.41091200
N	0.25180200	-1.28014900	-2.93399500
N	3.71886000	-2.27978100	-2.26820600
H	3.94303800	-2.47566800	-3.23601200
N	-1.24781200	-2.89728000	0.69547700
N	-4.00862600	-0.71556800	0.23575600
H	-4.71243100	-2.34860000	-0.12984700
C	-0.18382400	-2.62539600	-2.39064400
H	0.51460900	-2.84019700	-1.58621900
H	-0.05518900	-3.35230100	-3.19892900
C	-1.59306800	-2.62782300	-1.80881500
H	-1.85204200	-1.61452700	-1.50767400
H	-2.33385600	-2.94778900	-2.54701100
C	-1.67448400	-3.56504900	-0.60268900
H	-2.70081400	-3.91276200	-0.46154700
H	-1.01066600	-4.42623900	-0.72743100
C	-0.35420100	-0.89709500	-4.30128400
H	-0.35672200	-1.81837000	-4.89093800
C	-1.73034200	-0.22233400	-4.21259800
H	-1.93392800	0.02171500	-3.16878700
H	-2.53162900	-0.86257100	-4.58763800
C	-1.56851900	1.09107000	-5.00941600
H	-1.72770400	0.91529900	-6.08105900
H	-2.28638500	1.85299800	-4.69051500
C	-0.10889500	1.49529100	-4.74429200
H	-0.01933300	1.89497200	-3.72857100
H	0.26634200	2.24499000	-5.44826200
C	0.65208200	0.15880200	-4.84246800
H	0.86414100	-0.06983700	-5.89260000
C	1.93712400	0.02094100	-3.98824900
H	2.85780900	0.01131700	-4.57743700
H	2.00028600	0.82384600	-3.25639100
C	1.73402200	-1.31480200	-3.26006300
H	1.86579800	-2.14490600	-3.96519600
C	2.58105300	-1.59476900	-2.01627600
C	4.74346100	-2.53087900	-1.28782500
C	5.99675900	-1.91971800	-1.46961500
C	6.97267600	-2.12262700	-0.48738300
H	7.94491200	-1.65190500	-0.60551700
C	6.70933700	-2.90906800	0.63138500
H	7.47346900	-3.04708400	1.39078000
C	5.47158000	-3.53672000	0.76468000
H	5.28130700	-4.18119600	1.61930300
C	4.46726800	-3.37598900	-0.19738200
C	6.32770100	-1.12965300	-2.71775300
H	7.13429700	-0.42376900	-2.49228300
H	5.47165800	-0.52300400	-3.02811800
C	6.76692600	-2.04212100	-3.87946200
H	7.00742200	-1.45288000	-4.77062400
H	5.98360600	-2.76188600	-4.15399500
H	7.65137300	-2.62542600	-3.60381300
C	3.16976700	-4.14525400	-0.07016800
H	2.31430700	-3.46638100	-0.12263500
H	3.13501000	-4.59798400	0.92680300
C	3.00826100	-5.25783000	-1.12079200

H	2.06811900	-5.79846800	-0.96108600
H	3.82927400	-5.97976600	-1.06164100
H	2.99297300	-4.84942100	-2.13691300
C	-1.06836700	-3.98745300	1.79057000
H	-1.77214100	-4.77574300	1.51346400
C	0.35806700	-4.50375300	1.96045200
H	1.02848000	-3.72513200	1.59818300
H	0.55730000	-5.42495100	1.40336500
C	0.51701300	-4.65354000	3.48674500
H	0.04916300	-5.58322800	3.83508600
H	1.56836900	-4.68185000	3.79354900
C	-0.23892700	-3.43324800	4.04300600
H	-0.49919600	-3.53462300	5.10120300
H	0.37957900	-2.53808700	3.94050600
C	-1.47676100	-3.31537800	3.13436600
H	-2.30891300	-3.88505700	3.56222700
C	-1.96748700	-1.90915300	2.75017300
H	-2.80377900	-1.55594300	3.35953400
H	-1.16564400	-1.17109800	2.80276200
C	-2.40585200	-2.09236000	1.30375400
H	-3.25848100	-2.76815200	1.26275800
C	-2.74999100	-0.81359000	0.56926300
C	-4.54815400	0.50220100	-0.24310400
C	-4.72119000	1.59726400	0.63308000
C	-5.37682900	2.74073000	0.16374000
H	-5.51242000	3.58290700	0.83855900
C	-5.85314400	2.81415800	-1.14241700
H	-6.36271100	3.70836800	-1.49145300
C	-5.67921000	1.72729100	-2.00050100
H	-6.05992800	1.78982800	-3.01484100
C	-5.03985800	0.55995800	-1.56937200
C	-4.28133100	1.52044500	2.08055800
H	-4.24010400	2.53697700	2.49239600
H	-3.26457400	1.12073600	2.14405500
C	-5.22533800	0.66330600	2.94266400
H	-4.87388200	0.61026900	3.98062100
H	-5.28547400	-0.35458400	2.54531300
H	-6.23758700	1.08201400	2.94784500
C	-4.87893700	-0.65324200	-2.46736500
H	-3.83214000	-0.97184800	-2.43596900
H	-5.43009800	-1.49709500	-2.03456800
C	-5.30507400	-0.47217100	-3.92598800
H	-5.11568400	-1.39093500	-4.49295700
H	-4.75556500	0.34329900	-4.41206600
H	-6.37450700	-0.25046500	-4.01199500
O	2.22700700	1.61757700	-0.01118500
C	1.07040300	0.22752700	3.44347100
C	1.59418500	-0.06182800	4.71372500
C	2.57857900	-1.03808200	4.83767700
C	3.00755700	-1.71977200	3.69591800
C	2.42024000	-1.39497400	2.47435500
N	1.48353000	-0.44911100	2.34125100
H	3.00506700	-1.26448000	5.81055300
H	1.24501100	0.49733500	5.57561200
H	3.77702500	-2.48275300	3.74171300
H	2.71479700	-1.89376400	1.55862200
C	0.10474300	1.30425100	3.24572100
H	-0.27793700	1.82818000	4.12399400
N	-0.23175100	1.63300700	2.03508900
C	-1.00405300	2.71088400	1.70975300
H	-1.68607900	3.15712200	2.42919400
C	-1.31453700	2.78673600	0.28873600
O	-0.65584600	2.17086600	-0.56589900
O	-2.24264800	3.67845100	-0.02716800
C	-2.55430200	3.80092200	-1.43523000
H	-3.34579900	4.54578100	-1.48450700
H	-1.66806200	4.12425700	-1.98523100
H	-2.91810300	2.84208500	-1.80985400
N	1.16514400	4.12054300	0.44408500
C	0.47341500	4.35124000	1.58335200
H	-0.27654600	5.13499900	1.51172300
C	0.90174800	4.77992800	-0.77497800
C	0.41705900	6.09527800	-0.82274600
C	1.17430900	4.10137800	-1.97531400
C	0.17758000	6.70634100	-2.05434900
H	0.24918400	6.65396400	0.09275500
C	0.95425700	4.73081300	-3.19671300
H	1.51808600	3.07539500	-1.91842100
C	0.44528000	6.03269900	-3.24736100
H	-0.20175900	7.72402500	-2.07645300
H	1.16915700	4.19499700	-4.11685500
H	0.26888200	6.51805500	-4.20251100
C	1.03903200	4.16349400	2.92036000
C	2.11511100	3.29562100	3.17633400
C	0.44937200	4.84750800	4.00089600
C	2.63276700	3.17273200	4.46706900
H	2.53172100	2.71269100	2.36560000
C	0.95288700	4.70563000	5.29000700

H	-0.39843700	5.50458600	3.82078400
C	2.05616000	3.87441600	5.52539800
H	3.47399500	2.50862000	4.64214500
H	0.49820300	5.25297300	6.11050900
H	2.45851500	3.77808900	6.52987700
H	1.64431000	3.18962300	0.34203800
C	3.64283600	1.73829100	-0.15158300
C	4.35503500	0.75117900	0.78860500
H	4.06483600	0.94259600	1.82755600
H	4.07946600	-0.27535700	0.53841800
H	5.44595500	0.83678300	0.71552200
C	4.06500400	1.45858700	-1.60504700
H	3.79582500	0.44548000	-1.89445700
H	3.57551500	2.16167400	-2.28696700
H	5.14850200	1.56656200	-1.73123800
C	4.13604200	3.17233500	0.15905000
H	5.22190400	3.23076600	0.02224800
H	3.67799500	3.90244500	-0.51593400
H	3.92367500	3.47898000	1.18541500
C	-6.07428500	-3.69313600	0.39836800
C	-5.78451200	-3.70038700	1.90912800
H	-4.95492100	-4.38348700	2.13073600
H	-5.50874600	-2.69538500	2.24929900
H	-6.65828900	-4.02583300	2.48503400
C	-6.41411400	-5.10114700	-0.09217800
H	-7.31066500	-5.48682100	0.40490500
H	-6.58921100	-5.09003000	-1.17297300
H	-5.58194200	-5.78460000	0.11255400
C	-7.20880100	-2.70865600	0.07630400
H	-6.93249500	-1.69099900	0.37664200
H	-7.41004900	-2.70450200	-1.00041600
H	-8.13239800	-2.98106200	0.59962000
O	-4.89007400	-3.30822200	-0.31824600

2*S*3*R*-TS_1

Yb	0.83337800	-0.28084400	-0.48899600
O	1.81351900	-2.28241200	-0.74779300
O	3.20995000	0.12096300	-0.37954800
O	1.87825700	-0.54610300	1.83103100
O	-0.51760500	-1.73002300	1.15831500
N	3.07249800	-2.77562400	-0.59499200
N	5.42554000	0.03242100	-0.75966100
H	6.21541400	-0.52796800	-1.05211100
N	1.67278000	-1.15907500	3.03186900
N	-1.78556100	-2.45359800	2.88678900
H	-1.93320800	-2.39646500	3.88736400
C	3.45340700	-2.87023000	0.87313400
H	3.73226100	-1.85628600	1.15162900
H	4.32546400	-3.52718200	0.94401500
C	2.29421000	-3.30668100	1.77249000
H	1.36088400	-3.10642800	1.24950200
H	2.34361500	-4.37912800	1.98641400
C	2.32051000	-2.54502100	3.09835000
H	1.81102400	-3.10020100	3.89063300
H	3.34961600	-2.37266800	3.42512800
C	3.14053300	-4.08592800	-1.40951800
H	4.07262300	-4.57682300	-1.11377300
C	1.89860700	-4.97945600	-1.25778300
H	1.16160300	-4.46378600	-0.64058600
H	2.14082700	-5.93759200	-0.78840200
C	1.34539900	-5.13268700	-2.69644500
H	1.81696100	-5.99037400	-3.19257900
H	0.26460600	-5.30670800	-2.70213400
C	1.74452900	-3.82473400	-3.40047200
H	1.08159000	-3.01234900	-3.08730000
H	1.71225100	-3.90067500	-4.49195000
C	3.16836200	-3.55764700	-2.87324500
H	3.89075500	-4.15518900	-3.44026000
C	3.62028300	-2.07448100	-2.80117300
H	4.42240300	-1.82840800	-3.50158000
H	2.78495700	-1.38952000	-2.96245600
C	4.09649800	-1.92271500	-1.35363400
H	5.05902300	-2.42842300	-1.20523400
C	4.18699200	-0.50625800	-0.79367800
C	5.67240800	1.32315400	-0.16404500
C	5.31425600	2.48770100	-0.86429900
C	5.53720400	3.71677800	-0.23014000
H	5.27371700	4.63234000	-0.75306900
C	6.08661500	3.77977600	1.05036600
H	6.25115400	4.74379600	1.52332500
C	6.42528400	2.60722600	1.72415800
H	6.85124900	2.65596900	2.72295700
C	6.22015500	1.35638900	1.12977000
C	4.70710000	2.43280900	-2.24918100
H	4.28717500	3.41674300	-2.48590900
H	3.86041000	1.73799500	-2.25667500

C	5.71851500	2.04452900	-3.34162700
H	5.23744600	2.04530400	-4.32505400
H	6.12716400	1.04236400	-3.16984200
H	6.55694700	2.74856900	-3.37107800
C	6.61343000	0.08830200	1.85766800
H	5.85510000	-0.68854500	1.69751700
H	6.62703700	0.28648600	2.93510000
C	7.99322800	-0.44173000	1.42728800
H	8.25483300	-1.35196000	1.97778200
H	8.77060500	0.30605800	1.61372100
H	8.02277800	-0.67758600	0.35609600
C	2.24860400	-0.27997000	4.18043900
H	2.34872400	-0.95243900	5.03782800
C	3.55576400	0.42764300	3.80412000
H	3.68937600	0.34052800	2.72242400
H	4.42671500	-0.00262800	4.30768500
C	3.31059500	1.90940100	4.17189500
H	3.54085900	2.08429300	5.23056700
H	3.93825700	2.58315000	3.57972000
C	1.80849700	2.11027500	3.90910800
H	1.39894000	3.00012200	4.39638300
H	1.63665400	2.20402300	2.83640000
C	1.16712100	0.80509700	4.41466700
H	0.97602800	0.87680800	5.49096000
C	-0.10093300	0.30776500	3.68187600
H	-1.01659700	0.41877900	4.26859100
H	-0.23999000	0.82557900	2.73329100
C	0.19317700	-1.17386300	3.41342000
H	0.13167900	-1.73552700	4.35299000
C	-0.71251800	-1.81473100	2.37243300
C	-2.91522700	-2.86754500	2.08929400
C	-4.05757200	-2.05214200	2.11223700
C	-5.17090300	-2.47392900	1.37706600
H	-6.05749600	-1.84823600	1.35428000
C	-5.13589400	-3.66417700	0.65720500
H	-6.00578500	-3.97299500	0.08497400
C	-3.97857300	-4.44418300	0.63685100
H	-3.96713700	-5.35985600	0.05574600
C	-2.83288000	-4.05179700	1.33642900
C	-4.09078500	-0.75295800	2.88639600
H	-4.74563700	-0.03925900	2.38098600
H	-3.10063300	-0.28110500	2.86938100
C	-4.55078900	-0.93016600	4.34345900
H	-4.57902100	0.03572700	4.85955900
H	-3.88741400	-1.59750300	4.91182900
H	-5.55449600	-1.36622400	4.38345600
C	-1.52341900	-4.81658000	1.26660600
H	-0.79532900	-4.17089500	0.75667900
H	-1.13491200	-4.95215700	2.28547400
C	-1.56774300	-6.17291500	0.55870700
H	-0.58094900	-6.64710500	0.59298100
H	-1.84459300	-6.07096500	-0.49681100
H	-2.28475000	-6.85390600	1.02972800
O	1.27101500	0.36662700	-2.40098900
C	-0.35025000	2.60947600	0.77847400
C	-0.44326500	3.91723400	1.27166500
C	0.69721600	4.71761700	1.28946600
C	1.90677300	4.18748600	0.83848100
C	1.92722300	2.86662800	0.38868700
N	0.83612100	2.09250600	0.35545600
H	0.63913500	5.74218300	1.64503400
H	-1.40564700	4.29749500	1.59281100
H	2.82268900	4.76849700	0.83744000
H	2.84461700	2.39548300	0.05841000
C	-1.53204700	1.76059000	0.63857300
H	-2.46105700	2.05749800	1.11932900
N	-1.40517900	0.67497300	-0.07054400
C	-2.53979300	-0.03040900	-0.48191900
H	-3.32599100	-0.18508500	0.25638200
C	-2.11534200	-1.28098800	-1.16171800
O	-0.95023000	-1.49870400	-1.53590300
O	-3.09648400	-2.13400600	-1.40436000
C	-2.77098000	-3.28077900	-2.20764900
H	-3.70366800	-3.83331400	-2.30433700
H	-2.40464300	-2.96130100	-3.18647400
H	-2.00831100	-3.88610400	-1.71086400
N	-3.64541300	2.24483800	-1.02388200
C	-3.44863300	1.03876500	-1.64952400
H	-2.76554200	1.04757200	-2.50429100
C	-3.08465800	3.41105800	-1.51424700
C	-2.52053700	3.58387100	-2.80192400
C	-3.12633500	4.55749300	-0.67792100
C	-2.00435500	4.81600700	-3.20132900
H	-2.50556700	2.76047700	-3.50792200
C	-2.61701600	5.78320100	-1.08955200
H	-3.56752100	4.44944000	0.30832800
C	-2.03896600	5.92679600	-2.35583300
H	-1.57839300	4.90824400	-4.19791400

H	-2.67235100	6.63598100	-0.41594000
H	-1.64263300	6.88424000	-2.68088900
C	-4.68470000	0.23908700	-1.98642700
C	-5.71939000	0.04565600	-1.06152800
C	-4.79172200	-0.34692300	-3.25285300
C	-6.83086300	-0.72557100	-1.39796800
H	-5.64558200	0.50020300	-0.07974300
C	-5.90439800	-1.12051000	-3.59075100
H	-3.99596600	-0.19866900	-3.98034300
C	-6.92775700	-1.31629600	-2.66162100
H	-7.62934500	-0.86291500	-0.67219900
H	-5.97365000	-1.56360600	-4.58095700
H	-7.79747000	-1.91335100	-2.92255300
H	-4.46606000	2.23576200	0.48917200
C	0.98549100	0.99963100	-3.62463500
C	-0.34541800	0.46356200	-4.17849200
H	-0.28639400	-0.62305100	-4.30566000
H	-1.14601700	0.67294900	-3.46616400
H	-0.60321700	0.92181900	-5.14102400
C	0.89134600	2.51610100	-3.38591500
H	1.84456300	2.89567900	-3.00132900
H	0.64785000	3.06207900	-4.30531900
H	0.11449300	2.73346900	-2.64777100
C	2.10625800	0.69738900	-4.63766700
H	1.95397800	1.24200500	-5.57690800
H	3.07929500	0.98738500	-4.22747800
H	2.13773600	-0.37334400	-4.87122100
C	-5.90353800	2.86320700	1.78460800
C	-6.59139800	3.40501100	0.52279200
H	-5.91381300	4.04725200	-0.04908900
H	-6.90451600	2.58422700	-0.13073900
H	-7.47750200	3.99265900	0.78826100
C	-6.85909200	1.94797000	2.56278600
H	-7.18118800	1.11208800	1.93071200
H	-6.35697700	1.53667200	3.44653200
H	-7.75048900	2.49110500	2.89637500
C	-5.43218500	4.01606400	2.68495200
H	-4.88877500	3.61494500	3.54824600
H	-4.75771800	4.68223400	2.13576300
H	-6.27584100	4.61314900	3.05085700
O	-4.76086200	2.06564500	1.43730500

2*S*3*S*-TS

Yb	0.46178100	0.12551500	-0.02965900
O	-0.25051500	-0.62173400	-2.03950100
O	2.01043300	-1.60051100	-0.88057600
O	-0.26057600	-2.02114200	0.84432400
O	-1.80897500	0.29122200	0.45395800
N	-0.16274600	-1.79176500	-2.74149300
N	3.25040200	-2.99028700	-2.17483900
H	3.36216000	-3.37208200	-3.10591000
N	-1.45547000	-2.60445900	1.22550400
N	-4.05882800	-0.29495000	0.53248000
H	-4.93495300	-1.89116800	0.56621300
C	-0.65122800	-2.98264600	-1.94184200
H	0.10256300	-3.12517000	-1.17186200
H	-0.65972400	-3.84254500	-2.61920700
C	-1.99253000	-2.75208900	-1.25570800
H	-2.13666600	-1.68212800	-1.12131800
H	-2.82400000	-3.11967600	-1.86354700
C	-2.03563500	-3.45211300	0.10320300
H	-3.06832300	-3.68080900	0.37801500
H	-1.44806800	-4.37552800	0.09235800
C	-0.86141200	-1.56675900	-4.09999200
H	-0.98554600	-2.56144000	-4.53805900
C	-2.16780400	-0.76549000	-4.00613100
H	-2.27092700	-0.38005400	-2.99100300
H	-3.04415400	-1.37197300	-4.24468700
C	-1.96507400	0.42665100	-4.96943400
H	-2.23610300	0.14482000	-5.99501600
H	-2.58423800	1.28453000	-4.68982600
C	-0.45831500	0.71693600	-4.87399000
H	-0.24124100	1.21627200	-3.92334500
H	-0.08403700	1.33978100	-5.69301000
C	0.16792300	-0.69220000	-4.87046500
H	0.24116100	-1.06601000	-5.89748300
C	1.52289100	-0.85133500	-4.13549100
H	2.35999700	-1.07261500	-4.80304400
H	1.76014000	0.04760700	-3.56928000
C	1.27268900	-2.02093100	-3.17175400
H	1.27202800	-2.96601900	-3.72881800
C	2.20119900	-2.16707500	-1.96382900
C	4.34394600	-3.17681800	-1.25669200
C	5.61814700	-2.74385000	-1.66496300
C	6.67980200	-2.89043200	-0.76596000
H	7.67098800	-2.55590000	-1.06020000

C	6.47544600	-3.44209100	0.49638700
H	7.30677900	-3.53745400	1.18877700
C	5.20610000	-3.88402600	0.86568500
H	5.05523600	-4.33742500	1.84199900
C	4.11469500	-3.77630500	-0.00458500
C	5.86274600	-2.17675800	-3.04725500
H	6.75386900	-1.54050300	-3.01867700
H	5.03428100	-1.52472000	-3.34346600
C	6.06719400	-3.27678800	-4.10679100
H	6.24909600	-2.84002900	-5.09440200
H	5.19386100	-3.93834800	-4.18941300
H	6.92148500	-3.91075100	-3.84866100
C	2.76770300	-4.33305500	0.40456300
H	1.99636900	-3.56251500	0.32252000
H	2.81583600	-4.60252600	1.46560000
C	2.34054300	-5.57613100	-0.39594000
H	1.37320500	-5.94633200	-0.03653200
H	3.07242200	-6.38385100	-0.29201800
H	2.23885100	-5.35156900	-1.46304400
C	-1.28165800	-3.49642100	2.48971500
H	-2.04781900	-4.26828900	2.38970400
C	0.11561400	-4.08011900	2.68385300
H	0.81645500	-3.42452500	2.16770100
H	0.22446400	-5.09154800	2.27903800
C	0.34007700	-3.99623000	4.20695200
H	-0.16734100	-4.82487300	4.71720600
H	1.40132000	-4.05180100	4.47390800
C	-0.30865100	-2.65405800	4.59386200
H	-0.53539200	-2.58119600	5.66202000
H	0.36585300	-1.83110100	4.34508600
C	-1.56946800	-2.57588600	3.71256500
H	-2.43058500	-2.98379700	4.25299500
C	-1.95112800	-1.21425200	3.10830200
H	-2.70833900	-0.67679500	3.68550700
H	-1.08239600	-0.56541100	2.98694100
C	-2.49926500	-1.60159700	1.74313300
H	-3.40269400	-2.19470200	1.86918000
C	-2.79184300	-0.44303000	0.81275100
C	-4.53562800	0.85378600	-0.14238900
C	-4.52342300	2.11735600	0.49031900
C	-5.11314300	3.20504700	-0.16367400
H	-5.10199800	4.17829900	0.32171900
C	-5.71079300	3.05996700	-1.41240400
H	-6.16926300	3.91397500	-1.90394900
C	-5.72817500	1.80620500	-2.02524100
H	-6.20641200	1.69903600	-2.99344800
C	-5.15515000	0.69051600	-1.40605400
C	-3.96063500	2.29486400	1.88574500
H	-3.80884600	3.36600000	2.07115700
H	-2.97451900	1.82644700	1.95794000
C	-4.88577800	1.72248500	2.97496400
H	-4.44760100	1.84996100	3.97266200
H	-5.05924800	0.65502300	2.80733400
H	-5.85868700	2.22577100	2.96440800
C	-5.19905500	-0.69130300	-2.03287400
H	-4.18777100	-1.10993900	-2.02421300
H	-5.77319700	-1.36301100	-1.38334800
C	-5.76213100	-0.76902900	-3.45396500
H	-5.70892300	-1.79853700	-3.82619400
H	-5.20402500	-0.12986200	-4.14905300
H	-6.81312600	-0.46205400	-3.49171100
O	2.39403500	1.30927100	-0.24993500
C	1.11216800	0.94040200	3.20266100
C	1.64124000	0.92932600	4.50094100
C	2.54398000	-0.06963900	4.85786900
C	2.88917100	-1.04102700	3.91672100
C	2.28864400	-0.98533400	2.65816700
N	1.42340800	-0.02978300	2.30625900
H	2.97878200	-0.08315700	5.85285300
H	1.37705600	1.71941300	5.19585500
H	3.59771500	-1.82943700	4.14660700
H	2.50929600	-1.72468300	1.89537800
C	0.27822200	2.04419300	2.72996200
H	0.01321100	2.83035400	3.44201100
N	-0.05778600	2.09424600	1.48361700
C	-0.69964000	3.15097900	0.88886500
H	-1.32008600	3.82230700	1.47920800
C	-1.10214000	2.85687700	-0.47748100
O	-0.55634900	1.97227300	-1.16217400
O	-2.00993800	3.68411400	-0.97015000
C	-2.41741100	3.44681700	-2.33496000
H	-3.14361800	4.22700800	-2.55362400
H	-1.55294800	3.51372100	-2.99881800
H	-2.89001700	2.46493400	-2.40482400
N	2.04863500	3.82072500	1.03240600
C	1.00593200	4.48631100	0.47878800
H	0.61324900	5.29343200	1.08872700
C	2.60900400	4.10839000	2.29261700

C	2.43278500	5.34700400	2.93257100
C	3.41919200	3.13839900	2.90885900
C	3.03068500	5.58803300	4.16970100
H	1.86039900	6.13935800	2.46191300
C	4.02532000	3.39796700	4.13474100
H	3.54588800	2.17464800	2.42998700
C	3.83087800	4.62093100	4.78103300
H	2.88202600	6.55221100	4.64784500
H	4.64512700	2.62996100	4.58937500
H	4.30322800	4.82206500	5.73758800
C	0.90930200	4.73692300	-0.97031800
C	1.46655800	3.87829300	-1.93429200
C	0.21111100	5.88062100	-1.39646000
C	1.35211300	4.18174300	-3.28834500
H	1.93248900	2.95543400	-1.61083600
C	0.10031200	6.18040500	-2.75239800
H	-0.24207500	6.54007100	-0.66030900
C	0.67556900	5.33280000	-3.70360500
H	1.78544400	3.50923800	-4.02414300
H	-0.43075600	7.07432800	-3.06602400
H	0.59258400	5.56712800	-4.76129700
H	2.29258300	2.90800600	0.58777500
C	3.77545100	1.20455400	-0.59529200
C	3.91969900	0.81308900	-2.07782900
H	3.49511300	-0.17207600	-2.25655500
H	3.39655600	1.53736200	-2.71255900
H	4.97103700	0.78681300	-2.38700500
C	4.47169000	0.15798000	0.29303300
H	4.41717800	0.45879600	1.34471700
H	3.98742500	-0.81350200	0.19072400
H	5.52967800	0.04084700	0.02920400
C	4.52303100	2.55137200	-0.43097800
H	4.57002100	2.87935800	0.60959500
H	5.55751700	2.44675300	-0.77767100
H	4.05512000	3.34624400	-1.01980000
C	-6.33572900	-2.96236100	1.47983800
C	-7.41711100	-1.95761400	1.05427500
H	-7.03410100	-0.93190800	1.11256300
H	-7.72494200	-2.14950600	0.02074200
H	-8.30176000	-2.02922200	1.69719100
C	-6.83179800	-4.40077800	1.32678000
H	-7.11779200	-4.59138100	0.28717600
H	-6.03712300	-5.10567700	1.59724600
H	-7.69791400	-4.59029800	1.96982700
C	-5.89704700	-2.69180400	2.92961100
H	-5.10848200	-3.39575400	3.22393200
H	-5.50729900	-1.67173500	3.02586000
H	-6.73282000	-2.80321500	3.62965500
O	-5.20399500	-2.84729000	0.60210300

L-TQ*t*Bu/Yb-*Si*

Yb	0.23228900	0.12909500	0.59933000
O	1.51415700	0.10821800	-1.33401800
O	0.01415000	-2.16472700	-0.34648000
O	-1.46484400	-0.08456000	-1.18390900
O	-0.66757900	2.37641700	-0.03316300
N	1.70855400	-0.88118100	-2.26695800
N	0.83610500	-4.19712800	-0.93302800
H	1.57109100	-4.67962100	-1.43315800
N	-1.87272200	0.80926000	-2.13724200
N	-1.42793600	4.20164900	-1.14739400
H	-2.12903100	4.54679400	-1.78966900
C	0.47520100	-1.08193000	-3.14040900
H	-0.29466800	-1.44942300	-2.46988200
H	0.73970300	-1.84530400	-3.87941800
C	-0.04529600	0.16947100	-3.85306000
H	0.75168100	0.66713700	-4.41601600
H	-0.74723300	-0.18744100	-4.61564400
C	-0.69930300	1.25893700	-2.99938800
H	0.03137200	1.65450900	-2.29949800
H	-1.06598900	2.05899200	-3.65217800
C	2.89539800	-0.46321200	-3.11339800
H	2.64652400	0.53205900	-3.48040500
H	2.95197000	-1.15199500	-3.96602300
C	4.18467600	-0.42267600	-2.33139500
C	5.23032200	0.37711300	-2.80604400
H	5.08682400	0.97538000	-3.70333400
C	6.45123100	0.41402100	-2.13619500
H	7.25819800	1.03564100	-2.51244600
C	6.62596100	-0.34378100	-0.97470900
H	7.57184900	-0.31620600	-0.44169000
C	5.58223600	-1.13362900	-0.49779700
H	5.70998900	-1.71584800	0.41162000
C	4.35634600	-1.18508400	-1.17179300
C	3.22003800	-1.99566800	-0.60760300
H	2.79986400	-1.50639200	0.28023000

H	3.56825900	-2.98189300	-0.27893100
C	2.08602600	-2.22452400	-1.60814200
H	2.41576000	-2.86285600	-2.43624700
C	0.87355700	-2.85538500	-0.90724700
C	-0.22085400	-5.05170100	-0.31165300
C	0.22181300	-6.50434000	-0.53770800
H	0.31269300	-6.73521600	-1.60666700
H	-0.51922900	-7.18682800	-0.11224700
H	1.18227800	-6.70586200	-0.04887400
C	-0.32946400	-4.75997300	1.19383700
H	0.62158500	-4.96021000	1.69599400
H	-1.09255600	-5.41127400	1.63316500
H	-0.60456800	-3.72125400	1.38106500
C	-1.55813300	-4.78499700	-1.02537100
H	-1.48132600	-5.01802500	-2.09361200
H	-1.85636000	-3.74021900	-0.91358800
H	-2.34108800	-5.41700200	-0.59358000
C	-2.94368800	0.13775400	-2.96792000
H	-3.17045900	0.79407800	-3.81857400
H	-2.49163600	-0.77883500	-3.34588800
C	-4.17235100	-0.18832900	-2.15340400
C	-5.01501100	-1.21513900	-2.59389300
H	-4.76099400	-1.77177800	-3.49345700
C	-6.17408800	-1.53105500	-1.88794800
H	-6.82306900	-2.32768100	-2.23918000
C	-6.48999300	-0.82364100	-0.72495800
H	-7.38758500	-1.06693400	-0.16409300
C	-5.64711000	0.19286500	-0.28120400
H	-5.87828900	0.72942400	0.63545100
C	-4.48709900	0.52340800	-0.98999300
C	-3.54895400	1.57719700	-0.45690000
H	-2.99375100	1.17667300	0.39519800
H	-4.10395800	2.45515600	-0.10491800
C	-2.53418200	2.05049900	-1.49469100
H	-3.02326000	2.58854500	-2.31438100
C	-1.45573100	2.89956900	-0.82904200
C	-0.47482800	5.20834800	-0.58468500
C	-0.82282300	6.54206500	-1.26053100
H	-1.85006200	6.85098800	-1.03060100
H	-0.70569700	6.48177100	-2.34976500
H	-0.15189600	7.32487700	-0.89615500
C	-0.66983100	5.31103600	0.93693100
H	-1.70072700	5.59556200	1.17559900
H	-0.00073100	6.07954300	1.33785600
H	-0.43623600	4.36536500	1.43043800
C	0.96516600	4.79175200	-0.93300300
H	1.09585300	4.72715200	-2.01975200
H	1.21841200	3.82852800	-0.48588900
H	1.66153400	5.54578500	-0.55118200
O	1.39374700	-1.08128100	1.82485500
C	-2.20489900	0.12473200	2.93019000
C	-3.35619400	-0.25366400	3.65627900
C	-4.16273500	-1.27604600	3.18257700
C	-3.82846000	-1.91853300	1.98186400
C	-2.68351600	-1.49747300	1.31696200
N	-1.88489800	-0.51561400	1.76477500
H	-5.04867800	-1.57252200	3.73772300
H	-3.59371400	0.26303100	4.58130100
H	-4.44224400	-2.71037900	1.56733200
H	-2.38164500	-1.93646400	0.37339000
C	-1.34384800	1.19172100	3.37017200
H	-1.58104000	1.71914000	4.29657600
N	-0.28453600	1.49126100	2.65264600
C	0.61752600	2.44229100	2.92219600
H	0.56867200	3.08068700	3.80174900
C	1.69515500	2.53852300	2.00427100
O	1.82974900	1.80749800	0.98485900
O	2.60394900	3.49293300	2.28333500
C	3.76892400	3.51885700	1.44579400
H	4.40502900	4.30238500	1.85965500
H	4.28361900	2.55403300	1.46876500
H	3.50216400	3.75088600	0.41073600
C	1.91698100	-1.53251500	3.04862800
C	2.81709000	-2.75962000	2.79938600
H	2.26690500	-3.51874400	2.23232600
H	3.70524100	-2.47646300	2.22247100
H	3.15807800	-3.20786700	3.73992800
C	2.75521800	-0.41656500	3.69839900
H	3.54553200	-0.09943600	3.00783000
H	2.12640200	0.45217600	3.91245500
H	3.22153600	-0.75014100	4.63375900
C	0.75338400	-1.93026600	3.97488000
H	1.10937000	-2.30343300	4.94292200
H	0.10850000	-1.06410600	4.15112400
H	0.14921300	-2.71153000	3.50005900

L-TQ*t*Bu/Yb-*Re*

Yb	0.13671900	0.01235800	0.61413300
O	1.24503300	-0.23552400	-1.43415700
O	-0.27609000	-2.27793600	-0.10767700
O	-1.68599900	-0.17806800	-1.04090900
O	-0.54432600	2.28695500	-0.16690200
N	1.28366400	-1.29680700	-2.30799200
N	0.17042900	-4.40587500	-0.74024200
H	0.77254200	-5.00165100	-1.29290900
N	-2.10949900	0.69682900	-2.00401600
N	-1.38590900	4.12123100	-1.21043100
H	-2.15568600	4.47372500	-1.76414900
C	-0.03047500	-1.44729000	-3.06380200
H	-0.76763700	-1.70405100	-2.30890800
H	0.11068200	-2.27611400	-3.76529000
C	-0.51178000	-0.20599900	-3.82089000
H	0.26627300	0.17892100	-4.48929700
H	-1.30880100	-0.55268000	-4.48903400
C	-0.99238700	0.99234000	-2.99701200
H	-0.17022600	1.36732200	-2.39519900
H	-1.35455900	1.77625500	-3.67171300
C	2.43084500	-1.04034400	-3.26531000
H	2.21475400	-0.07774600	-3.72787800
H	2.39912800	-1.81871800	-4.03783800
C	3.75706900	-0.98848800	-2.54964100
C	4.78913100	-0.21408500	-3.09162700
H	4.62039300	0.34034300	-4.01223000
C	6.02784900	-0.14370900	-2.45658300
H	6.82463500	0.45658200	-2.88547400
C	6.23251800	-0.84141500	-1.26265700
H	7.19046100	-0.78460000	-0.75427100
C	5.20154300	-1.60398500	-0.71771800
H	5.35076400	-2.13015800	0.22179400
C	3.95814100	-1.68959500	-1.35499000
C	2.82858400	-2.43440700	-0.69251700
H	2.50943800	-1.89844000	0.21280400
H	3.15235400	-3.42923800	-0.36444100
C	1.60395900	-2.62473200	-1.58911900
H	1.80398800	-3.34096900	-2.39405700
C	0.40477200	-3.08464400	-0.74941000
C	-0.89528600	-5.07955800	0.06428500
C	-0.75898800	-6.58065200	-0.22578700
H	-0.90363900	-6.79753700	-1.29152000
H	-1.51804500	-7.13491500	0.33316600
H	0.22374800	-6.95798600	0.08233900
C	-0.67375800	-4.80095300	1.56041000
H	0.30453000	-5.17222600	1.88284700
H	-1.44412300	-5.31688200	2.14342000
H	-0.72875000	-3.73187400	1.77461900
C	-2.27329000	-4.56826100	-0.39370000
H	-2.41949700	-4.75985600	-1.46329300
H	-2.37692200	-3.49732000	-0.20551300
H	-3.05939200	-5.09607100	0.15639300
C	-3.30475000	0.08090600	-2.69794800
H	-3.52843500	0.68865300	-3.58495300
H	-2.97174800	-0.90369800	-3.02422600
C	-4.50409700	-0.04926200	-1.78867300
C	-5.50743000	-0.96085000	-2.13613600
H	-5.39109900	-1.57604100	-3.02586900
C	-6.65121800	-1.08844100	-1.35076200
H	-7.42426800	-1.79813900	-1.62961700
C	-6.79364100	-0.30458400	-0.20272000
H	-7.68060000	-0.39863100	0.41681400
C	-5.79293900	0.60027900	0.14710100
H	-5.89701000	1.20869600	1.04238400
C	-4.64397000	0.73837400	-0.64019200
C	-3.53823300	1.67212500	-0.21903400
H	-2.94253800	1.19409300	0.56298400
H	-3.94224300	2.60750400	0.18550800
C	-2.60223300	2.01800800	-1.37491400
H	-3.13484800	2.55162100	-2.17023600
C	-1.41056800	2.82431900	-0.86854500
C	-0.38246900	5.12800100	-0.74533500
C	-0.77350300	6.45309400	-1.41492400
H	-1.77977300	6.77191600	-1.11563300
H	-0.73727100	6.37466500	-2.50846000
H	-0.07451400	7.23801600	-1.11312400
C	-0.46096000	5.25459900	0.78481900
H	-1.46297700	5.56951400	1.09661400
H	0.25676300	6.00788600	1.12656300
H	-0.22006500	4.30582900	1.26819000
C	1.02415900	4.69883700	-1.19592900
H	1.06278300	4.58831100	-2.28583700
H	1.31851900	3.75745000	-0.73146900
H	1.74889800	5.46581300	-0.90453600
O	1.41278000	-1.12851900	1.79832900

C	2.02180300	2.55294900	1.84230300
C	3.06610800	3.47993500	2.03957600
C	4.15775000	3.48096500	1.18328700
C	4.19870100	2.55876200	0.13177200
C	3.12464900	1.68490400	-0.01477100
N	2.05841600	1.67468300	0.79851900
H	4.97106400	4.18546800	1.33514900
H	3.00947000	4.17376400	2.87308000
H	5.03649600	2.50638000	-0.55503000
H	3.09781800	0.95649700	-0.81599700
C	0.89564100	2.49893500	2.74520400
H	0.86832100	3.19096800	3.59057500
N	-0.04791800	1.61129000	2.55418300
C	-1.13611500	1.43212200	3.32311200
H	-1.33994000	2.01754900	4.21655500
C	-1.98444700	0.38108700	2.91637300
O	-1.77912200	-0.33243100	1.88454800
O	-3.07224500	0.16497800	3.67797500
C	-3.92072100	-0.92061900	3.27604300
H	-4.72414600	-0.94633700	4.01348800
H	-4.32628300	-0.75145000	2.27351400
H	-3.36976700	-1.86555400	3.27955300
C	1.97639000	-1.54297000	3.01738300
C	2.71925900	-2.87758700	2.80699300
H	2.04403000	-3.61350800	2.35591500
H	3.57696100	-2.74052400	2.13726100
H	3.09489100	-3.28473500	3.75304900
C	2.97228400	-0.47884200	3.51223800
H	3.72501700	-0.28786500	2.73853700
H	2.44881400	0.46021100	3.71271800
H	3.48405200	-0.79516000	4.42964500
C	0.85416700	-1.73990400	4.05307700
H	1.24525400	-2.08369000	5.01858300
H	0.32116900	-0.79664800	4.20964000
H	0.13357400	-2.47929100	3.68524600

2*R*3*S*-TS-co

Yb	0.08656200	-0.19402800	0.13712800
O	-1.72064500	-1.46184500	1.03984700
O	-0.40206500	0.58816300	2.39978500
O	-1.75462700	1.32767800	0.09812600
O	-0.89978400	-0.38978800	-1.93172700
N	-2.59950200	-1.27863300	2.08588400
N	-1.50720400	1.18275300	4.28864400
H	-2.14653900	0.86885200	5.00663400
N	-2.85062700	1.34660800	-0.76034200
N	-2.48155600	-0.46786800	-3.66068300
H	-4.19995500	-0.03449400	-3.71795900
C	-3.51758400	-0.06815100	1.92401800
H	-2.85025200	0.78562500	1.85179000
H	-4.10894400	-0.02108500	2.84389900
C	-4.42488300	-0.06854300	0.70449800
H	-5.02385000	-0.98332200	0.63841500
H	-5.15507200	0.73325600	0.86567600
C	-3.72028700	0.09852200	-0.63228300
H	-3.04340100	-0.73330100	-0.79830700
H	-4.45437100	0.14612000	-1.44059000
C	-3.42263500	-2.54179800	2.20175700
H	-3.85304300	-2.69104600	1.21218000
H	-4.23273400	-2.34531900	2.91519800
C	-2.59657100	-3.73569100	2.60430900
C	-3.04417700	-5.01647700	2.26157400
H	-3.96354800	-5.13119300	1.69229000
C	-2.31598000	-6.14334800	2.64000400
H	-2.67233900	-7.13351400	2.37231000
C	-1.12643600	-5.99135600	3.35865900
H	-0.55118600	-6.86362300	3.65438000
C	-0.67509500	-4.71533000	3.69139200
H	0.25943400	-4.59153800	4.23256100
C	-1.40525600	-3.57993800	3.32053800
C	-0.83839500	-2.20739100	3.58081800
H	-0.02268800	-2.02720700	2.88243300
H	-0.41225300	-2.14288800	4.58753200
C	-1.83778000	-1.07636700	3.40751200
H	-2.59725800	-1.10198800	4.19604300
C	-1.17094700	0.30955600	3.32952500
C	-1.14159600	2.63318900	4.30812300
C	0.38601400	2.79027000	4.33492400
H	0.82029200	2.24111800	5.17670200
H	0.63490800	3.85053200	4.44894100
H	0.83148700	2.42274500	3.41072400
C	-1.74942400	3.31726100	3.06862600
H	-1.39217100	2.86750300	2.13873500
H	-1.48128600	4.37878200	3.06553600
H	-2.84419100	3.24771600	3.09210800
C	-1.75434700	3.20453800	5.59458600

H	-1.33299200	2.72314700	6.48503800
H	-2.84484600	3.08208900	5.60783100
H	-1.54202300	4.27518900	5.65978100
C	-3.65251600	2.57988200	-0.44351200
H	-4.61168100	2.48050800	-0.96649100
H	-3.83292300	2.56015300	0.63185900
C	-2.95373500	3.85965100	-0.83157400
C	-3.35327400	5.05151600	-0.21650100
H	-4.10829700	5.02451600	0.56666900
C	-2.80140200	6.27112300	-0.60448100
H	-3.12123600	7.19054700	-0.12297000
C	-1.84132900	6.30187900	-1.61904400
H	-1.41323000	7.24779500	-1.93836900
C	-1.43493800	5.11313200	-2.22368100
H	-0.68725800	5.13313400	-3.01335500
C	-1.97573500	3.88191400	-1.83345100
C	-1.47032100	2.59008500	-2.42633300
H	-0.52938200	2.33073800	-1.94630900
H	-1.28142200	2.69522600	-3.50064000
C	-2.43413800	1.43192500	-2.24211900
H	-3.36503100	1.60874000	-2.78153500
C	-1.86892600	0.07145400	-2.65405100
C	-2.11118800	-1.79786600	-4.17885800
C	-3.10770800	-2.10426300	-5.31094300
H	-3.04482400	-1.33884600	-6.09255300
H	-4.13439000	-2.11097700	-4.92636500
H	-2.90196600	-3.08089800	-5.76428600
C	-0.68032700	-1.77692600	-4.74716000
H	-0.60225300	-1.03380700	-5.54892600
H	-0.41082400	-2.75696800	-5.15974000
H	0.03254500	-1.51583900	-3.96382200
C	-2.24847900	-2.87437300	-3.08170400
H	-3.27027700	-2.87189700	-2.68180000
H	-1.55451900	-2.68775500	-2.26171800
H	-2.04913500	-3.87138200	-3.49290900
O	2.05831100	-0.54431100	1.27987700
C	1.30885800	-2.76980200	-1.74271700
C	1.53891700	-4.04175000	-2.28380300
C	0.96130700	-5.15653000	-1.68167300
C	0.16018900	-4.97072900	-0.55593500
C	-0.02939000	-3.67200700	-0.08351000
N	0.52734600	-2.59248800	-0.64671700
H	1.13069600	-6.14961800	-2.08758200
H	2.16863900	-4.14006400	-3.16221100
H	-0.31978700	-5.80044700	-0.04813100
H	-0.66825400	-3.47287400	0.76601900
C	1.91352200	-1.59017800	-2.35268800
H	2.48204700	-1.72747300	-3.27505400
N	1.73529000	-0.42818300	-1.81677600
C	2.29978100	0.72383200	-2.29837500
H	2.64737000	0.80614800	-3.32576000
C	1.82675500	1.89989000	-1.60574500
O	1.17063600	1.82938400	-0.54365000
O	2.23325500	3.06110600	-2.11113700
C	1.93596000	4.25189400	-1.34245900
H	2.23414800	5.08107800	-1.98358900
H	0.87081900	4.30327100	-1.11339100
H	2.51644800	4.24983800	-0.41837900
C	2.71514000	-1.17004200	2.38054100
C	2.21759800	-0.59491100	3.72546900
H	1.14369600	-0.68658700	3.84441300
H	2.70152000	-1.09594600	4.57269900
H	2.45846500	0.47113100	3.78021900
C	2.50251800	-2.69647200	2.29950700
H	1.44502900	-2.95530100	2.21317300
H	2.99189400	-3.09576300	1.40432600
H	2.91668400	-3.21549900	3.17285100
C	4.24747000	-0.92102500	2.39288300
H	4.69501400	-1.43802500	3.24940200
H	4.74683500	-1.29247400	1.49591500
H	4.47802500	0.14352600	2.49583400
C	-5.65723200	0.89685300	-4.66501300
H	-5.74343100	0.19453900	-5.50807200
H	-5.05355000	1.75651300	-4.99917100
H	-6.66128600	1.26103600	-4.42301800
O	-5.12448800	0.27775900	-3.50748700
N	3.96783900	0.97151500	0.01046600
C	4.25757800	0.73403100	-1.28948000
H	4.64737400	1.59009900	-1.83495000
C	4.10656500	2.23629800	0.61540100
C	5.10916600	3.13859100	0.22796700
C	3.23831100	2.57797300	1.66608200
C	5.21299100	4.37905900	0.85871700
H	5.82416900	2.86729600	-0.54253100
C	3.36707700	3.80939500	2.30183800
H	2.45954200	1.87435100	1.93708600
C	4.34675100	4.72268400	1.89825500
H	5.99164100	5.06952100	0.54709900

H	2.68785200	4.06420300	3.10958600
H	4.43958600	5.68443300	2.39367000
C	4.69809100	-0.56905400	-1.78904000
C	4.41127000	-1.77347800	-1.12185500
C	5.38737400	-0.62133200	-3.01611500
C	4.85945000	-2.99132000	-1.63768200
H	3.82410400	-1.74992700	-0.21393300
C	5.81546600	-1.83768300	-3.53714000
H	5.59684900	0.30094300	-3.55328100
C	5.55981600	-3.02810800	-2.84281300
H	4.63760700	-3.91030100	-1.10310300
H	6.35979300	-1.86141800	-4.47659000
H	5.90668400	-3.97564400	-3.24518100
H	3.29215300	0.32020000	0.48897000

2*R*3*S*-TS-dis

Yb	-0.28572600	-0.06172100	0.14309900
O	2.04746600	-0.13110800	-0.36596700
O	2.44666000	-1.97892500	-2.87519400
O	-0.38464300	1.20235900	-1.73794400
O	-0.36916500	2.04473900	0.94377400
N	3.07525100	0.28130100	-1.19202200
N	4.60795200	-2.70779000	-2.92239100
H	5.54084400	-2.54614000	-2.56778600
N	-0.18645800	2.56625600	-1.91460000
N	0.06373100	4.33589300	1.05015900
H	0.69101600	5.51345400	-0.11803900
C	2.54300400	0.79641200	-2.54783700
H	1.65396700	0.20109500	-2.71610600
H	3.29594100	0.53088600	-3.29363000
C	2.26397900	2.28975500	-2.67557800
H	3.19057700	2.86629800	-2.57697700
H	1.95959200	2.43950900	-3.71767800
C	1.26886900	2.93400600	-1.72476000
H	1.47548300	2.65038600	-0.69832500
H	1.33149200	4.02399500	-1.78940500
C	3.87875800	1.35245900	-0.46984200
H	3.15621000	2.08365900	-0.12086800
H	4.52861100	1.82617300	-1.21378400
C	4.70055400	0.85274800	0.69455500
C	5.15824700	1.79241800	1.62653200
H	4.86810000	2.83468400	1.52750200
C	5.98138800	1.39961900	2.67971000
H	6.33476500	2.13735000	3.39364600
C	6.34802000	0.05677100	2.81212600
H	6.99593400	-0.25616400	3.62573800
C	5.87671400	-0.88263100	1.89595300
H	6.14983200	-1.93004000	2.00064100
C	5.05379800	-0.49298100	0.83243400
C	4.50339000	-1.50361600	-0.13967500
H	3.63780800	-2.02151800	0.28367000
H	5.25142900	-2.27453700	-0.35043100
C	4.08892200	-0.85773900	-1.46142200
H	4.95412700	-0.33916500	-1.89203600
C	3.60619800	-1.90198800	-2.49229000
C	4.44865800	-3.86008400	-3.85561200
C	3.49375800	-4.89322600	-3.23522300
H	3.87742200	-5.24424300	-2.27069900
H	3.39647900	-5.75663900	-3.90179100
H	2.50495100	-4.45805300	-3.08126800
C	3.91537500	-3.36266200	-5.20978800
H	2.92773300	-2.91211900	-5.09337100
H	3.83701700	-4.20388300	-5.90656600
H	4.59536400	-2.62091000	-5.64357200
C	5.84888900	-4.46628600	-4.02680300
H	6.25025300	-4.81796400	-3.06814900
H	6.54845900	-3.73964900	-4.45806300
H	5.80132000	-5.32479200	-4.70263800
C	-0.64185600	2.91698800	-3.30809800
H	-0.21319800	3.89883800	-3.54228200
H	-0.19204400	2.16692100	-3.95911300
C	-2.14125500	2.93565400	-3.47626900
C	-2.66281700	2.83857100	-4.77181900
H	-1.98950000	2.68095400	-5.61166300
C	-4.03345100	2.94915600	-4.99523500
H	-4.42690200	2.87418400	-6.00459200
C	-4.89426400	3.16092400	-3.91485100
H	-5.96349400	3.26028300	-4.07874600
C	-4.37562600	3.24598500	-2.62436200
H	-5.04177300	3.40877900	-1.78028900
C	-3.00044900	3.12705600	-2.38874900
C	-2.45565100	3.14082100	-0.98358600
H	-2.62622200	2.16826200	-0.52622300
H	-2.97074200	3.88929500	-0.37009600
C	-0.97232700	3.45269200	-0.91127400
H	-0.76887500	4.47067400	-1.24579200

C	-0.38463600	3.25884900	0.49157900
C	0.70145400	4.29107800	2.38209100
C	1.09652000	5.74153500	2.70966800
H	0.20814600	6.38217500	2.73505200
H	1.77514700	6.13613100	1.94422100
H	1.59663600	5.80453500	3.68304100
C	-0.26839200	3.76592800	3.45775300
H	-1.17019000	4.38792900	3.48668900
H	0.20297300	3.80151400	4.44744100
H	-0.56297700	2.73696100	3.24436400
C	1.97579400	3.42047600	2.32880700
H	2.65320200	3.81017700	1.55757800
H	1.73351700	2.38076200	2.09692200
H	2.50663500	3.44686700	3.28776100
O	-0.69503800	-2.17985500	-0.10669700
C	0.08101000	-0.03316700	3.57687000
C	0.64700200	0.06718200	4.85281400
C	2.02262900	-0.09311000	5.00214500
C	2.79165700	-0.36080200	3.87193600
C	2.14896200	-0.44036100	2.63477100
N	0.83239700	-0.27496400	2.47262100
H	2.48183900	-0.01407600	5.98323100
H	0.00723500	0.26316600	5.70722800
H	3.86561700	-0.49315100	3.92560300
H	2.69386300	-0.61949800	1.72186200
C	-1.35741200	0.07116900	3.39263100
H	-1.98250300	0.19313100	4.28041900
N	-1.85865900	-0.02567100	2.20785600
C	-3.21794800	-0.09322900	1.99357900
H	-3.91289900	0.24984900	2.75908700
C	-3.55962300	0.12032000	0.60009700
O	-2.70890400	0.02644300	-0.30280700
O	-4.85468300	0.31657900	0.38178400
C	-5.34421600	0.35457600	-0.98017800
H	-6.12070800	1.12004800	-0.99557900
H	-4.54281100	0.60243900	-1.67456400
H	-5.76476800	-0.62370900	-1.21693700
C	-0.13553000	-3.39186200	-0.62091600
C	1.31874800	-3.48425200	-0.13643400
H	1.85093500	-2.59625400	-0.47485400
H	1.34368500	-3.50978100	0.96053500
H	1.82873600	-4.37579600	-0.52011400
C	-0.91172200	-4.62806600	-0.12559500
H	-0.92318100	-4.69101900	0.96706000
H	-1.94669600	-4.62215300	-0.48127700
H	-0.43869500	-5.54060200	-0.50634400
C	-0.20252100	-3.35886700	-2.15691500
H	0.31931900	-4.21625800	-2.59771900
H	-1.24278500	-3.39870800	-2.49176300
H	0.25959900	-2.44735200	-2.53597800
C	0.49373400	7.27126000	-0.99006000
H	0.89409200	7.76912600	-1.87923500
H	0.75508700	7.88031400	-0.11155000
H	-0.60494800	7.25096000	-1.07540800
O	1.05715500	5.97210300	-0.92432900
N	-3.29554800	-2.66724600	0.78218100
C	-3.63376500	-2.18049400	2.00689700
C	-4.21828800	-2.83867100	-0.27166500
C	-5.53161500	-3.27473400	-0.03957800
C	-3.79098200	-2.60680200	-1.58885900
C	-6.40565600	-3.45423000	-1.11280600
H	-5.86308100	-3.50309000	0.96897900
C	-4.66313700	-2.81638300	-2.65389000
H	-2.79032600	-2.22646800	-1.74962400
C	-5.97756600	-3.23430400	-2.42463000
H	-7.42022800	-3.79161100	-0.92028400
H	-4.31731600	-2.63250900	-3.66735300
H	-6.65832200	-3.39076500	-3.25594300
C	-2.87716900	-2.54705700	3.21551500
C	-1.53032600	-2.94741800	3.16720000
C	-3.50787100	-2.43277900	4.46597000
C	-0.84907800	-3.26554400	4.34094900
H	-1.01300300	-2.97386700	2.21503900
C	-2.82136700	-2.74327300	5.63868100
H	-4.54487200	-2.10802200	4.51672000
C	-1.48977300	-3.16697400	5.57816800
H	0.19094600	-3.57469500	4.28747900
H	-3.32672700	-2.66527900	6.59695000
H	-0.95664400	-3.41670400	6.49111200
H	-2.30097000	-2.56196500	0.48668700
H	-4.70366100	-2.06018400	2.15876800

2*R*3*R*-TS-co

Yb	-0.08156200	-0.10808000	-0.14781100
O	0.63772300	-2.21123900	-0.99965500
O	0.72775200	0.19895400	-2.36933700

O	2.30733700	0.06539900	-0.11471000
O	0.62652200	-0.68833600	1.99019400
N	1.49619100	-2.58561700	-2.01344200
N	2.06831500	0.12025700	-4.18570300
H	2.49912400	-0.47703200	-4.87941700
N	3.20827200	-0.49665200	0.78324900
N	1.87580400	-1.46549700	3.81935500
H	3.51687700	-2.13496800	3.89863900
C	2.94793200	-2.16080400	-1.79859200
H	2.92281800	-1.07498300	-1.76746300
H	3.48838300	-2.51095300	-2.68353700
C	3.62803900	-2.66288400	-0.53324100
H	3.52013300	-3.74595500	-0.41165300
H	4.70254900	-2.50332600	-0.68248000
C	3.16513500	-2.02065800	0.76539400
H	2.12610400	-2.27131600	0.95381100
H	3.78503200	-2.36421600	1.59790700
C	1.41427500	-4.09193700	-2.12572900
H	1.63431500	-4.46251700	-1.12524300
H	2.21096900	-4.41609200	-2.80693900
C	0.06150800	-4.56686100	-2.58402800
C	-0.36142000	-5.85407500	-2.23391800
H	0.27070500	-6.47582300	-1.60418700
C	-1.58599900	-6.34128500	-2.68773400
H	-1.90644200	-7.34196600	-2.41385300
C	-2.39725500	-5.53550800	-3.49133700
H	-3.35385000	-5.90602100	-3.84750200
C	-1.98114000	-4.24926800	-3.82991000
H	-2.62009400	-3.61215000	-4.43549900
C	-0.75070000	-3.75491300	-3.38152300
C	-0.37633200	-2.31971200	-3.65000800
H	-0.98284500	-1.68376100	-3.01053200
H	-0.60298700	-2.03353300	-4.68309500
C	1.07528200	-1.97375900	-3.36314600
H	1.74352000	-2.41696400	-4.10861400
C	1.28154800	-0.45090400	-3.26753700
C	2.49454500	1.55780800	-4.17796300
C	1.26360600	2.48020700	-4.19139200
H	0.60351100	2.23474500	-5.03032200
H	1.59901600	3.51609100	-4.30856600
H	0.69837600	2.40031400	-3.26264000
C	3.36075000	1.80820300	-2.92960600
H	2.82154000	1.57587900	-2.00803000
H	3.66738200	2.85888900	-2.89688500
H	4.27058200	1.19618700	-2.96369100
C	3.31593100	1.75692100	-5.45937900
H	2.70785700	1.58093300	-6.35451300
H	4.18721800	1.08985100	-5.48598300
H	3.68785100	2.78433200	-5.50207300
C	4.58172800	0.00304300	0.42432600
H	5.29885700	-0.57959200	1.01555600
H	4.73058600	-0.22391200	-0.63173100
C	4.74641300	1.48371400	0.66458500
C	5.74708700	2.16924200	-0.03325100
H	6.34738100	1.63956300	-0.77023400
C	5.98833500	3.51974400	0.21337100
H	6.77042600	4.04059000	-0.33122300
C	5.22337700	4.19368300	1.16871900
H	5.41007500	5.24320500	1.37776200
C	4.21897700	3.51345700	1.85621700
H	3.61730000	4.03530700	2.59698500
C	3.96285700	2.15952900	1.60827000
C	2.81920600	1.45176400	2.29190600
H	1.88702700	1.74598100	1.81324900
H	2.75542600	1.73507500	3.34859300
C	2.93186800	-0.06164800	2.23401000
H	3.79605700	-0.41100000	2.79903100
C	1.68427800	-0.79430400	2.72677200
C	0.80525800	-2.25642800	4.45264900
C	1.44812800	-2.94567800	5.66953400
H	1.85983000	-2.19984400	6.35858100
H	2.26749800	-3.60050500	5.35004300
H	0.71383100	-3.55214200	6.21235300
C	-0.33344000	-1.33586900	4.92990200
H	0.04888100	-0.60354300	5.65006000
H	-1.12575700	-1.91670100	5.41788500
H	-0.76202300	-0.79532400	4.08474200
C	0.27345600	-3.33536100	3.48750800
H	1.09679200	-3.98225000	3.15986200
H	-0.17934400	-2.87912400	2.60753100
H	-0.47448900	-3.96454800	3.98479100
O	-2.09262300	0.47834600	-1.18609400
C	-2.46251800	-1.20984700	1.94878200
C	-3.36421400	-2.02376300	2.64419900
C	-3.55290800	-3.34140900	2.23120800
C	-2.83447800	-3.81136700	1.13343100
C	-1.94628300	-2.94234500	0.49656500
N	-1.76086400	-1.67647500	0.88564500

H	-4.24921000	-3.98682800	2.75833100
H	-3.91438900	-1.61580800	3.48545500
H	-2.94571400	-4.82808200	0.77174000
H	-1.34440900	-3.26318700	-0.34732800
C	-2.27558000	0.18926100	2.34093300
H	-2.85812200	0.55505400	3.19066700
N	-1.45174600	0.93936400	1.69654800
C	-1.26473900	2.28238800	1.94083500
H	-1.45712900	2.70095000	2.92723900
C	-0.17848900	2.82333000	1.15371700
O	0.30988400	2.22004200	0.17208100
O	0.21021200	4.04729300	1.49070700
C	1.12399200	4.70685200	0.58264800
H	1.32866500	5.67325900	1.04236900
H	2.04109800	4.12564000	0.47755500
H	0.64523600	4.83634600	-0.38925600
C	-2.92883900	0.23312400	-2.31436800
C	-2.19867600	0.61065600	-3.62469800
H	-1.21187600	0.16444800	-3.68620000
H	-2.77793500	0.31595100	-4.50843600
H	-2.05611600	1.69620700	-3.66457100
C	-3.38755400	-1.24213000	-2.29727800
H	-2.55481900	-1.92555700	-2.12762100
H	-4.08653400	-1.40525200	-1.47008800
H	-3.89600700	-1.53067700	-3.22591100
C	-4.22173000	1.09439100	-2.31755300
H	-4.77419600	0.91724200	-3.24774500
H	-4.89751900	0.84589300	-1.49651300
H	-3.99603400	2.16342000	-2.26717000
N	-3.67744700	2.05036400	0.55443200
C	-2.97483400	3.15275100	0.90923200
H	-3.35856500	3.66448800	1.78625200
C	-4.80992000	1.56358100	1.23814600
C	-5.57863300	2.37008800	2.09315000
C	-5.21114500	0.23364300	1.02024500
C	-6.69779900	1.84092600	2.73729400
H	-5.33407100	3.41692000	2.23884000
C	-6.33778900	-0.27850800	1.65647300
H	-4.61535300	-0.39808700	0.37251800
C	-7.08678000	0.51708800	2.52759300
H	-7.27850200	2.48076400	3.39578700
H	-6.62345900	-1.31096500	1.47451600
H	-7.96450600	0.11625200	3.02497600
C	-2.39107900	4.06774600	-0.09430300
C	-1.88706600	3.62849600	-1.33090500
C	-2.36200700	5.43986400	0.20834400
C	-1.41506600	4.55497500	-2.25842700
H	-1.84238000	2.56339500	-1.53211600
C	-1.88477100	6.36184500	-0.72172300
H	-2.72930500	5.78720200	1.17094200
C	-1.41866500	5.92217900	-1.96386100
H	-1.03866300	4.20510100	-3.21576900
H	-1.88401900	7.42079700	-0.48029900
H	-1.05555000	6.64035700	-2.69382200
H	-3.20229900	1.41694300	-0.12911600
C	5.26617700	-2.20135600	4.80807000
H	4.92529700	-2.73869200	5.70607600
H	5.32343800	-1.12864400	5.05540200
H	6.27682000	-2.54859000	4.56854100
O	4.43803900	-2.45611400	3.68728500

2*R*3*R*-TS-dis

Yb	0.33120000	-0.10602300	0.02027300
O	-1.95995600	0.34528200	0.35830600
O	-2.50535500	-0.28222100	3.35693700
O	0.58436200	1.78925900	1.26675100
O	0.75263100	1.50056400	-1.52445800
N	-2.93175000	1.15939400	0.90602800
N	-4.72486300	-0.72942600	3.63229100
H	-5.64421800	-0.62476700	3.22472900
N	0.58083400	3.12438800	0.88417100
N	0.67370800	3.61110500	-2.52745300
H	0.21969000	5.22459000	-1.95425300
C	-2.35566700	2.12623200	1.96390800
H	-1.54697600	1.56494600	2.41459500
H	-3.14655800	2.27308600	2.70365700
C	-1.89510400	3.49698800	1.48611400
H	-2.73421000	4.07592100	1.08524800
H	-1.60004400	4.03709700	2.39275500
C	-0.79787900	3.56306500	0.43849000
H	-1.02105600	2.90789300	-0.39777200
H	-0.69892600	4.58284000	0.05580100
C	-3.60892800	1.92657600	-0.21889700
H	-2.80862700	2.39191600	-0.78755700
H	-4.21556300	2.71212800	0.24520800
C	-4.45922500	1.07959200	-1.13309700

C	-4.78009600	1.59222800	-2.39614700
H	-4.36945900	2.54868600	-2.70884400
C	-5.61679200	0.88141900	-3.25392600
H	-5.86443500	1.28922800	-4.22924700
C	-6.13042400	-0.35677900	-2.85522600
H	-6.78788500	-0.91461500	-3.51577500
C	-5.79407300	-0.87680200	-1.60608900
H	-6.18062200	-1.84531000	-1.29771900
C	-4.96141900	-0.16317000	-0.73560900
C	-4.54364300	-0.74370200	0.59043100
H	-3.74789800	-1.47832600	0.44736000
H	-5.37939500	-1.27727600	1.05505100
C	-4.05397500	0.32718200	1.56568000
H	-4.85811800	1.05460500	1.73132900
C	-3.65801900	-0.25862900	2.94305100
C	-4.67040100	-1.36228200	4.98162900
C	-3.79151100	-2.62279400	4.92864300
H	-4.18154600	-3.33411400	4.19248200
H	-3.78708100	-3.11010500	5.90934900
H	-2.76483500	-2.36832200	4.65996200
C	-4.11952900	-0.35237200	6.00290600
H	-3.09966300	-0.06065100	5.74341000
H	-4.11072900	-0.80223200	7.00130900
H	-4.74820700	0.54460100	6.03742500
C	-6.11658800	-1.73971200	5.33335800
H	-6.52934300	-2.45203600	4.60843400
H	-6.76376200	-0.85432300	5.36729900
H	-6.14749100	-2.21173400	6.31947500
C	1.03498800	3.94500700	2.06488300
H	0.71656000	4.97666600	1.87259100
H	0.48176700	3.55402000	2.91940700
C	2.52136600	3.89576300	2.32290600
C	2.98599000	4.32759000	3.57097800
H	2.27227900	4.60977900	4.34208100
C	4.35229300	4.40723700	3.83105200
H	4.70113200	4.74531500	4.80227300
C	5.26696200	4.05583200	2.83478800
H	6.33447700	4.12643900	3.02263700
C	4.80456000	3.61585800	1.59588300
H	5.51266500	3.33830400	0.81835200
C	3.43279900	3.52199600	1.32892500
C	2.93241700	2.97380600	0.01745200
H	2.94374800	1.88566100	0.05431400
H	3.57671800	3.28627900	-0.81223200
C	1.52252500	3.42865500	-0.30963400
H	1.47781100	4.51342400	-0.41355800
C	0.94336400	2.78126800	-1.57144200
C	0.05691900	3.15573600	-3.78939100
C	-0.12361400	4.41698900	-4.65218100
H	0.84611800	4.88863100	-4.84598800
H	-0.75692700	5.14774900	-4.13542900
H	-0.58831100	4.17310200	-5.61454500
C	0.96573500	2.15449200	-4.52653600
H	1.94136800	2.61093900	-4.72794100
H	0.51966400	1.86300200	-5.48522700
H	1.11983800	1.25892700	-3.92287500
C	-1.32864000	2.53328100	-3.51086600
H	-1.96022600	3.26184000	-2.98496000
H	-1.23790100	1.63481700	-2.89786800
H	-1.83156000	2.26639600	-4.44798000
O	0.29950800	-2.13351100	0.84380300
C	0.13226100	-1.22731800	-3.22172600
C	-0.35525000	-1.51754900	-4.49886100
C	-1.73228400	-1.56528600	-4.70629700
C	-2.58063900	-1.32969400	-3.62747300
C	-2.01219900	-1.03429800	-2.38620700
N	-0.69445500	-0.97215900	-2.17715100
H	-2.13157600	-1.79210500	-5.69034700
H	0.34115400	-1.71907400	-5.30633500
H	-3.65939200	-1.35567200	-3.72790600
H	-2.62045000	-0.81820200	-1.52096600
C	1.56363000	-1.25223700	-2.94474500
H	2.24022500	-1.51335000	-3.76382100
N	1.98332500	-1.02535300	-1.75132500
C	3.30321700	-1.18190900	-1.36772700
H	4.09652500	-1.19176200	-2.11440000
C	3.56834100	-0.53583600	-0.10088800
O	2.65850500	-0.19825600	0.68642200
O	4.85583200	-0.38671100	0.18691700
C	5.19188000	0.08477300	1.51218100
H	6.26658100	0.26243200	1.48381000
H	4.65292400	1.00340200	1.74376800
H	4.94996500	-0.68569800	2.24500100
C	-0.32954100	-2.94049700	1.84145800
C	-1.84192800	-2.93195400	1.58359600
H	-2.17144000	-1.89689600	1.55808800
H	-2.06328100	-3.39631000	0.61466700
H	-2.39941800	-3.46851500	2.35959800

C	0.16239400	-4.40202100	1.78808600
H	-0.04638800	-4.85822000	0.81496900
H	1.23523600	-4.48618500	1.98084400
H	-0.35639400	-4.99590800	2.54934500
C	-0.01532200	-2.34436000	3.22477900
H	-0.48260800	-2.92370700	4.03048800
H	1.06646200	-2.33683000	3.39690100
H	-0.38370800	-1.31754500	3.28718200
C	0.70565500	7.12733100	-1.77155400
H	0.35575900	7.98702200	-1.19076000
H	0.61943400	7.37929000	-2.83923300
H	1.77297900	6.97275600	-1.54317900
O	-0.09173300	6.00876500	-1.42212000
N	1.97232700	-3.62400000	-0.81528300
C	3.28071300	-3.26996900	-0.79414200
H	3.84721200	-3.58154600	-1.66628900
C	1.28980300	-4.19311700	-1.90858400
C	1.94335600	-4.84710300	-2.96476600
C	-0.11510600	-4.13333900	-1.90492100
C	1.19965000	-5.39990900	-4.00810900
H	3.02371300	-4.95011200	-2.97285600
C	-0.84450600	-4.69967500	-2.94588400
H	-0.61133500	-3.60491000	-1.09972000
C	-0.19508500	-5.33135100	-4.00969400
H	1.72025000	-5.90379500	-4.81787000
H	-1.92880600	-4.63249200	-2.92966700
H	-0.76535800	-5.77157300	-4.82201600
C	4.04950000	-3.30001600	0.46512100
C	3.45807900	-3.02786100	1.71008100
C	5.41552900	-3.61943500	0.41176500
C	4.20839900	-3.13170300	2.87879100
H	2.42630300	-2.70465400	1.74821900
C	6.16652100	-3.71196000	1.58264100
H	5.88784300	-3.80971000	-0.54898900
C	5.56209900	-3.47967900	2.82095200
H	3.73564700	-2.93104800	3.83632600
H	7.21916400	-3.97382000	1.52904100
H	6.14371700	-3.56322100	3.73457900
H	1.35723700	-3.17593800	-0.10322200

(L) References

1 Y. H. Wen, J. L. Huang, Y. Xiong, B. Qin and X. M. Feng, *Synlett*, **2005**, 2445.

2 (a) A. Yanagisawa, H. Saito, M. Harada and T. Arai, *Adv. Synth. Catal.*, 2005, **347**, 1517; (b) J. L. G. Ruano, J. Alemán, I. Alonso, A. Parra, V. Marcos and J. Aguirre, *Chem. -Eur. J.*, 2007, **13**, 6179; (c) S. Hayashi, H. Yorimitsu and K. Oshima, *Angew. Chem., Int. Ed.*, 2009, **48**, 7224; (d) P. Eisenberger, A. M. Bailey and C. M. Crudden, *J. Am. Chem. Soc.*, 2012, **134**, 17384; (e) A. Yangisawa, Y. Lin, R. Miyake and K. Yoshida, *Org. Lett.*, 2014, **16**, 86.

3 J. Chen, X. Gong, J. Li, Y. Li, J. Ma, C. Hou, G. Zhao, W. Yuan and B. Zhao, *Science*, 2018, **360**, 1438.

4 M. J. Frisch, G. W. Trucks, H. B. Schlegel, G. E. Scuseria, M. A. Robb, J. R. Cheeseman, G. Scalmani, V. Barone, G. A. Petersson, H. Nakatsuji, X. Li, M. Caricato, A. V. Marenich, J. Bloino, B. G.Janesko, R. Gomperts, B. Mennucci, H. P. Hratchian, J. V. Ortiz, A. F. Izmaylov, J. L. Sonnenberg, Williams, F. Ding, F. Lipparini, F. Egidi, J. Goings, B. Peng, A. Petrone, T. Henderson, D. Ranasinghe, V. G. Zakrzewski, J. Gao, N. Rega, G. Zheng, W. Liang, M. Hada, M. Ehara, K. Toyota, R. Fukuda, J. Hasegawa, M. Ishida, T. Nakajima, Y. Honda, O. Kitao, H. Nakai, T. Vreven, K. Throssell, J. A. Montgomery Jr., J. E. Peralta, F. Ogliaro, M. J. Bearpark, J. J. Heyd, E. N. Brothers, K. N. Kudin, V. N. Staroverov, T. A. Keith, R. Kobayashi, J. Normand, K. Raghavachari, A. P. Rendell, J. C. Burant, S. S. Iyengar, J. Tomasi, M. Cossi, J. M. Millam, M. Klene, C. Adamo, R. Cammi, J. W. Ochterski, R. L. Martin, K. Morokuma, O. Farkas, J. B. Foresman and D. J. Fox, 2013, Gaussian 09, Rev. D.01; Gaussian, Inc.; Wallingford CT.

5 P. J. Stephens, F. J. Devlin, C. F. Chabalowski and M. J. Frisch, *J. Phys. Chem.*, 1994, **98**, 11623.

6 S. Grimme, S. A. J. Fau-Ehrlich, H. E. S. Fau-Krieg and H. Krieg, *J. Chem. Phys.*, 2010, **132**, 154104.

7(a) M. Dolg, H. Stoll and A. Savin, *Theor. Chim. Acta.*, 1989, **75**, 173; (b) M. Dolg, H. Stoll and H. Preuss, *Theor. Chim. Acta.*, 1993, **85**, 441.

8 (a) R. Ditchfield, W. J. Hehre and J. A. Pople, *J. Chem. Phys.*, 1971, **54**, 724; (b) W. J. Hehre, R. Ditchfield and J. A. Pople, *J. Chem. Phys.*, 1972, **56**, 2257; (c) P. C. Hariharan and J. A. Pople, *Theor. Chim. Acta.*, 1973, **28**, 213.

9 A. V. Marenich, C. J. Cramer and D. G. Truhlar, *J. Phys. Chem. B.*, 2009, **113**, 6378.

10. CYLview, 1.0b; C. Y. Legault, Université de Sherbrooke, 2009. (http://www.cylview.org)

(M) The Analytical and Spectral Characterization Data of the Products

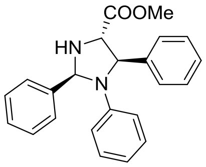

3a

Methyl (2*S*,4*S*,5*R*)-1,2,5-triphenylimidazolidine-4-carboxylate (3a): Procedure A: 62% yield, Colorless oil; R_f = 0.4 (petroleum ether/ethyl acetate = 7/1); Dissolved in iPrOH for HPLC; **HPLC** (Chiralcel IA, hexane/iPrOH = 90/10, flow rate 1.0 mL/min, λ = 254 nm) major isomer: t_r (major) = 10.26 min, t_r (minor) = 8.27 min, ee = 97%; minor isomer: t_r (major) = 7.71 min. dr = 94:6 determined by ^{1}H NMR. $[\alpha]^{24}_D$ = +26.1 (*c* = 0.82, in tetrahydrofuran). **^{1}H NMR (400 MHz, CDCl$_3$)** δ 7.73 – 7.69 (m, 2H), 7.58 – 7.55 (m, 2H), 7.44 – 7.33 (m, 6H), 7.13 – 7.07 (m, 2H), 6.76 – 6.72 (m, 1H), 6.55 – 6.52 (m, 2H), 5.73 (s, 1H), 4.92 (d, *J* = 4.8 Hz, 1H), 3.96 (d, *J* = 4.4 Hz, 1H), 3.79 (s, 3H), 2.98 (s, 1H) ppm; **^{13}C{^{1}H} NMR (101 MHz, CDCl$_3$)** δ 172.2, 147.0, 142.1, 140.9, 128.9, 128.8, 128.7, 128.3, 127.7, 127.1, 126.3, 118.4, 114.5, 79.9, 69.8, 67.7, 52.5 ppm; **IR (neat) ν (cm^{-1}):** 3028, 1739, 1599, 1502, 1451, 1331, 1210, 1135, 1028, 751, 669**; HRMS** (ESI-FT) calcd for $C_{23}H_{23}N_2O_2^+$ ([M]+H$^+$) **=** 359.1754, found 359.1754.

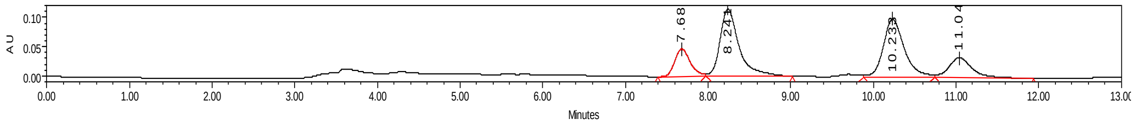

	Retention Time	Area	% Area
1	7.682	627557	12.70
2	8.241	1837118	37.18
3	10.233	1786501	36.15
4	11.041	690451	13.97

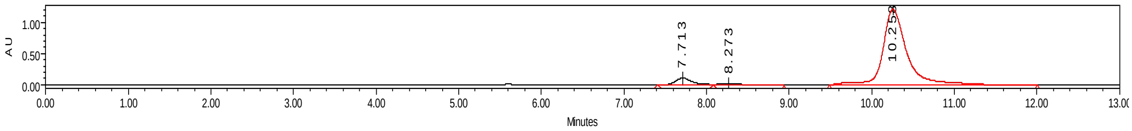

	Retention Time	Area	% Area
1	7.713	1468086	5.70
2	8.273	321664	1.25
3	10.258	23979606	93.05

Procedure B: 55% yield, Colorless oil; R_f = 0.4 (petroleum ether/ethyl acetate = 7/1); Dissolved in iPrOH for HPLC; **HPLC** (Chiralcel IA, hexane/iPrOH = 90/10, flow rate 1.0 mL/min, λ = 254 nm) major isomer: t_r (major) = 10.22 min, t_r (minor) = 8.23 min, ee = 96%; minor isomer: t_r (major) = 7.68 min. dr = 93:7 determined by ^{1}H NMR. $[\alpha]^{24}_D$ = +25.0 (*c* = 0.24, in tetrahydrofuran).

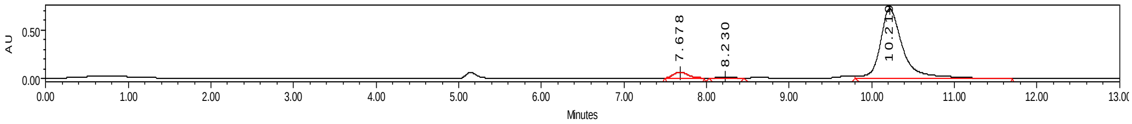

	Retention Time	Area	% Area
1	7.678	740070	5.05
2	8.230	251502	1.72
3	10.219	13662953	93.23

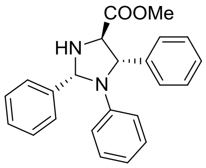

(2*R*,4*R*,5*S*)-**3a**

Methyl (2*R*,4*R*,5*S*)-1,2,5-triphenylimidazolidine-4-carboxylate [(2*R*,4*R*,5*S*)-3a]: Procedure A: 50% yield, Colorless oil; R_f = 0.4 (petroleum ether/ethyl acetate = 7/1); Dissolved in iPrOH for HPLC; **HPLC** (Chiralcel IA, hexane/iPrOH = 90/10, flow rate 1.0 mL/min, λ = 254 nm) major isomer: t_r (major) = 8.26 min, t_r (minor) = 10.27 min, ee = –95%; minor isomer: t_r (major) = 11.08 min, t_r (minor) = 7.71. dr = 90:10 determined by ^{1}H NMR. $[\alpha]^{27}_D$ = –36.8 (*c* = 0.32, in tetrahydrofuran). **^{1}H NMR (400 MHz, CDCl$_3$)** δ 7.73 – 7.70 (m, 2H), 7.58 – 7.55 (m, 2H), 7.45 – 7.39 (m, 4H), 7.35 – 7.31 (m, 2H), 7.12 – 7.08 (m, 2H), 6.77 – 6.73 (m, 1H), 6.56 – 6.53 (m, 2H), 5.73 (s, 1H), 4.93 (d, *J* = 4.8 Hz, 1H), 3.97 (d, *J* = 4.4 Hz, 1H), 3.79 (s, 3H), 2.99 (s, 1H) ppm; **^{13}C{^{1}H} NMR (101 MHz, CDCl$_3$)** δ 172.2, 147.0, 142.1, 140.9, 128.90, 128.85, 128.79, 128.7, 128.5, 128.4, 127.7, 127.1,

126.9, 126.3, 118.4, 115.0, 114.5, 79.9, 69.8, 67.7, 52.5 ppm.

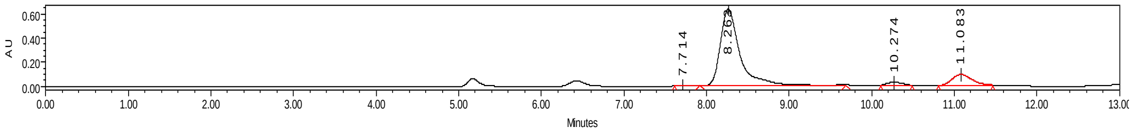

	Retention Time	Area	% Area
1	7.714	48196	0.38
2	8.263	10661227	85.02
3	10.274	315843	2.52
4	11.083	1514176	12.08

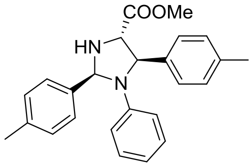

3b

Methyl (2*S*,4*S*,5*R*)-1-phenyl-2,5-di-*p*-tolylimidazolidine-4-carboxylate (3b): Procedure A: 46% yield, Colorless oil; R_f = 0.4 (petroleum ether/ethyl acetate = 7/1); Dissolved in iPrOH for HPLC; **HPLC** (Chiralcel IA, hexane/iPrOH = 90/10, flow rate 1.0 mL/min, λ = 254 nm) major isomer: t_r (major) = 8.93 min, t_r (minor) = 6.83 min, ee = 93%; minor isomer: t_r (major) = 6.11 min. dr = 93:7 determined by ^{1}H NMR. $[\alpha]^{28}_D$ = +45.2 (*c* = 0.25, in tetrahydrofuran). **^{1}H NMR (400 MHz, CDCl$_3$)** δ 7.60 – 7.57 (m, 2H), 7.46 – 7.43 (m, 2H), 7.24 – 7.19 (m, 4H), 7.11 – 7.07 (m, 2H), 6.75 – 6.71 (m, 1H), 6.55 – 6.49 (m, 2H), 5.68 (s, 1H), 4.88 (d, *J* = 4.4 Hz, 1H), 3.94 (d, *J* = 4.6 Hz, 1H), 3.78 (s, 3H), 2.38 (s, 3H), 2.37 (s, 3H) ppm; **^{13}C{^{1}H} NMR (101 MHz, CDCl$_3$)** δ 172.3, 147.0, 139.1, 138.0, 137.9, 137.3, 129.5, 129.4, 128.8, 127.0, 126.2, 118.1, 114.4, 79.7, 69.5, 67.7, 52.5, 21.2, 21.1 ppm; **IR (neat) ν (cm^{-1}):** 2953, 1740, 1600, 1502, 1445, 1326, 1207, 1177, 1135, 815, 752, 694; **HRMS** (ESI-FT) calcd for $C_{25}H_{27}N_2O_2^+$ ([M]+H$^+$) = 387.2067, found 387.2060.

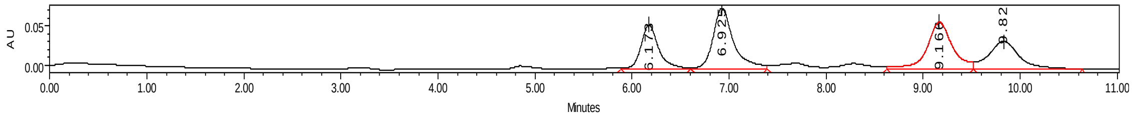

	Retention Time	Area	% Area
1	6.173	696038	19.08
2	6.925	1131237	31.00
3	9.166	1068414	29.28
4	9.829	753188	20.64

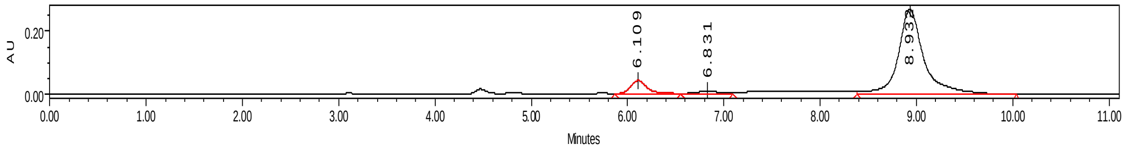

	Retention Time	Area	% Area
1	6.109	552803	9.59
2	6.831	179704	3.12
3	8.932	5030252	87.29

Procedure B: 40% yield, Colorless oil; R_f = 0.4 (petroleum ether/ethyl acetate = 7/1); Dissolved in iPrOH for HPLC; **HPLC** (Chiralcel IA, hexane/iPrOH = 90/10, flow rate 1.0 mL/min, λ = 254 nm) major isomer: t_r (major) = 9.19 min, t_r (minor) = 6.94 min, ee = 88%; minor isomer: t_r (major) = 6.18 min. dr = 88:12 determined by ^{1}H NMR. $[\alpha]^{28}_D$ = +39.6 (*c* = 0.23, in tetrahydrofuran).

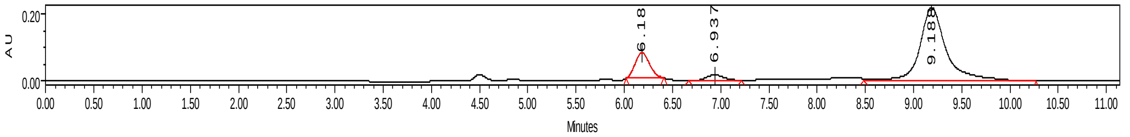

	Retention Time	Area	% Area
1	6.183	795097	14.99
2	6.937	267500	5.04
3	9.188	4241378	79.97

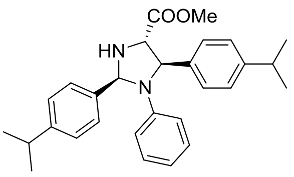

3c

Methyl (2*S*,4*S*,5*R*)-2,5-bis(4-isopropylphenyl)-1-phenylimidazolidine-4-carboxylate (3c): Procedure A: 38% yield, Colorless oil; R_f = 0.4 (petroleum ether/ethyl acetate = 8/1); Dissolved in iPrOH for HPLC; **HPLC** (Chiralcel IF, hexane/iPrOH = 98/2, flow rate 1.0 mL/min, λ = 254 nm) major isomer: t_r (major) = 12.83 min, t_r (minor) = 11.87 min, ee = 93%; minor isomer: t_r (major) = 17.41 min, t_r (minor) = 11.54 min. dr = 88:12 determined by ^{1}H NMR. $[\alpha]^{29}_D$ = +20.1 (*c* = 0.30, in tetrahydrofuran). **^{1}H NMR (400 MHz, CDCl$_3$)** δ 7.63 – 7.59 (m, 2H), 7.52 – 7.42 (m, 2H), 7.28 – 7.24 (m, 4H), 7.13 – 7.09 (m, 2H), 6.76 – 6.72 (m, 1H), 6.56 – 6.51 (m, 2H), 5.69 (s, 1H), 4.91 (d, *J* = 4.0 Hz, 1H), 3.97 (d, *J* = 4.4 Hz, 1H), 3.79 (s, 3H), 2.99 – 2.85 (m, 3H), 1.29 – 1.26 (m, 12H) ppm; **^{13}C{^{1}H} NMR (101 MHz, CDCl$_3$)** δ 172.4, 148.9, 148.2, 147.0, 139.5, 138.1, 128.8, 127.0, 126.9, 126.7, 126.2, 118.1, 114.5, 79.7, 69.5, 67.6, 52.5, 33.8, 33.7, 23.99, 23.97 ppm**; IR (neat) ν (cm^{-1}):** 2959. 1741, 1599, 1502, 1461, 1329, 1211, 1132, 828, 751, 693; **HRMS** (ESI-FT) calcd for $C_{29}H_{35}N_2O_2^+$ ([M]+H$^+$) = 443.2693, found 443.2686.

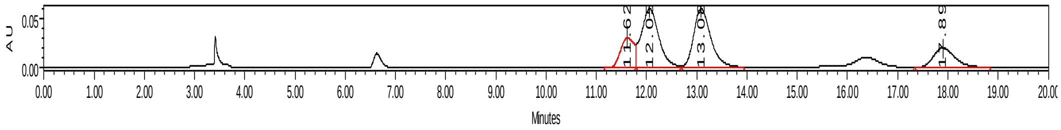

	Retention Time	Area	% Area
1	11.620	562681	13.99
2	12.059	1454207	36.15
3	13.086	1399731	34.79
4	17.898	606276	15.07

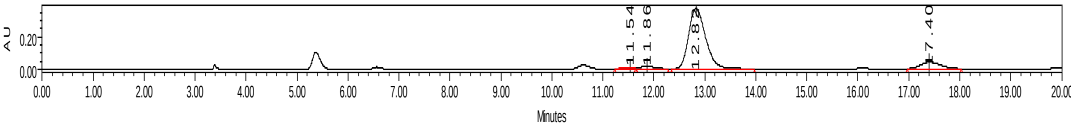

	Retention Time	Area	% Area
1	11.542	157892	1.54
2	11.869	318090	3.10
3	12.826	8660043	84.38
4	17.406	1127486	10.99

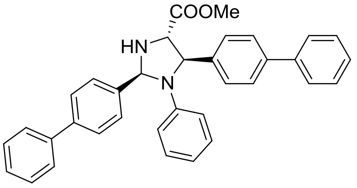

3d

Methyl (2*S*,4*S*,5*R*)-2,5-di[(1,1'-biphenyl)-4-yl]-1-phenylimidazolidine-4-carboxylate (3d): Procedure A: 50% yield, Colorless oil; R_f = 0.4 (petroleum ether/ethyl acetate = 6/1); Dissolved in iPrOH for HPLC; **HPLC** (Chiralcel IA, hexane/iPrOH = 90/10, flow rate 1.0 mL/min, λ = 254 nm) major isomer: t_r (major) = 19.58 min, t_r (minor) = 14.05 min, ee = 93%; minor isomer: t_r (major) = 10.32 min, t_r (minor) = 15.37 min. dr = 89:11 determined by ^{1}H NMR. $[\alpha]^{25}_D$ = +31.8 (*c* = 0.38, in tetrahydrofuran). **^{1}H NMR (400 MHz, CDCl$_3$)** δ 7.83 – 7.78 (m, 2H), 7.69 – 7.60 (m, 10H), 7.49 – 7.43 (m, 4H), 7.39 – 7.35 (m, 2H), 7.19 – 7.12 (m, 2H), 6.81 – 6.76 (m, 1H), 6.64 – 6.57 (m, 2H), 5.81 (s, 1H), 4.99 (d, *J* = 4.8 Hz, 1H), 4.04 (d, *J* = 4.8 Hz, 1H), 3.83 (s, 3H), 3.08 (s, 1H) ppm; **^{13}C{^{1}H} NMR (101 MHz, CDCl$_3$)** δ 172.2 141.3, 141.0, 140.7, 140.6, 139.9, 128.9, 128.8, 128.7, 127.6, 127.6, 127.5, 127.4, 127.3, 127.1, 127.0, 126.8, 118.5, 114.5, 79.7, 69.6, 67.7, 52.6 ppm; **IR (neat) ν (cm^{-1}):** 2928, 1729, 1599, 1502, 1459, 1269, 1253, 1102, 1028, 732 ppm**; HRMS** (ESI-FT) calcd for $C_{35}H_{31}N_2O_2^+$ ([M]+H$^+$) = 511.2380, found 511.2375.

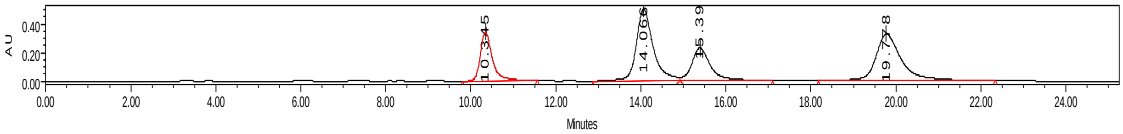

	Retention Time	Area	% Area
1	10.345	6839070	16.33
2	14.066	14176457	33.85
3	15.390	7191904	17.17
4	19.778	13671541	32.65

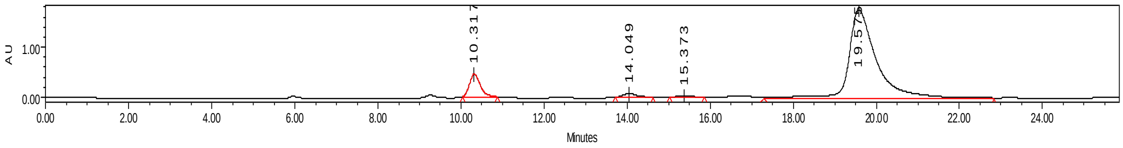

	Retention Time	Area	% Area
1	10.317	8084947	8.99
2	14.049	1639612	1.82
3	15.373	801555	0.89
4	19.575	79441521	88.30

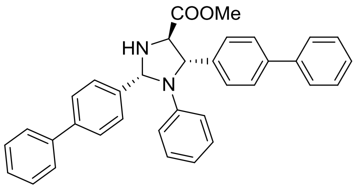

(2*R*,4*R*,5*S*)-**3d**

Methyl (2*R*,4*R*,5*S*)-2,5-di[(1,1'-biphenyl)-4-yl]-1-phenylimidazolidine-4-carboxylate [(2*R*,4*R*,5*S*)-3d]: Procedure A: 38% yield, Colorless oil; R_f = 0.4 (petroleum ether/ethyl acetate = 7/1); Dissolved in iPrOH for HPLC; **HPLC** (Chiralcel IA, hexane/iPrOH = 90/10, flow rate 1.0 mL/min, λ = 254 nm) major isomer: t_r (major) = 13.06 min, t_r (minor) = 18.18 min, ee = –95%; minor isomer: t_r (major) = 14.19 min, t_r (minor) = 9.67. dr = 88:12 determined by ^{1}H NMR. $[\alpha]^{29}_D$ = –45.7 (*c* = 0.40, in tetrahydrofuran). **^{1}H NMR (400 MHz, CDCl$_3$)** δ 7.82 – 7.79 (m, 2H), 7.71 – 7.56 (m, 10H), 7.49 – 7.43 (m, 4H), 7.40 – 7.34 (m, 2H), 7.18 – 7.13 (m, 2H), 6.81 – 6.76 (m, 1H), 6.64 – 6.60 (m, 2H), 5.81 (s, 1H), 4.99 (d, *J* = 4.8 Hz, 1H), 4.04 (d, *J* = 4.8 Hz, 1H), 3.83 (s, 3H), 3.08 (s, 1H) ppm; **^{13}C{^{1}H} NMR (101 MHz, CDCl$_3$)** δ 172.2, 146.9, 141.3, 141.1, 140.7, 139.9, 128.9, 128.8, 127.63, 127.57, 127.5, 127.4, 127.3, 127.1, 127.04, 126.98, 126.8, 118.5, 114.5, 79.7, 69.6, 67.7, 52.6 ppm.

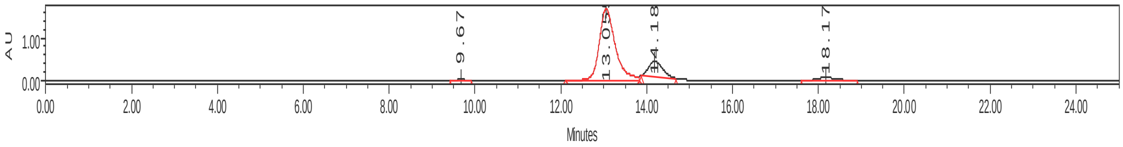

	Retention Time	Area	% Area
1	9.674	475370	0.86
2	13.055	44468600	80.07
3	14.188	8197771	14.76
4	18.175	2394749	4.31

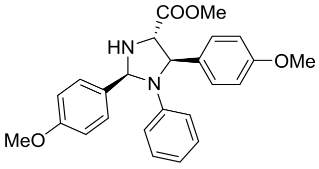

3e

Methyl (2*S*,4*S*,5*R*)-2,5-bis(4-methoxyphenyl)-1-phenylimidazolidine-4-carboxylate (3e): Procedure A: 31% yield, Colorless oil; R_f = 0.4 (petroleum ether/ethyl acetate = 3/1); Dissolved in iPrOH for HPLC; **HPLC** (Chiralcel IF, hexane/iPrOH = 90/10, flow rate 1.0 mL/min, λ = 254 nm) major isomer: t_r (major) = 18.64 min, t_r (minor) = 16.77 min, ee = 73%; minor isomer: t_r (major) = 17.80 min, t_r (minor) = 24.76, ee = 63%. dr = 82:18 determined by ^{1}H NMR. $[\alpha]^{23}_D$ = +12.4 (*c* = 0.20, in tetrahydrofuran, λ = 546 nm). **^{1}H NMR (400 MHz, CDCl$_3$)** δ 7.62 – 7.59 (m, 2H), 7.47 – 7.44 (m, 2H), 7.12 – 7.07 (m, 3H), 6.96 – 6.91 (m, 4H), 6.75 – 6.71 (m, 1H), 6.55 – 6.51 (d, *J* = 8.1 Hz, 2H), 5.65 (s, 1H), 4.84 (d, *J* = 4.8 Hz, 1H), 3.91 (d, *J* = 4.8 Hz, 1H), 3.83 (s, 3H), 3.82 (s, 3H), 3.77 (s, 3H). ppm; **^{13}C{^{1}H} NMR (101 MHz, CDCl$_3$)** δ 172.3, 159.6, 159.1, 147.0, 134.1, 133.1, 128.8, 128.3, 128.1, 127.5, 118.2, 114.5, 114.3,

114.1, 79.5, 69.2, 67.7, 55.32, 55.29, 52.4 ppm; **IR (neat) ν (cm^{-1}):** 2953, 1741, 1605, 1508, 1456, 1302, 1240, 1174, 1033, 832, 752**; HRMS** (ESI-FT) calcd for $C_{25}H_{27}N_2O_4^+$ ([M]+H$^+$) **=** 419.1965, found 419.1963.

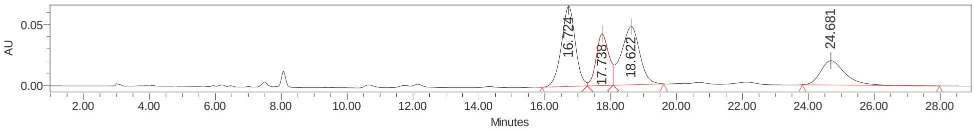

	Retention Time	Area	% Area
1	16.724	1942662	32.52
2	17.738	1121509	18.78
3	18.622	1876647	31.42
4	24.681	1032450	17.28

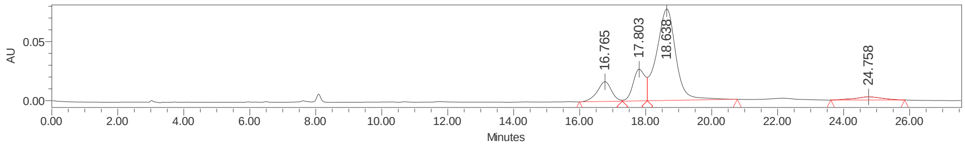

	Retention Time	Area	% Area
1	16.765	504513	11.29
2	17.803	688150	15.40
3	18.638	3118901	69.80
4	24.758	157022	3.51

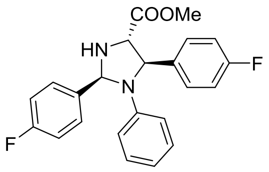

3f

Methyl (2*S*,4*S*,5*R*)-2,5-bis(4-fluorophenyl)-1-phenylimidazolidine-4-carboxylate (3f): Procedure A: 53% yield, Colorless oil; R_f = 0.4 (petroleum ether/ethyl acetate = 6/1); Dissolved in iPrOH for HPLC; **HPLC** (Chiralcel IA, hexane/iPrOH = 90/10, flow rate 1.0 mL/min, λ = 254 nm) major isomer: t_r (major) = 9.39 min, t_r (minor) = 7.71 min, ee = 96%; minor isomer: t_r (major) = 6.95 min, t_r (minor) = 12.33 min. dr = 94:6 determined by ^{1}H NMR. $[\alpha]^{24}_D$ = +38.9 (*c* = 0.33, in tetrahydrofuran). **^{1}H NMR (400 MHz, CDCl$_3$)** δ 7.68 – 7.63 (m, 2H), 7.51 – 7.46 (m, 2H), 7.15 – 7.06 (m, 6H), 6.80 – 6.75 (m, 1H), 6.52 – 6.49 (m, 2H), 5.70 (s, 1H), 4.85 (d, *J* = 5.2 Hz, 1H), 3.89 – 3.85 (m, 1H), 3.79 (s, 3H), 3.01 (s, 1H) ppm; **^{13}C{^{1}H} NMR (101 MHz, CDCl$_3$)** δ 171.9, 162.7 (d, J_{CF} = 247.2 Hz), 162.3 (d, J_{CF} = 247.8 Hz), 146.7, 137.52, 137.49, 136.8, 129.0, 128.8 (d, J_{CF} = 8.1 Hz), 128.7, 127.9 (d, J_{CF} = 8.1 Hz), 118.7, 116.0, 115.7, 115.5, 114.4, 79.3, 69.3, 67.6, 52.6 ppm; **^{19}F{^{1}H} (376 MHz, CDCl$_3$)** δ -113.9, -114.6 ppm; **IR (neat) ν (cm^{-1}):** 2954, 1739, 1600, 1594, 1504, 1435, 1324, 1221, 1156, 1099, 835, 752, 694**; HRMS** (ESI-FT) calcd for $C_{23}H_{21}F_2N_2O_2^+$ ([M]+H$^+$) = 395.1566, found 395.1562.

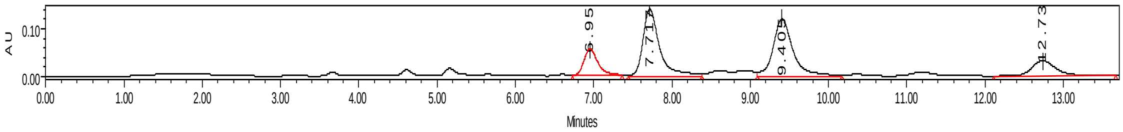

	Retention Time	Area	% Area
1	6.953	674939	12.11
2	7.717	2110532	37.87
3	9.405	2123280	38.10
4	12.735	663995	11.92

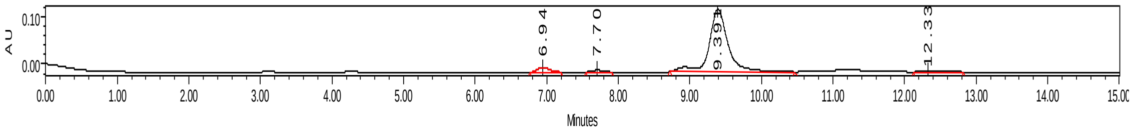

	Retention Time	Area	% Area
1	6.946	108686	4.23
2	7.706	40879	1.59
3	9.391	2408537	93.69
4	12.331	12523	0.49

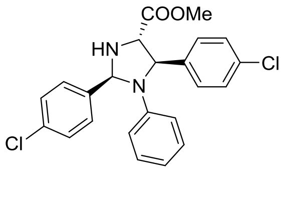

3g

Methyl (2*S*,4*S*,5*R*)-2,5-bis(4-chlorophenyl)-1-phenylimidazolidine-4-carboxylate (3g): Procedure A: 55% yield, Colorless oil; R_f = 0.4 (petroleum ether/ethyl acetate = 6/1); Dissolved in iPrOH for HPLC; **HPLC** (Chiralcel IF, hexane/iPrOH = 90/10, flow rate 1.0 mL/min, λ = 254 nm) major isomer: t_r (major) = 9.08 min, t_r (minor) = 8.63 min, ee = 91%; minor isomer: t_r (major) = 11.70 min, t_r (minor) = 27.55 min. dr = 93:7 determined by ^{1}H NMR. $[\alpha]^{28}_D$ = +71.4 (*c* = 0.23, in tetrahydrofuran). **^{1}H NMR (400 MHz, CDCl$_3$)** δ 7.68 – 7.59 (m, 2H), 7.45 – 7.34 (m, 6H), 7.15 – 7.10 (m, 2H), 6.81 – 6.76 (m, 1H), 6.51 – 6.47 (m, 2H), 5.69 (s, 1H), 4.83 (d, *J* = 5.2 Hz, 1H), 3.86 (d, *J* = 4.8 Hz, 1H), 3.79 (s, 3H), 3.03 (s, 1H) ppm; **^{13}C{^{1}H} NMR (101 MHz, CDCl$_3$)** δ 171.8, 146.6, 140.242 139.5, 134.2, 133.6, 129.2, 129.1, 129.0, 128.9, 128.6, 127.7, 118.9, 114.3, 79.3, 69.3, 67.6, 52.6 ppm; **IR (neat) ν (cm^{-1}):** 2925, 1739, 1599, 1490, 1327, 1210, 1138, 1090, 1013, 824, 751, 694**; HRMS** (ESI-FT) calcd for $C_{23}H_{21}{}^{35}Cl_2N_2O_2^+$ ([M]+H$^+$) = 427.0975, found 427.0975, calcd for $C_{23}H_{21}{}^{35}Cl^{37}Cl N_2O_2^+$ ([M]+H$^+$) = 429.0945, found 429.0942, calcd for $C_{23}H_{21}{}^{37}Cl_2N_2O_2^+$ ([M]+H$^+$) = 430.0975, found 430.0975.

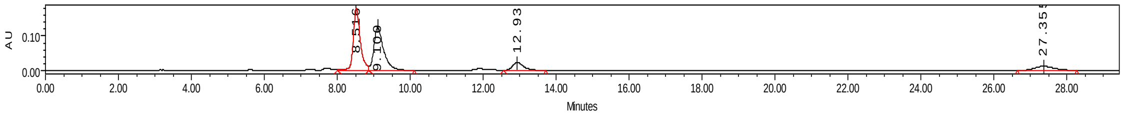

	Retention Time	Area	% Area
1	8.516	2308119	42.19
2	9.100	2252552	41.17
3	12.930	450915	8.24
4	27.355	459318	8.40

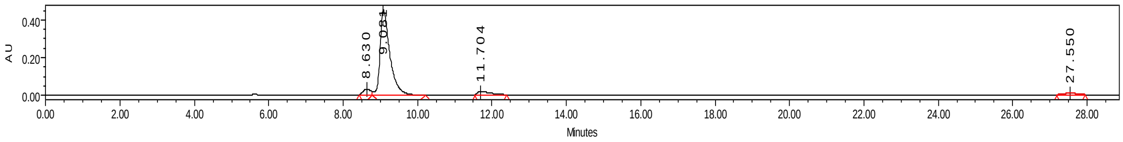

	Retention Time	Area	% Area
1	8.630	437967	4.67
2	9.081	8280607	88.39
3	11.704	430458	4.59
4	27.550	219570	2.34

Procedure B: 53% yield, Colorless oil; R_f = 0.4 (petroleum ether/ethyl acetate = 6/1); Dissolved in iPrOH for HPLC; **HPLC** (Chiralcel IF, hexane/iPrOH = 90/10, flow rate 1.0 mL/min, λ = 254 nm) major isomer: t_r (major) = 9.05 min, t_r (minor) = 8.61 min, ee = 91%; minor isomer: t_r (major) = 11.66 min, t_r (minor) = 27.15 min. dr = 94:6 determined by ^{1}H NMR. $[\alpha]^{28}_D$ = +61.5 (*c* = 0.29, in tetrahydrofuran).

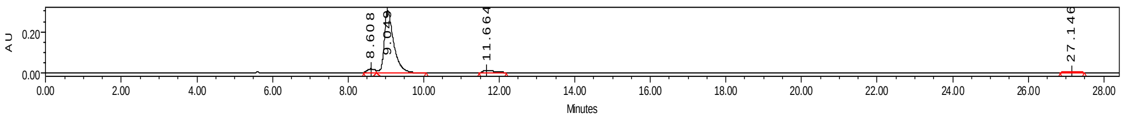

	Retention Time	Area	% Area
1	8.608	241989	4.14
2	9.049	5256353	89.96
3	11.664	238290	4.08
4	27.146	106306	1.82

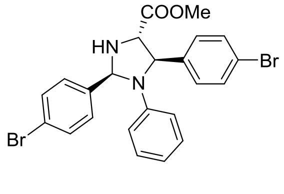

3h

Methyl (2*S*,4*S*,5*R*)-2,5-bis(4-bromophenyl)-1-phenylimidazolidine-4-carboxylate (3h): Procedure A: 66% yield, Pale yellow oil; R_f = 0.4 (petroleum ether/ethyl acetate = 6/1); Dissolved in iPrOH for HPLC; **HPLC** (Chiralcel IF, hexane/iPrOH = 90/10, flow rate 1.0 mL/min, λ = 254 nm) major isomer: t_r (major) = 10.44 min, t_r (minor) = 10.06 min, ee = 96%; minor isomer: t_r (major) = 34.66 min, t_r (minor) = 16.29 min. dr = 95:5 determined by ^{1}H NMR. $[\alpha]^{24}_D$ = +41.4 (*c* = 0.49, in tetrahydrofuran). **^{1}H NMR (400 MHz, CDCl$_3$)** δ 7.56 – 7.50 (m, 6H), 7.39 – 7.36 (m, 2H), 7.15 – 7.10 (m, 2H), 6.81 – 6.77 (m, 1H), 6.50 – 6.47 (m, 2H), 5.68 (s, 1H), 4.81 (d, *J* = 5.2 Hz, 1H), 3.89 – 3.82 (m, 1H), 3.79 (s, 3H), 3.03 (s, 1H) ppm; **^{13}C{^{1}H} NMR (101 MHz, CDCl$_3$)** δ 171.7, 146.6, 140.7, 140.0, 132.1, 131.9, 129.1, 128.9, 128.1, 122.4, 121.7, 118.9 114.3, 79.3, 69.4, 67.5, 52.6 ppm; **IR (neat) ν (cm^{-1}):** 2950, 1739, 1599, 1502, 1487, 1434, 1405, 1328, 1211, 1137, 1071, 1010, 820, 751, 694; **HRMS** (ESI-FT) calcd for $C_{23}H_{21}{}^{79}Br_2N_2O_2^+$ ([M]+H$^+$) = 514.9954, found 514.9964, calcd for $C_{23}H_{21}{}^{79}Br^{81}BrN_2O_2^+$ ([M]+H$^+$) = 516.994, found 516.9933, calcd for $C_{23}H_{21}{}^{81}Br_2N_2O_2^+$ ([M]+H$^+$) = 518.9923, found 518.9910.

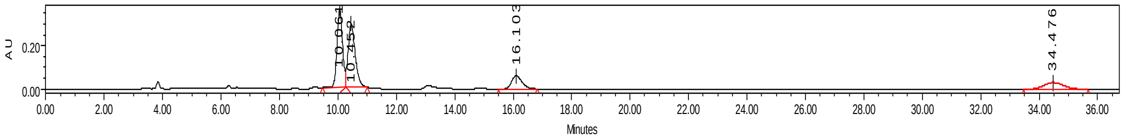

	Retention Time	Area	% Area
1	10.061	4757102	37.12
2	10.452	4981487	38.87
3	16.103	1536507	11.99
4	34.476	1540402	12.02

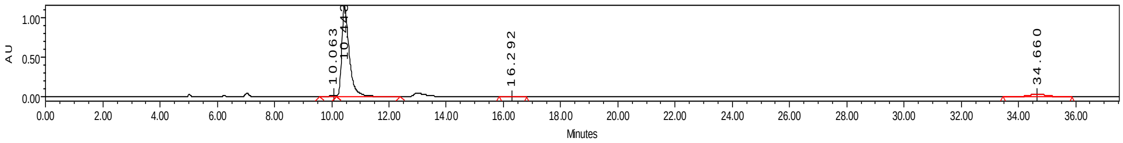

	Retention Time	Area	% Area
1	10.063	357117	1.56
2	10.443	20957493	91.65
3	16.292	83617	0.37
4	34.660	1469577	6.43

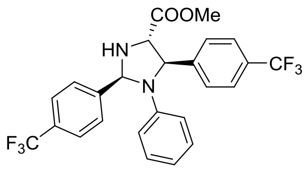

3i

Methyl (2*S*,4*S*,5*R*)-1-phenyl-2,5-bis[4-(trifluoromethyl)phenyl]imidazolidine-4-carboxylate (3i): Procedure A: 52% yield, Colorless oil; R_f = 0.4 (petroleum ether/ethyl acetate = 7/1); Dissolved in iPrOH for HPLC; **HPLC** (Chiralcel IA, hexane/iPrOH = 90/10, flow rate 1.0 mL/min, λ = 254 nm) major isomer: t_r (major) = 9.15 min, t_r (minor) = 8.16 min, ee = 93%; minor isomer: t_r (major) = 10.55 min. dr = 92:8 determined by ^{1}H NMR. $[\alpha]^{22}_D$ = +17.7 (*c* = 0.30, in tetrahydrofuran, λ = 546 nm). **^{1}H NMR (400 MHz, CDCl$_3$)** δ 7.84 – 7.81 (m, 2H), 7.72 – 7.69 (m, 2H), 7.68 – 7.61 (m), 7.17 – 7.12 (m, 2H), 6.86 – 6.79 (m, 1H), 6.51 – 6.47 (m, 2H), 5.81 (s, 1H), 4.93 (d, *J* = 5.6 Hz, 1H), 3.88 (d, *J* = 5.6 Hz, 1H), 3.81 (s, 3H), 3.13 (s, 1H) ppm; **^{13}C{^{1}H} NMR (101 MHz, CDCl$_3$)** δ 171.6, 146.5, 145.6, 145.0, 129.2, 127.6, 126.9 (q, J_{CF} = 273.0 Hz) 126.7, 126.3 (q, J_{CF} = 273.2 Hz) 126.1 (q, J_{CF} = 3.6 Hz), 125.8

(q, J_{CF} = 3.7 Hz), 119.2, 114.3, 79.4, 69.6, 67.5, 52.7 ppm; **$^{19}F\{^1H\}$ (376 MHz, $CDCl_3$)** δ -62.53, -62.54 ppm **IR (neat) ν (cm^{-1}):** 2919, 1740, 1600, 1501, 1420, 1320, 1165, 1068, 837, 753**; HRMS** (ESI-FT) calcd for $C_{25}H_{21}F_6N_2O_2^+$ ([M]+H$^+$) **=** 495.1502, found 495.1503.

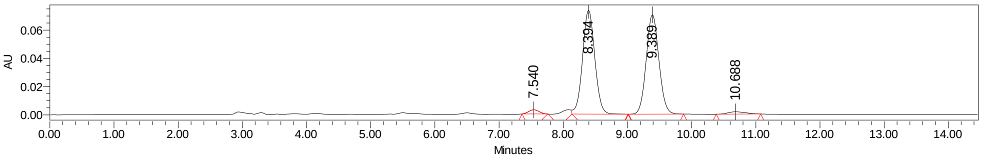

	Retention Time	Area	% Area
1	7.540	33411	1.67
2	8.394	975548	48.64
3	9.389	965337	48.13
4	10.688	31333	1.56

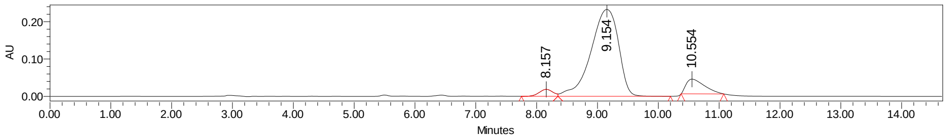

	Retention Time	Area	% Area
1	8.157	290424	3.44
2	9.154	7334844	86.76
3	10.554	828927	9.80

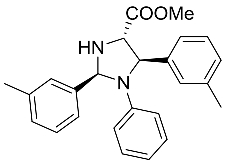

3j

Methyl (2*S*,4*S*,5*R*)-1-phenyl-2,5-di-*m*-tolylimidazolidine-4-carboxylate (3h): Procedure A: 43% yield, Colorless oil; R_f = 0.4 (petroleum ether/ethyl acetate = 7/1); Dissolved in iPrOH for HPLC; **HPLC** (Chiralcel IF, hexane/iPrOH = 90/10, flow rate 1.0 mL/min, λ = 254 nm) major isomer: t_r (major) = 9.94 min, t_r (minor) = 9.38 min, ee = 92%; minor isomer: t_r (major) = 12.90 min, t_r (minor) = 8.93 min. dr = 94:6 determined by ^1H NMR. $[\alpha]^{28}_D$ = +27.5 (*c* = 0.28, in tetrahydrofuran). **^1H NMR (400 MHz, $CDCl_3$)** δ 7.55 – 7.50 (m, 2H), 7.39 – 7.37 (m, 2H), 7.33 – 7.29 (m, 2H), 7.20 – 7.17 (m, 1H), 7.13 – 7.08 (m, 3H), 6.77 – 6.73 (m, 1H), 6.55 – 6.53 (m, 2H), 5.67 (s, 1H), 4.90 (d, *J* = 4.4 Hz, 1H), 3.96 (d, *J* = 4.4 Hz, 1H), 3.79 (s, 3H), 2.92 (s, 1H), 2.40 (s, 3H), 2.37 (s, 3H) ppm; **$^{13}C\{^1H\}$ NMR (101 MHz, $CDCl_3$)** δ 172.3, 147.0, 142.2, 140.7, 138.5, 138.4, 129.1, 128.8, 128.7, 128.6, 128.4, 127.8, 126.9, 124.1, 123.4, 118.2, 114.5, 79.9, 69.7, 67.7, 52.5, 21.6, 21.5 ppm; **IR (neat) ν (cm^{-1}):** 2921, 1739, 1600, 1502, 1447, 1329, 1204, 1037, 752, 694; **HRMS** (ESI-FT) calcd for $C_{25}H_{27}N_2O_2^+$ ([M]+H$^+$) = 387.2067, found 387.2064.

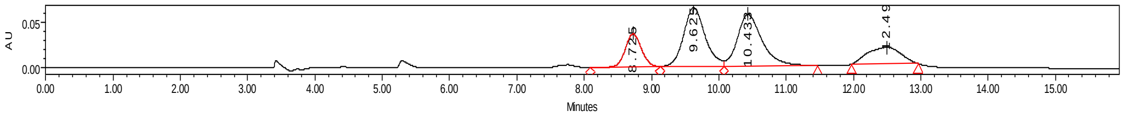

	Retention Time	Area	% Area
1	8.725	607912	14.83
2	9.625	1435081	35.01
3	10.433	1440446	35.14
4	12.495	615945	15.03

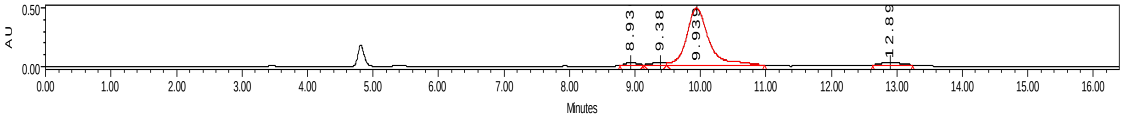

	Retention Time	Area	% Area

1	8.930	252023	1.93
2	9.383	471068	3.61
3	9.939	11897297	91.17
4	12.895	429023	3.29

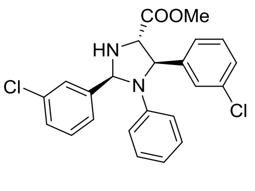

3k

Methyl (2*S*,4*S*,5*R*)-2,5-bis(3-chlorophenyl)-1-phenylimidazolidine-4-carboxylate (3k): Procedure A: 62% yield, Pale yellow oil; R_f = 0.4 (petroleum ether/ethyl acetate = 6/1); Dissolved in iPrOH for HPLC; **HPLC** (Chiralcel IA, hexane/iPrOH = 90/10, flow rate 1.0 mL/min, λ = 254 nm) major isomer: t_r (major) = 7.82 min, t_r (minor) = 6.76 min, ee = 97%; minor isomer: t_r (major) = 6.31 min, t_r (minor) = 7.27 min. dr = 94:6 determined by ^{1}H NMR. $[\alpha]^{23}_D$ = +35.0 (*c* = 0.39, in tetrahydrofuran). **^{1}H NMR (400 MHz, CDCl$_3$)** δ 7.71 – 7.69 (m, 1H), 7.60 – 7.55 (m, 1H), 7.51 – 7.49 (m, 1H), 7.42 – 7.31 (m, 5H), 7.17 – 7.13 (m, 2H), 6.83 – 6.79 (m, 1H), 6.53 – 6.50 (m, 2H), 5.69 (d, *J* = 5.6 Hz, 1H), 4.84 (d, *J* = 5.2 Hz, 1H), 3.92 – 3.86 (m, 1H), 3.80 (s, 3H), 3.05 (s, 1H) ppm; **^{13}C{^{1}H} NMR (101 MHz, CDCl$_3$)** δ 171.7, 146.5, 143.8, 143.0, 134.9, 134.8, 130.3, 130.1, 129.1, 128.6, 128.1, 127.3, 126.5, 125.3, 124.5, 119.0, 114.3, 79.3, 69.4, 67.5, 52.7 ppm; **IR (neat) ν (cm^{-1}):** 2952, 1740, 1598, 1575, 1502, 1475, 1434, 1325, 1205, 1077, 1000, 873, 789, 751, 693; **HRMS** (ESI-FT) calcd for $C_{23}H_{21}{}^{35}Cl_2N_2O_2^+$ ([M]+H$^+$) = 427.0975, found 427.0968, calcd for $C_{23}H_{21}{}^{35}Cl^{37}Cl\,N_2O_2^+$ ([M]+H$^+$) = 429.0945, found 429.0936, calcd for $C_{23}H_{21}{}^{37}Cl_2N_2O_2^+$ ([M]+H$^+$) = 430.0975, found 430.0970.

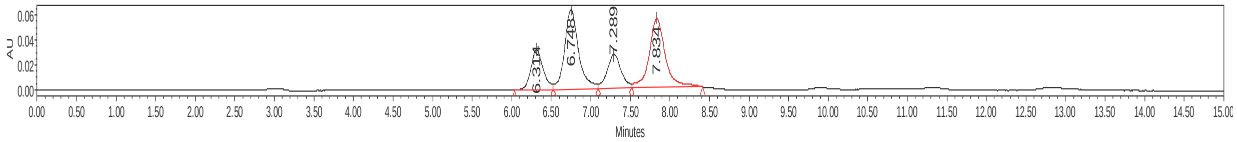

	Retention Time	Area	% Area
1	6.314	349856	15.40
2	6.748	789522	34.76
3	7.289	337320	14.85
4	7.834	794667	34.99

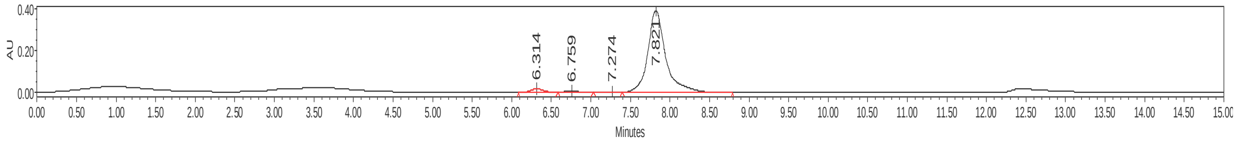

	Retention Time	Area	% Area
1	6.314	202371	3.11
2	6.759	94301	1.45
3	7.274	35710	0.55
4	7.821	6166290	94.89

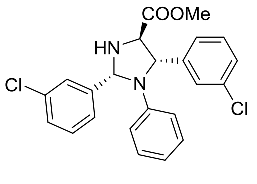

(2*R*,4*R*,5*S*)-**3k**

Methyl (2*R*,4*R*,5*S*)-2,5-bis(3-chlorophenyl)-1-phenylimidazolidine-4-carboxylate [(2*R*,4*R*,5*S*)-3k]: Procedure A: 45% yield, Pale yellow oil; R_f = 0.4 (petroleum ether/ethyl acetate = 7/1); Dissolved in iPrOH for HPLC; **HPLC** (Chiralcel IA, hexane/iPrOH = 90/10, flow rate 1.0 mL/min, λ = 254 nm) major isomer: t_r (major) = 6.68 min, t_r (minor) = 7.34 min, ee = –90%; minor isomer: t_r (major) = 7.12 min, t_r (minor) = 6.28. dr = 86:14 determined by ^{1}H NMR. $[\alpha]^{28}_D$ = –38.8 (*c* = 0.41, in tetrahydrofuran). **^{1}H NMR (400 MHz, CDCl$_3$)** δ 7.71 – 7.69 (m, 1H), 7.59 – 7.56 (m, 1H), 7.51 – 7.49 (m, 1H), 7.41 – 7.32 (m, 5H), 7.16 – 7.13 (m, 2H), 6.83 – 6.79 (m, 1H), 6.54 – 6.49 (m, 2H), 5.69 (s, 1H), 4.83 (d, *J* = 5.2 Hz, 1H), 3.89 (d, *J* = 5.2 Hz, 1H), 3.80 (s, 3H), 3.05 (s, 1H) ppm; **^{13}C{^{1}H} NMR (101 MHz, CDCl$_3$)** δ 171.7, 146.6, 143.9, 143.1, 134.9, 134.8, 130.3, 130.1, 129.1, 128.7, 128.1, 127.3, 126.6, 125.3, 124.5, 119.0, 114.3, 79.4, 69.4, 67.5, 52.7 ppm.

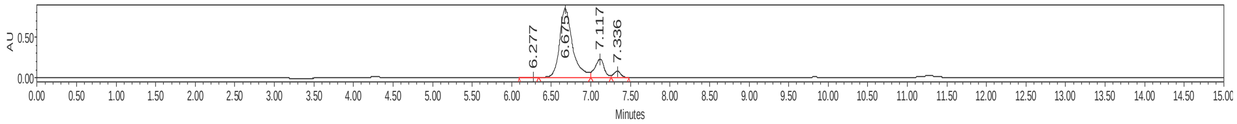

	Retention Time	Area	% Area

1	6.277	94426	0.77
2	6.675	9830224	79.98
3	7.117	1868552	15.20
4	7.336	498401	4.05

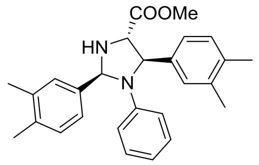

3l

Methyl (2*S*,4*S*,5*R*)-2,5-bis(3,4-dimethylphenyl)-1-phenylimidazolidine-4-carboxylate (3l): Procedure A: 39% yield, Colorless oil; R_f = 0.4 (petroleum ether/ethyl acetate = 8/1); Dissolved in iPrOH for HPLC; **HPLC** (Chiralcel IB, hexane/iPrOH = 95/5, flow rate 1.0 mL/min, λ = 254 nm) major isomer: t_r (major) = 7.75 min, t_r (minor) = 8.50 min, ee = 90%; minor isomer: t_r (major) = 9.76 min, t_r (minor) = 10.29 min. dr = 93:7 determined by ^{1}H NMR. $[\alpha]^{28}_D$ = +18.8 (*c* = 0.13, in tetrahydrofuran). **^{1}H NMR (400 MHz, CDCl$_3$)** δ 7.49 – 7.41 (m, 2H), 7.34 – 7.29 (m, 2H), 7.19 – 7.14 (m, 2H), 7.11 – 7.08 (m, 2H), 6.74 – 6.71 (m, 1H), 6.55 – 6.52 (m, 2H), 5.63 (s, 1H), 4.86 (d, *J* = 4.4 Hz, 1H), 3.94 (d, *J* = 4.0 Hz, 1H), 3.78 (s, 3H), 3.10 (s, 1H), 2.32 – 2.28 (m, 6H), 2.27 (s, 6H) ppm; **^{13}C{^{1}H} NMR (101 MHz, CDCl$_3$)** δ 172.4, 147.1, 139.7, 138.3, 137.0, 136.9, 136.7, 135.9, 130.0, 129.9, 128.8, 128.3, 127.5, 124.4, 123.8, 118.0, 114.4, 79.8, 69.5, 67.8, 52.5, 20.0, 19.9, 19.6, 19.5 ppm; **IR (neat)** ν **(cm^{-1}):** 2920, 1740, 1600, 1502, 1453, 1327, 1202, 1130, 1034, 825, 751, 694; **HRMS** (ESI-FT) calcd for $C_{27}H_{31}N_2O_2^+$ ([M]+H$^+$) = 415.2380, found 415.2374.

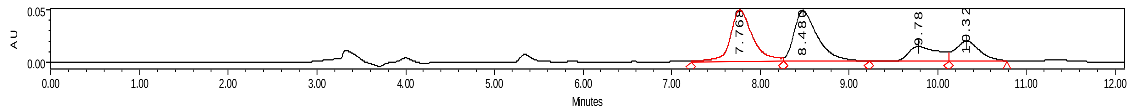

	Retention Time	Area	% Area
1	7.768	858142	35.51
2	8.480	883921	36.57
3	9.782	322495	13.34
4	10.326	352290	14.58

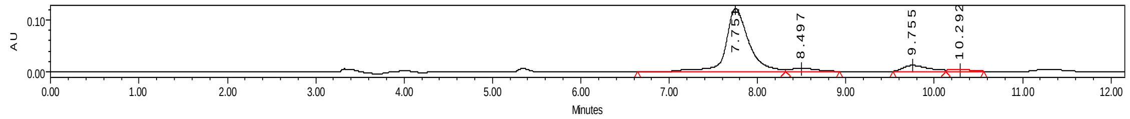

	Retention Time	Area	% Area
1	7.751	2202938	84.49
2	8.497	125667	4.82
3	9.755	220742	8.47
4	10.292	58073	2.23

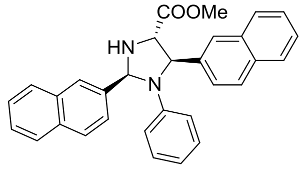

3m

Methyl (2*S*,4*S*,5*R*)-2,5-di(naphthalen-2-yl)-1-phenylimidazolidine-4-carboxylate (3m): Procedure A: 50% yield, Pale yellow oil; R_f = 0.4 (petroleum ether/ethyl acetate = 6/1); Dissolved in iPrOH for HPLC; **HPLC** (Chiralcel IC, hexane/iPrOH = 90/10, flow rate 1.0 mL/min, λ = 254 nm) major isomer: t_r (major) = 18.56 min, t_r (minor) = 11.73 min, ee = 96%; minor isomer: t_r (major) = 10.74 min, t_r (minor) = 17.22 min. dr = 93:7 determined by ^{1}H NMR. $[\alpha]^{29}_D$ = +35.6 (*c* = 0.33, in tetrahydrofuran). **^{1}H NMR (400 MHz, CDCl$_3$)** δ 8.28 – 8.26 (m, 1H), 8.14 – 8.12 (m, 1H), 7.98 – 7.90 (m, 5H), 7.88 – 7.81 (m, 2H), 7.73 – 7.69 (m, 1H), 7.55 – 7.49 (m, 4H), 7.13 – 7.08 (m, 2H), 6.78 – 6.74 (m, 1H), 6.67 – 6.64 (m, 2H), 5.95 (s, 1H), 5.14 (d, *J* = 4.8 Hz, 1H), 4.09 (d, *J* = 4.8 Hz, 1H), 3.82 (s, 3H) , 3.16 (s, 1H) ppm; **^{13}C{^{1}H} NMR (101 MHz, CDCl$_3$)** δ 172.2, 147.0, 139.4, 138.3, 133.40, 133.37, 133.3, 133.0, 128.9, 128.7, 128.2, 128.0, 127.74, 127.72, 126.4, 126.32, 126.29, 126.26, 126.0, 125.3, 125.0, 124.4, 118.5, 114.5, 80.2, 70.1, 67.6, 52.6 ppm; **IR (neat)** ν **(cm^{-1}):** 3055, 2958, 1739, 1599, 1501, 1440, 1320, 1208, 1133, 1037, 859, 822, 751, 694; **HRMS** (ESI-FT) calcd for $C_{31}H_{27}N_2O_2^+$ ([M]+H$^+$) = 459.2067, found 459.2060.

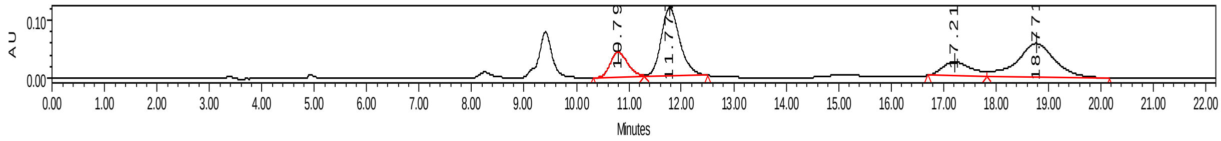

	Retention Time	Area	% Area

1	10.796	993419	12.93
2	11.777	2925697	38.07
3	17.211	918274	11.95
4	18.771	2848608	37.06

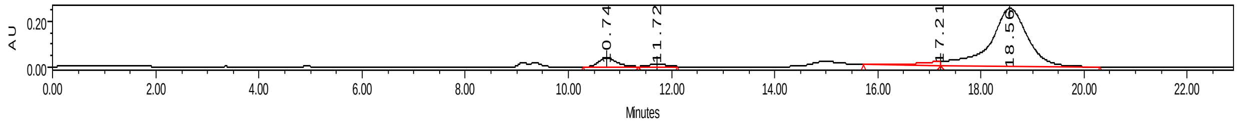

	Retention Time	Area	% Area
1	10.741	942601	6.58
2	11.727	257395	1.80
3	17.217	468048	3.27
4	18.563	12656938	88.36

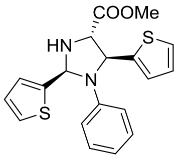

3n

Methyl (2*S*,4*S*,5*S*)-1-phenyl-2,5-di(thiophen-2-yl)imidazolidine-4-carboxylate (3n): Procedure A: 53% yield, White solid; **m.p.**: 118.5 – 124.0 °C; R_f = 0.4 (petroleum ether/ethyl acetate = 6/1); Dissolved in iPrOH for HPLC; **HPLC** (Chiralcel IB, hexane/iPrOH = 95/5, flow rate 1.0 mL/min, λ = 254 nm) major isomer: t_r (major) = 13.24 min, t_r (minor) = 14.96 min, ee = 94%. dr = 94:6 determined by ^{1}H NMR. $[\alpha]^{29}_D$ = +48.5 (*c* = 0.26, in tetrahydrofuran). **^{1}H NMR (400 MHz, CDCl$_3$)** δ 7.35 – 7.30 (m, 2H), 7.25 – 7.22 (m, 1H), 7.20 – 7.13 (m, 3H), 7.06 – 7.00 (m, 2H), 6.83 – 6.78 (m, 1H), 6.72 – 6.69 (m, 2H), 5.93 (s, 1H), 5.12 (d, *J* = 5.2 Hz, 1H), 4.12 (d, *J* = 4.4 Hz, 1H), 3.80 (s, 3H) , 3.23 (s, 1H) ppm; **^{13}C{^{1}H} NMR (101 MHz, CDCl$_3$)** δ 171.4, 146.5, 146.2, 145.9, 129.0, 127.2, 127.0, 125.9, 125.7, 124.8, 124.4, 119.1, 114.2, 76.4, 67.9, 65.3, 52.6 ppm; **IR (neat) ν (cm^{-1}):** 2922, 2851, 1738, 1599, 1501, 1437, 1316, 1219, 1125, 1035, 836, 753, 695; **HRMS** (ESI-FT) calcd for $C_{19}H_{19}N_2O_2S_2^+$ ([M]+H$^+$) = 371.0882, found 371.0875.

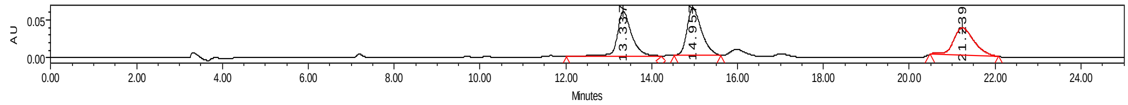

	Retention Time	Area	% Area
1	13.337	1408988	34.42
2	14.957	1457481	35.60
3	21.239	1227237	29.98

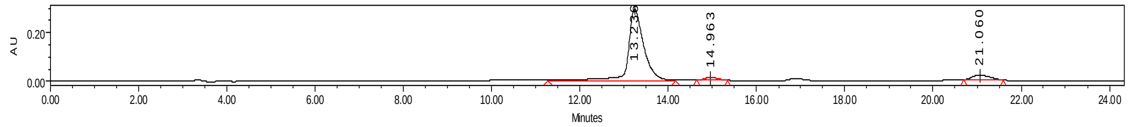

	Retention Time	Area	% Area
1	13.236	7719842	91.08
2	14.963	218515	2.58
3	21.060	537577	6.34

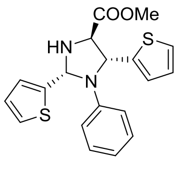

(2*R*,4*R*,5*R*)-**3n**

Methyl (2*R*,4*R*,5*R*)-1-phenyl-2,5-di(thiophen-2-yl)imidazolidine-4-carboxylate [(2*R*,4*R*,5*R*)-3l]: Procedure A: 34% yield, Pale yellow oil; R_f = 0.4 (petroleum ether/ethyl acetate = 6/1); Dissolved in iPrOH for HPLC; **HPLC** (Chiralcel IB, hexane/iPrOH = 95/5, flow rate 1.0 mL/min, λ = 254 nm) major isomer: t_r (major) = 15.42 min, t_r (minor) = 13.57 min, ee = –95%. dr = 90:10 determined by ^{1}H NMR. $[\alpha]^{29}_D$ = –49.6 (*c* = 0.27, in tetrahydrofuran). **^{1}H NMR (400 MHz, CDCl$_3$)** δ 7.34 – 7.29 (m, 2H), 7.25 – 7.22 (m, 1H), 7.18 – 7.13 (m, 3H), 7.05 – 7.00 (m, 2H), 6.83 – 6.77 (m, 1H), 6.72 – 6.69 (m, 2H), 5.92 (s, 1H), 5.12 (d, *J* = 5.2 Hz, 1H), 4.11 (d, *J* = 4.8 Hz, 1H), 3.79 (s, 3H), 3.23 (s, 1H) ppm; **^{13}C{^{1}H} NMR (101 MHz, CDCl$_3$)** δ 171.4, 146.5, 146.2, 145.8, 129.0, 127.2, 127.1, 125.9, 125.7, 124.7, 124.4, 119.1, 114.2, 76.4, 67.9, 65.3, 52.6 ppm.

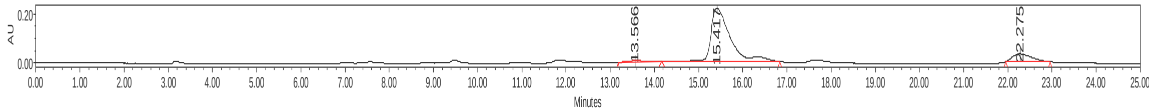

	Retention Time	Area	% Area
1	13.566	202918	2.56
2	15.417	6765283	85.33
3	22.275	960204	12.11

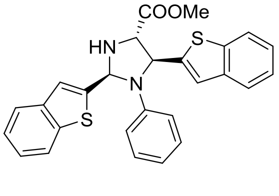

3o

Methyl (2*S*,4*S*,5*S*)-2,5-bis(benzo[b]thiophen-2-yl)-1-phenylimidazolidine-4-carboxylate (3m): Procedure A: 56% yield, Pale yellow oil; R_f = 0.4 (petroleum ether/ethyl acetate = 6/1); Dissolved in iPrOH for HPLC; **HPLC** (Chiralcel IC, hexane/iPrOH = 90/10, flow rate 1.0 mL/min, λ = 254 nm) major isomer: t_r (major) = 18.46 min, t_r (minor) = 13.45 min, ee = 97%; minor isomer: t_r (major) = 26.19 min, t_r (minor) = 16.86 min. dr = 92:8 determined by ^{1}H NMR. $[\alpha]^{23}_D$ = +78.0 (*c* = 0.25, in tetrahydrofuran). **^{1}H NMR (400 MHz, CDCl$_3$)** δ 7.88 – 7.85 (m, 1H), 7.83 – 7.80 (m, 2H), 7.78 – 7.75 (m, 1H), 7.65 – 7.64 (m, 1H), 7.49 – 7.48 (m, 1H), 7.40 – 7.32 (m, 4H), 7.19 – 7.13 (m, 2H), 6.90 – 6.77 (m, 3H), 6.03 (s, 1H), 5.20 (d, *J* = 5.6 Hz, 1H), 4.26 (s, 1H), 3.83 (s, 3H), 3.36 (s, 1H) ppm; **^{13}C{^{1}H} NMR (101 MHz, CDCl$_3$)** δ 171.3, 147.1, 146.5, 146.1, 140.0, 139.8, 139.7, 139.4, 129.1, 124.5, 124.4, 124.34, 124.29, 123.8, 123.5, 122.8, 122.6 122.6, 121.3, 119.5, 114.4, 77.0, 67.5, 65.9, 52.7 ppm; **IR (neat) ν (cm^{-1}):** 2953, 1738, 1599, 1501, 1458, 1436, 1315, 1211, 1065, 931, 834, 749, 693; **HRMS** (ESI-FT) calcd for $C_{27}H_{23}N_2O_2S_2^+$ ([M]+H$^+$) = 471.1195, found 471.1195.

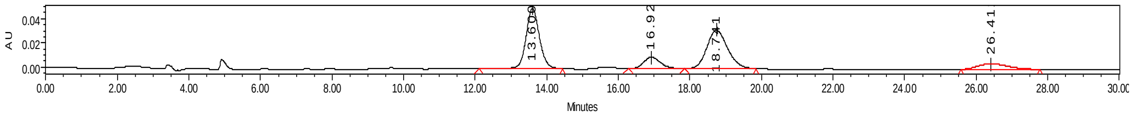

	Retention Time	Area	% Area
1	13.600	1317168	40.96
2	16.922	326391	10.15
3	18.741	1290950	40.14
4	26.411	281495	8.75

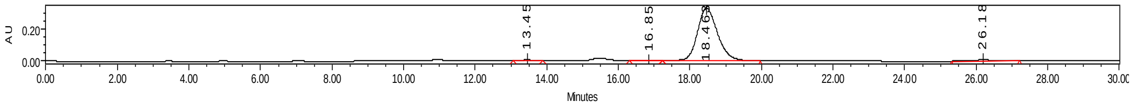

	Retention Time	Area	% Area
1	13.453	207132	1.51
2	16.856	175573	1.28
3	18.463	12979622	94.45
4	26.189	380323	2.77

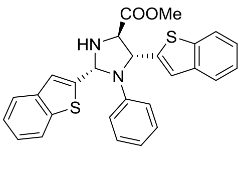

(2*R*,4*R*,5*R*)-**3o**

Methyl (2*R*,4*R*,5*R*)-2,5-bis(benzo[b]thiophen-2-yl)-1-phenylimidazolidine-4-carboxylate [(2*R*,4*R*,5*R*)-3o]: Procedure A: 35% yield, Pale yellow oil; R_f = 0.4 (petroleum ether/ethyl acetate = 6/1); Dissolved in iPrOH for HPLC; **HPLC** (Chiralcel IC, hexane/iPrOH = 90/10, flow rate 1.0 mL/min, λ = 254 nm) major isomer: t_r (major) = 13.76 min, t_r (minor) = 19.82 min, ee = –91%; minor isomer: t_r (major) = 16.52 min, t_r (minor) = 27.21. dr = 95:5 determined by ^{1}H NMR. $[\alpha]^{29}_D$ = –58.3 (*c* = 0.29, in tetrahydrofuran). **^{1}H NMR (400 MHz, CDCl$_3$)** δ 7.88 – 7.84 (m, 1H), 7.83 – 7.79 (m, 2H), 7.77 – 7.74 (m, 1H), 7.64 – 7.62 (m, 1H), 7.49 – 7.47 (m, 1H), 7.44 – 7.32 (m, 4H), 7.18 – 7.13 (m, 2H), 6.86 – 6.78 (m, 3H), 6.03 (s, 1H), 5.20 (d, *J* = 5.6 Hz, 1H), 4.25 (s, 1H), 3.82 (s, 3H), 3.35 (s, 1H) ppm; **^{13}C{^{1}H} NMR (101 MHz, CDCl$_3$)** δ 171.3, 147.1, 146.5, 146.1, 140.0, 139.8, 139.7, 139.4, 129.1, 124.5, 124.41, 124.35, 124.3, 123.8, 123.5, 122.8,

122.61, 122.55, 121.3, 119.5, 114.4, 77.0, 67.5, 65.9, 52.7 ppm.

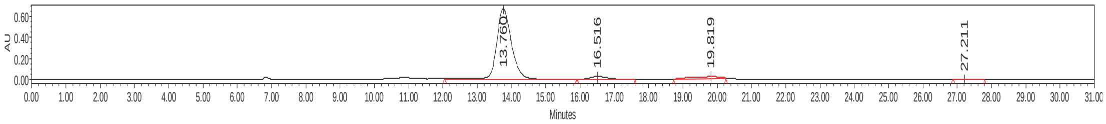

	Retention Time	Area	% Area
1	13.760	20589942	91.31
2	16.516	1048463	4.65
3	19.819	870995	3.86
4	27.211	38898	0.17

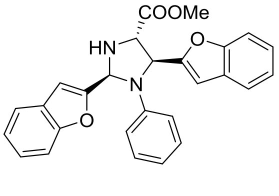

3p

Methyl (2*S*,4*S*,5*S*)-2,5-di(benzofuran-2-yl)-1-phenylimidazolidine-4-carboxylate (3p): Procedure A: 56% yield, Pale yellow oil; R_f = 0.4 (petroleum ether/ethyl acetate = 6/1); Dissolved in iPrOH for HPLC; **HPLC** (Chiralcel IC, hexane/iPrOH = 90/10, flow rate 1.0 mL/min, λ = 254 nm) major isomer: t_r (major) = 13.06 min, t_r (minor) = 11.56 min, ee = 96%; minor isomer: t_r (major) = 14.91 min, t_r (minor) = 12.22 min. dr = 90:10 determined by ^{1}H NMR. $[\alpha]^{25}_D$ = +32.0 (*c* = 0.48, in tetrahydrofuran). **^{1}H NMR (400 MHz, CDCl$_3$)** δ 7.61 – 7.48 (m, 4H), 7.36 – 7.29 (m, 2H), 7.26 – 7.22 (m, 2H), 7.21 – 7.15 (m, 2H), 7.08 – 6.93 (m, 2H), 6.91 – 6.48 (m, 4H) , 5.96 (d, *J* = 5.6 Hz, 1H), 5.13 (d, *J* = 4.8 Hz, 1H), 4.44 – 4.38 (m, 1H), 3.82 (s, 3H), 3.37 (s, 1H) ppm; **^{13}C{^{1}H} NMR (101 MHz, CDCl$_3$)** δ 171.2, 156.0, 155.5, 155.3, 155.1, 145.4, 129.2, 128.2, 128.0, 124.6, 124.3, 123.0, 121.3, 121.1, 118.9, 113.6, 111.5, 111.3, 105.8, 105.0, 73.9, 64.7, 62.3, 52.8 ppm; **IR (neat) ν (cm^{-1}):** 2954, 1742, 1599, 1502, 1453, 1326, 1253, 1208, 1166, 1065, 812, 750, 692; **HRMS** (ESI-FT) calcd for $C_{27}H_{23}N_2O_4^+$ ([M]+H$^+$) = 439.1652, found 439.1645.

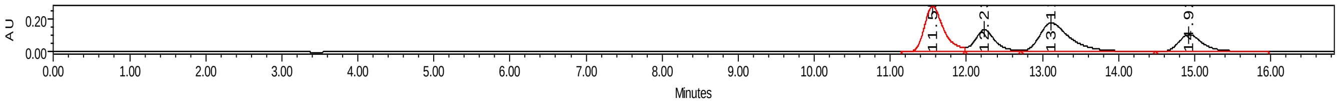

	Retention Time	Area	% Area
1	11.554	4896270	32.85
2	12.237	2545166	17.07
3	13.116	5055356	33.91
4	14.925	2409255	16.16

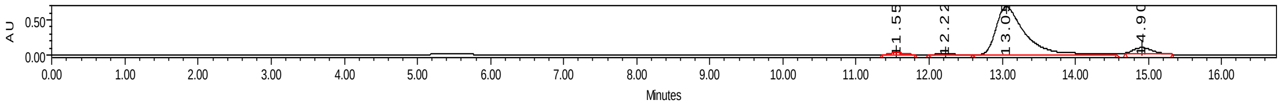

	Retention Time	Area	% Area
1	11.556	413219	1.87
2	12.221	529674	2.39
3	13.055	19726805	89.05
4	14.909	1482964	6.69

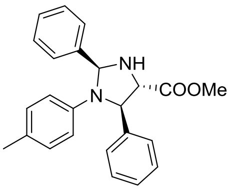

3q

Methyl (2*S*,4*S*,5*R*)-2,5-diphenyl-1-(*p*-tolyl)imidazolidine-4-carboxylate (3q): Procedure A: 41% yield, Colorless oil; R_f = 0.4 (petroleum ether/ethyl acetate = 7/1); Dissolved in iPrOH for HPLC; **HPLC** (Chiralcel IA, hexane/iPrOH = 90/10, flow rate 1.0 mL/min, λ = 254 nm) major isomer: t_r (major) = 8.97 min, t_r (minor) = 7.52 min, ee = 95%; minor isomer: t_r (major) = 8.00 min. dr = 91:9 determined by ^{1}H NMR. $[\alpha]^{24}_D$ = +42.9 (*c* = 0.17, in tetrahydrofuran). **^{1}H NMR (400 MHz, CDCl$_3$)** δ 7.72 – 7.70 (m, 2H), 7.60 – 7.56 (m, 2H), 7.44 – 7.38 (m, 4H), 7.34 – 7.29 (m, 2H), 6.93 – 6.89 (m, 2H), 6.46 – 6.43 (m, 2H) , 5.68 (s, 1H), 4.89 (d, *J* = 4.4 Hz, 1H), 3.96 (d, *J* = 4.4 Hz, 1H), 3.79 (s, 3H), 2.96 (s, 1H), 2.19 (s, 3H) ppm; **^{13}C{^{1}H} NMR (101 MHz, CDCl$_3$)** δ 172.3, 144.8, 142.4, 141.0, 129.4, 128.9, 128.7, 128.4, 128.3, 127.63, 127.60, 127.1, 127.0, 126.3, 114.6, 80.0, 70.0, 67.7, 52.5, 20.2 ppm; **IR (neat) ν (cm^{-1}):** 2951, 1741, 1618, 1510, 1452, 1328, 1209, 1143, 1028, 805, 756, 700; **HRMS** (ESI-FT) calcd for $C_{24}H_{25}N_2O_2^+$ ([M]+H$^+$) = 373.1911, found 373.1911.

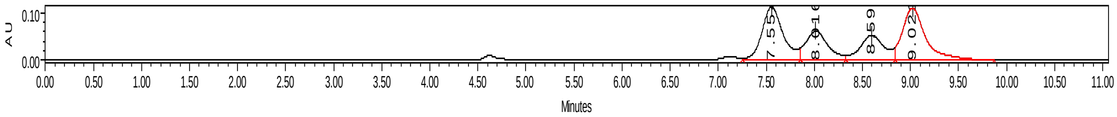

	Retention Time	Area	% Area
1	7.554	1742255	32.00
2	8.016	972943	17.87
3	8.596	919034	16.88
4	9.021	1810454	33.25

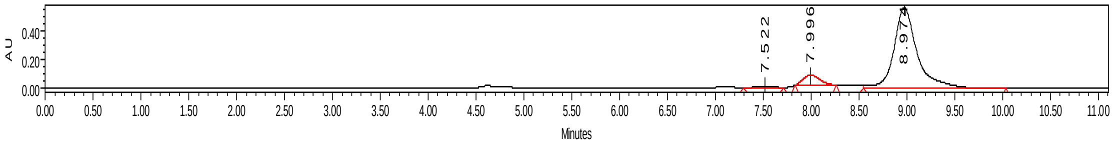

	Retention Time	Area	% Area
1	7.522	260889	2.44
2	7.996	855914	8.02
3	8.974	9553861	89.53

Procedure B: 39% yield, Colorless oil; R_f = 0.4 (petroleum ether/ethyl acetate = 7/1); Dissolved in iPrOH for HPLC; **HPLC** (Chiralcel IA, hexane/iPrOH = 90/10, flow rate 1.0 mL/min, λ = 254 nm) major isomer: t_r (major) = 8.96 min, t_r (minor) = 7.51 min, ee = 93%; minor isomer: t_r (major) = 7.99 min. dr = 94:6 determined by ^{1}H NMR. $[\alpha]^{28}_D$ = +37.6 (c = 0.23, in tetrahydrofuran).

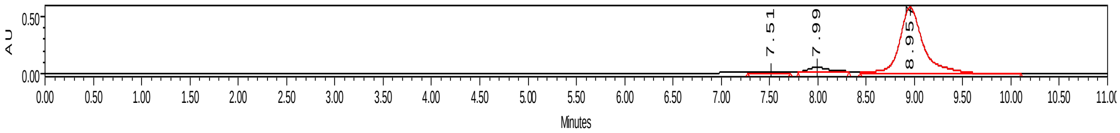

	Retention Time	Area	% Area
1	7.513	393467	3.56
2	7.992	525864	4.76
3	8.957	10122621	91.67

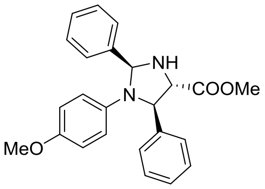

3r

Methyl (2*S*,4*S*,5*R*)-1-(4-methoxyphenyl)-2,5-diphenylimidazolidine-4-carboxylate (3r): Procedure A: 35% yield isolated by preparative TLC, Colorless oil; R_f = 0.4 (petroleum ether/ethyl acetate = 3/1); Dissolved in iPrOH for HPLC; **HPLC** (Chiralcel IC, hexane/iPrOH = 90/10, flow rate 1.0 mL/min, λ = 254 nm) major isomer: t_r (major) = 11.50 min, t_r (minor) = 16.11 min, ee = 95%; minor isomer: t_r (major) = 23.74 min, t_r (minor) = 13.26 min. dr = 92:8 determined by ^{1}H NMR. $[\alpha]^{24}_D$ = +13.7 (c = 0.10, in tetrahydrofuran, λ = 546 nm). **^{1}H NMR (400 MHz, d_6-Acetone)** δ 7.72 – 7.69 (m, 2H), 7.62 – 7.59 (m, 2H), 7.45 – 7.37 (m, 5H), 7.32 – 7.29 (m, 1H), 6.68 – 6.65 (m, 2H), 6.50 – 6.47 (m, 2H), 5.65 (s, 1H), 4.78 (d, J = 5.2 Hz, 1H), 3.88 (d, J = 5.2 Hz, 1H), 3.73 (s, 3H), 3.63 (s, 3H). ppm; **^{13}C{^{1}H} NMR (101 MHz, d_6-Acetone)** δ 173.3, 153.9, 144.2, 143.4, 142.9, 129.8, 129.6, 129.1, 128.6, 128.5, 127.7, 116.8, 115.3, 81.8, 71.6, 69.1, 55.9, 52.7 ppm; **IR (neat) ν (cm^{-1}):** 2955, 1732, 1641, 1513, 1448, 1346, 1242, 1188, 1065, 928, 847, 762; **HRMS** (ESI-FT) calcd for $C_{24}H_{25}N_2O_3^+$ ([M]+H$^+$) = 389.1860, found 389.1859.

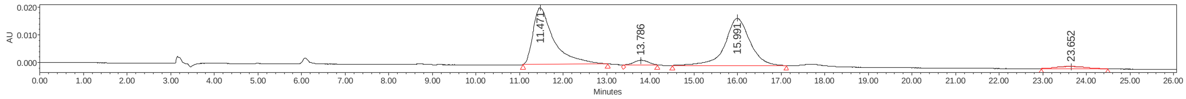

	Retention Time	Area	% Area
1	11.471	681528	47.62

2	13.786	37332	2.61
3	15.991	676095	47.24
4	23.652	36190	2.53

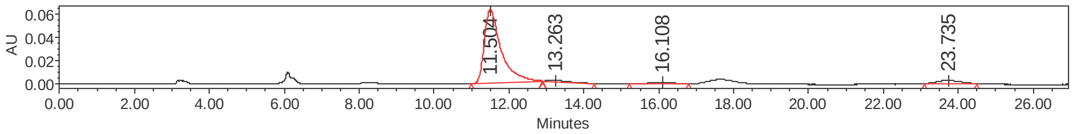

	Retention Time	Area	% Area
1	11.504	1981248	89.98
2	13.263	36535	1.66
3	16.108	56501	2.57
4	23.735	127557	5.79

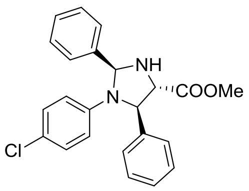

3s

Methyl (2*S*,4*S*,5*R*)-1-(4-chlorophenyl)-2,5-diphenylimidazolidine-4-carboxylate (3s): Procedure A: 44% yield, Colorless oil; R_f = 0.4 (petroleum ether/ethyl acetate = 6/1); Dissolved in iPrOH for HPLC; **HPLC** (Chiralcel IC, hexane/iPrOH = 90/10, flow rate 1.0 mL/min, λ = 254 nm) major isomer: t_r (major) = 7.37 min, t_r (minor) = 10.08 min, ee = 97%; minor isomer: t_r (major) = 13.14 min, t_r (minor) = 8.26 min. dr = 90:10 determined by ^{1}H NMR. $[\alpha]^{23}_D$ = +13.7 (*c* = 0.21, in tetrahydrofuran). **^{1}H NMR (400 MHz, CDCl$_3$)** δ 7.70 – 7.66 (m, 2H), 7.55 – 7.52 (m, 2H), 7.44 – 7.40 (m, 4H), 7.35 – 7.31 (m, 2H), 7.04 – 7.01 (m, 2H) , 6.46 – 6.43 (m, 2H), 5.67 (s, 1H), 4.88 (d, *J* = 4.8 Hz, 1H), 3.97 (d, *J* = 4.4 Hz, 1H), 3.80 (s, 3H), 2.96 (s, 1H) ppm; **^{13}C{^{1}H} NMR (101 MHz, CDCl$_3$)** δ 172.1, 145.5, 141.7, 140.3, 129.0, 128.9, 128.7, 128.6, 127.9, 127.0, 126.2, 123.5, 115.6, 80.0, 69.9, 67.7, 52.6 ppm; **IR (neat) ν (cm^{-1}):** 2952, 1739, 1598, 1496, 1451, 1333, 1209, 1099, 814, 679; **HRMS** (ESI-FT) calcd for $C_{23}H_{22}{}^{35}ClN_2O_2^+$ ([M]+H$^+$) = 393.1364, found 393.1364, calcd for $C_{23}H_{22}{}^{37}ClN_2O_2^+$ ([M]+H$^+$) = 395.1335, found 395.1334.

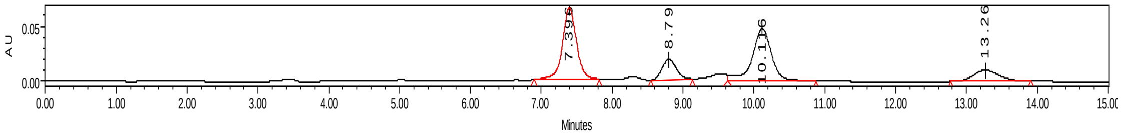

	Retention Time	Area	% Area
1	7.396	900275	38.20
2	8.797	259447	11.01
3	10.116	934296	39.64
4	13.263	262764	11.15

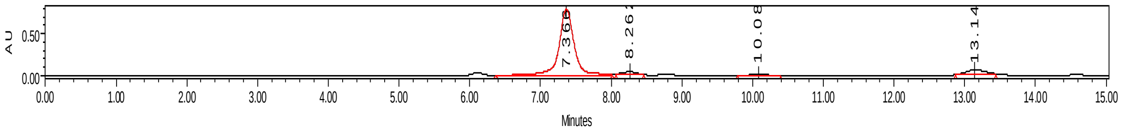

	Retention Time	Area	% Area
1	7.366	12063330	88.57
2	8.262	384393	2.82
3	10.084	193259	1.42
4	13.140	979593	7.19

Procedure B: 44% yield, Colorless oil; R_f = 0.4 (petroleum ether/ethyl acetate = 6/1); Dissolved in iPrOH for HPLC; **HPLC** (Chiralcel IC, hexane/iPrOH = 90/10, flow rate 1.0 mL/min, λ = 254 nm) major isomer: t_r (major) = 7.40 min, t_r (minor) = 10.16 min, ee = 97%; minor isomer: t_r (major) = 13.30 min, t_r (minor) = 8.30 min. dr = 94:6 determined by ^{1}H NMR. $[\alpha]^{28}_D$ = +14.3 (*c* = 0.18, in tetrahydrofuran).

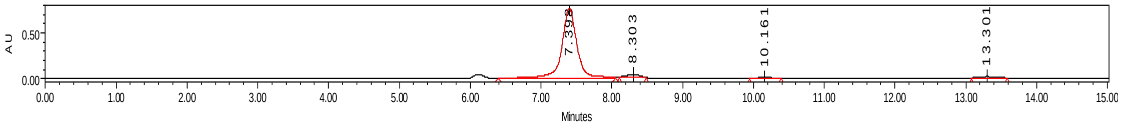

	Retention Time	Area	% Area
1	7.398	11733266	93.81
2	8.303	381248	3.05
3	10.161	144656	1.16
4	13.301	248757	1.99

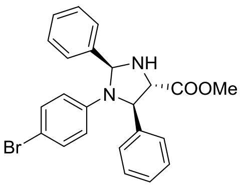

3t

Methyl (2*S*,4*S*,5*R*)-1-(4-bromophenyl)-2,5-diphenylimidazolidine-4-carboxylate (3t): Procedure A: 46% yield, Colorless oil; R_f = 0.4 (petroleum ether/ethyl acetate = 6/1); Dissolved in iPrOH for HPLC; **HPLC** (Chiralcel IC, hexane/iPrOH = 90/10, flow rate 1.0 mL/min, λ = 254 nm) major isomer: t_r (major) = 7.25 min, t_r (minor) = 10.15 min, ee = 97%; minor isomer: t_r (major) = 8.15 min, t_r (minor) = 12.53 min. dr = 94:6 determined by ^{1}H NMR. $[\alpha]^{27}_D$ = +38.6 (*c* = 0.18, in tetrahydrofuran). **^{1}H NMR (400 MHz, CDCl$_3$)** δ 7.69 – 7.65 (m, 2H), 7.55 – 7.51 (m, 2H), 7.45 – 7.39 (m, 4H), 7.36 – 7.31 (m, 2H), 7.18 – 7.15 (m, 2H), 6.41 – 6.37 (m, 2H), 5.66 (s, 1H), 4.87 (d, *J* = 4.8 Hz, 1H), 3.96 (d, *J* = 4.4 Hz, 1H), 3.80 (s, 3H), 2.96 (s, 1H) ppm; **^{13}C{^{1}H} NMR (101 MHz, CDCl$_3$)** δ 172.1, 145.9, 141.6 140.2, 131.6, 129.0, 128.9, 128.6, 127.9, 127.0, 126.2, 116.1, 110.7, 79.9, 69.8, 67.7, 52.6 ppm; **IR (neat)** ν **(cm^{-1}):** 2952, 1739, 1592, 1493, 1452, 1333, 1210, 1074, 811, 759, 700; **HRMS** (ESI-FT) calcd for $C_{23}H_{22}{}^{79}Br\ N_2O_2^+$ ([M]+H$^+$) = 437.0859, found 437.0860, calcd for $C_{23}H_{22}{}^{81}Br\ N_2O_2^+$ ([M]+H$^+$) = 439.0839, found 439.0839.

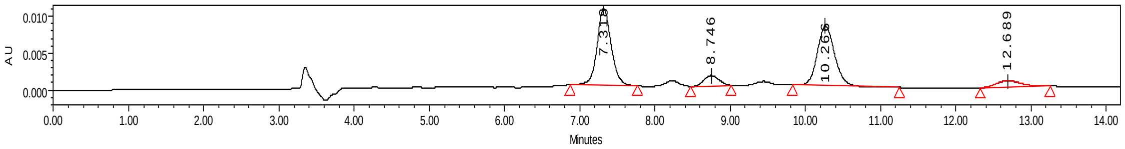

	Retention Time	Area	% Area
1	7.318	136774	44.58
2	8.746	19641	6.40
3	10.266	129826	42.31
4	12.689	20578	6.71

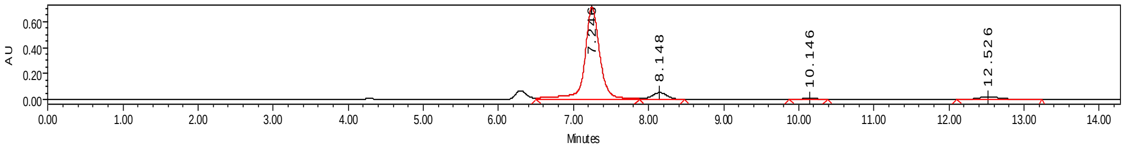

	Retention Time	Area	% Area
1	7.246	10234865	89.59
2	8.148	779289	6.82
3	10.146	181145	1.59
4	12.526	228548	2.00

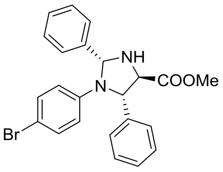

(2*R*,4*R*,5*S*)-**3t**

Methyl (2*R*,4*R*,5*S*)-1-(4-bromophenyl)-2,5-diphenylimidazolidine-4-carboxylate [(2*R*,4*R*,5*S*)-3t]: Procedure A: 36% yield, Colorless oil; R_f = 0.4 (petroleum ether/ethyl acetate = 6/1); Dissolved in iPrOH for HPLC; **HPLC** (Chiralcel IC, hexane/iPrOH = 90/10, flow rate 1.0 mL/min, λ = 254 nm) major isomer: t_r (major) = 10.55 min, t_r (minor) = 7.26 min, ee = –91%; minor isomer: t_r (major) = 8.60 min, t_r (minor) = 13.10. dr = 88:12 determined by ^{1}H NMR. $[\alpha]^{29}_D$ = –28.6 (*c* = 0.28, in tetrahydrofuran). **^{1}H NMR (400 MHz, CDCl$_3$)** δ 7.69 – 7.63 (m, 2H), 7.57 – 7.47 (m, 2H), 7.45 – 7.38 (m, 4H), 7.36 – 7.31 (m, 2H), 7.18 – 7.15 (m, 2H), 6.41 – 6.36 (m, 2H), 5.66 (s, 1H), 4.87 (d, *J* = 4.0 Hz, 1H), 3.97 (d, *J* = 4.4 Hz, 1H), 3.80 (s, 3H), 2.96 (s, 1H) ppm; **^{13}C{^{1}H} NMR (101 MHz, CDCl$_3$)** δ 172.1, 145.9, 141.6, 140.2, 131.6, 129.0, 128.9, 128.6, 127.9, 127.0, 126.2, 116.1, 110.7, 79.9, 69.8, 67.7, 52.6 ppm.

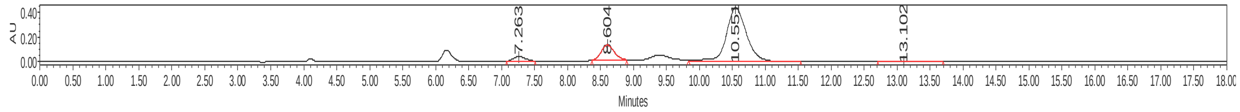

	Retention Time	Area	% Area
1	7.263	436201	3.79
2	8.604	1641641	14.26
3	10.551	9339474	81.11
4	13.102	97066	0.84

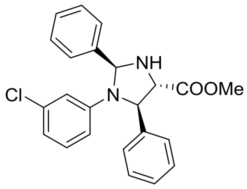

3u

Methyl (2*S*,4*S*,5*R*)-1-(3-chlorophenyl)-2,5-diphenylimidazolidine-4-carboxylate (3u): Procedure A: 35% yield, Colorless oil; R_f = 0.4 (petroleum ether/ethyl acetate = 6/1); Dissolved in iPrOH for HPLC; **HPLC** (Chiralcel IC, hexane/iPrOH = 90/10, flow rate 1.0 mL/min, λ = 254 nm) major isomer: t_r (major) = 7.45 min, t_r (minor) = 8.49 min, ee = 87%; minor isomer: t_r (major) = 11.91 min, t_r (minor) = 7.89 min. dr = 92:8 determined by ^{1}H NMR. $[\alpha]^{23}_D$ = +13.2 (c = 0.18, in tetrahydrofuran). **^{1}H NMR (400 MHz, CDCl$_3$)** δ 7.70 – 7.66 (m, 2H), 7.56 – 7.51 (m, 2H), 7.47 – 7.40 (m, 4H), 7.36 – 7.30 (m, 2H), 7.02 – 6.97 (m, 1H), 6.73 – 6.70 (m, 1H), 6.53 – 6.51 (m, 1H), 6.43 – 6.36 (m, 1H), 5.70 (s, 1H), 4.89 (d, J = 4.4 Hz, 1H), 3.97 (d, J = 4.4 Hz, 1H), 3.80 (s, 3H), 2.98 (s, 1H) ppm; **^{13}C{^{1}H} NMR (101 MHz, CDCl$_3$)** δ 172.0, 148.1, 141.4, 140.2, 134.6, 129.8, 129.0, 128.9, 128.8, 128.6, 127.9, 127.0, 126.3, 118.4, 114.4, 112.6, 79.9, 69.7, 67.7, 52.6 ppm; **IR (neat) ν (cm^{-1}):** 2952, 1739, 1563, 1505, 1485, 1452, 1337, 1210, 1097, 1009, 762, 700; **HRMS** (ESI-FT) calcd for $C_{23}H_{22}{}^{35}ClN_2O_2^+$ ([M]+H$^+$) = 393.1364, found 393.1364, calcd for $C_{23}H_{22}{}^{37}ClN_2O_2^+$ ([M]+H$^+$) = 395.1335, found 395.1335.

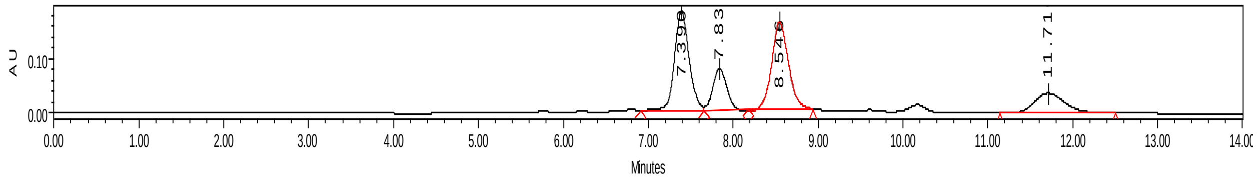

	Retention Time	Area	% Area
1	7.390	2217938	35.61
2	7.839	879219	14.12
3	8.546	2207103	35.44
4	11.710	923456	14.83

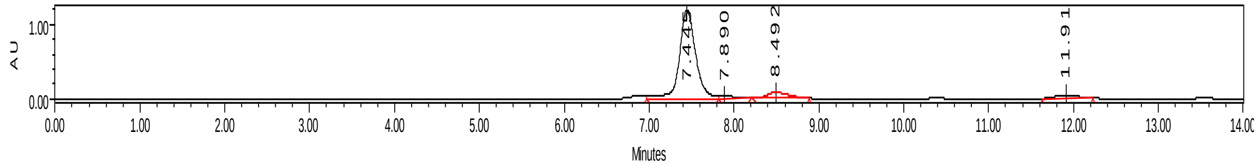

	Retention Time	Area	% Area
1	7.445	15749017	85.46
2	7.890	461393	2.50
3	8.492	1427259	7.75
4	11.910	790344	4.29

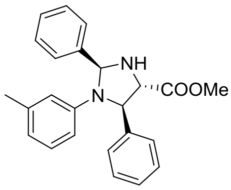

3v

Methyl (2*S*,4*S*,5*R*)-2,5-diphenyl-1-(*m*-tolyl)imidazolidine-4-carboxylate (3v): Procedure B: 41% yield, Colorless oil; R_f = 0.4 (petroleum ether/ethyl acetate = 7/1); Dissolved in iPrOH for HPLC; **HPLC** (Chiralcel IH, hexane/iPrOH = 90/10, flow rate 1.0 mL/min, λ = 254 nm) major isomer: t_r (major) = 9.46 min, t_r (minor) = 15.21 min, ee = 98%; minor isomer: t_r (major) = 11.13 min, t_r (minor) = 8.05 min. dr = 92:8 determined by ^{1}H NMR. $[\alpha]^{28}_D$ = +32.1 (*c* = 0.21, in tetrahydrofuran). **^{1}H NMR (400 MHz, CDCl$_3$)** δ 7.73 – 7.69 (m, 2H), 7.58 – 7.54 (m, 2H), 7.44 – 7.37 (m, 4H), 7.34 – 7.29 (m, 2H), 7.01 – 6.97 (m, 1H), 6.60 – 6.57 (m, 1H), 6.38 – 6.33 (m, 2H) , 5.72 (s, 1H), 4.91 (d, *J* = 4.8 Hz, 1H), 3.95 (d, *J* = 4.4 Hz, 1H), 3.79 (s, 3H), 2.98 (s, 1H), 2.17 (s, 3H) ppm; **^{13}C{^{1}H} NMR (101 MHz, CDCl$_3$)** δ 172.2, 147.1, 142.1, 140.9, 138.6, 128.9, 128.7, 128.7, 128.3, 127.6, 127.1, 126.3, 119.3, 115.1, 111.6, 79.9, 69.8, 67.7, 52.5, 21.8 ppm; **IR (neat) ν (cm^{-1}):** 3029, 2925, 1739, 1604, 1582, 1494, 1452, 1335, 1210, 1134, 1027, 769, 699; **HRMS** (ESI-FT) calcd for $C_{24}H_{25}N_2O_2^+$ ([M]+H$^+$) = 373.1911, found 373.1907.

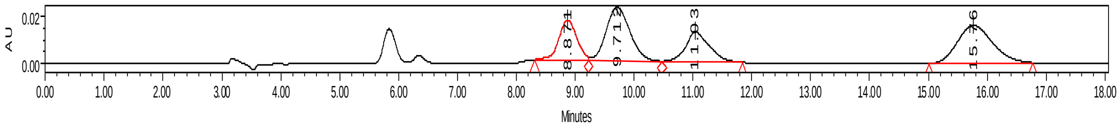

	Retention Time	Area	% Area
1	8.871	357032	17.92
2	9.712	630025	31.63
3	11.036	350000	17.57
4	15.767	654993	32.88

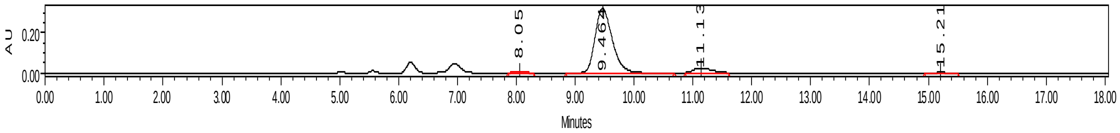

	Retention Time	Area	% Area
1	8.051	81948	1.14
2	9.464	6555020	91.34
3	11.134	490773	6.84
4	15.212	48695	0.68

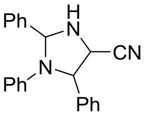

***rac*-3da**

1,2,5-Triphenylimidazolidine-4-carbonitrile (3da): Procedure A: 10% yield, White solid; **m.p.**: 137.3 – 142.5 °C; R_f = 0.7 (petroleum ether/ethyl acetate = 7/1); **^{1}H NMR (400 MHz, CDCl$_3$)** δ 7.76 – 7.65 (m, 2H), 7.60 – 7.57 (m, 2H), 7.51 – 7.32 (m, 6H), 7.15 – 7.10 (m, 2H), 6.83 – 6.78 (m, 1H), 6.55 – 6.51 (m, 2H), 5.76 (d, J = 8.8 Hz, 1H), 5.06 (d, J = 3.6 Hz, 1H), 4.15 (dd, J = 7.6, 3.6 Hz, 1H), 2.78 (t, J = 8.4 Hz, 1H) ppm; **^{13}C{^{1}H} NMR (101 MHz, CDCl$_3$)** δ 146.3, 140.4, 139.1, 129.3, 129.0, 128.9, 128.8, 128.5, 127.5, 127.0, 126.1, 119.3, 118.5, 114.9, 79.5, 70.8, 55.8 ppm; **IR (neat) ν (cm^{-1}):** 3032, 2923, 1600, 1499, 1453, 1320, 1030, 961, 752, 697; **HRMS** (ESI-FT) calcd for $C_{22}H_{20}N_3^+$ ([M]+H$^+$) = 326.1652, found 326.1652.

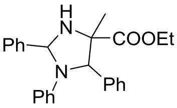

3ala

Ethyl 4-methyl-1,2,5-triphenylimidazolidine-4-carboxylate (3ala): Procedure A: 13% yield, Colorless oil; R_f = 0.6 (petroleum ether/ethyl acetate = 7/1); dr > 95:5 determined by ^{1}H NMR. **^{1}H NMR (400 MHz, CDCl$_3$)** δ 7.68 – 7.64 (m, 2H), 7.50 – 7.39 (m, 5H), 7.35 – 7.27 (m, 3H), 7.10 – 7.05 (m, 2H), 6.75 – 6.70 (t, *J* = 7.3 Hz, 1H), 6.50 – 6.45 (m, 2H), 5.59 (s, 1H), 4.65 (s, 1H), 3.73 – 3.65 (m, 1H), 3.55 – 3.50 (m, 1H), 1.66 (s, 3H), 0.91 (t, *J* = 7.2 Hz, 3H) ppm; **^{13}C{^{1}H} NMR (101 MHz, CDCl$_3$)** δ 171.1, 147.0, 141.1, 138.9, 128.83, 128.78, 128.4, 128.3, 127.9, 127.4, 127.1, 118.3, 114.6, 76.6, 70.3, 61.3, 23.9, 13.6 ppm; **IR (neat) ν (cm^{-1}):** 2922, 1735, 1599, 1499, 1453, 1261, 1153, 1025, 737, 699**; HRMS** (ESI-FT) calcd for $C_{25}H_{27}N_2O_2^+$ ([M]+H$^+$) = 387.2067, found 387.2071.

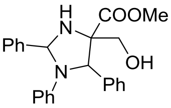

3ser

Methyl 4-(hydroxymethyl)-1,2,5-triphenylimidazolidine-4-carboxylate (3ser): Procedure A: 13% yield, Colorless oil; R_f = 0.3 (petroleum ether/ethyl acetate = 2/1); dr = 86:14 determined by ^{1}H NMR. **^{1}H NMR (400 MHz, CDCl$_3$)** δ 7.69 – 7.53 (m, 2H), 7.49 – 7.42 (m, 2H), 7.40 – 7.33 (m, 2H), 7.32 – 7.24 (m, 4H), 7.02 – 6.98 (m, 2H), 6.67 – 6.65 (m, 1H), 6.41 – 6.36 (m, 2H), 5.63 (s, 1H), 4.91 (s, 1H), 3.79 (s, 3H), 3.21 (d, *J* = 11.4, 1H), 3.04 (d, *J* = 11.8 Hz, 1H), 1.81 (s, 1H) ppm; **^{13}C{^{1}H} NMR (101 MHz, CDCl$_3$)** δ 173.6, 146.8, 141.6, 138.3, 128.84, 128.81, 128.2, 127.4, 126.7, 118.6, 114.6, 78.3, 72.7, 71.2, 63.7, 53.1 ppm; **IR (neat) ν (cm^{-1}):** 3332, 2921, 1736, 1600, 1500, 1453, 1327, 1217, 1073, 1034, 752, 699**; HRMS** (ESI-FT) calcd for $C_{24}H_{24}N_2O_3^+$ ([M]+H$^+$) **=** 389.1860, found 389.1851.

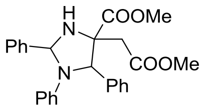

3asp

Methyl 4-(2-methoxy-2-oxoethyl)-1,2,5-triphenylimidazolidine-4-carboxylate (3ala): Procedure A: 11% yield, Colorless oil; R_f = 0.6 (petroleum ether/ethyl acetate = 7/1); dr = 94:6 determined by ^{1}H NMR. **^{1}H NMR (400 MHz, $CDCl_3$)** δ 7.58 – 7.55 (m, 2H), 7.41 – 7.38 (m, 2H), 7.35 – 7.30 (m, 3H), 7.27 – 7.21 (m, 3H), 7.03 – 6.98 (m, 2H), 6.69 – 6.65 (m, 1H), 6.43 – 6.40 (m, 2H), 5.53 (s, 1H), 4.60 (s, 1H), 3.63 (s, 3H), 3.24 (d, J = 15.6 Hz, 1H), 3.15 (s, 3H), 2.84 (d, J = 15.6 Hz, 1H) ppm; **^{13}C{^{1}H} NMR (101 MHz, $CDCl_3$)** δ 170.1, 128.84, 128.75, 128.5, 128.3, 128.1, 127.5, 127.2, 118.7, 114.8, 77.9, 75.0, 71.8, 52.1, 51.9, 41.1 ppm; **IR (neat) ν (cm^{-1}):** 2918, 1742, 1599, 1439, 1261, 1202, 752, 698**; HRMS** (ESI-FT) calcd for $C_{26}H_{27}N_2O_4^+$ ([M]+H$^+$) **=** 431.1965, found 431.1964.

(N) Copies of NMR Spectra

^{1}H NMR and $^{13}C\{^{1}H\}$ NMR spectra of compound **L-Ra(OiBu)₂** in $CDCl_3$

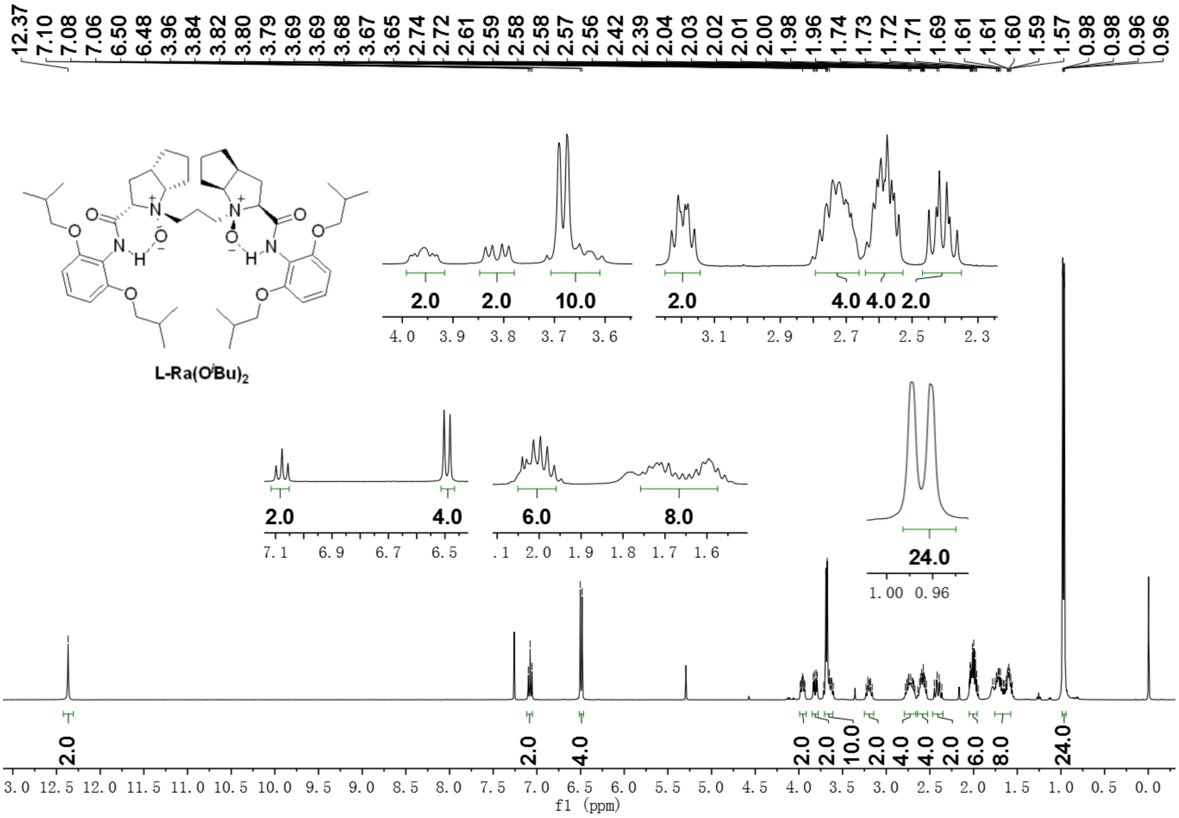

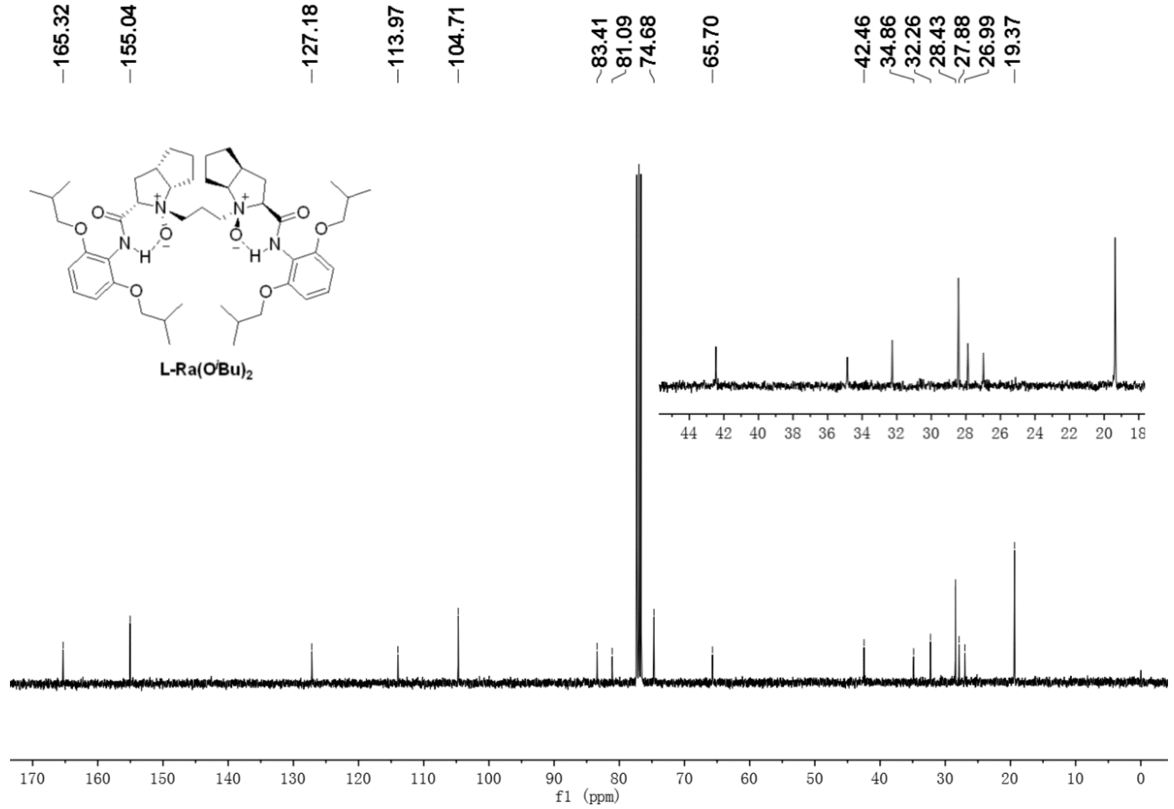

^{1}H NMR and $^{13}C\{^{1}H\}$ NMR spectra of compound **L-Pe(OiBu)$_2$** in $CDCl_3$

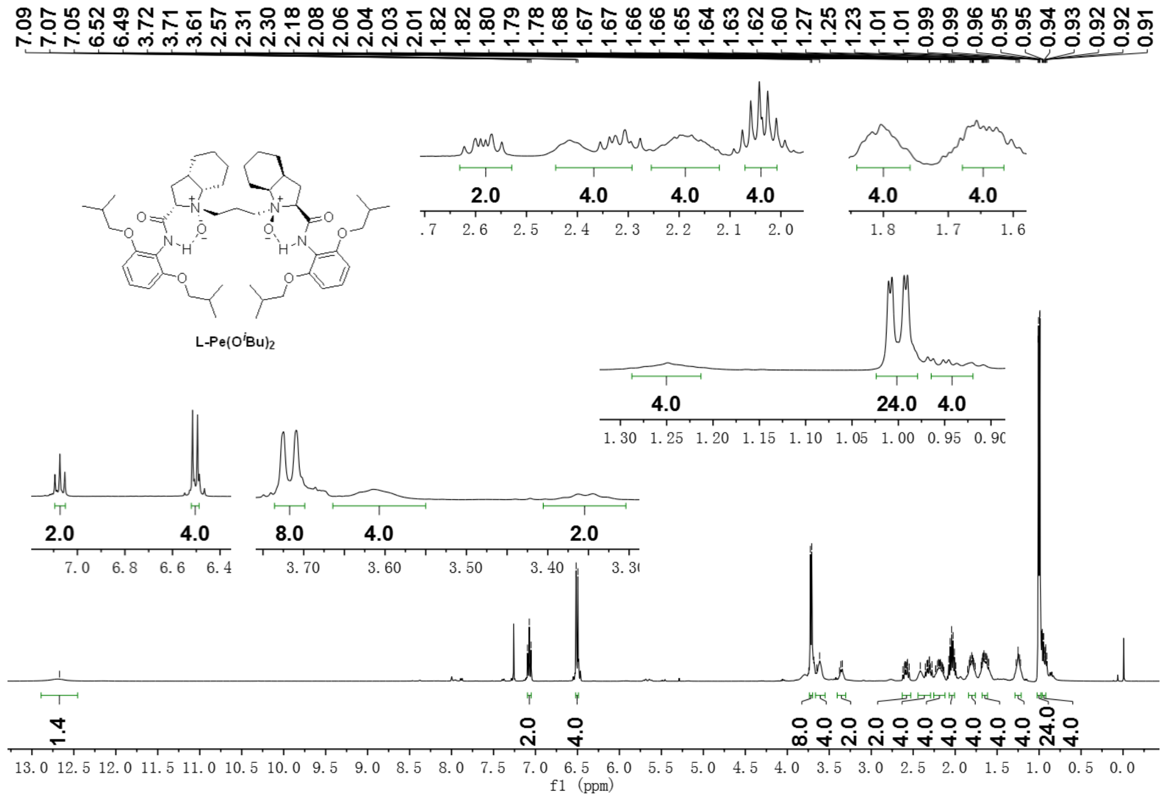

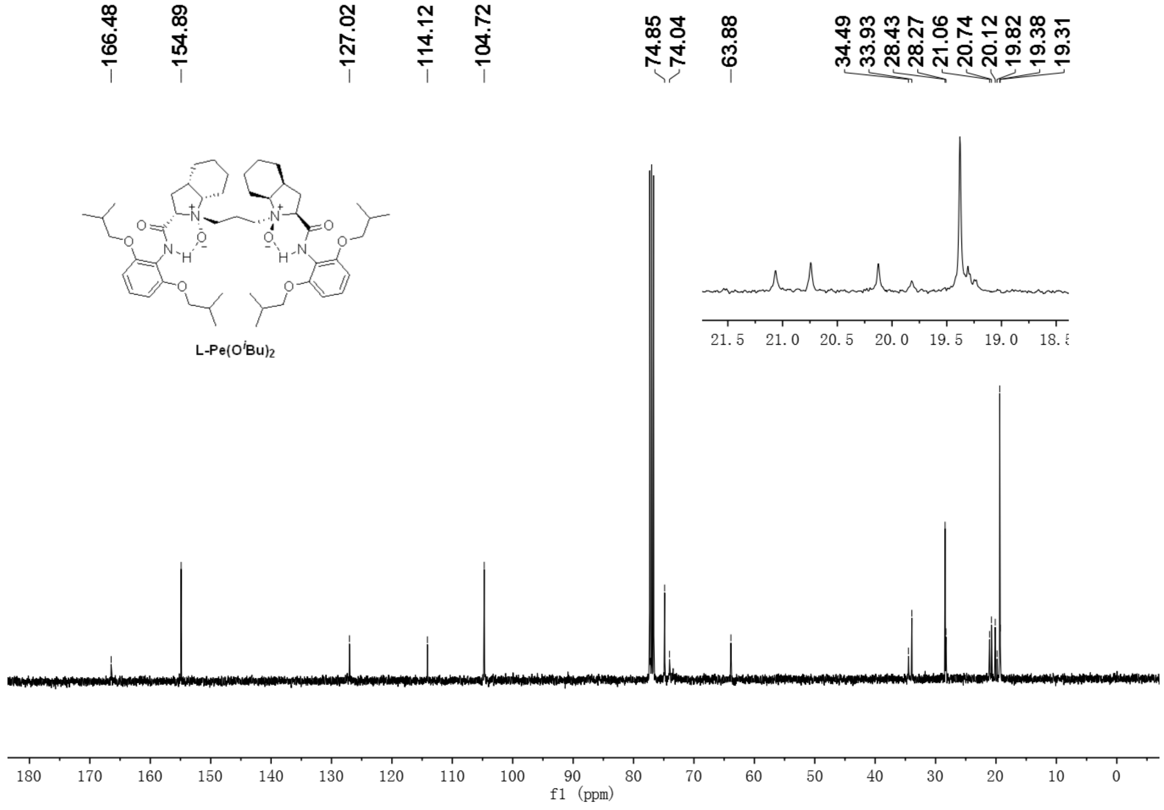

^{1}H NMR and $^{13}C\{^{1}H\}$ NMR spectra of compound of **6a** in $CDCl_3$

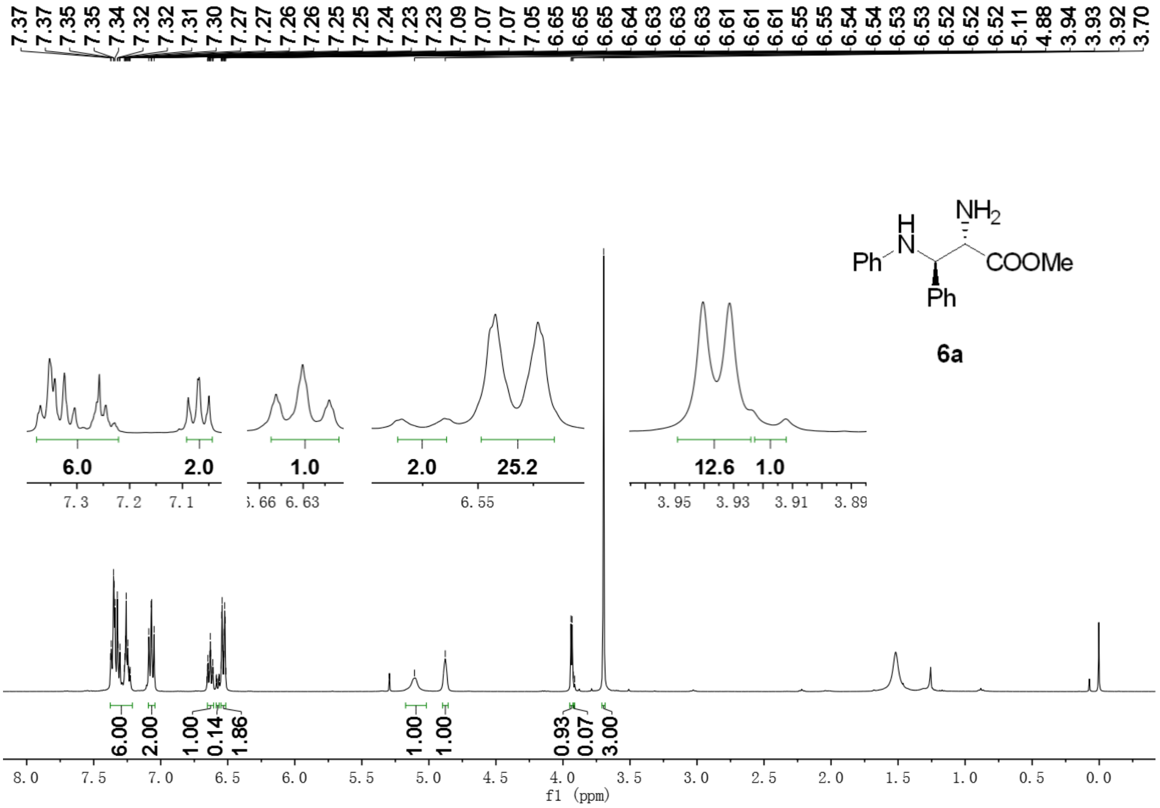

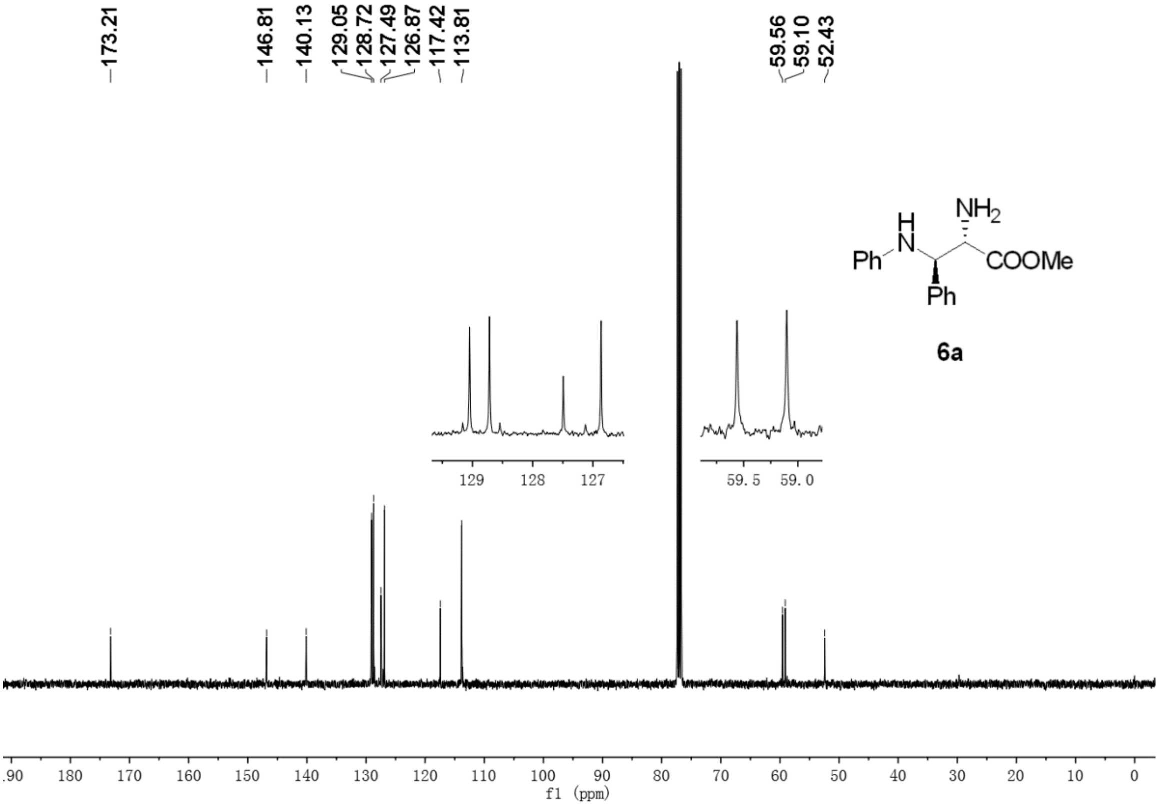

^{1}H NMR and ^{13}C{^{1}H} NMR spectra of compound of **6b** in $CDCl_3$

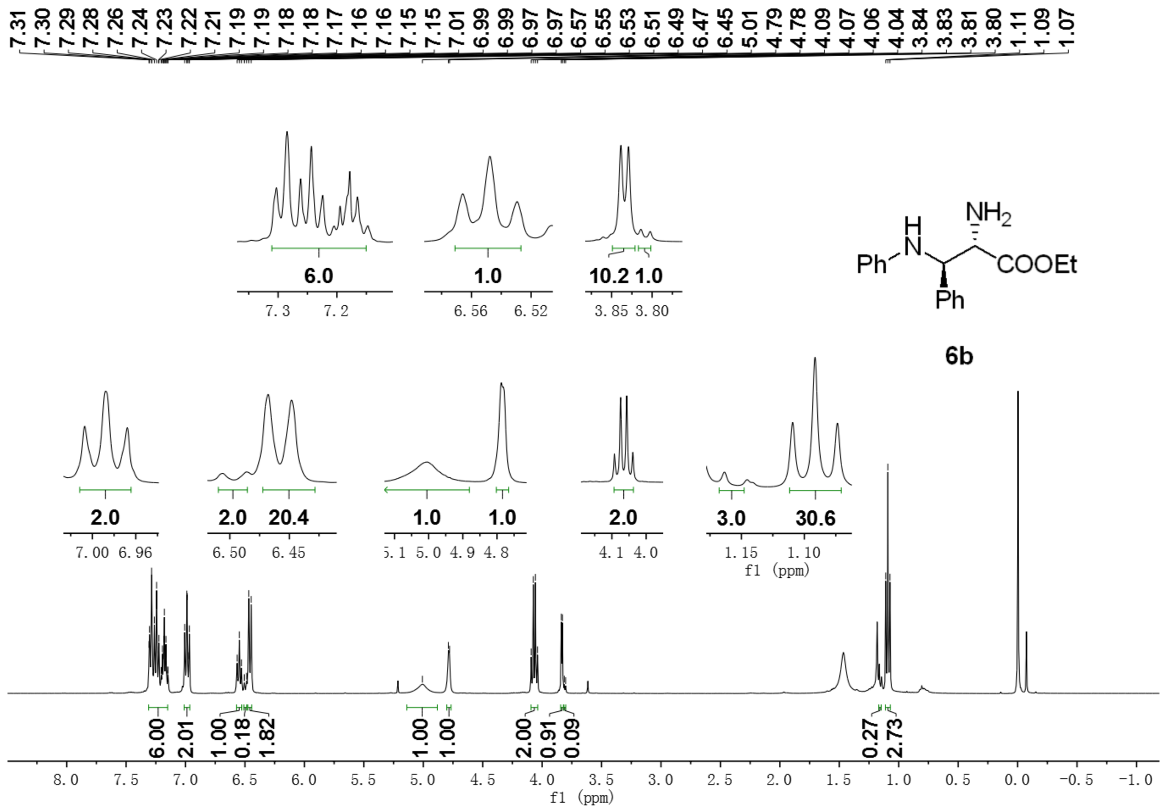

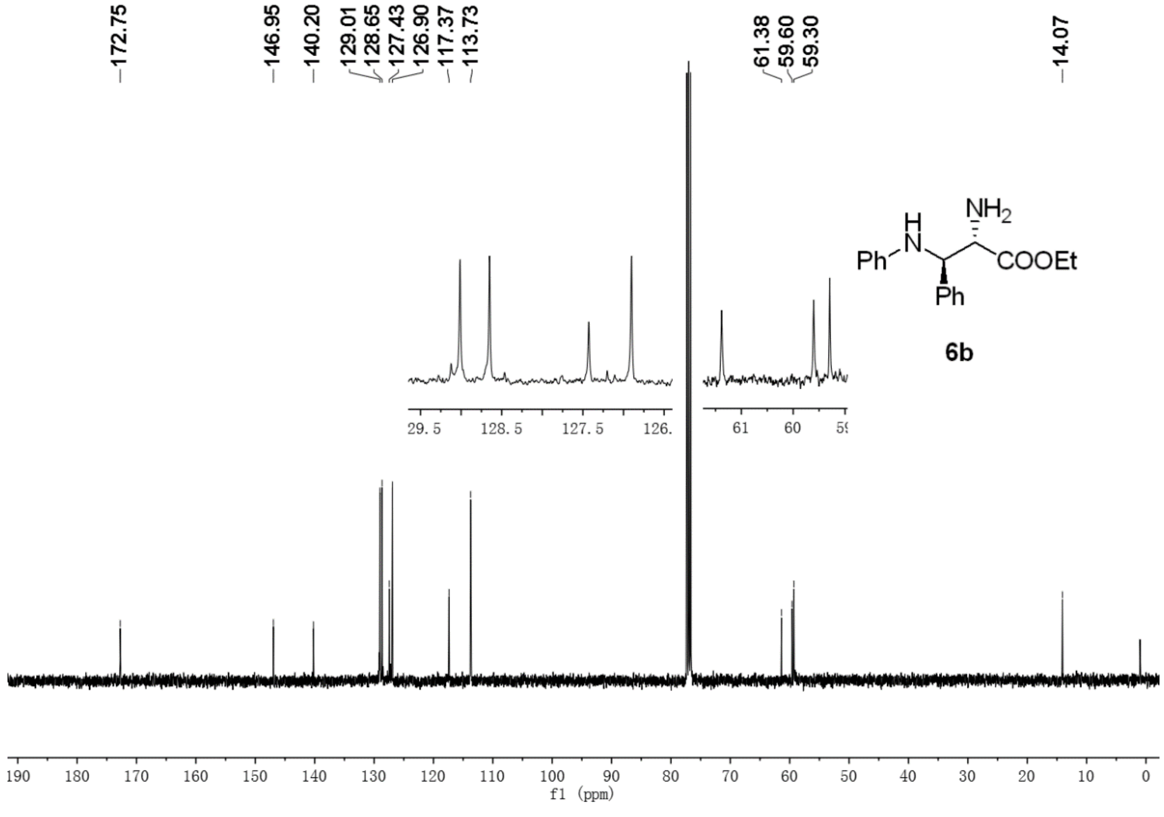

^{1}H NMR and ^{13}C{^{1}H} NMR spectra of compound of (2*R*,3*S*)-**6b** in $CDCl_3$

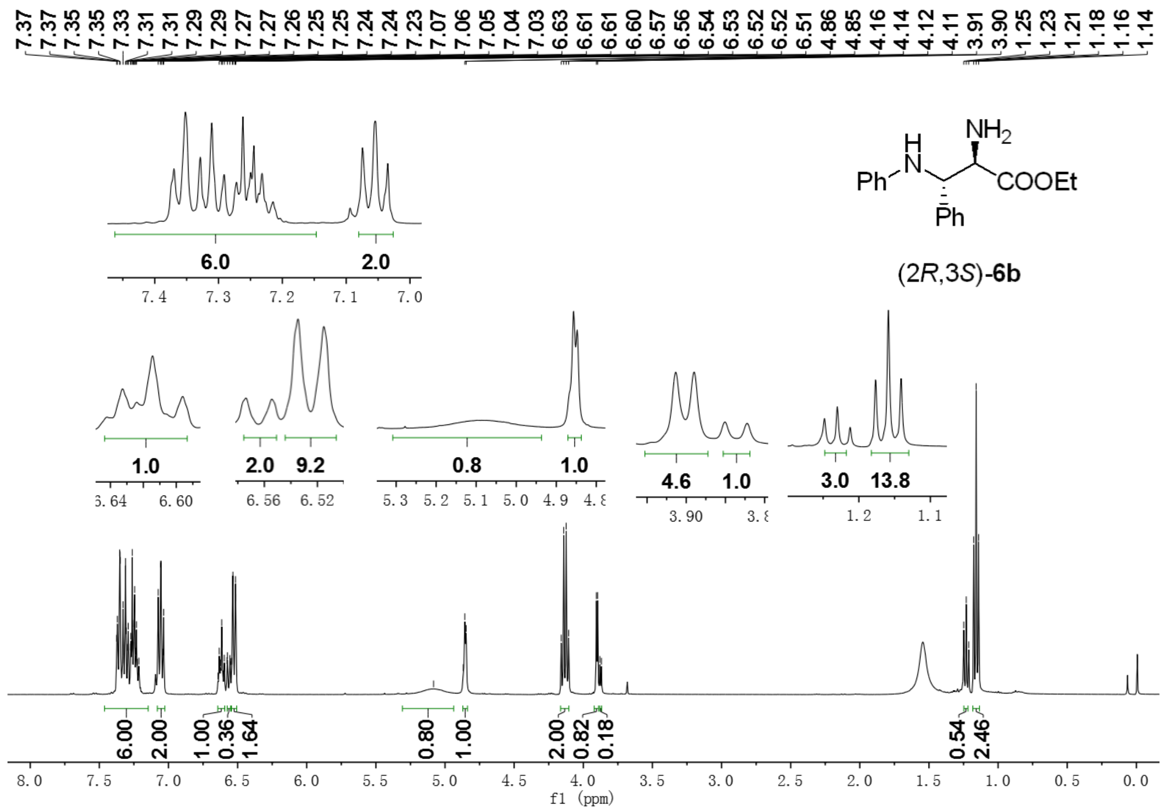

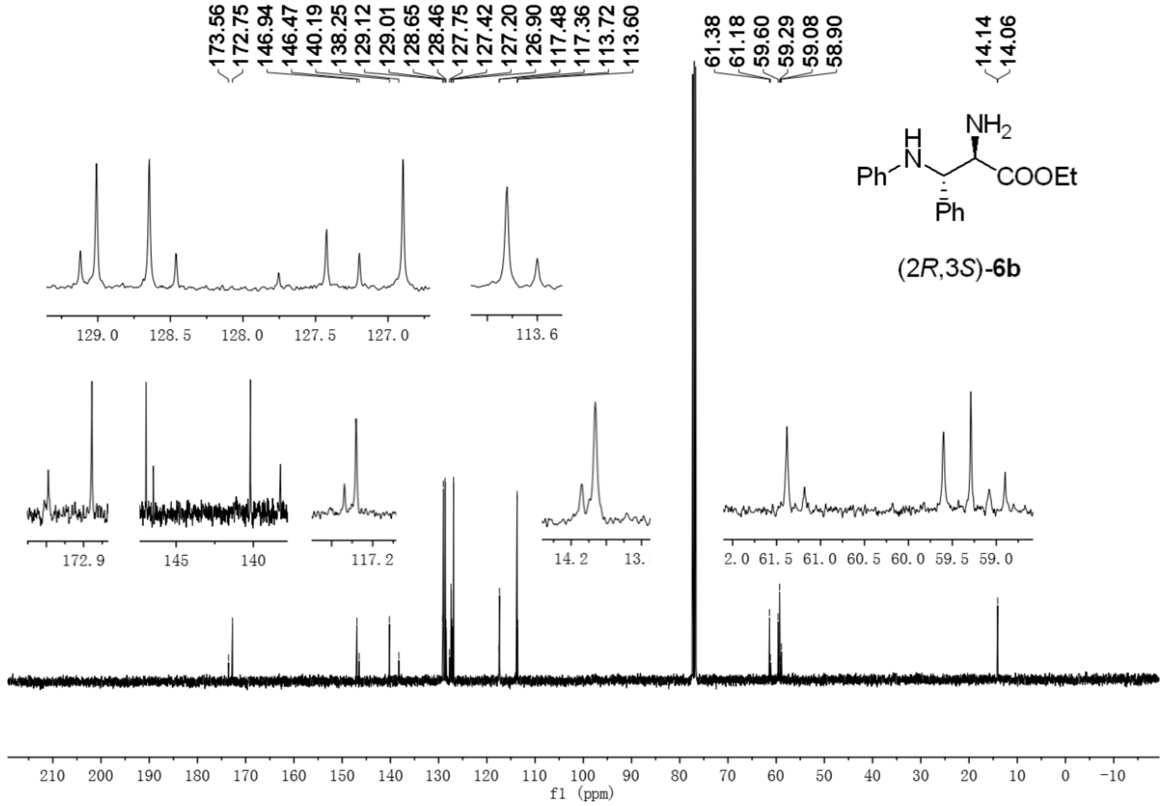

^{1}H NMR and $^{13}C\{^{1}H\}$ NMR spectra of compound of **6c** in $CDCl_3$

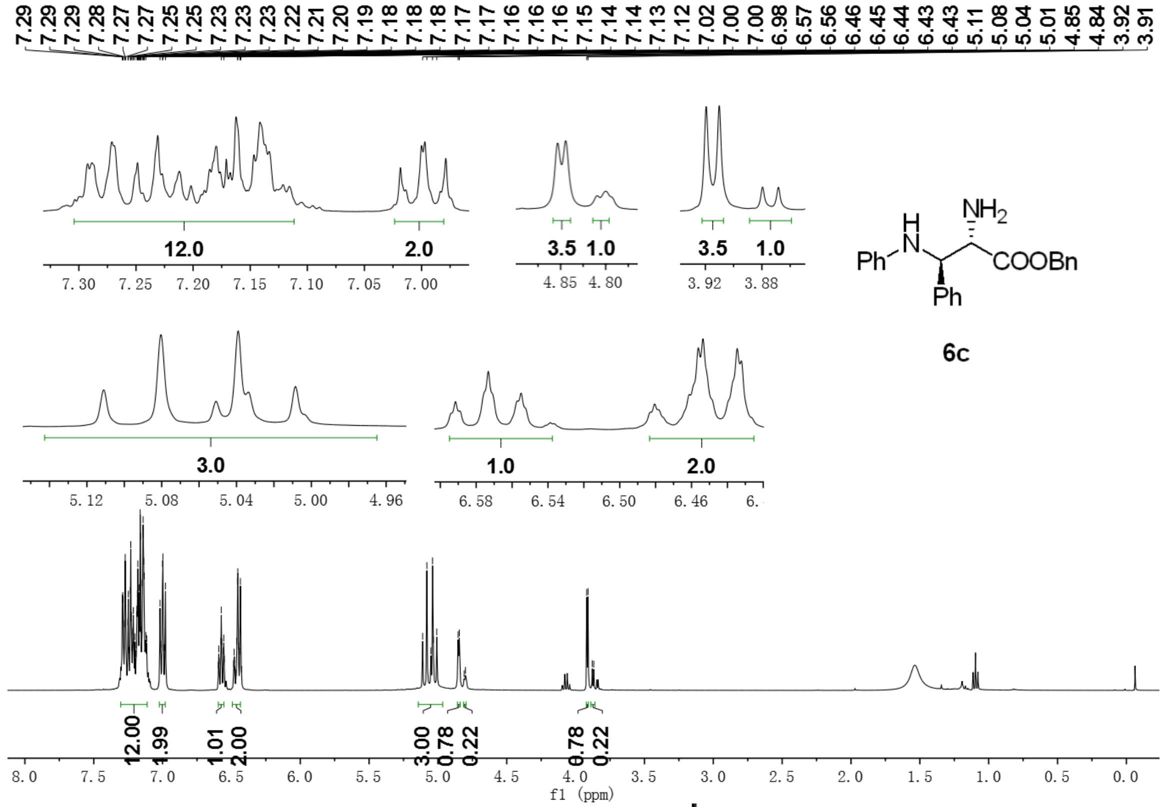

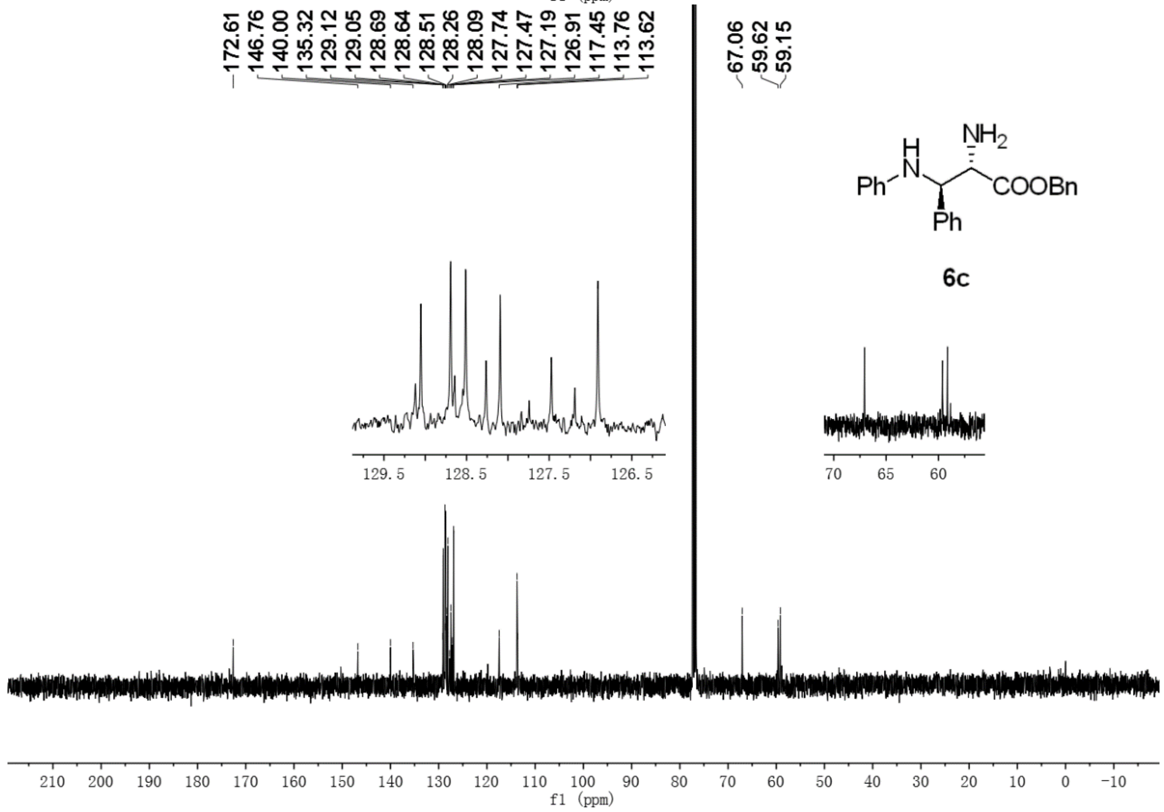

^{1}H NMR and $^{13}C\{^{1}H\}$ NMR spectra of compound of **7a** in $CDCl_3$

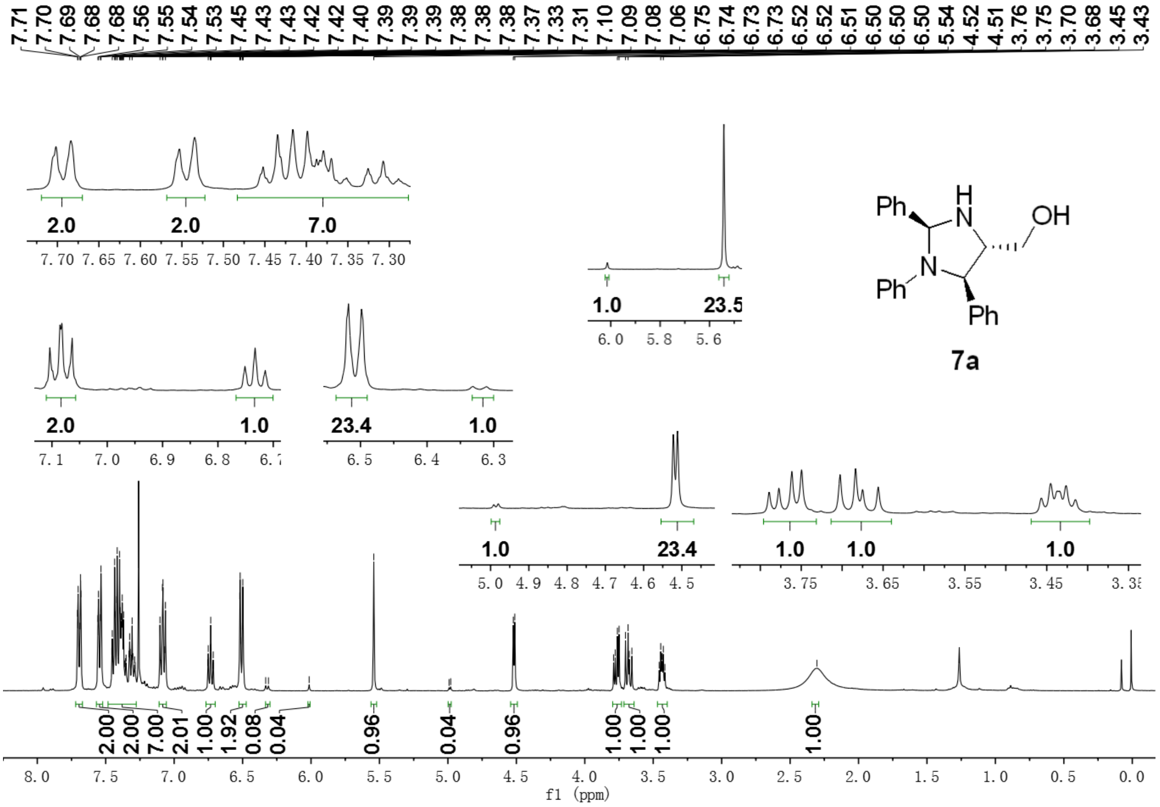

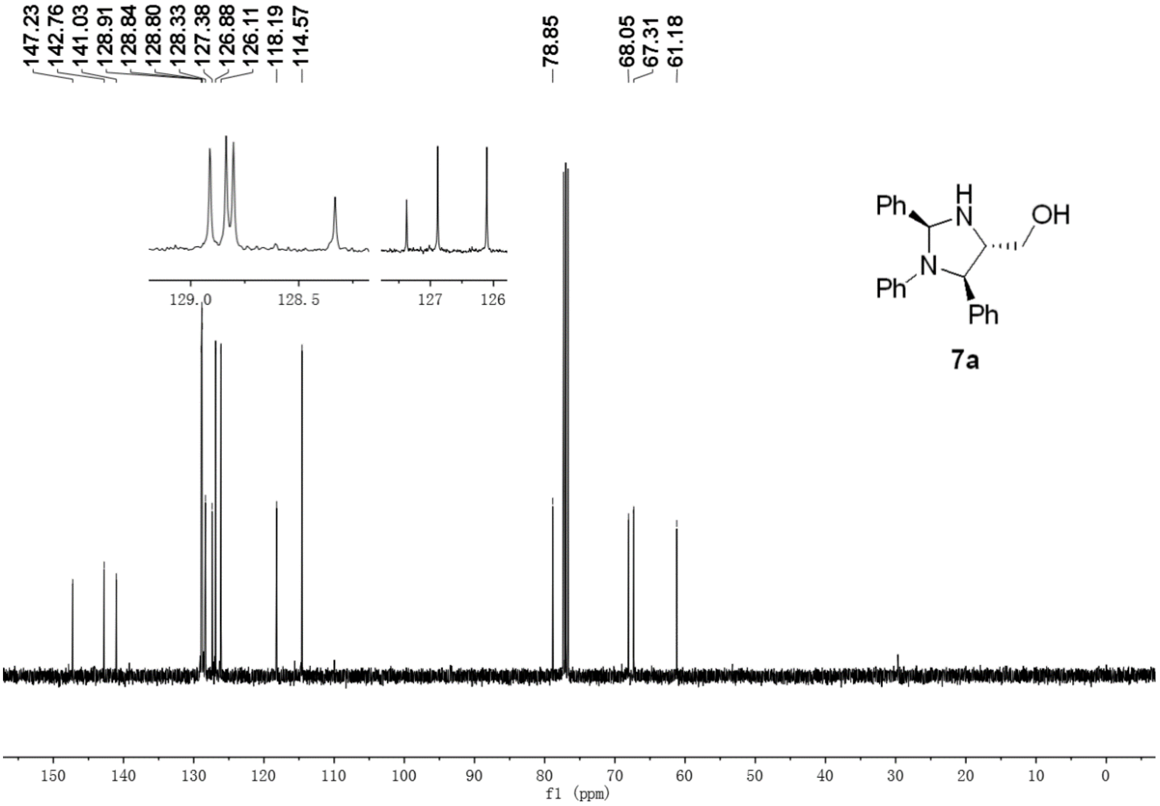

^{1}H NMR and ^{13}C{^{1}H} NMR spectra of compound of **8a** in $CDCl_3$

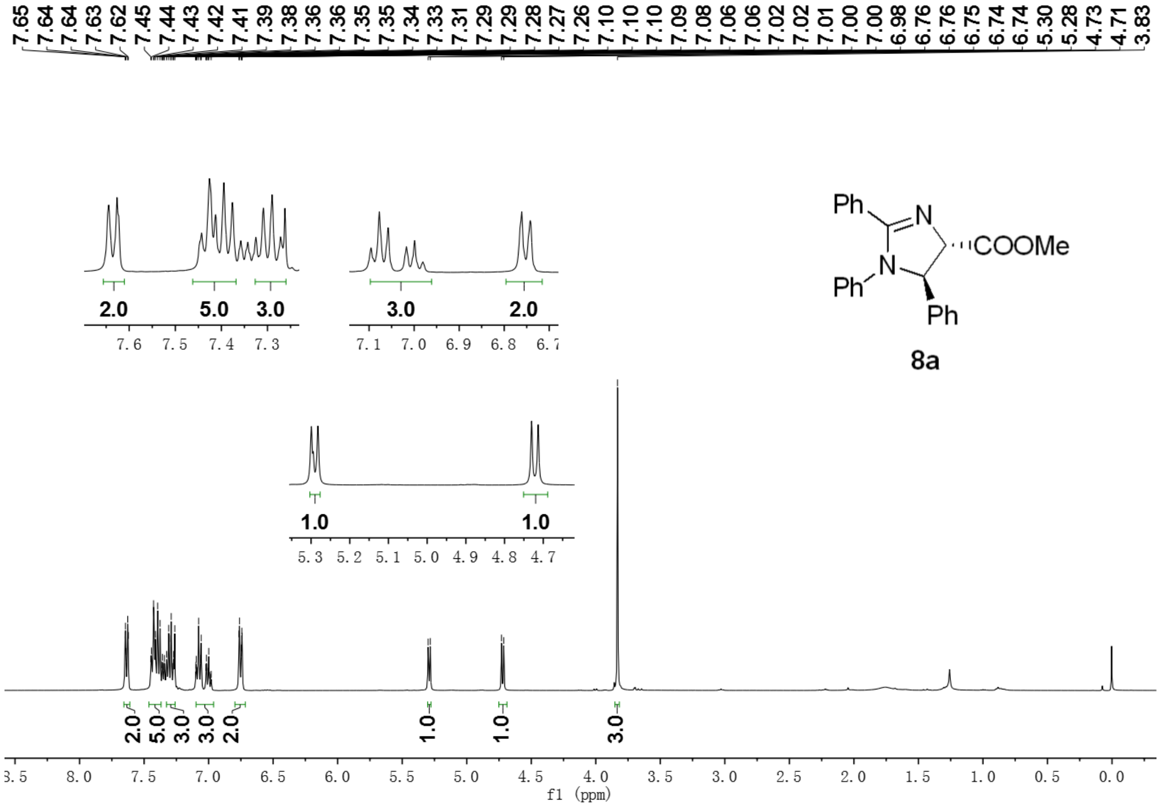

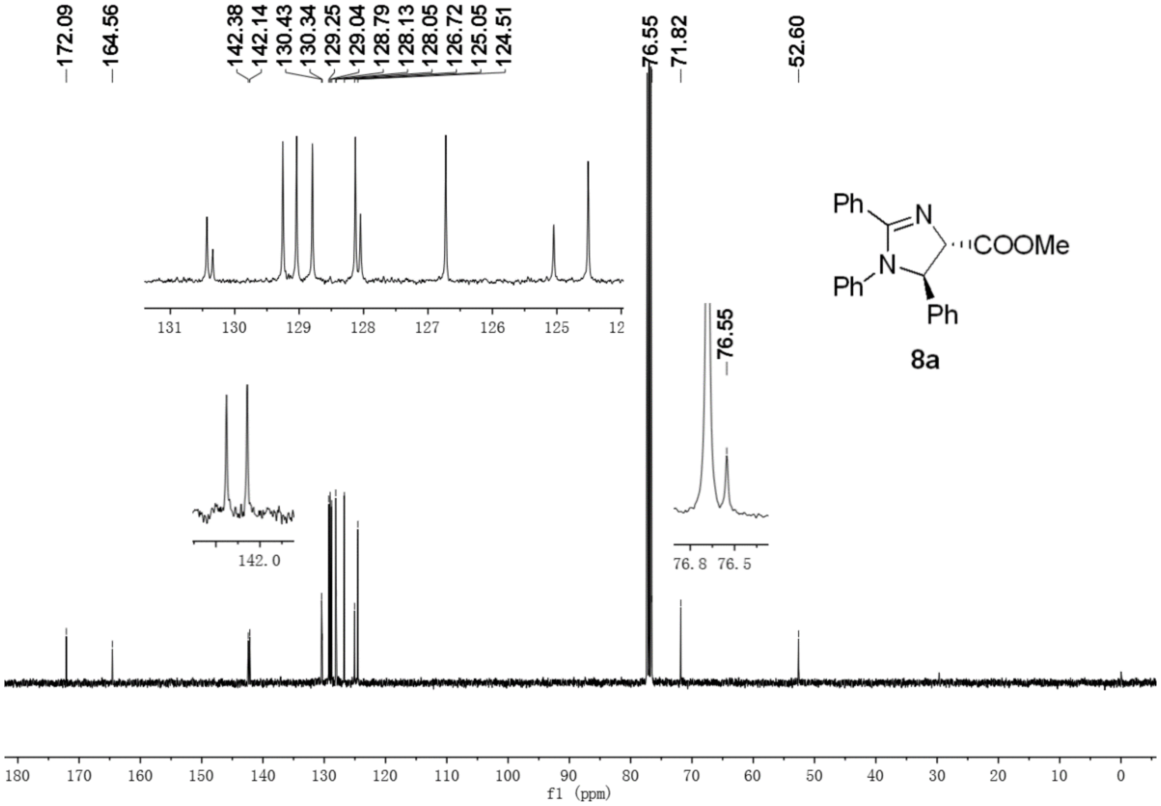

^{1}H NMR spectra of compound of **3a** in $CDCl_3$

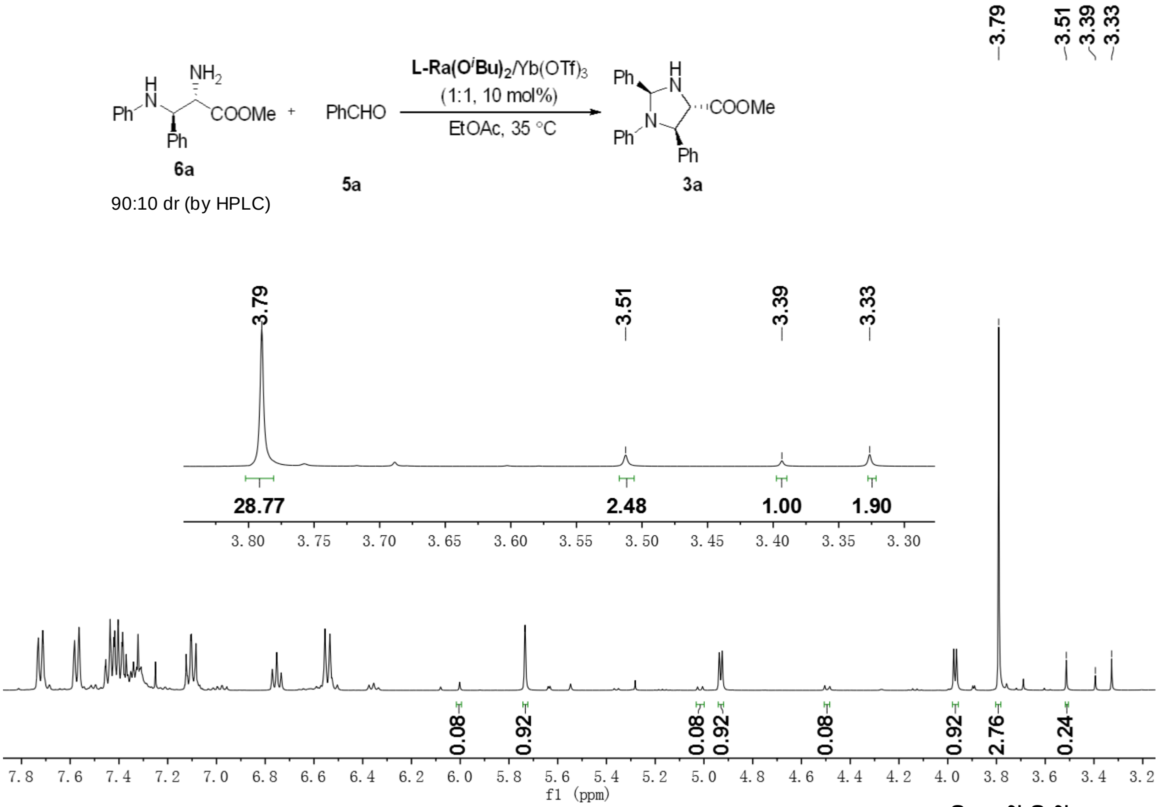

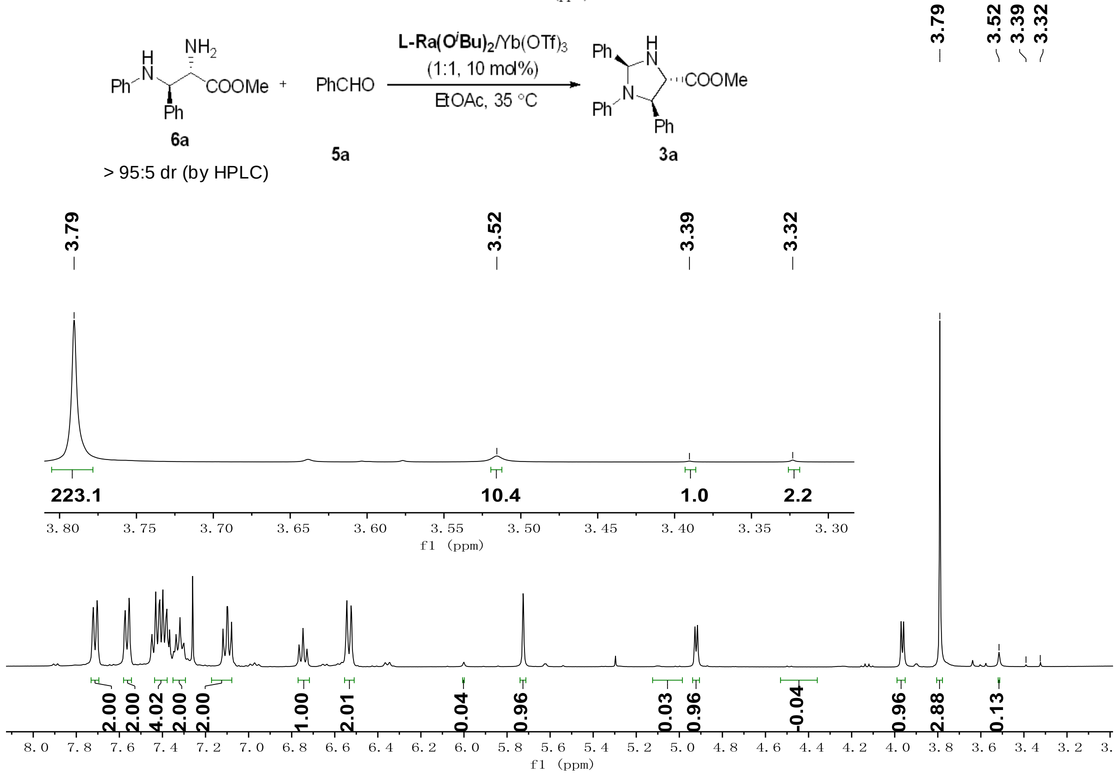

Different dr of **6a** (90:10 and >95:5) were reacted with **5a** to gain product **3a**. As ^{1}H NMR spectra showed, peak area of peak 3.39 ppm, peak 3.32 ppm were decreased with the increase of dr value. The results proposed that other isomers would produced during condensation step.

^{1}H NMR and $^{13}C\{^{1}H\}$ NMR spectra of compound of **3a** (procedure A) in $CDCl_3$

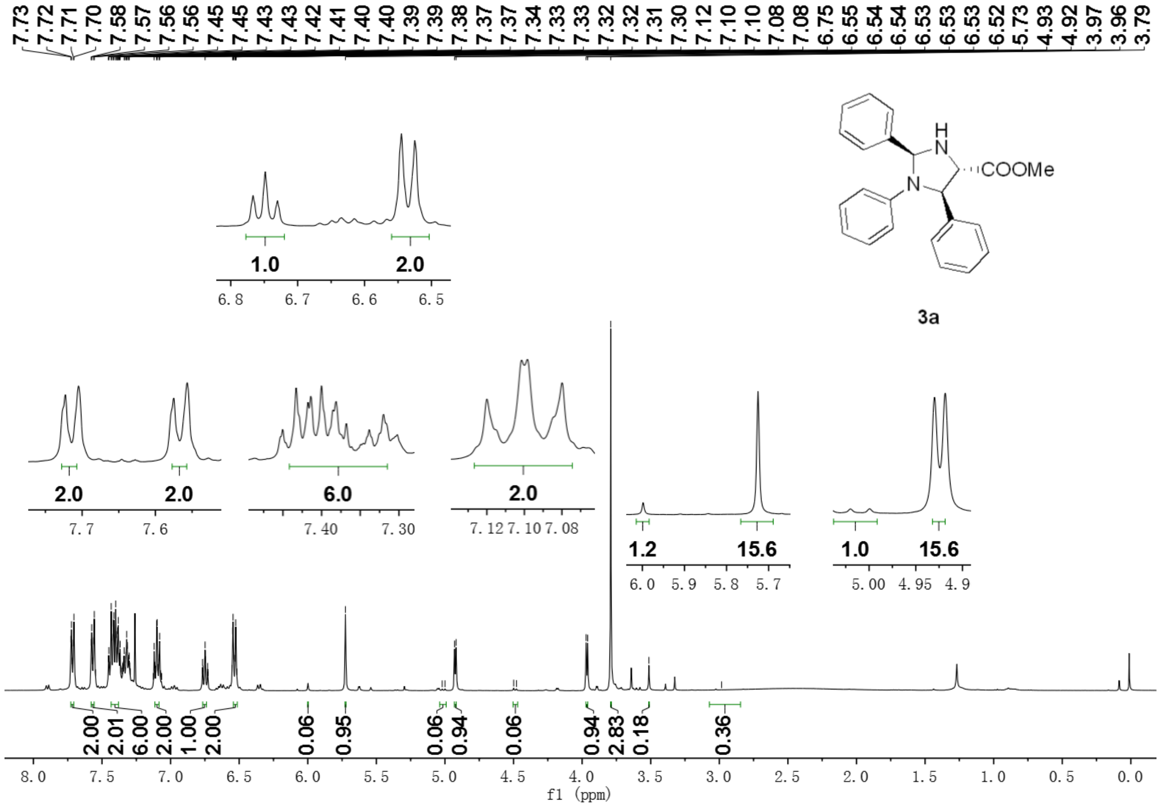

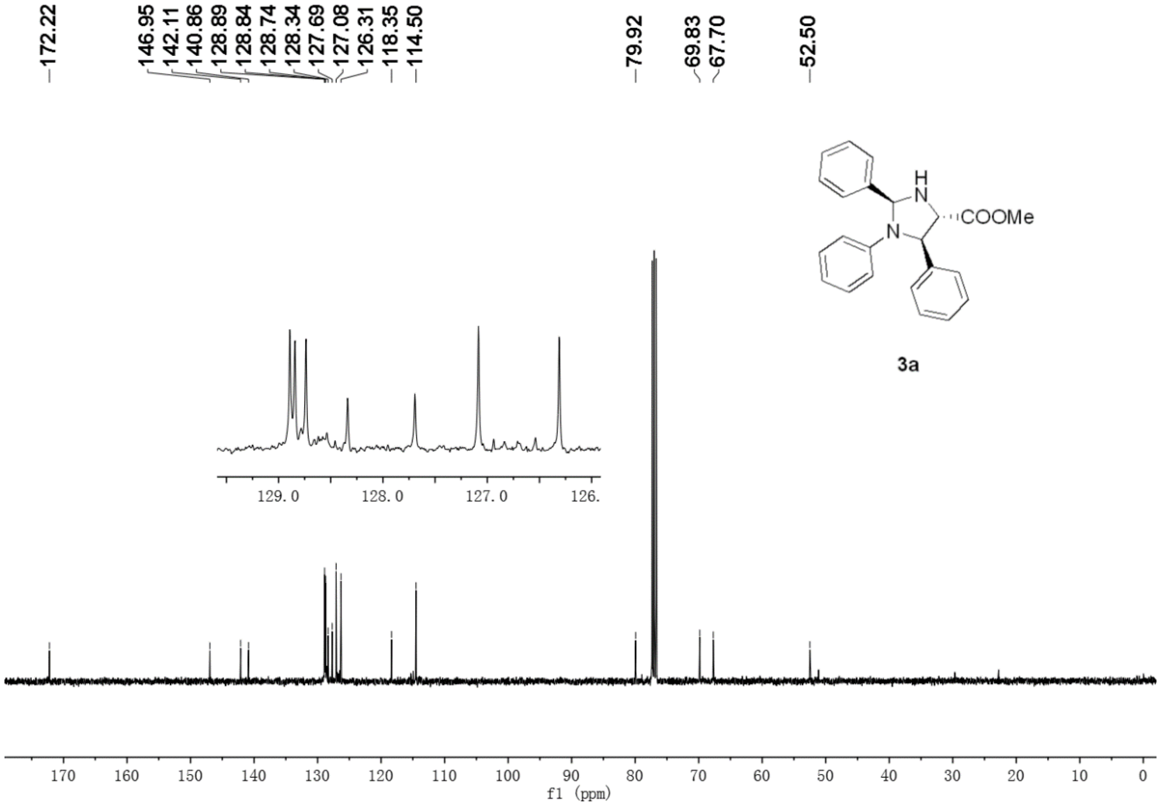

^{1}H NMR and $^{13}C\{^{1}H\}$ NMR spectra of compound of **3a** (procedure B) in $CDCl_3$

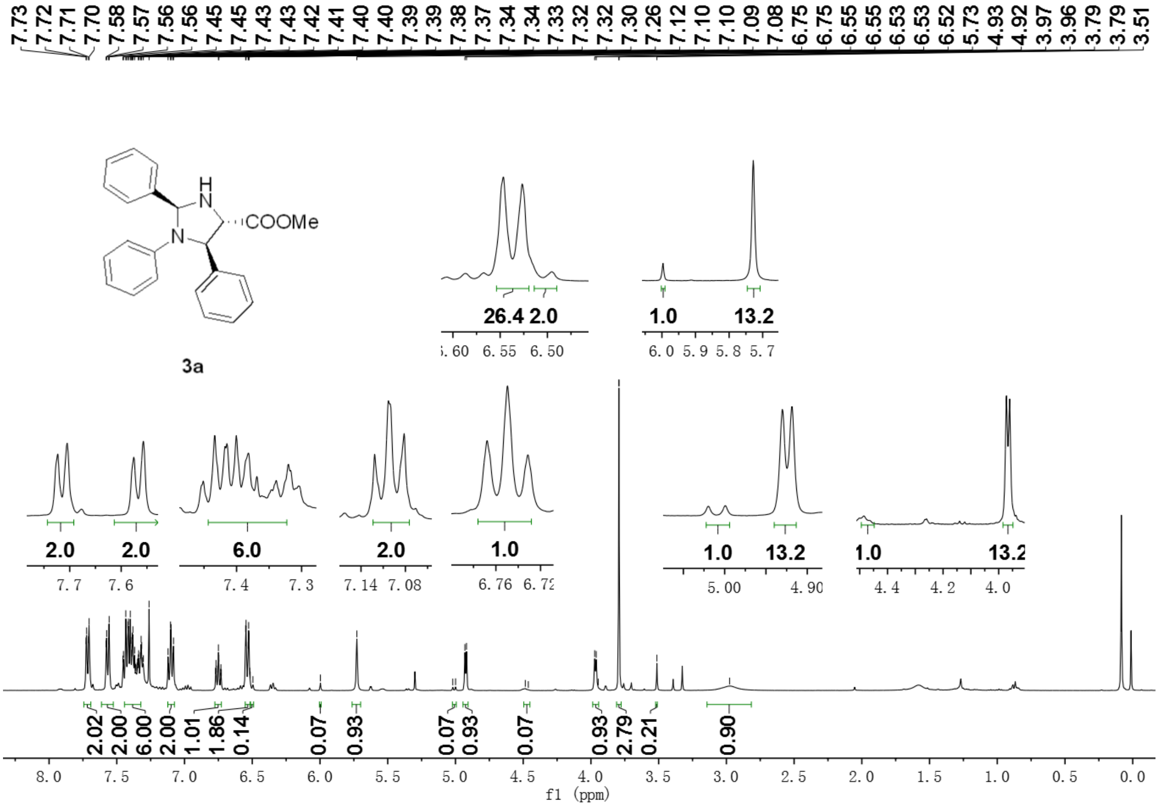

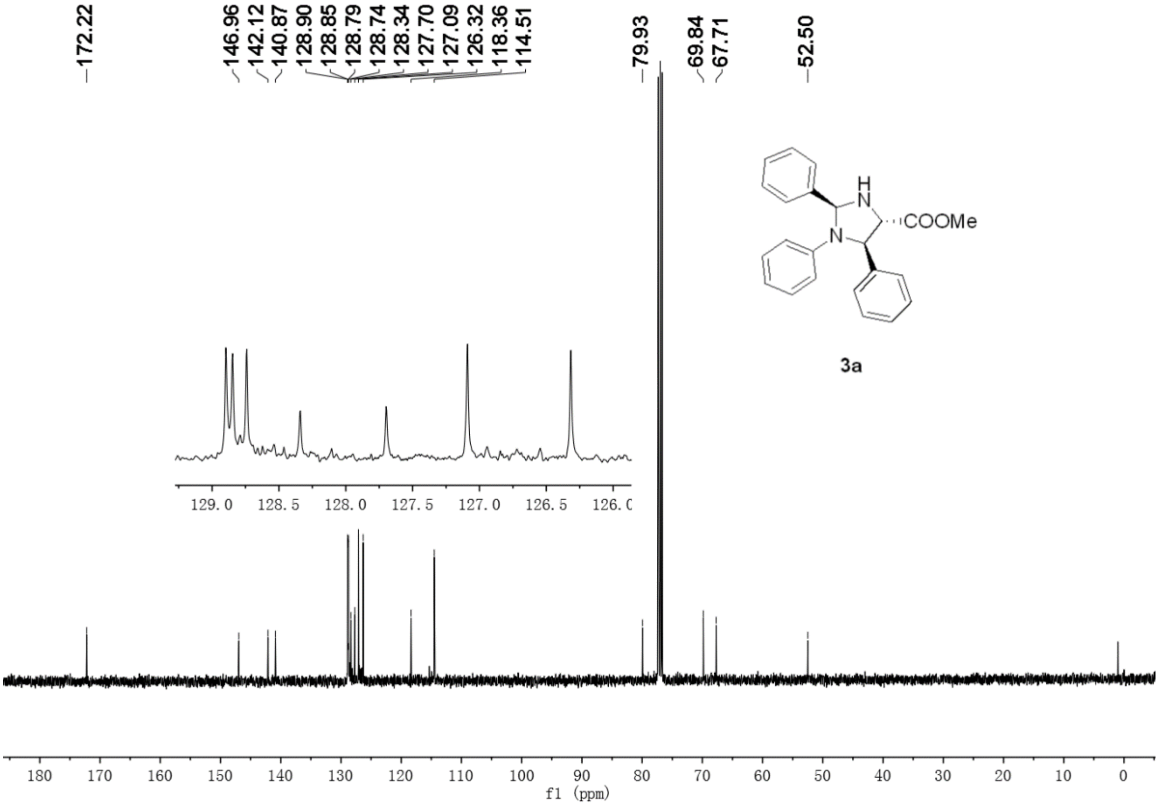

^{1}H NMR and $^{13}C\{^{1}H\}$ NMR spectra of compound of (2*R*,4*R*,5*S*)-**3a** (procedure A) in $CDCl_3$

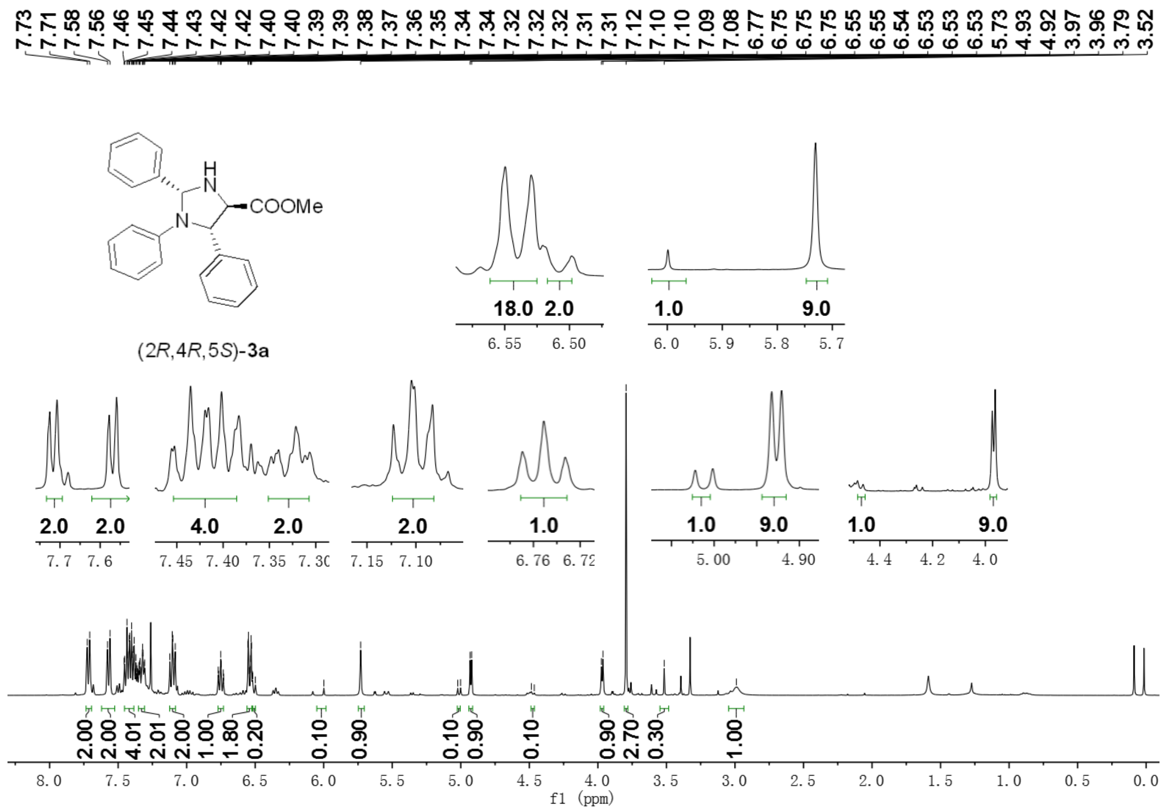

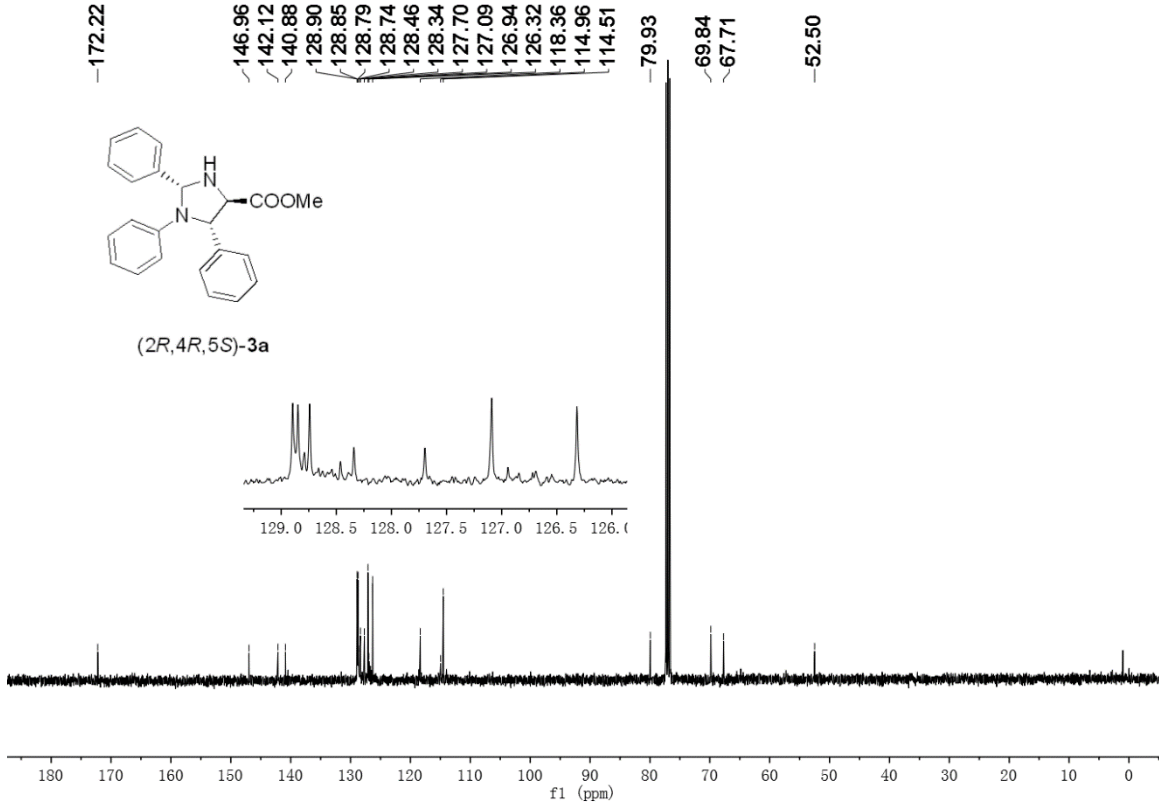

^{1}H NMR and ^{13}C{^{1}H} NMR spectra of compound of **3b** (procedure A) in $CDCl_3$

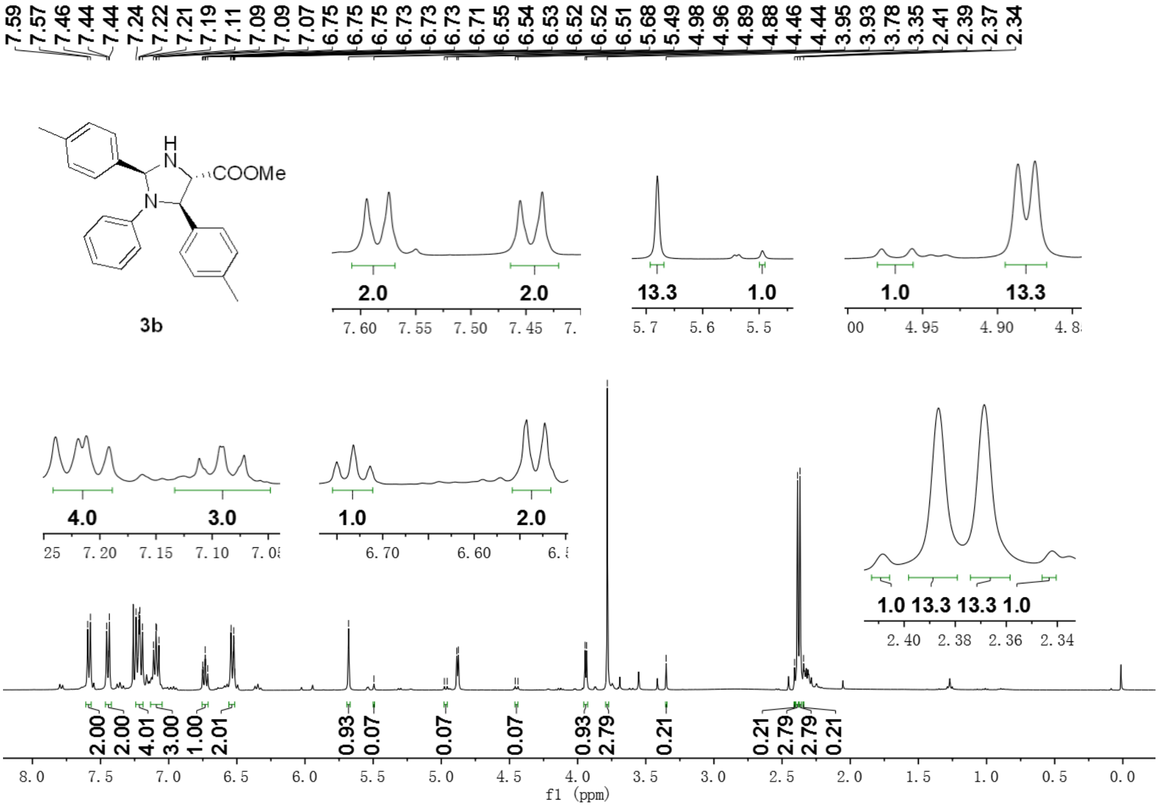

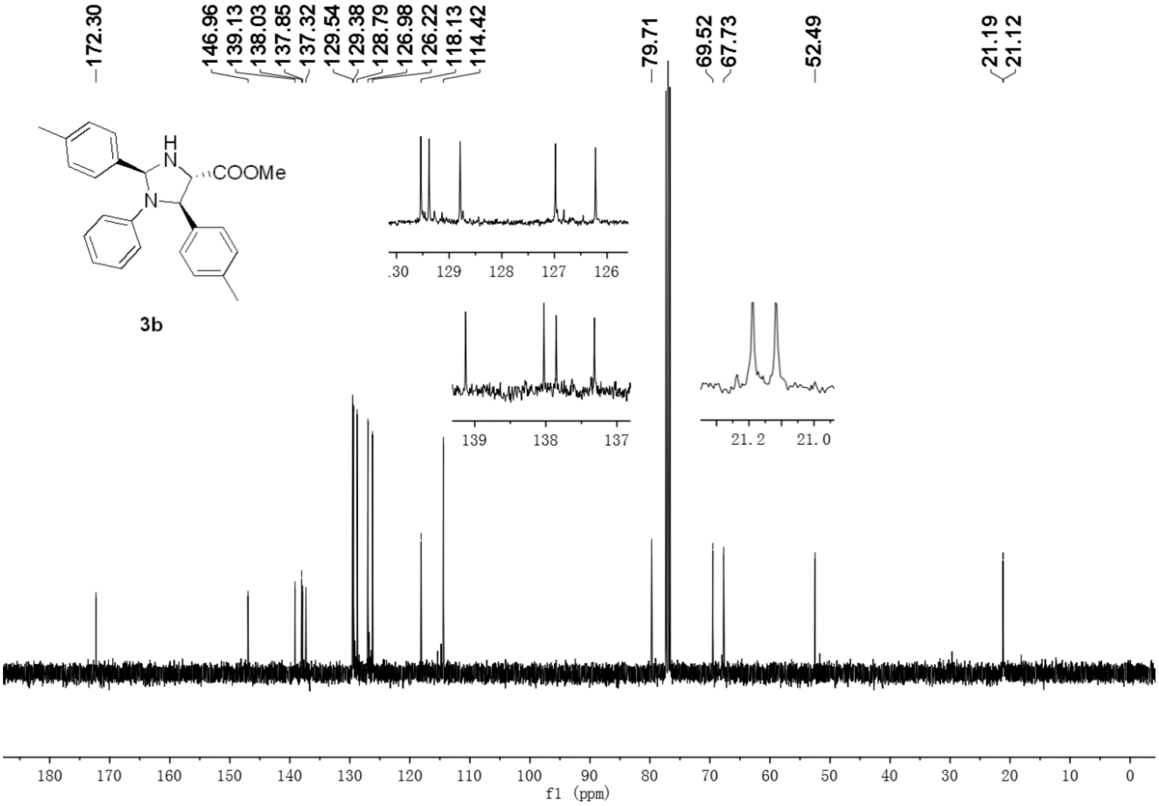

^{1}H NMR and ^{13}C{^{1}H} NMR spectra of compound of **3b** (procedure B) in $CDCl_3$

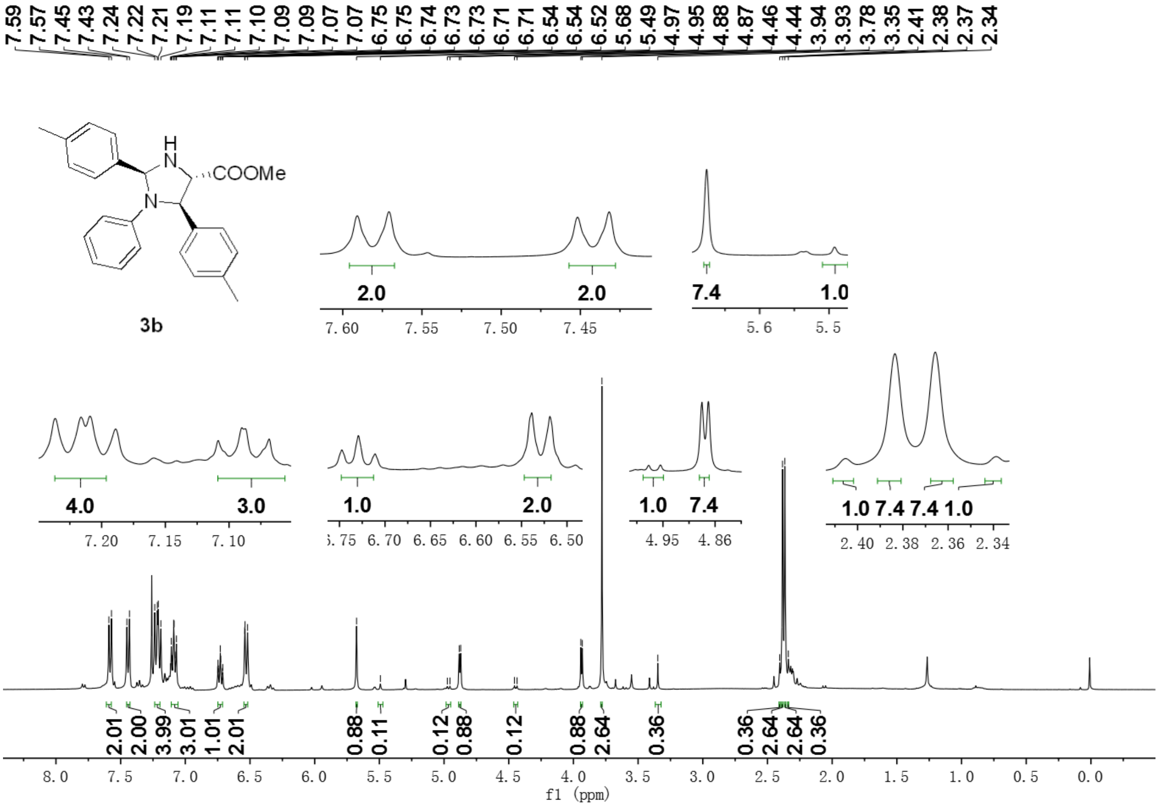

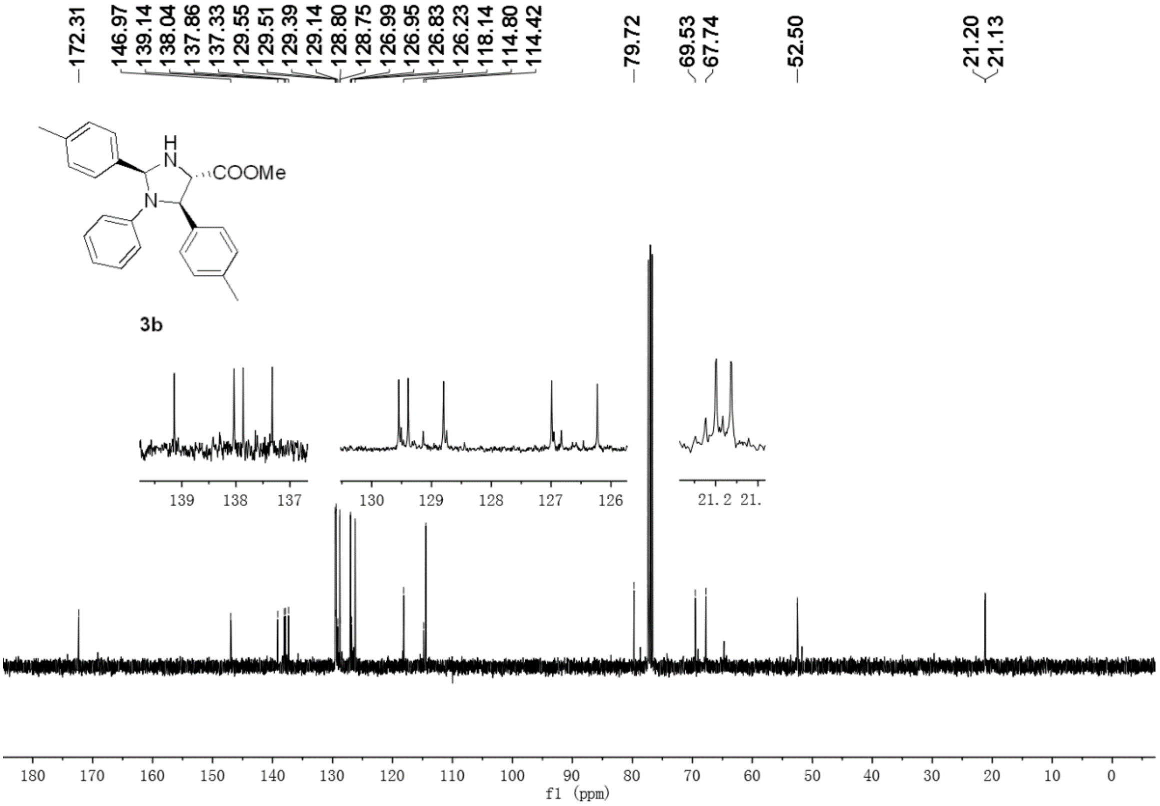

^{1}H NMR and ^{13}C{^{1}H} NMR spectra of compound of **3c** (procedure A) in $CDCl_3$

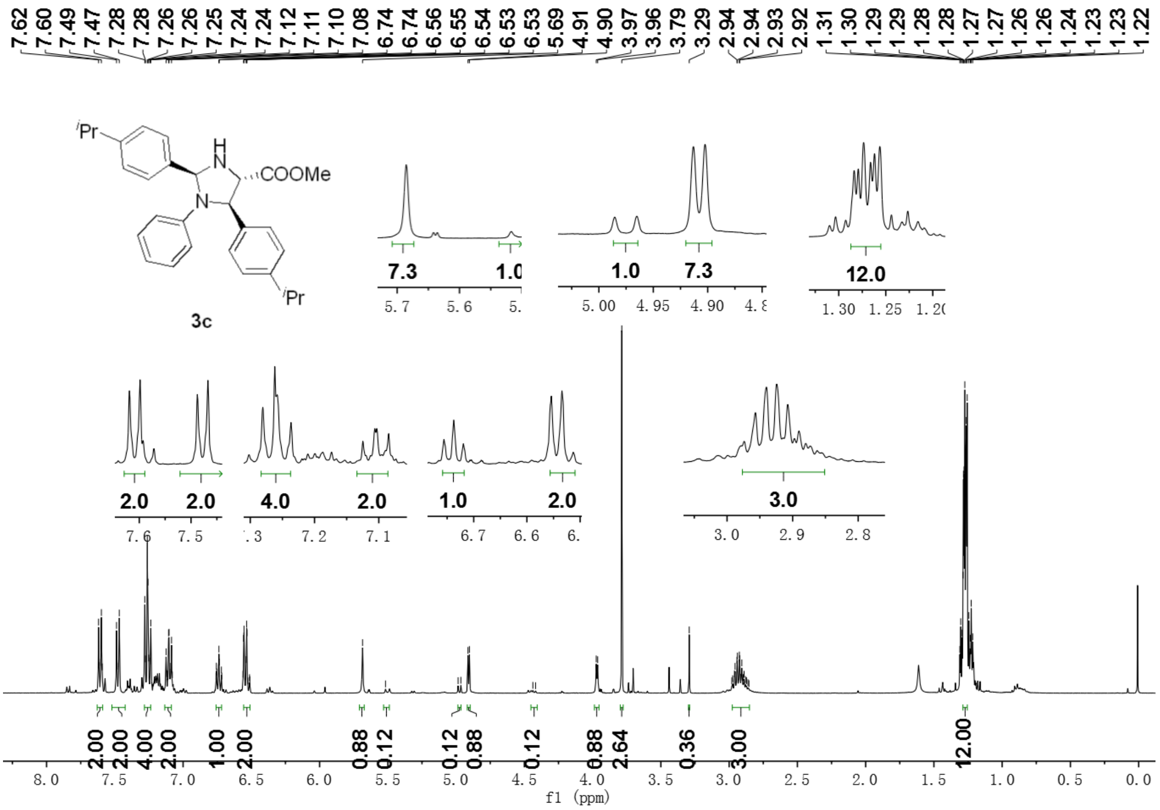

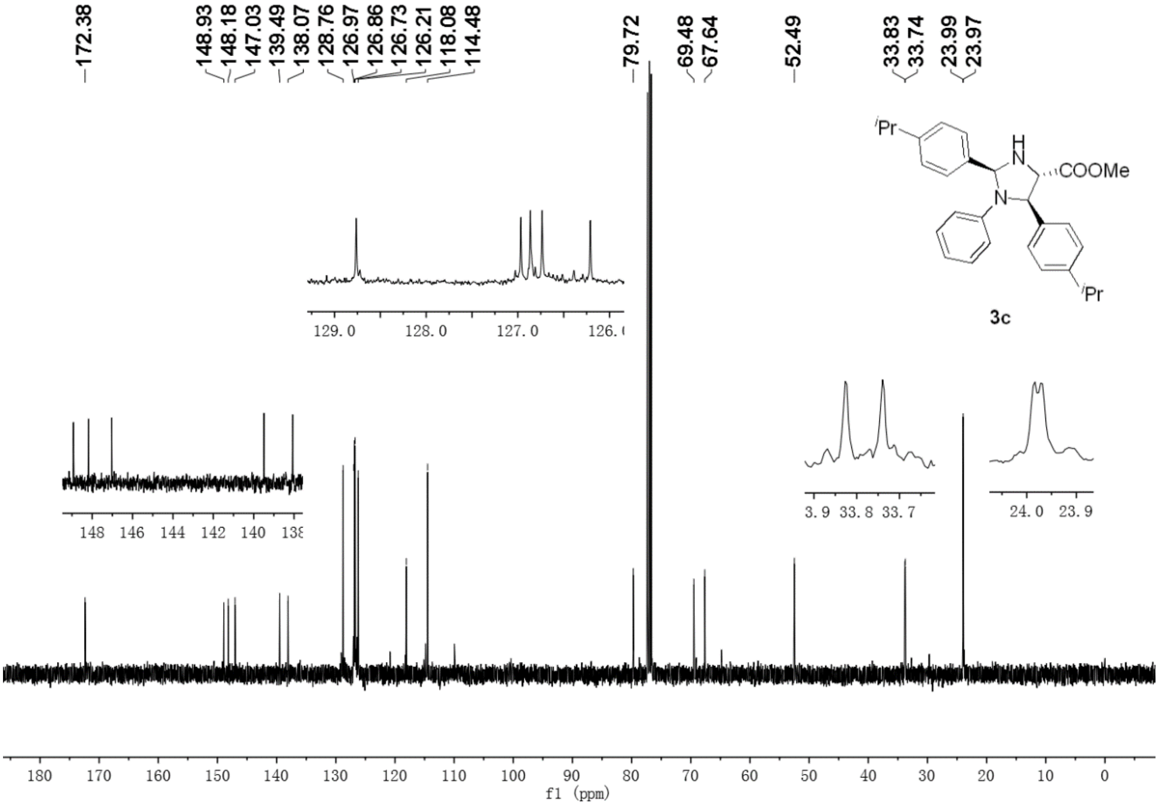

^{1}H NMR and ^{13}C{^{1}H} NMR spectra of compound of **3d** (procedure A) in $CDCl_3$

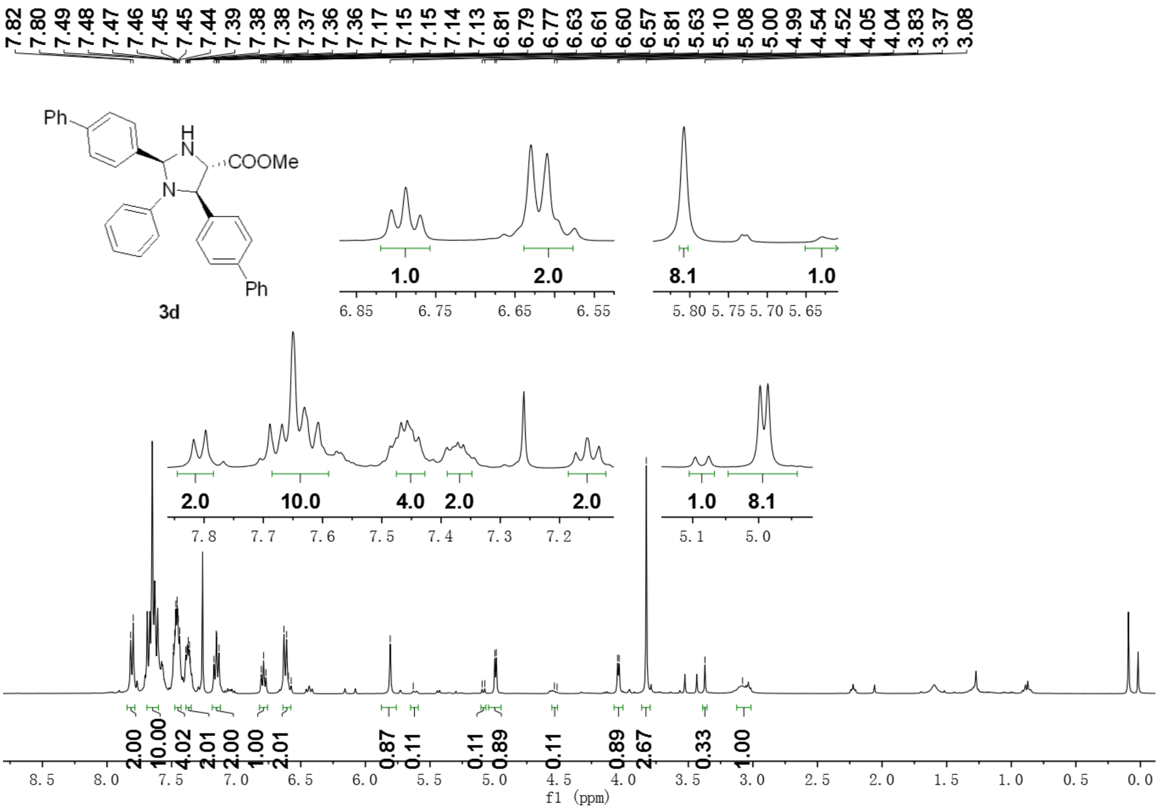

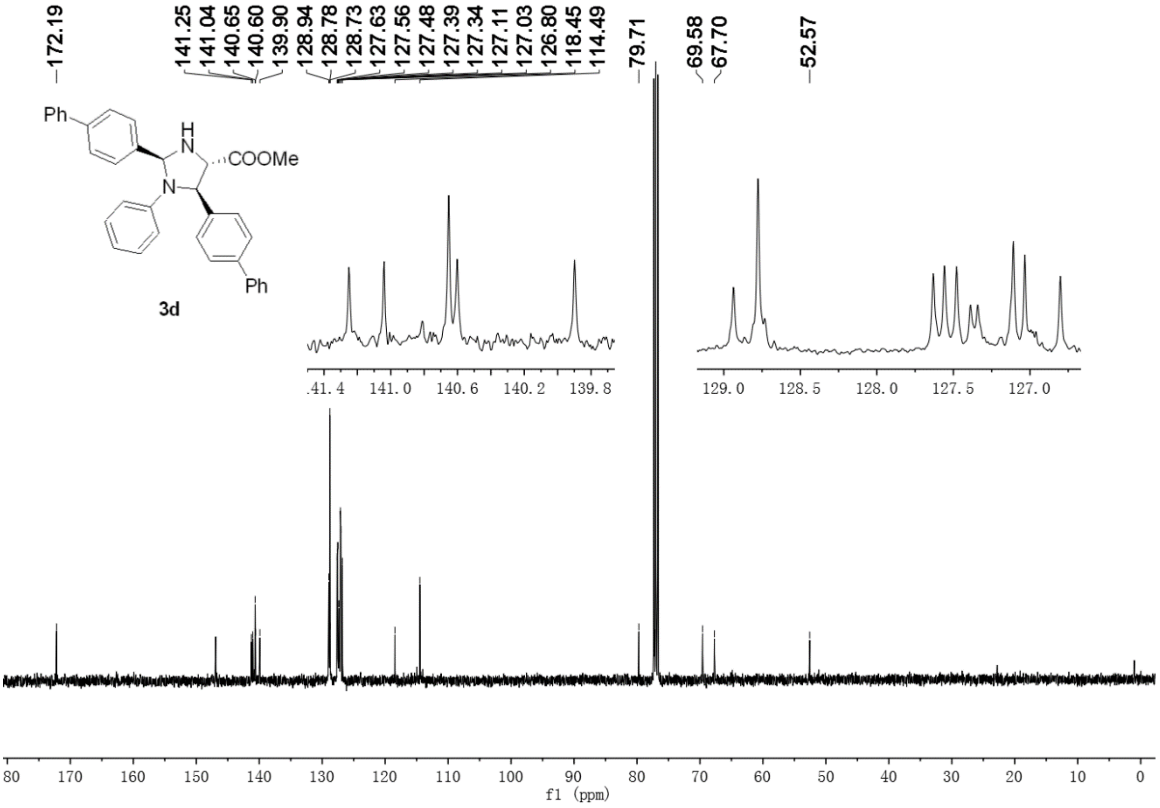

^{1}H NMR and ^{13}C{^{1}H} NMR spectra of compound of (2*R*,4*R*,5*S*)-**3d** (procedure A) in $CDCl_3$

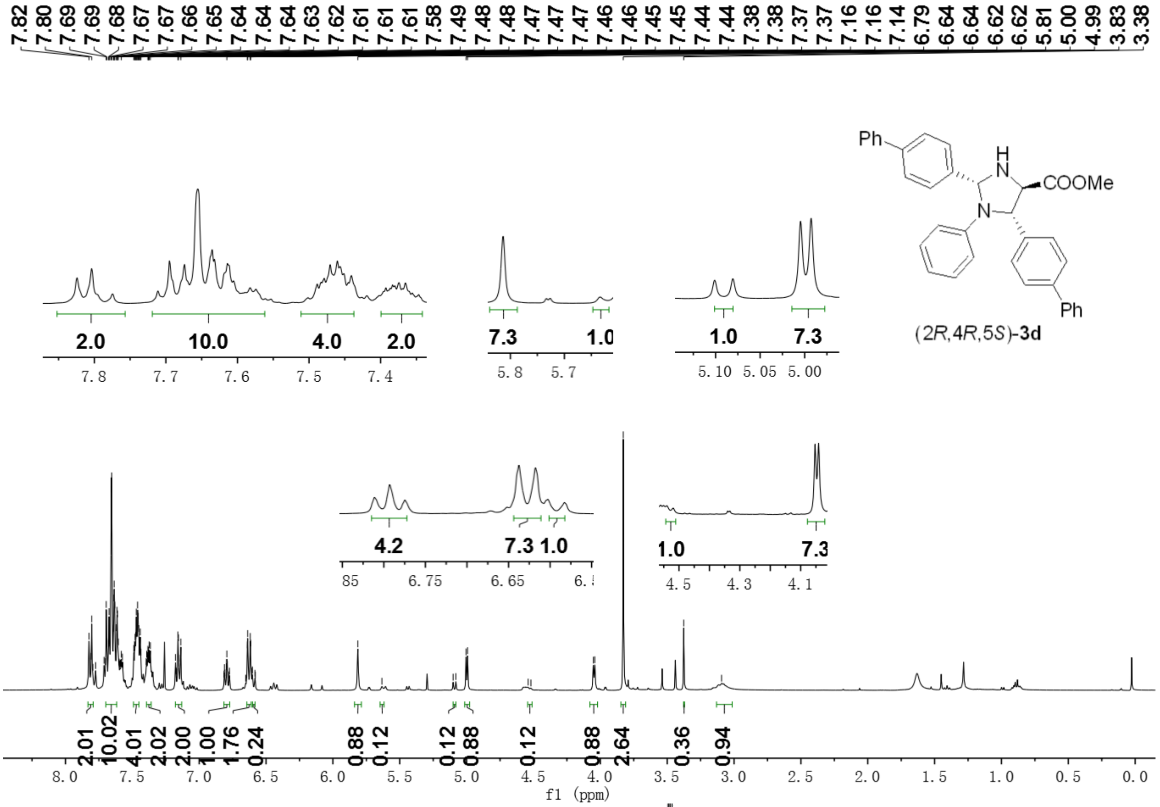

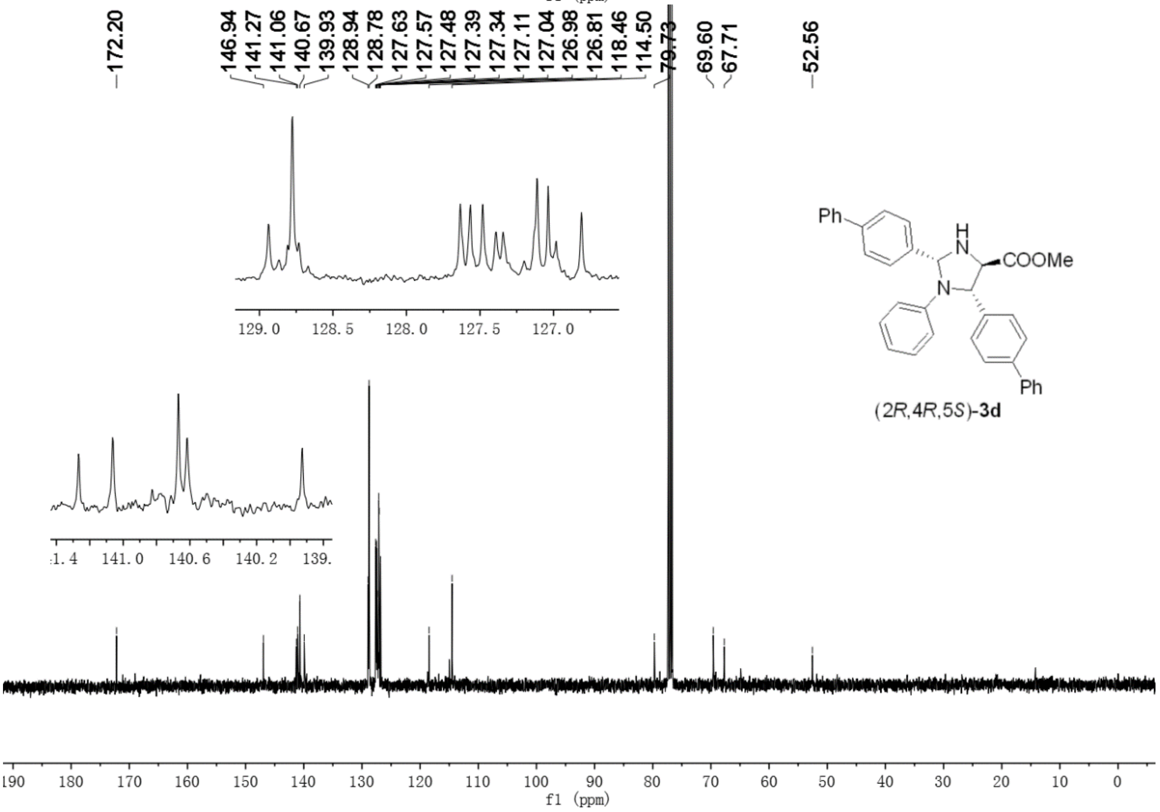

^{1}H NMR and $^{13}C\{^{1}H\}$ NMR spectra of compound of **3e** (procedure A) in $CDCl_3$

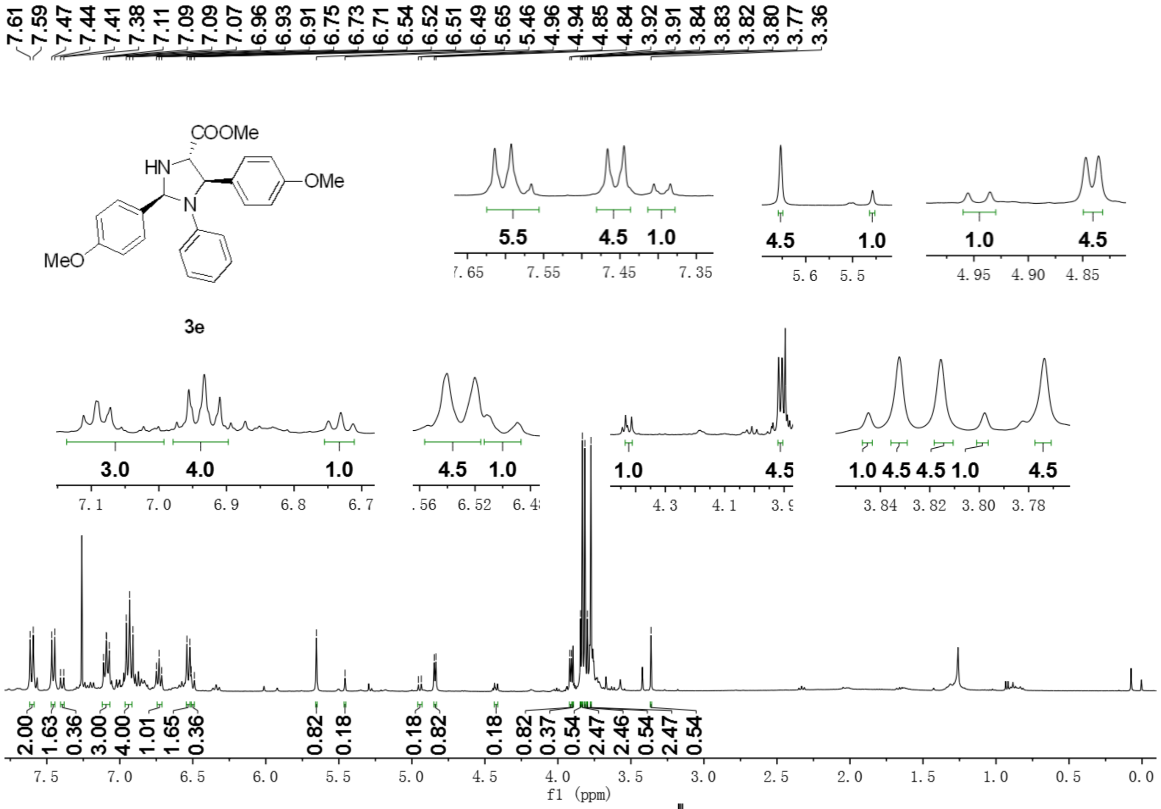

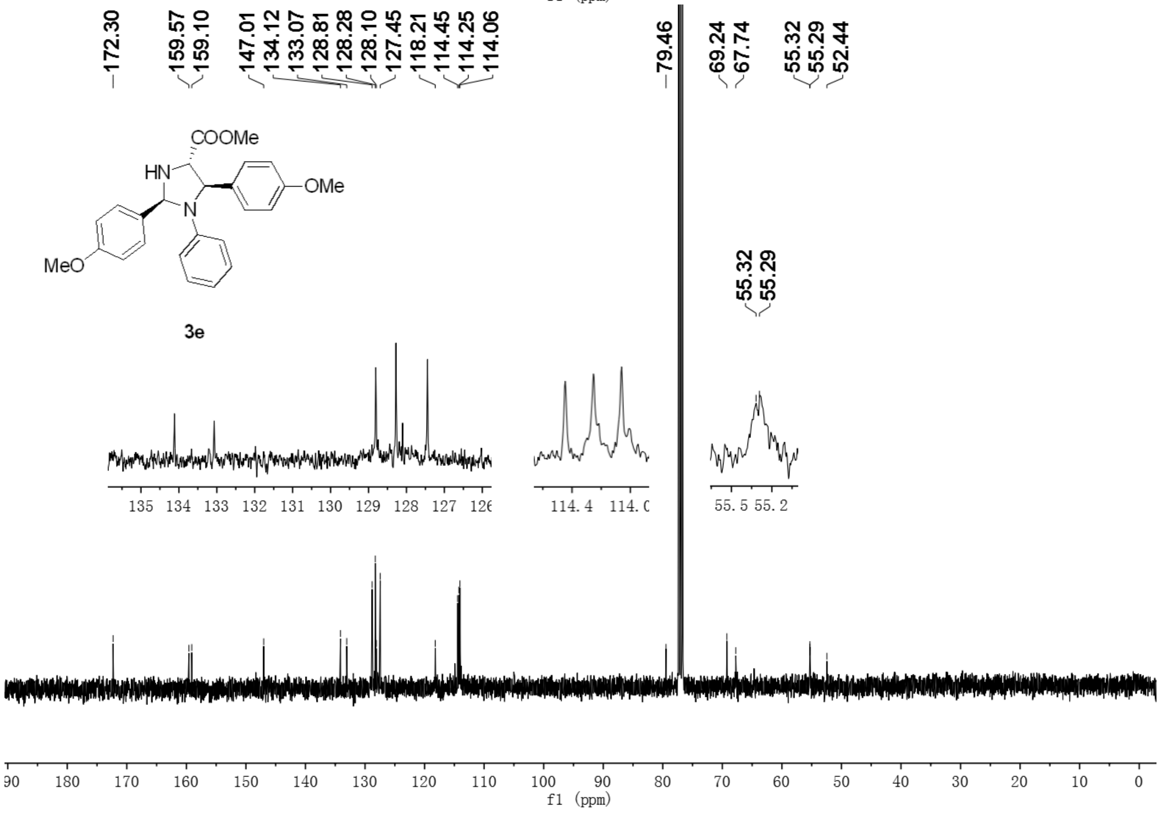

^{1}H NMR and ^{13}C{^{1}H} NMR spectra of compound of **3f** (procedure A) in $CDCl_3$

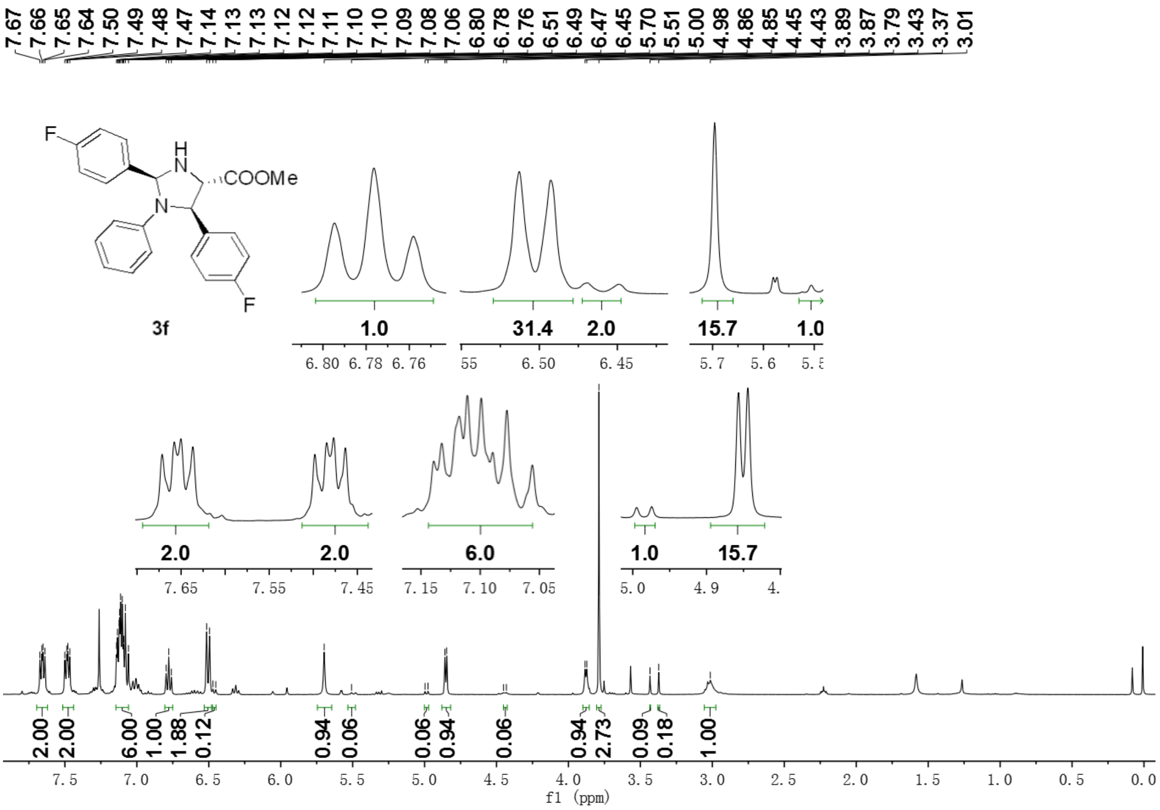

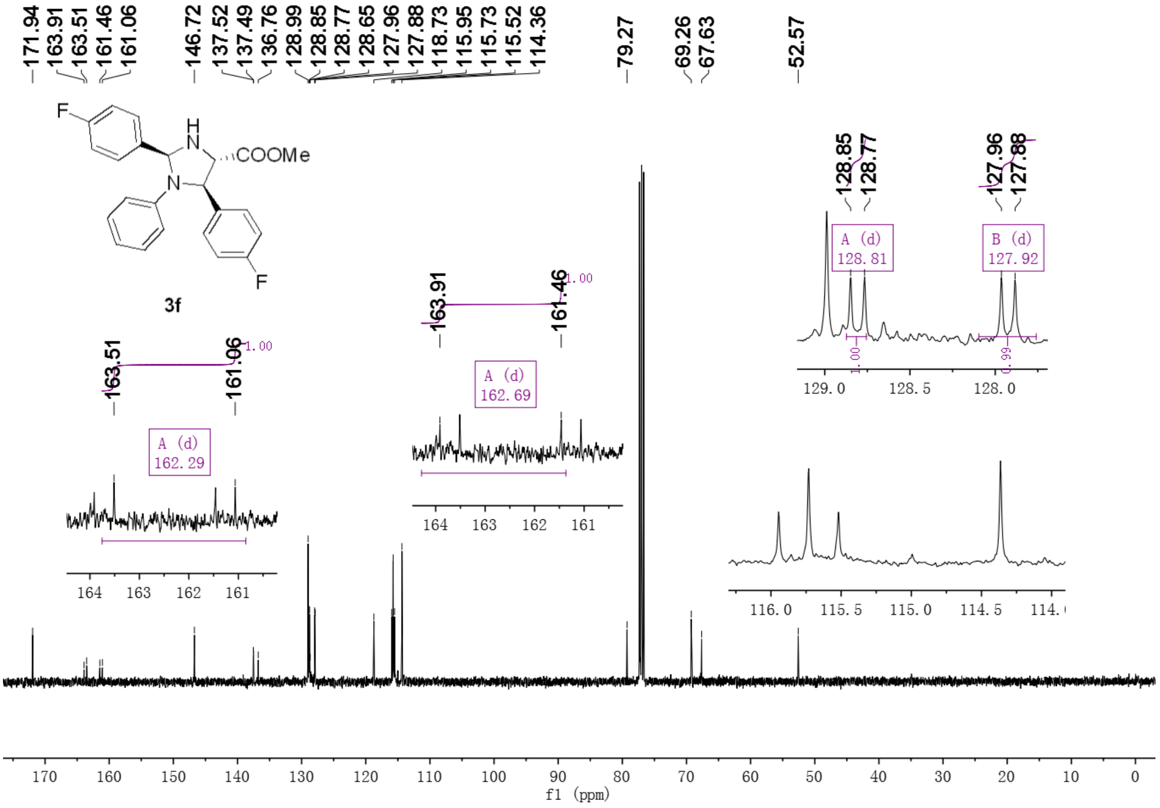

$^{19}F\{^{1}H\}$ NMR spectra of compound of **3f** (procedure A) in $CDCl_3$

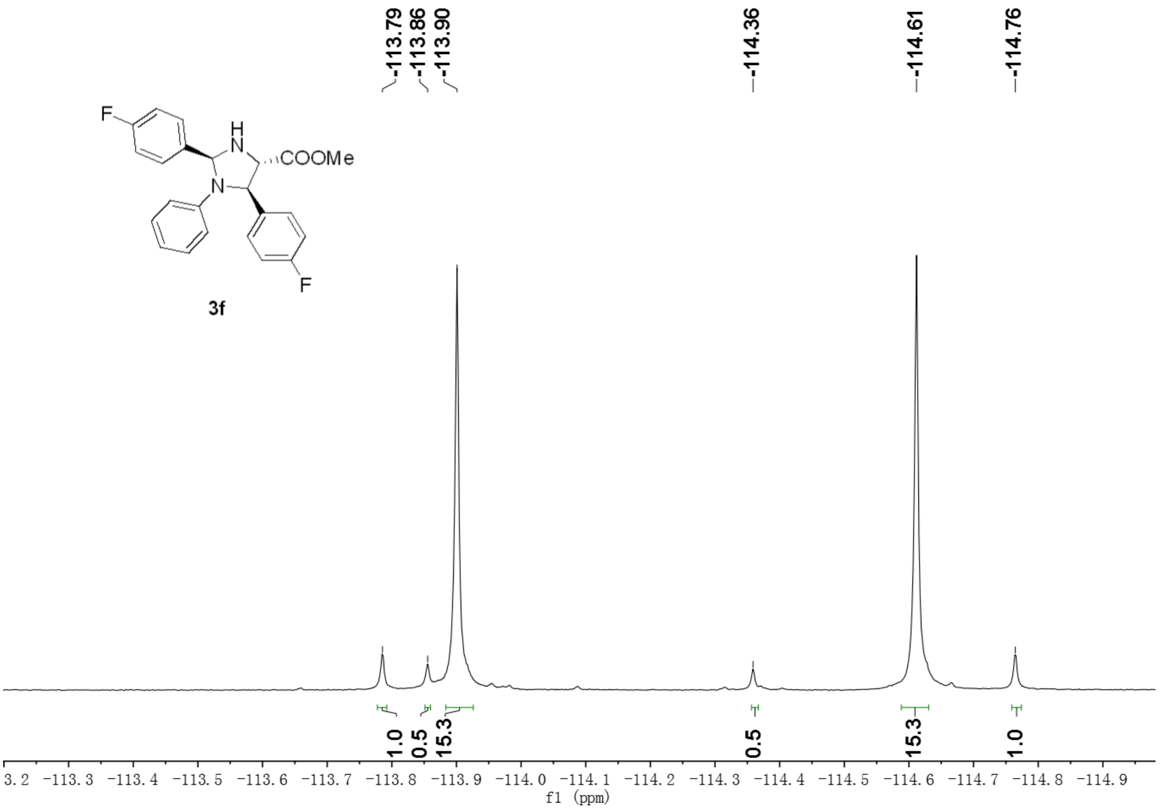

^{1}H NMR and $^{13}C\{^{1}H\}$ NMR spectra of compound of **3g** (procedure A) in $CDCl_3$

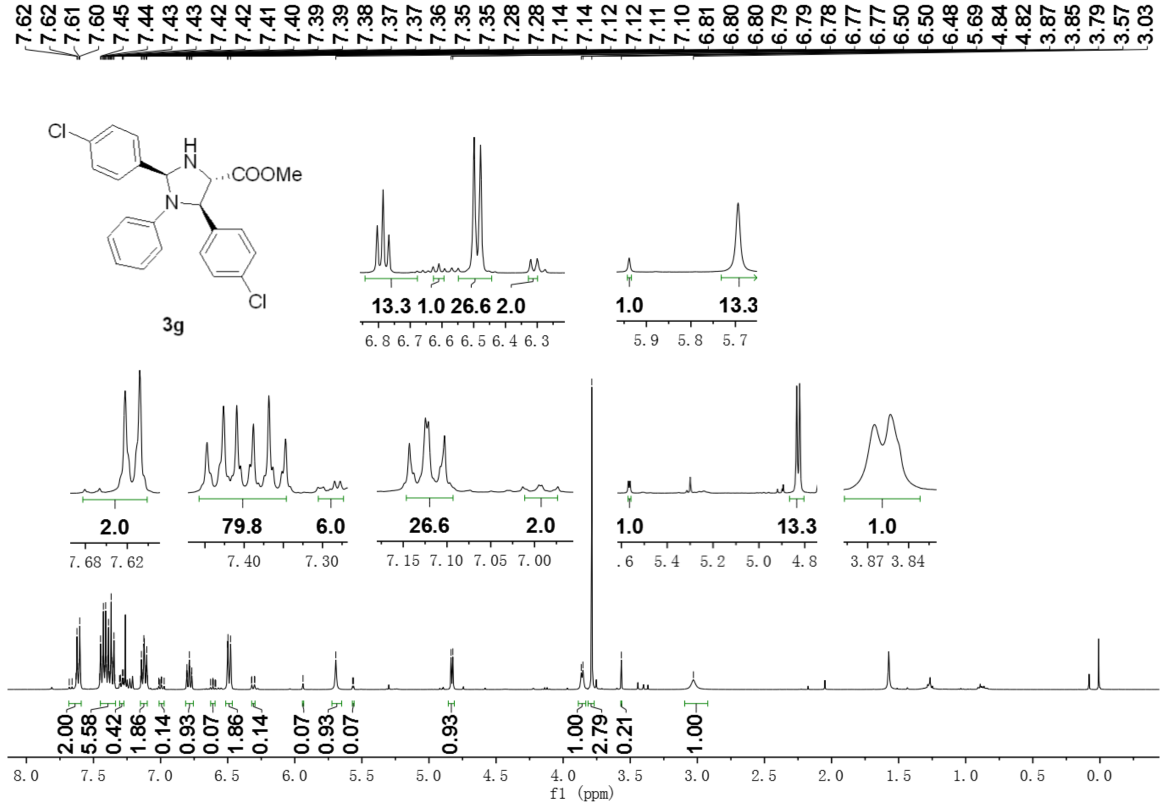

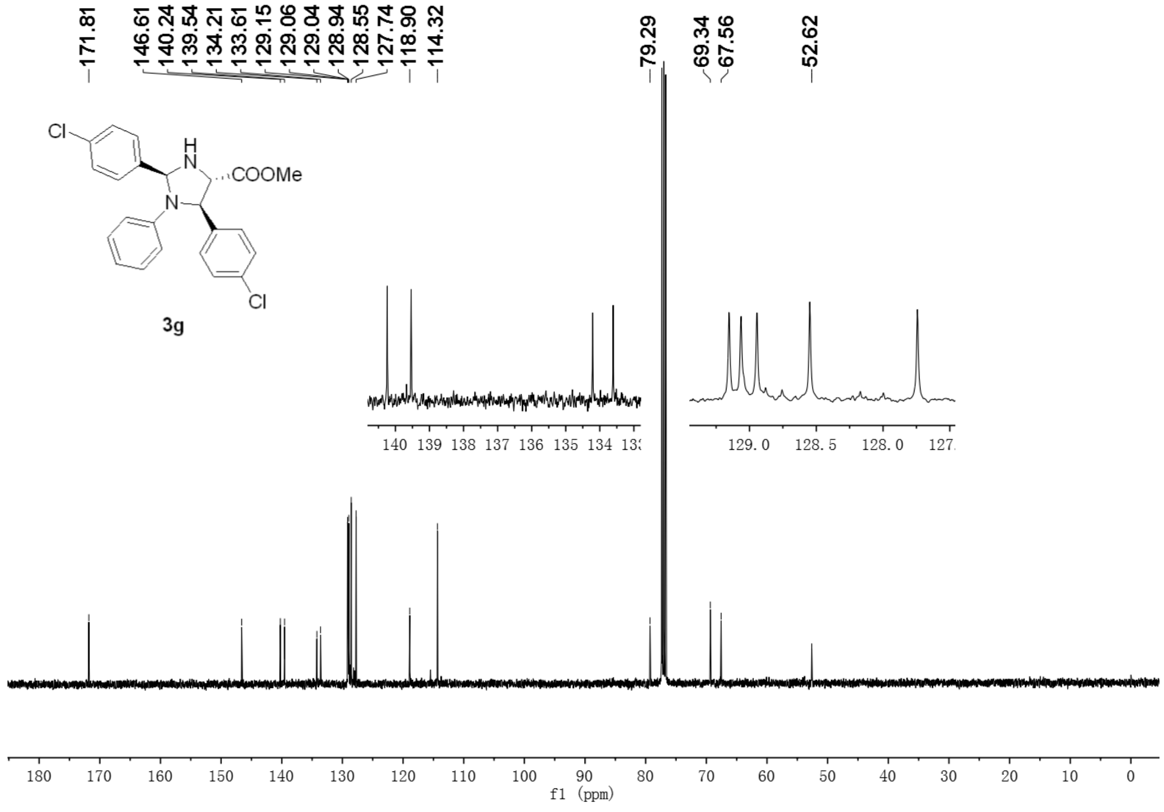

^{1}H NMR and $^{13}C\{^{1}H\}$ NMR spectra of compound of **3g** (procedure B) in $CDCl_3$

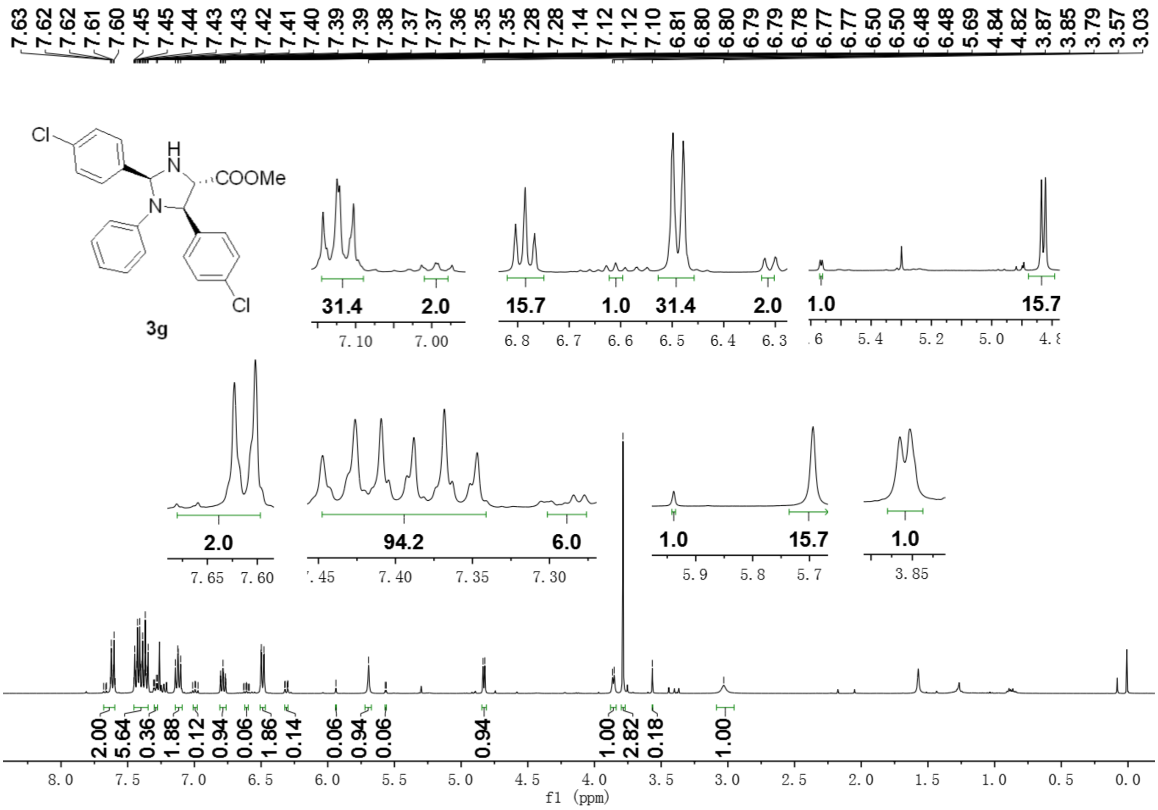

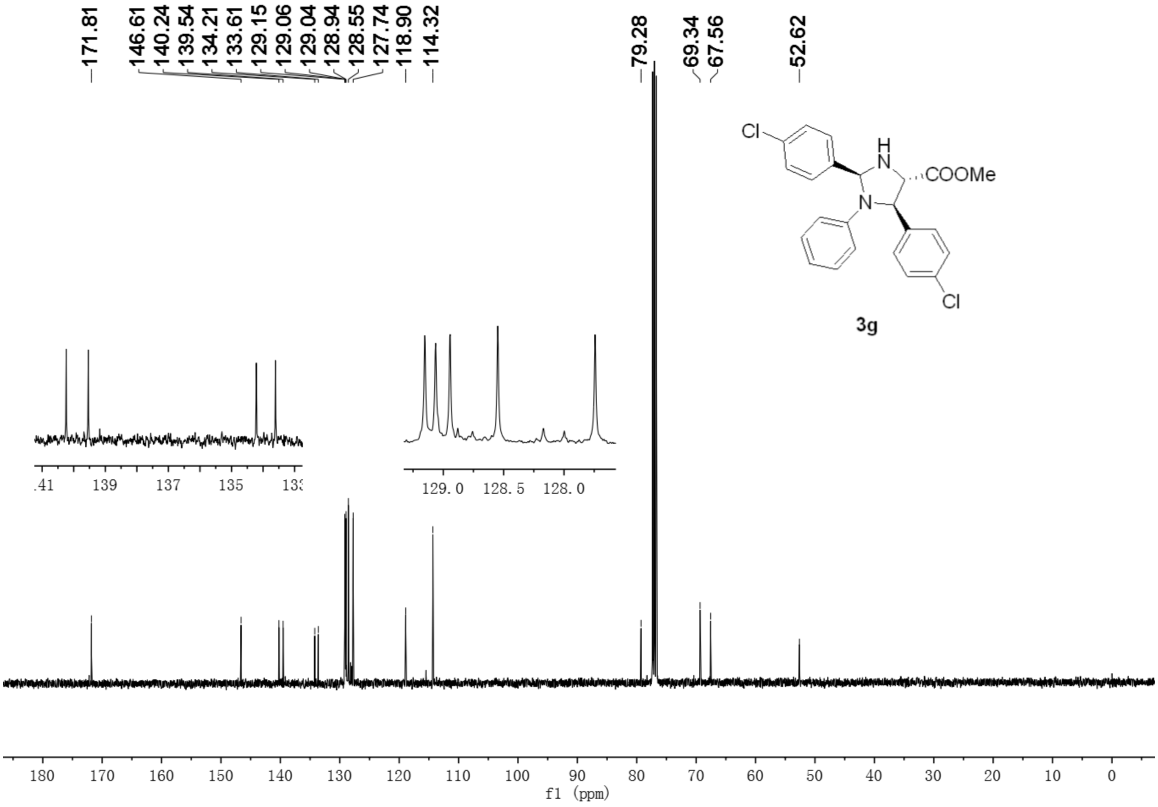

^{1}H NMR and $^{13}C\{^{1}H\}$ NMR spectra of compound of **3h** (procedure A) in $CDCl_3$

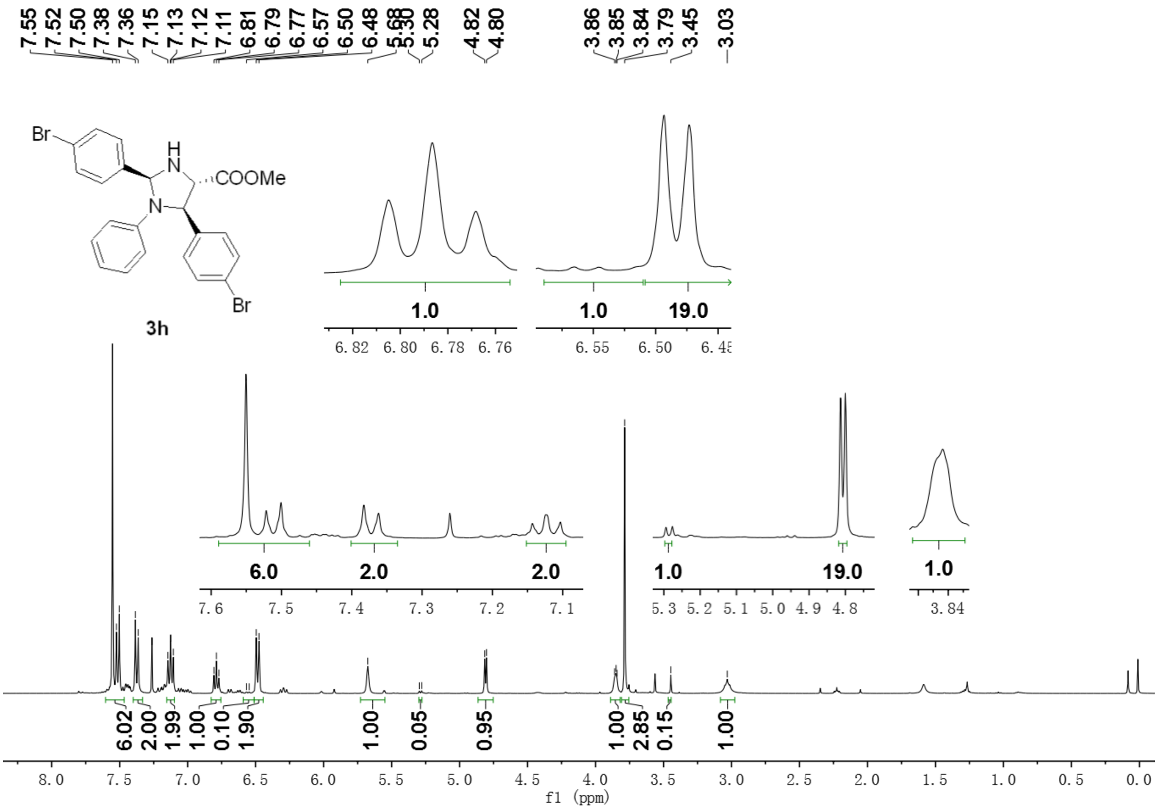

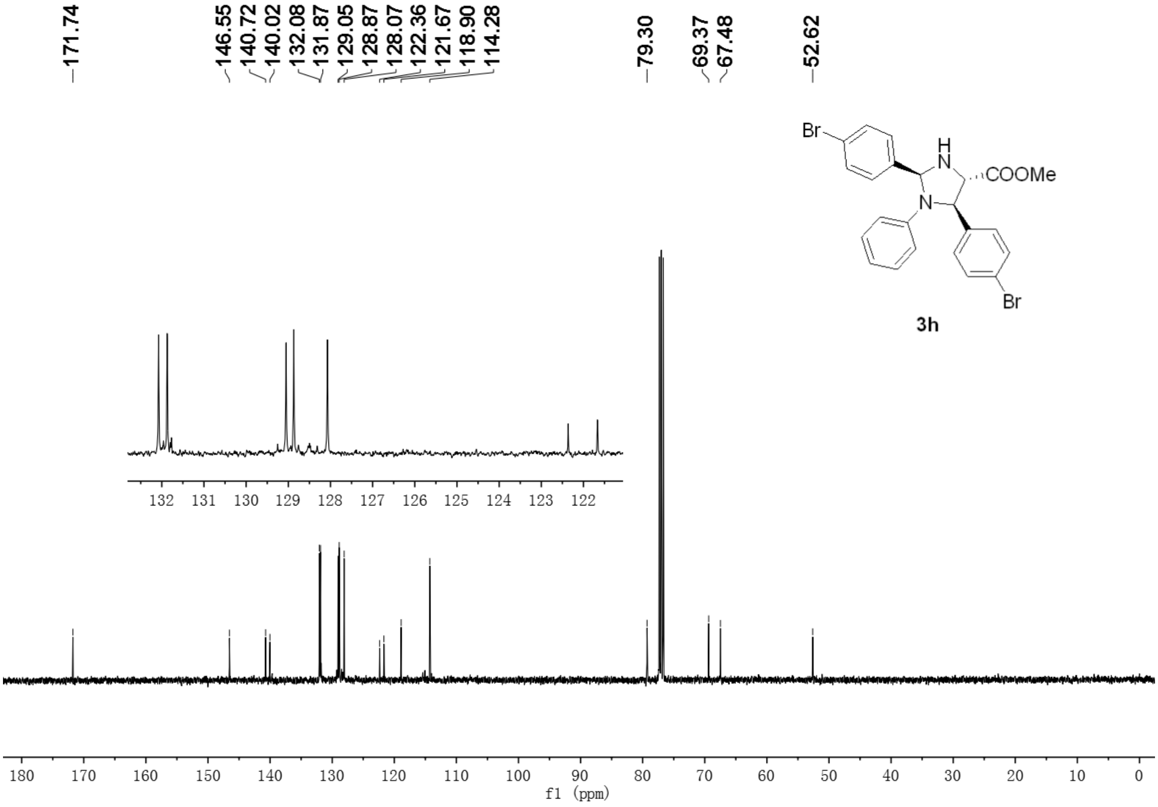

^{1}H NMR and ^{13}C{^{1}H} NMR spectra of compound of **3i** (procedure A) in $CDCl_3$

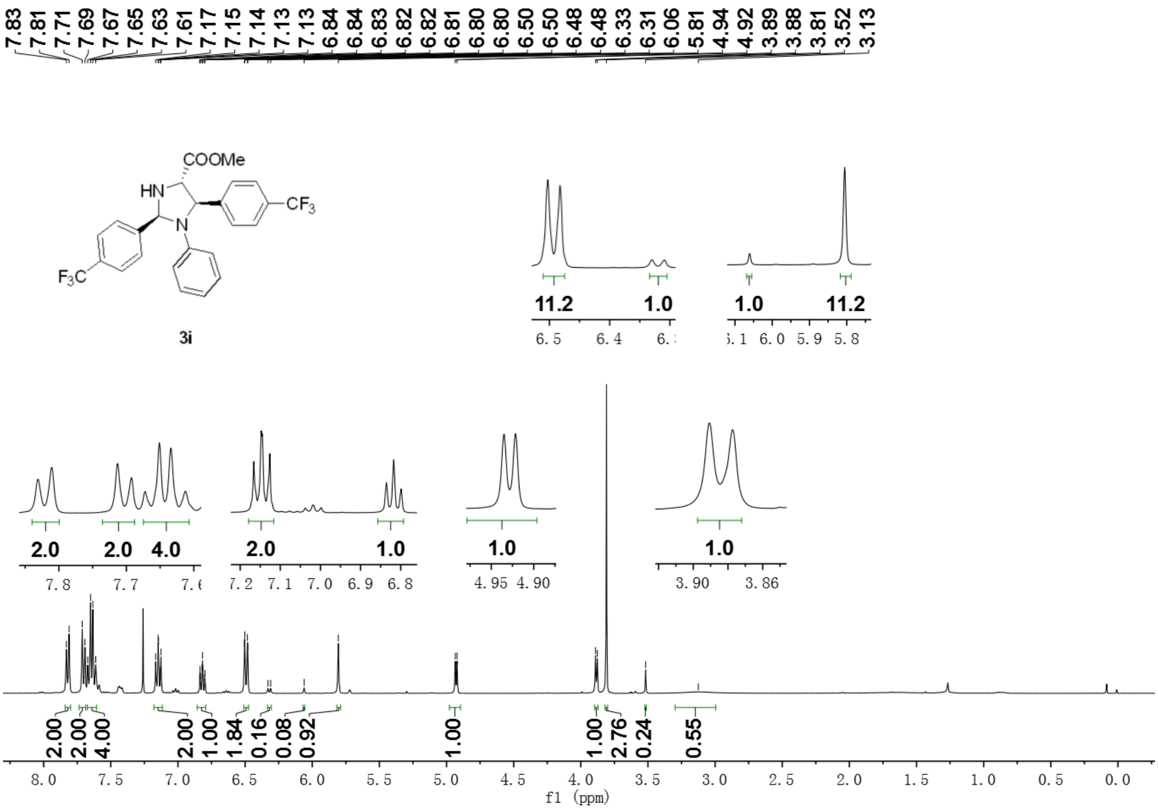

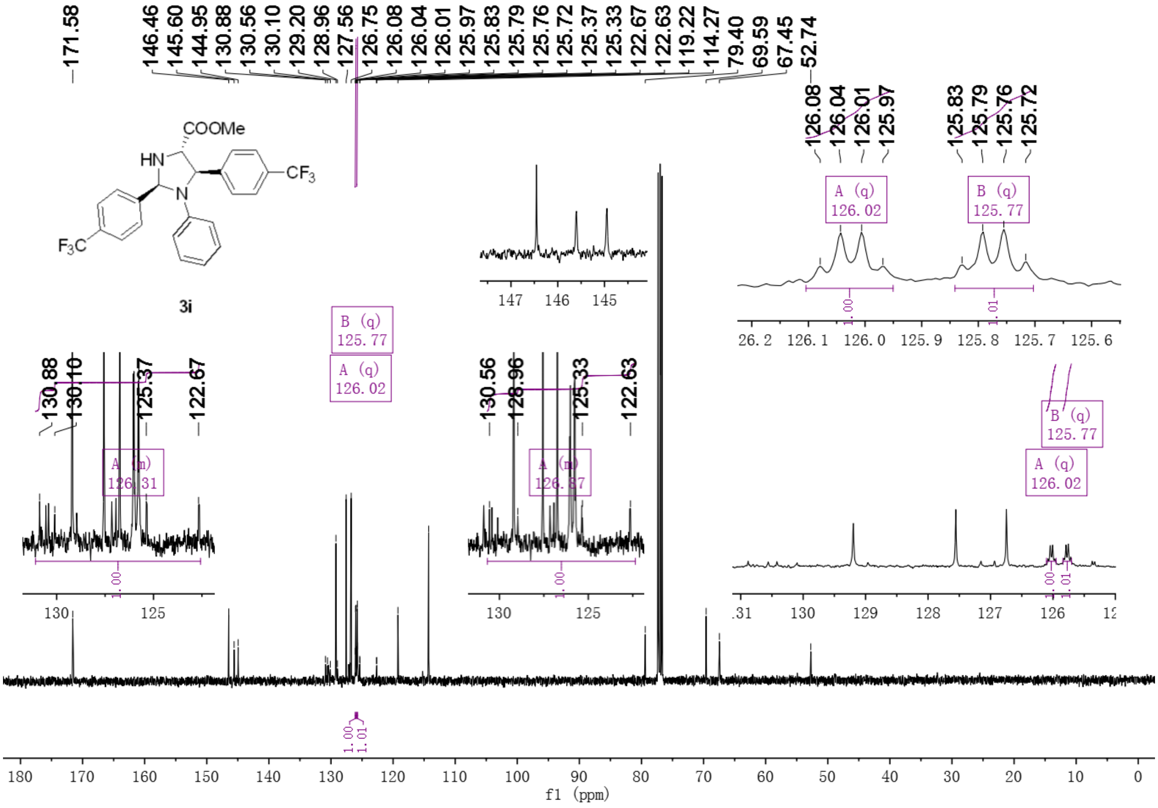

$^{19}F\{^1H\}$ NMR spectra of compound of **3i** (procedure A) in $CDCl_3$

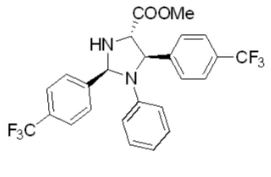

3i

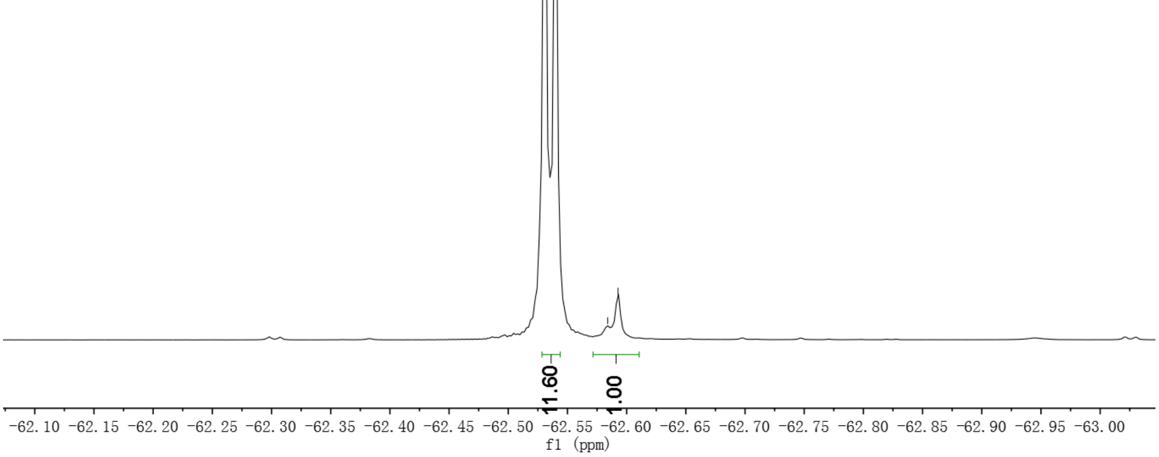

^{1}H NMR and ^{13}C{^{1}H} NMR spectra of compound of **3j** (procedure A) in $CDCl_3$

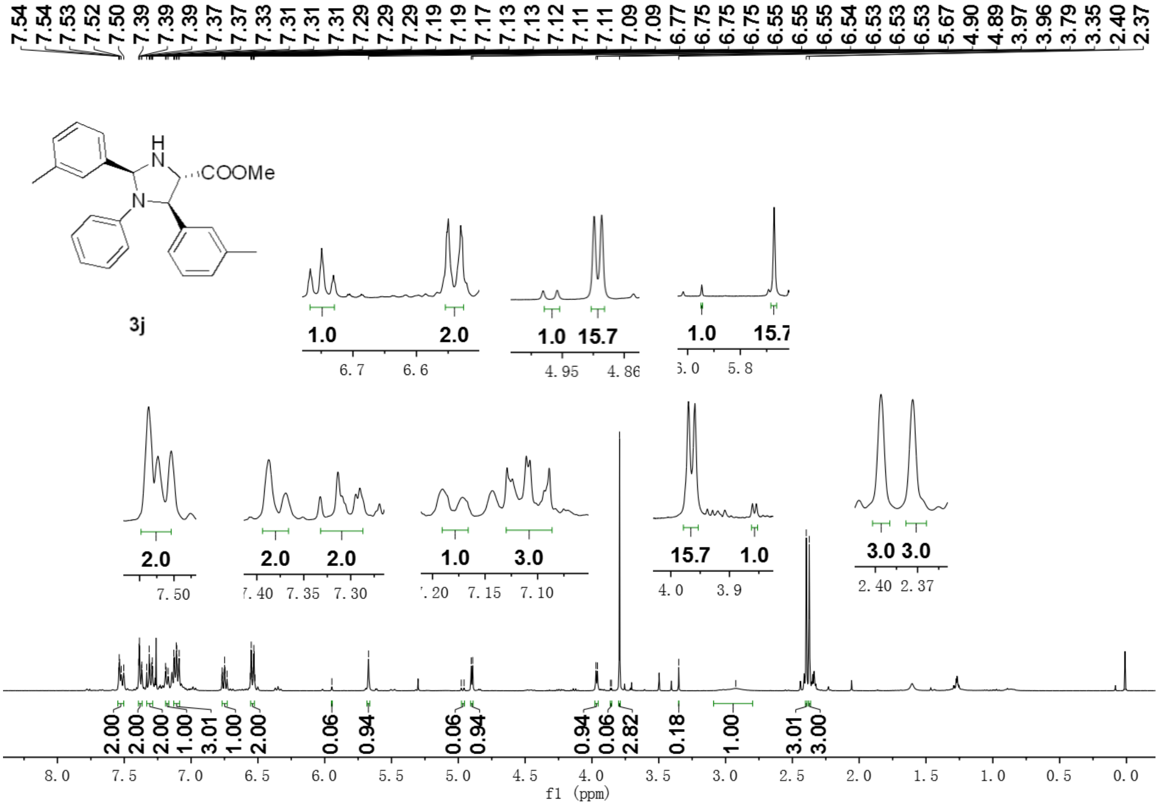

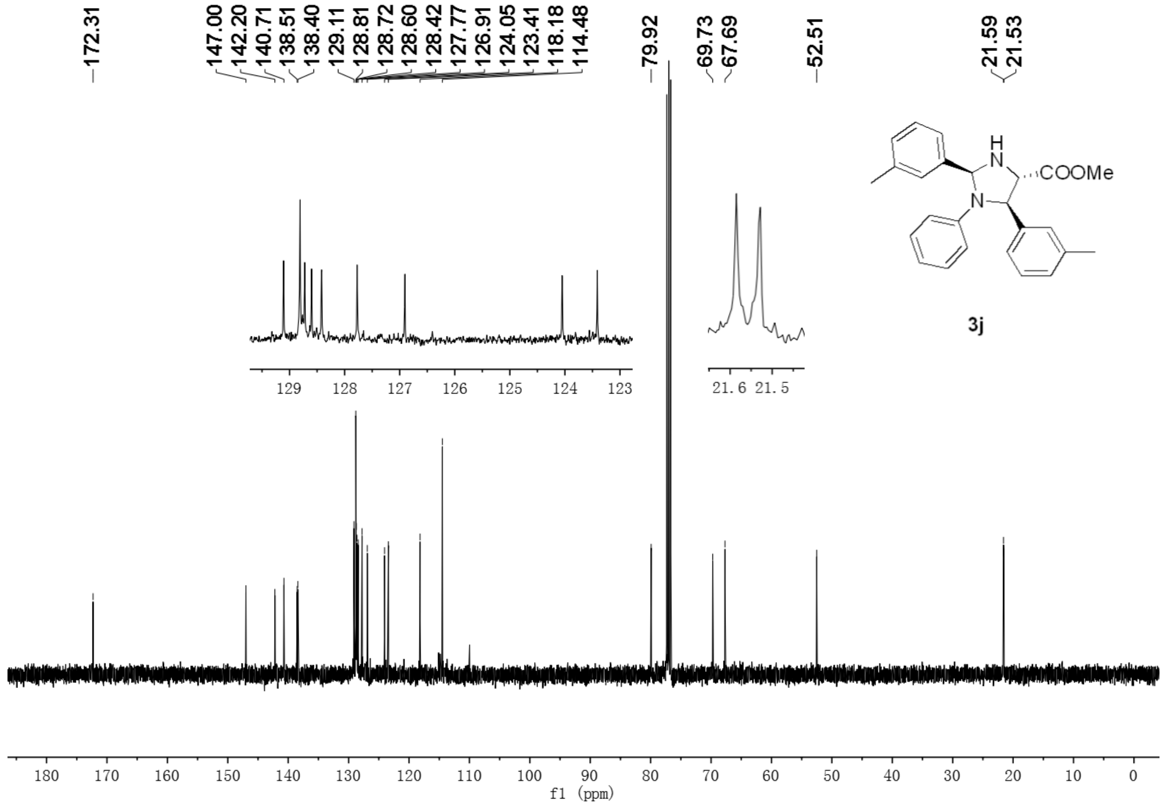

^{1}H NMR and $^{13}C\{^1H\}$ NMR spectra of compound of **3k** (procedure A) in $CDCl_3$

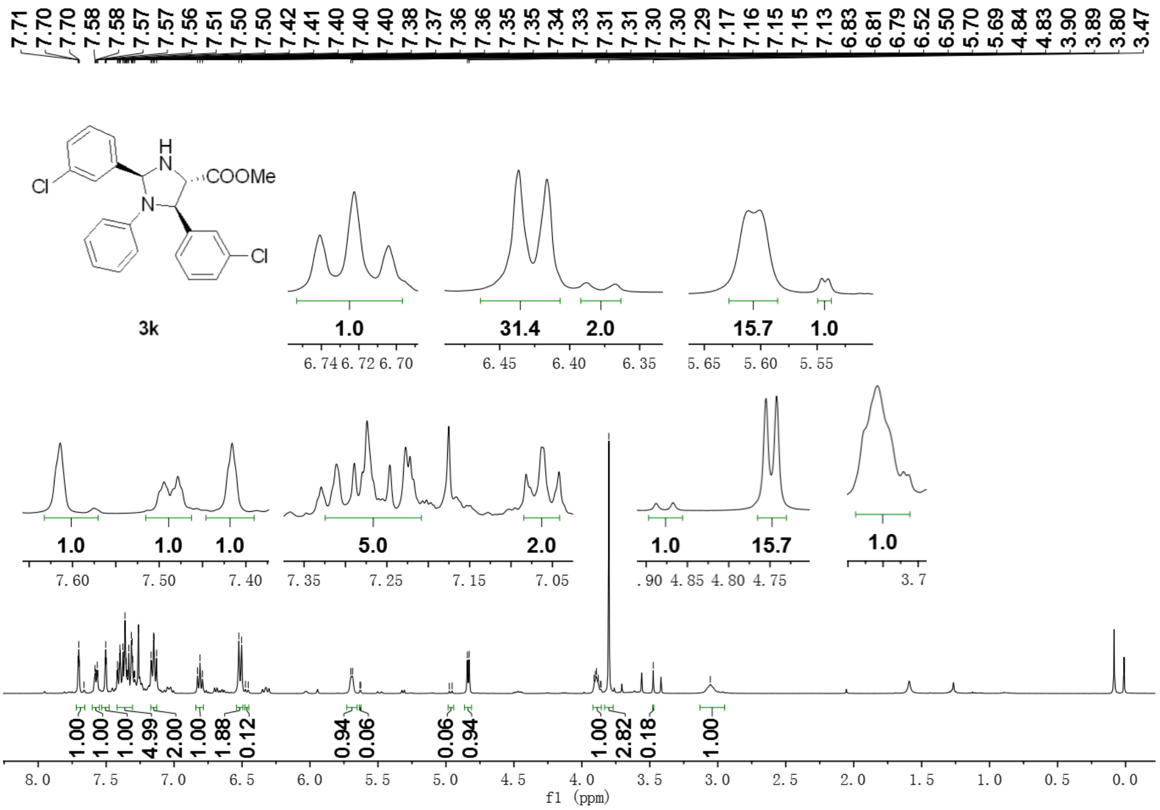

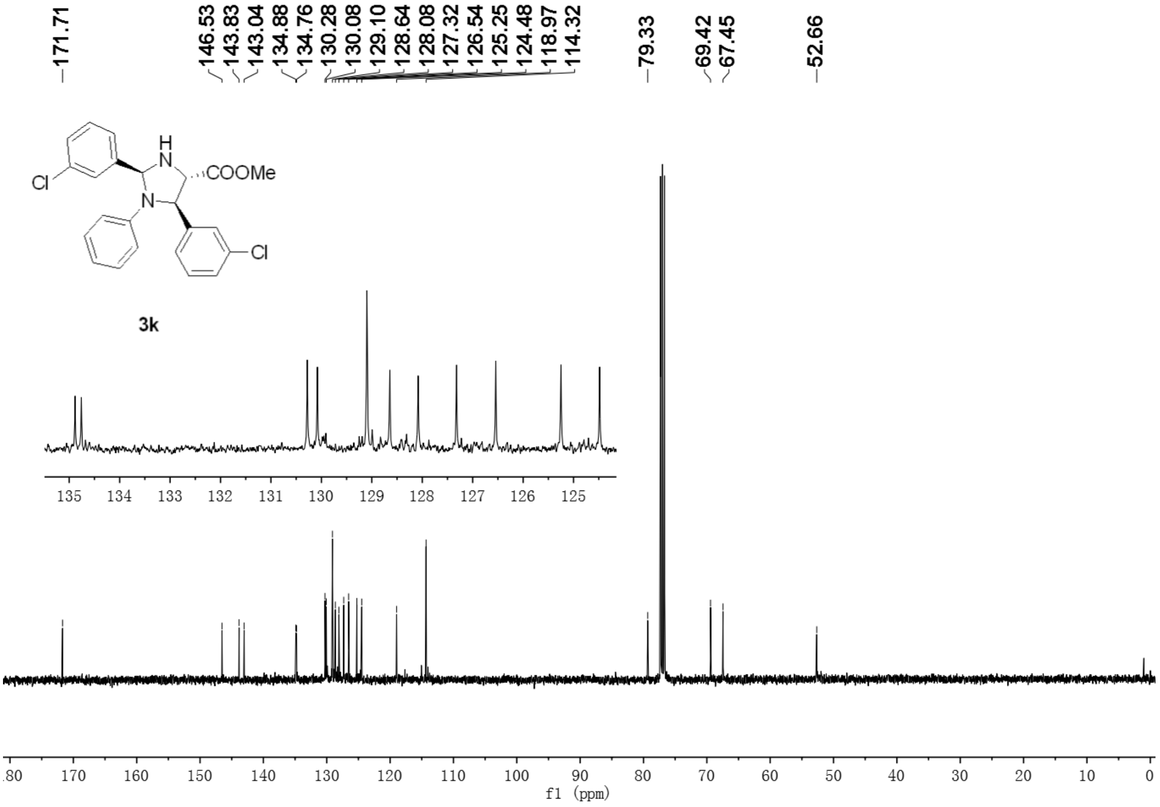

^{1}H NMR and ^{13}C{^{1}H} NMR spectra of compound of (2*R*,4*R*,5*S*)-**3k** (procedure A) in $CDCl_3$

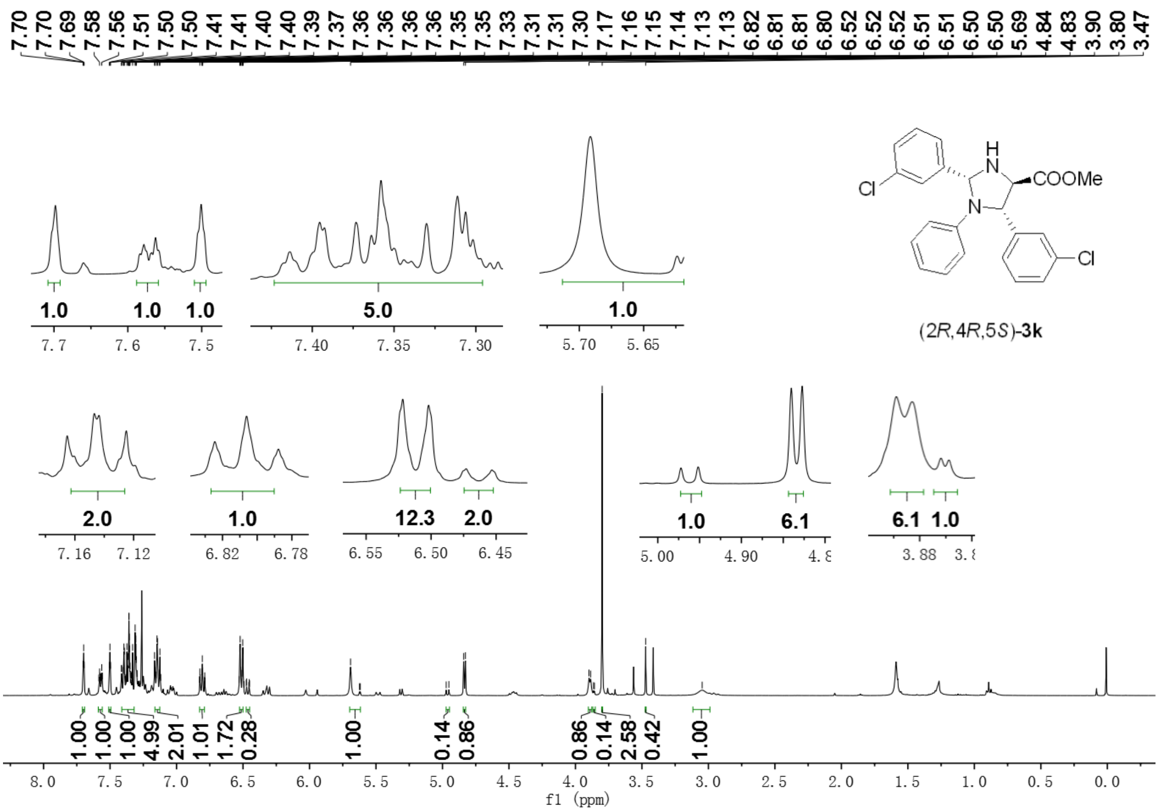

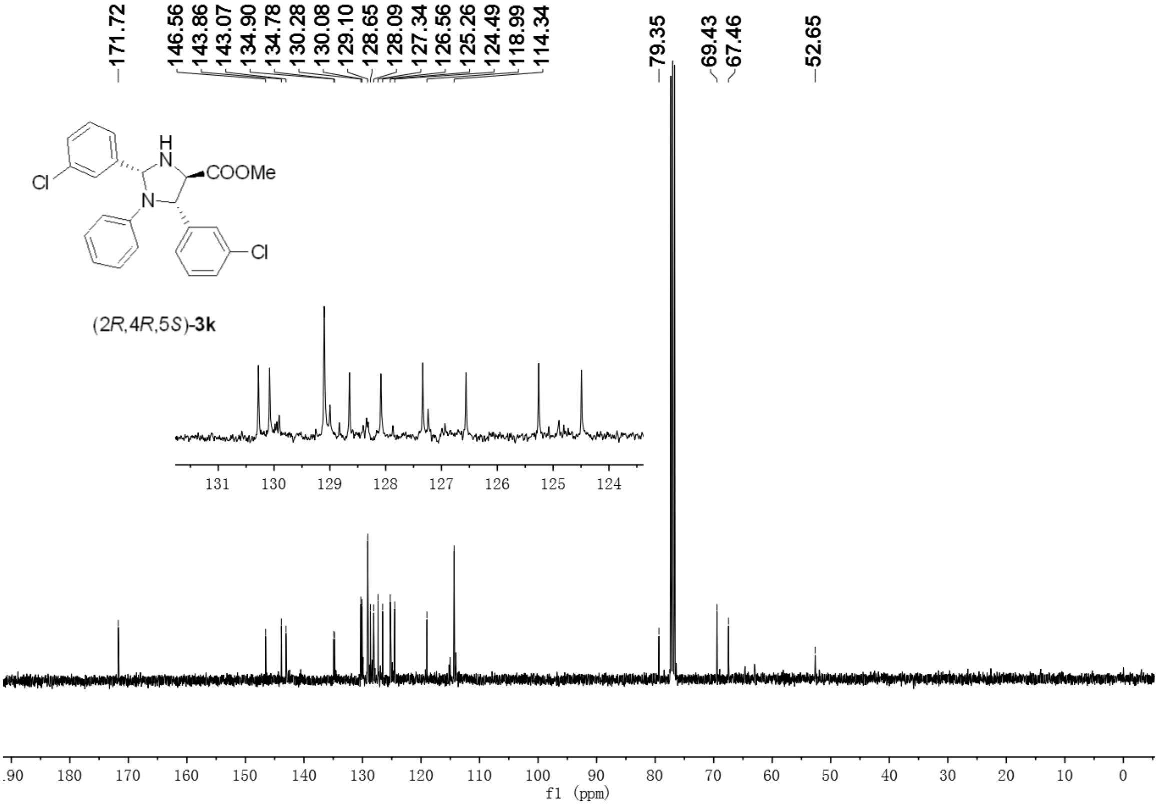

^{1}H NMR and ^{13}C{^{1}H} NMR spectra of compound of **3l** (procedure A) in $CDCl_3$

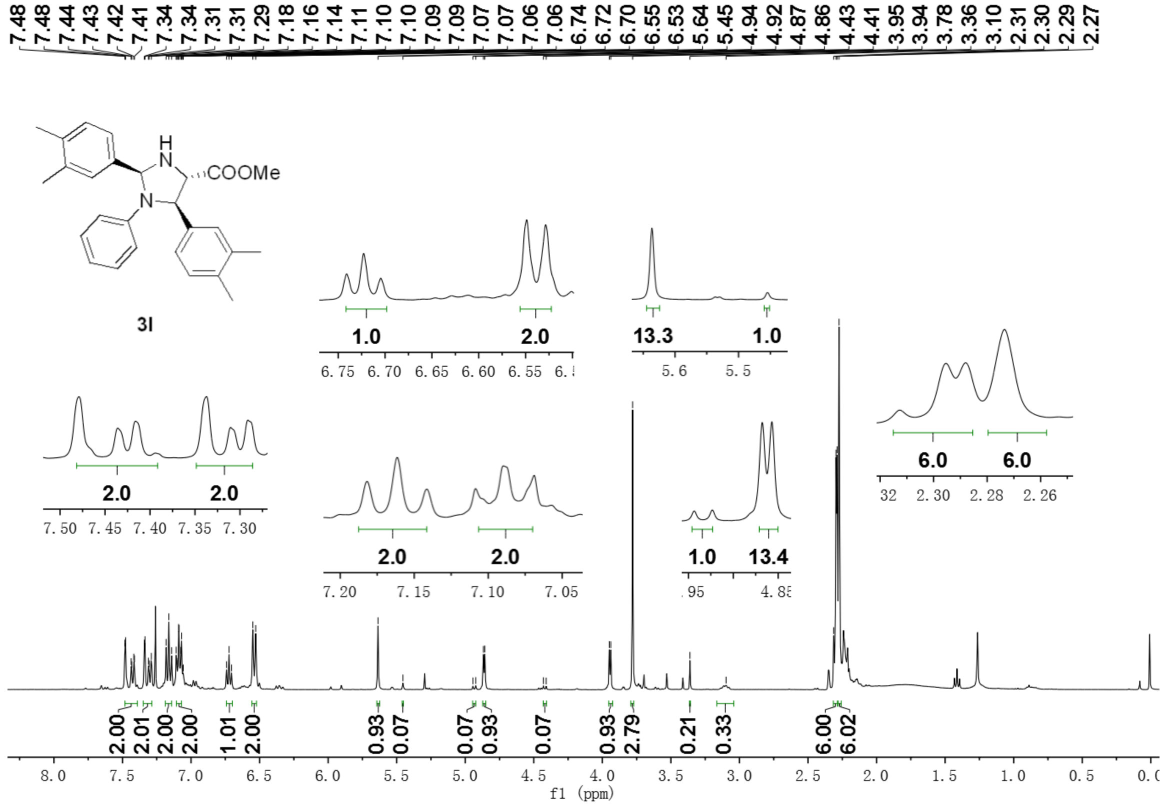

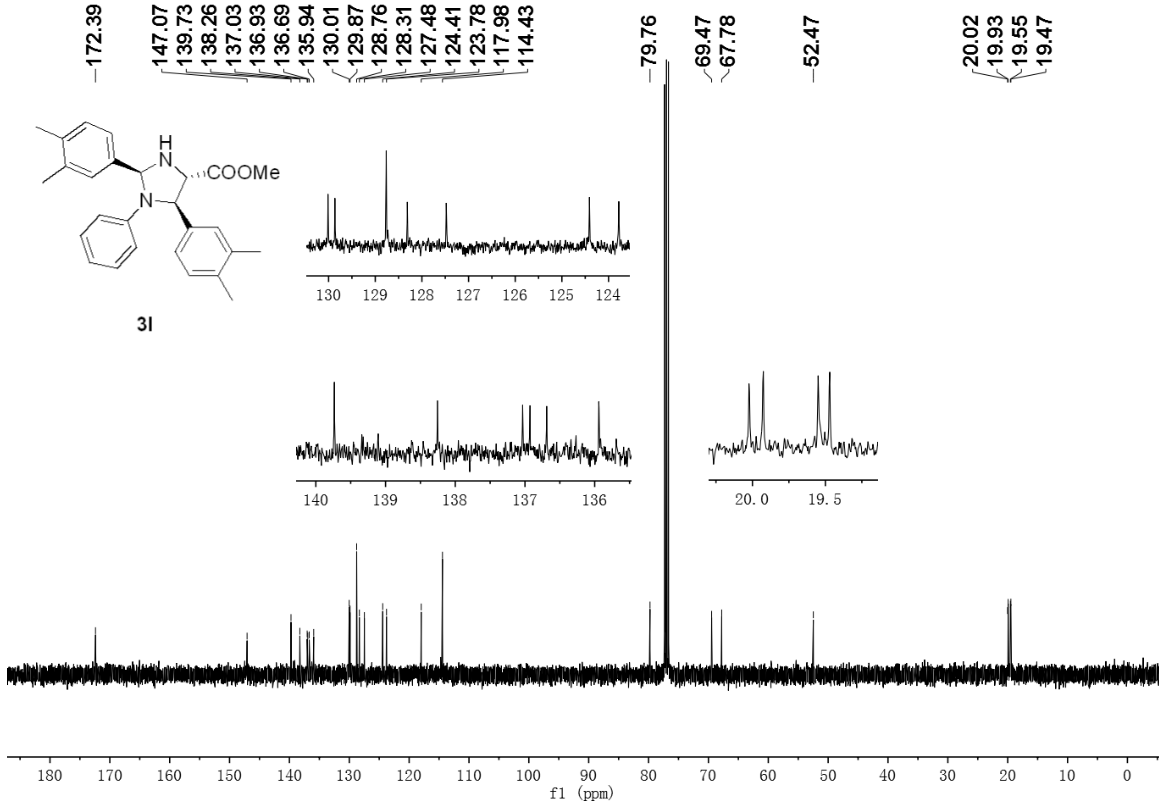

^{1}H NMR and ^{13}C{^{1}H} NMR spectra of compound of **3m** (procedure A) in $CDCl_3$

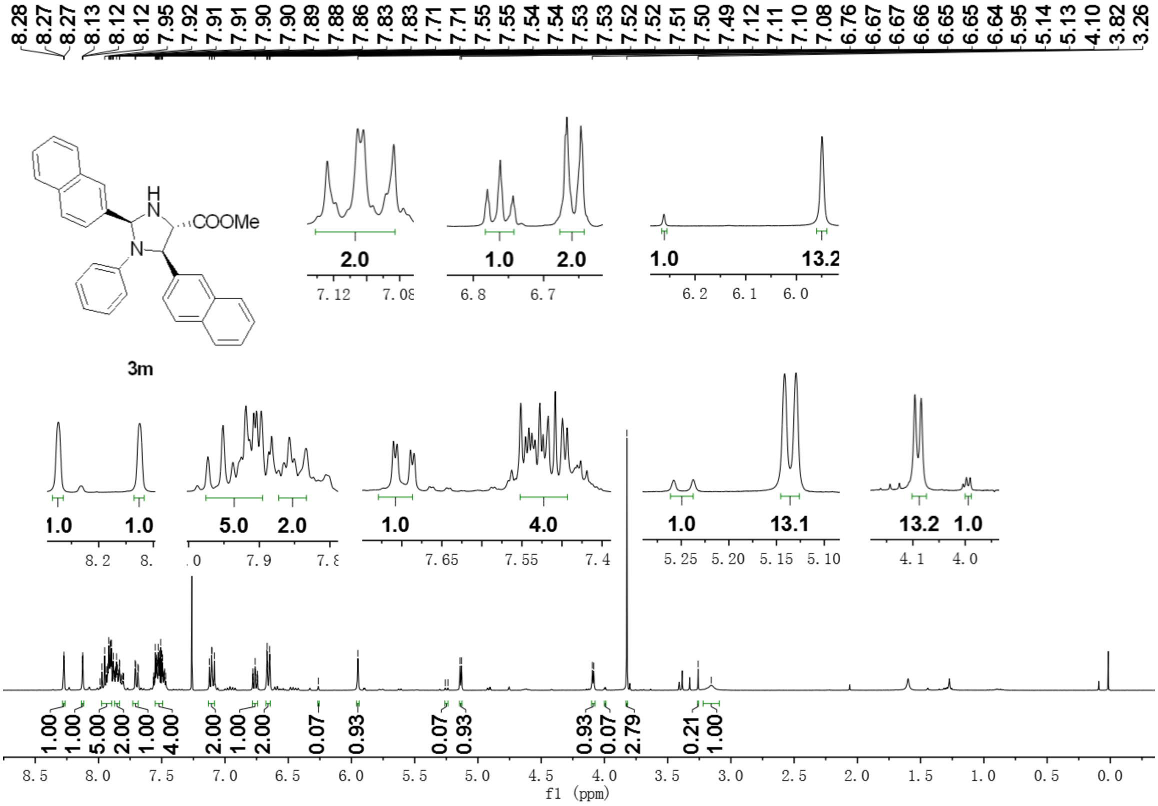

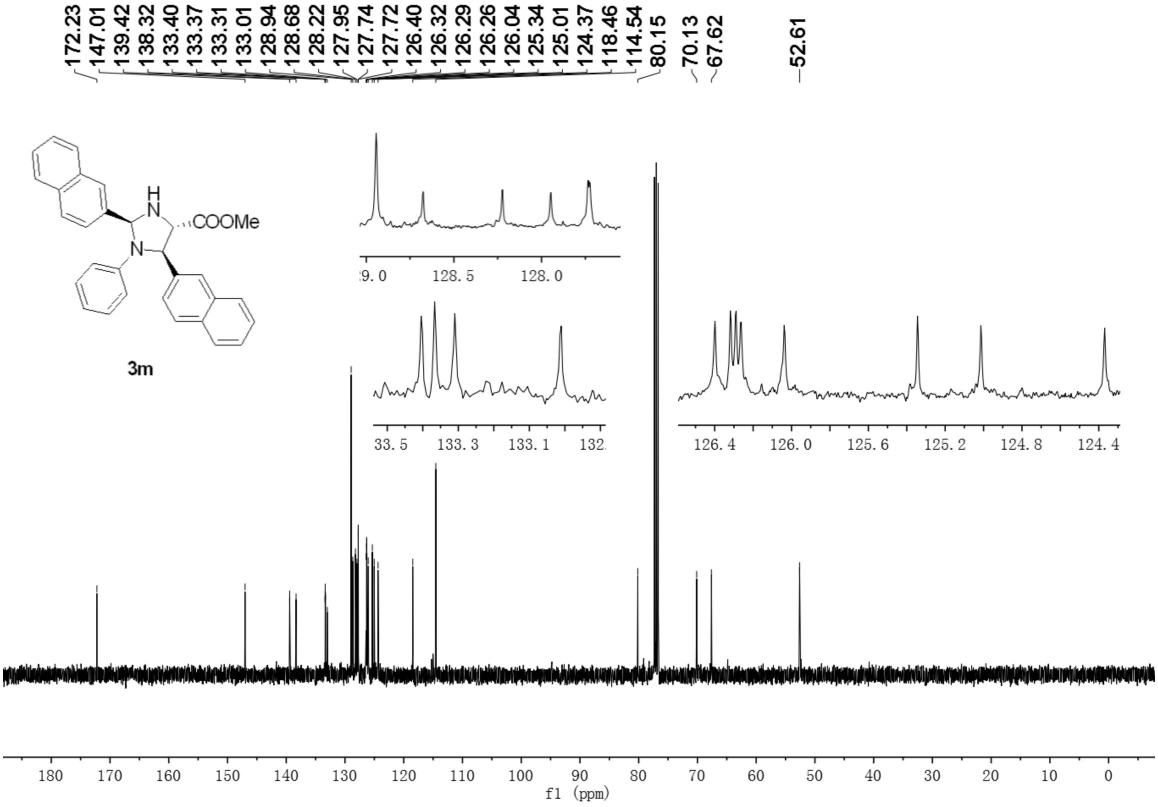

^{1}H NMR and ^{13}C{^{1}H} NMR spectra of compound of **3n** (procedure A) in $CDCl_3$

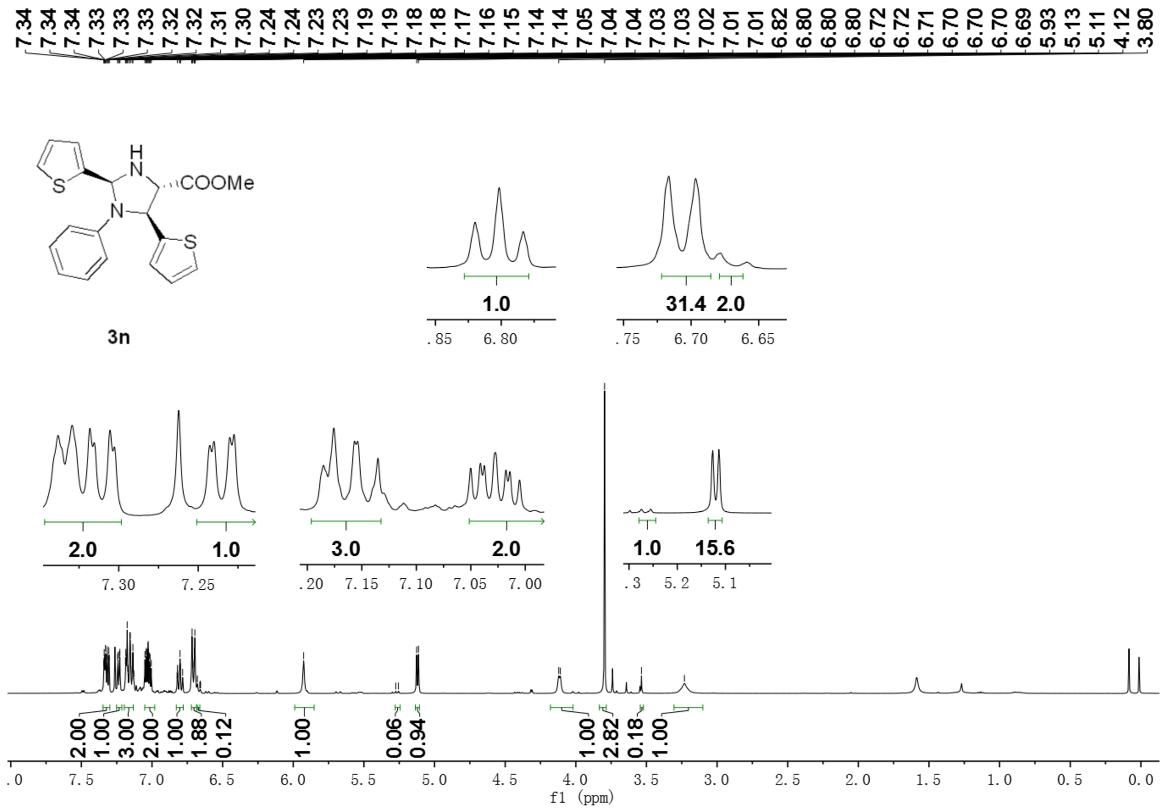

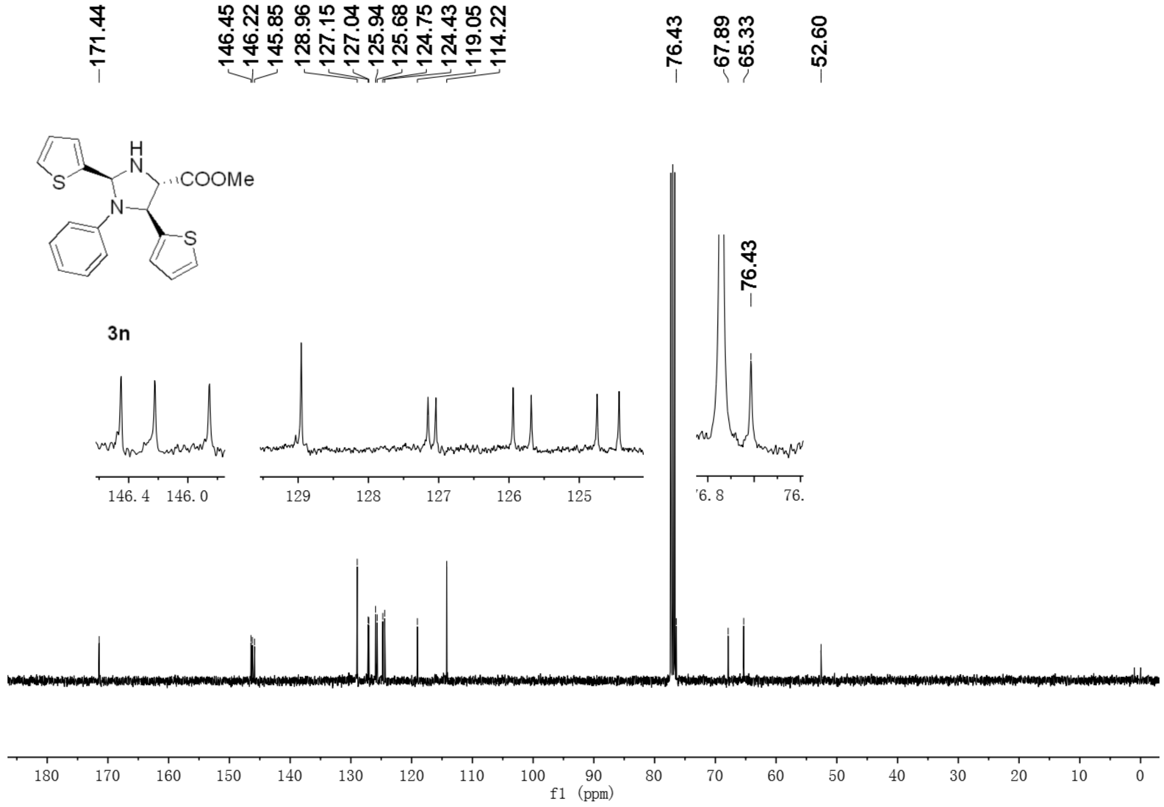

^{1}H NMR and $^{13}C\{^{1}H\}$ NMR spectra of compound of (2*R*,4*R*,5*R*)-**3n** (procedure A) in $CDCl_3$

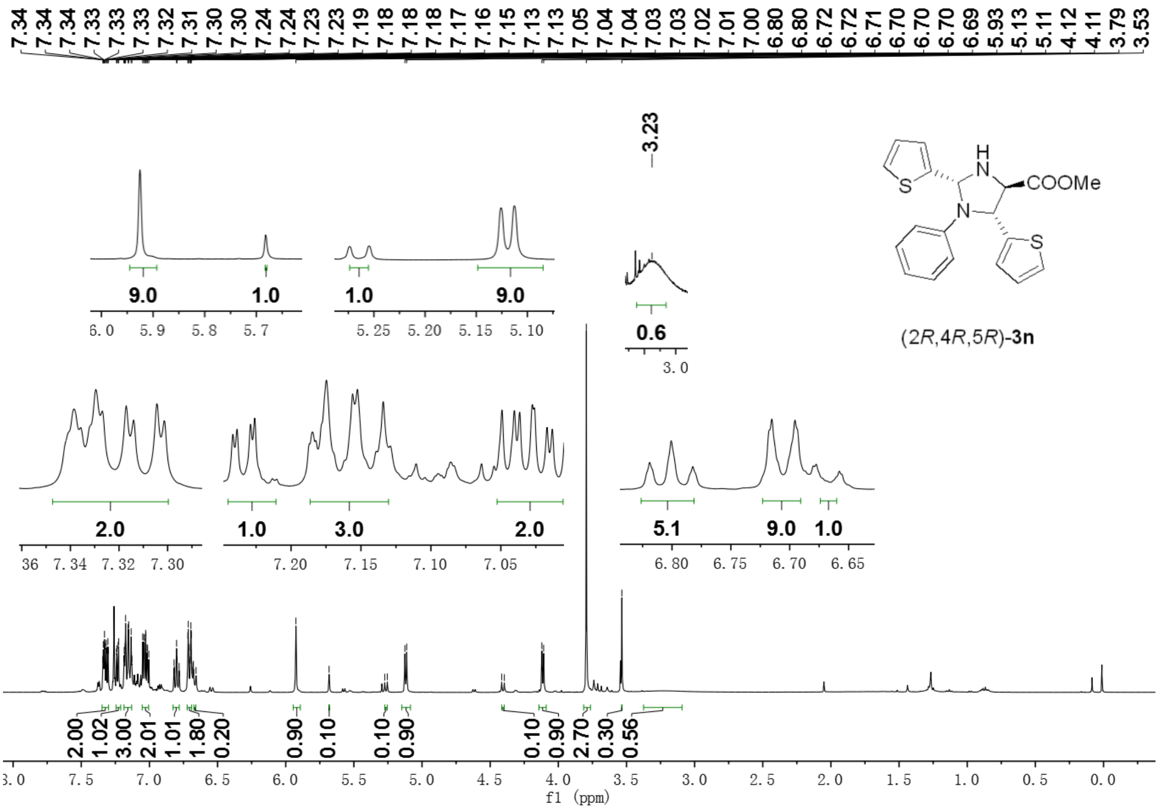

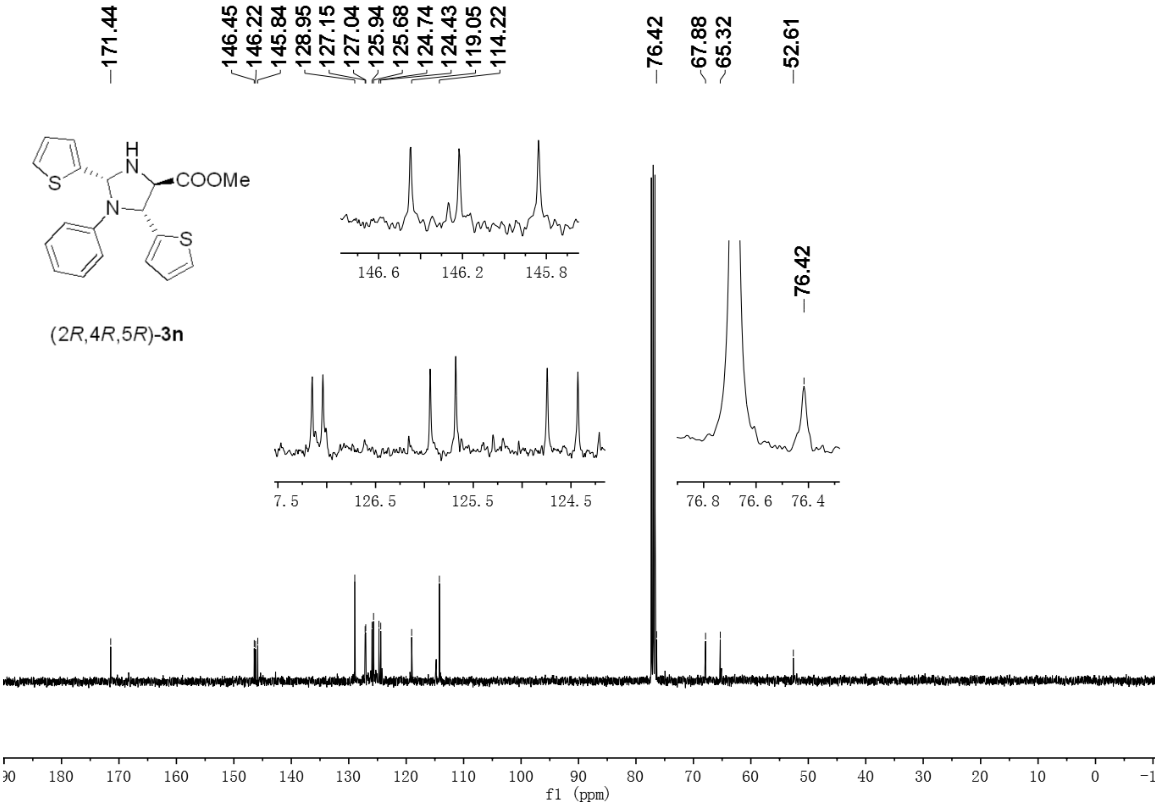

^{1}H NMR and ^{13}C{^{1}H} NMR spectra of compound of **3o** (procedure A) in $CDCl_3$

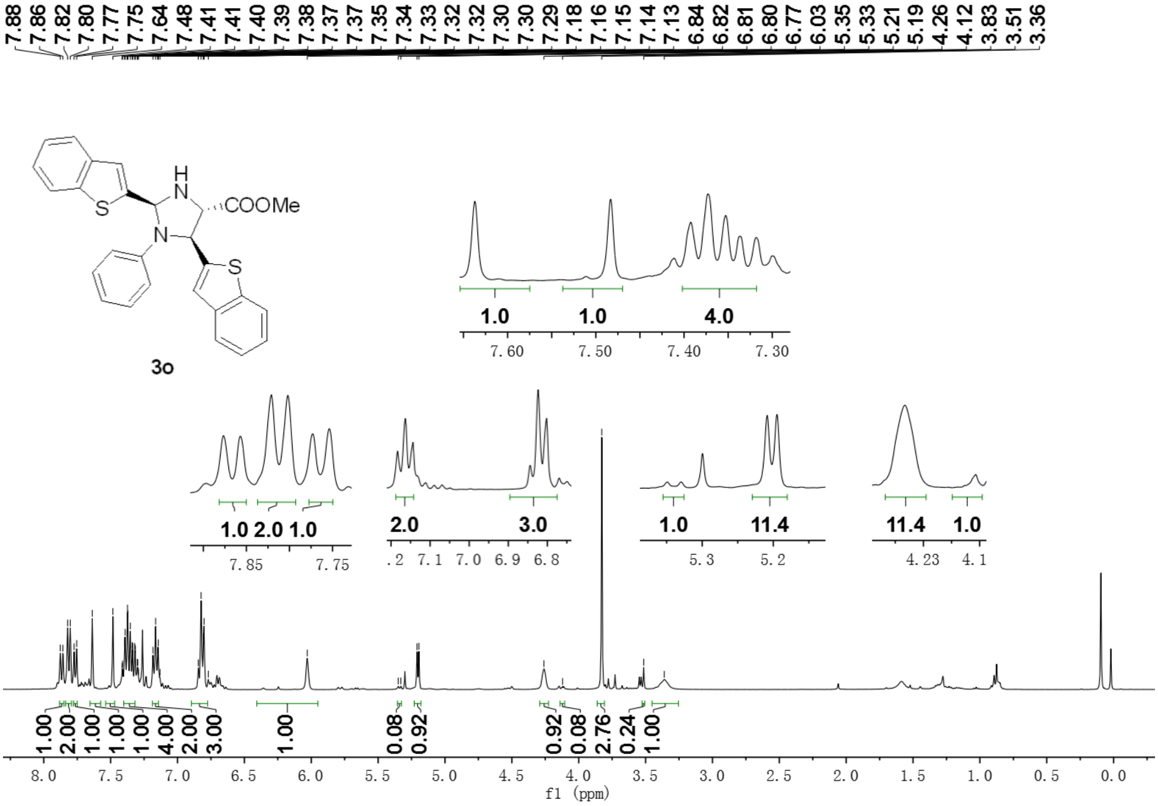

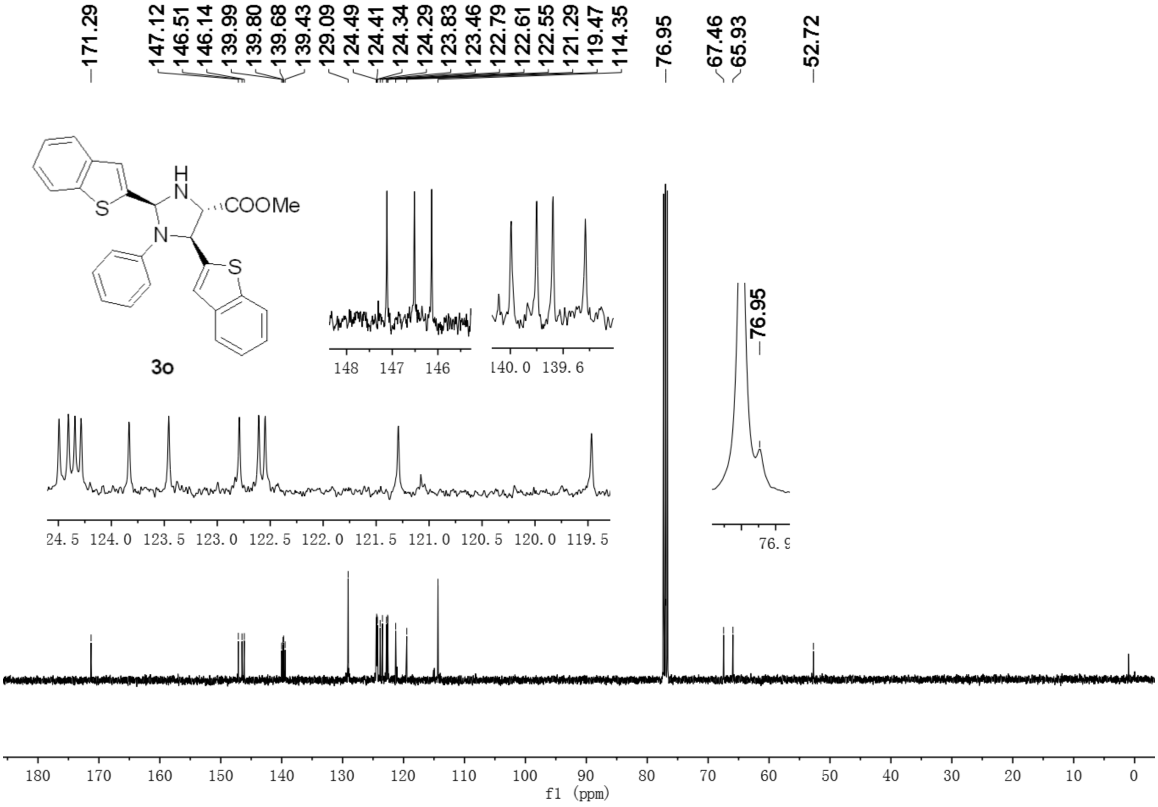

^{1}H NMR and ^{13}C{^{1}H} NMR spectra of compound of (2*R*,4*R*,5*R*)-**3o** (procedure A) in $CDCl_3$

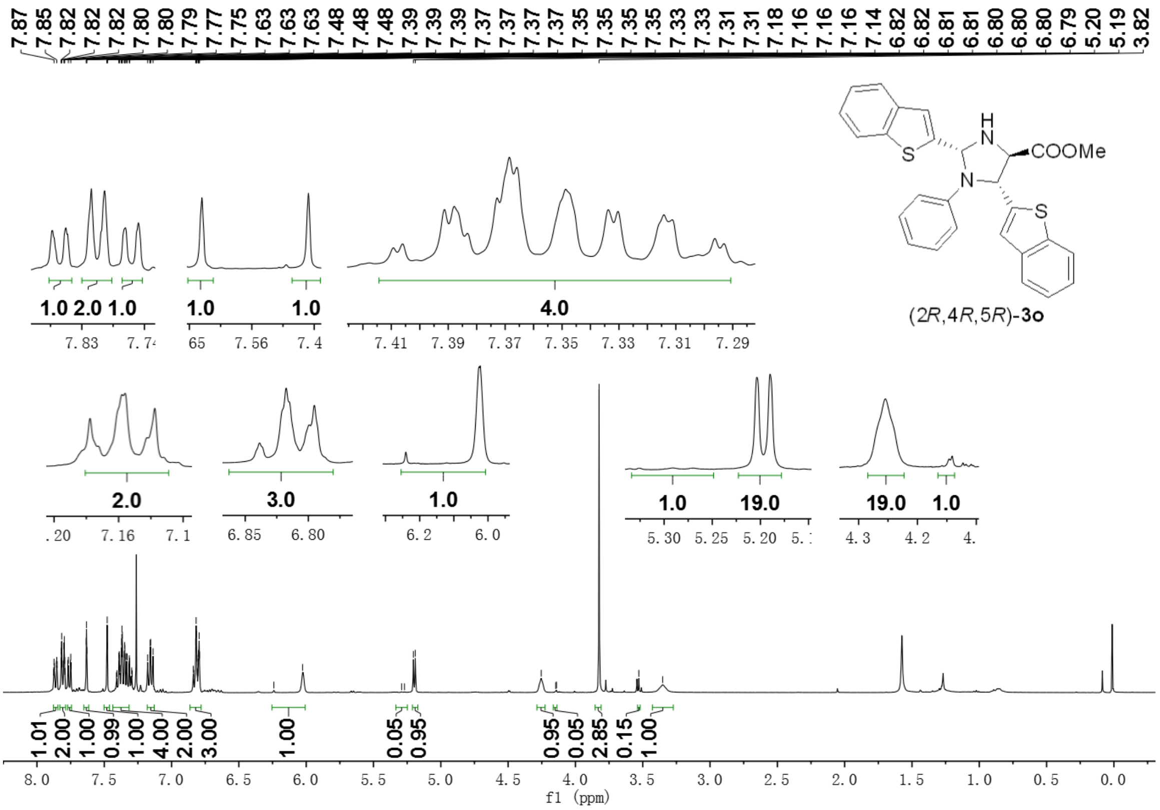

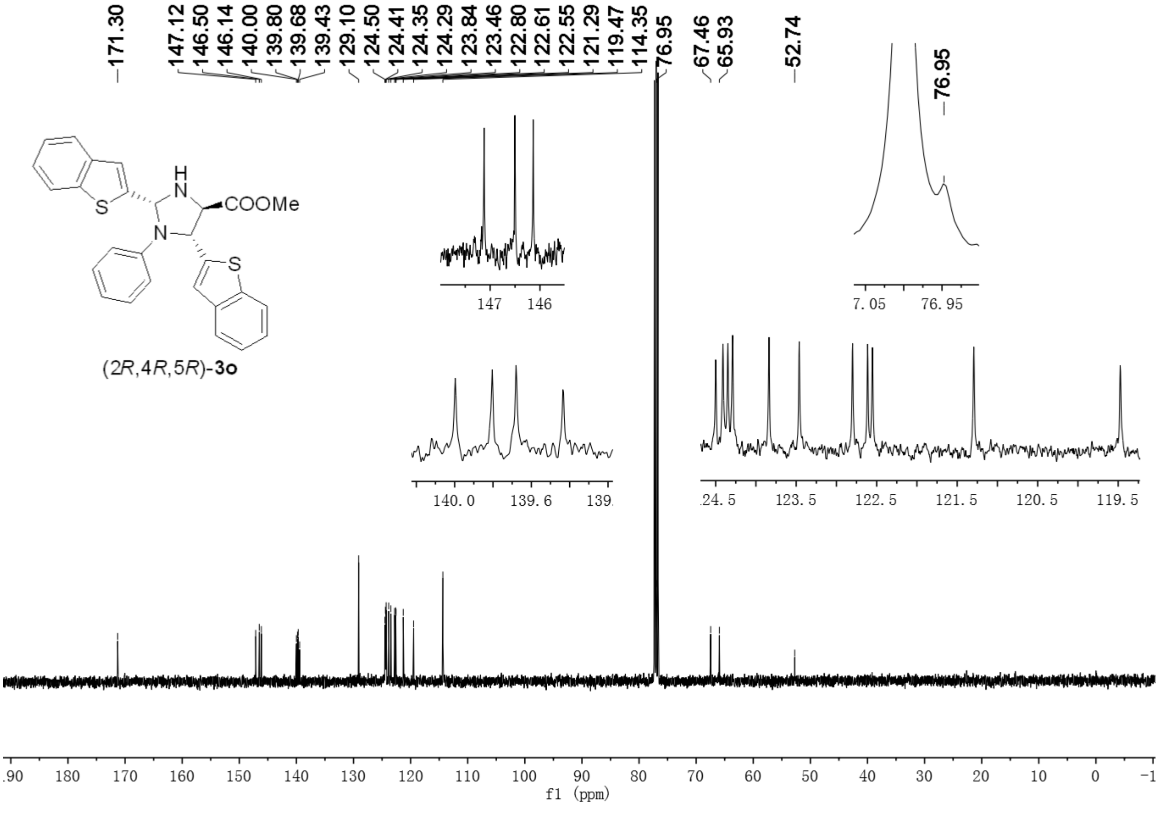

^{1}H NMR and ^{13}C{^{1}H} NMR spectra of compound of **3p** (procedure A) in $CDCl_3$

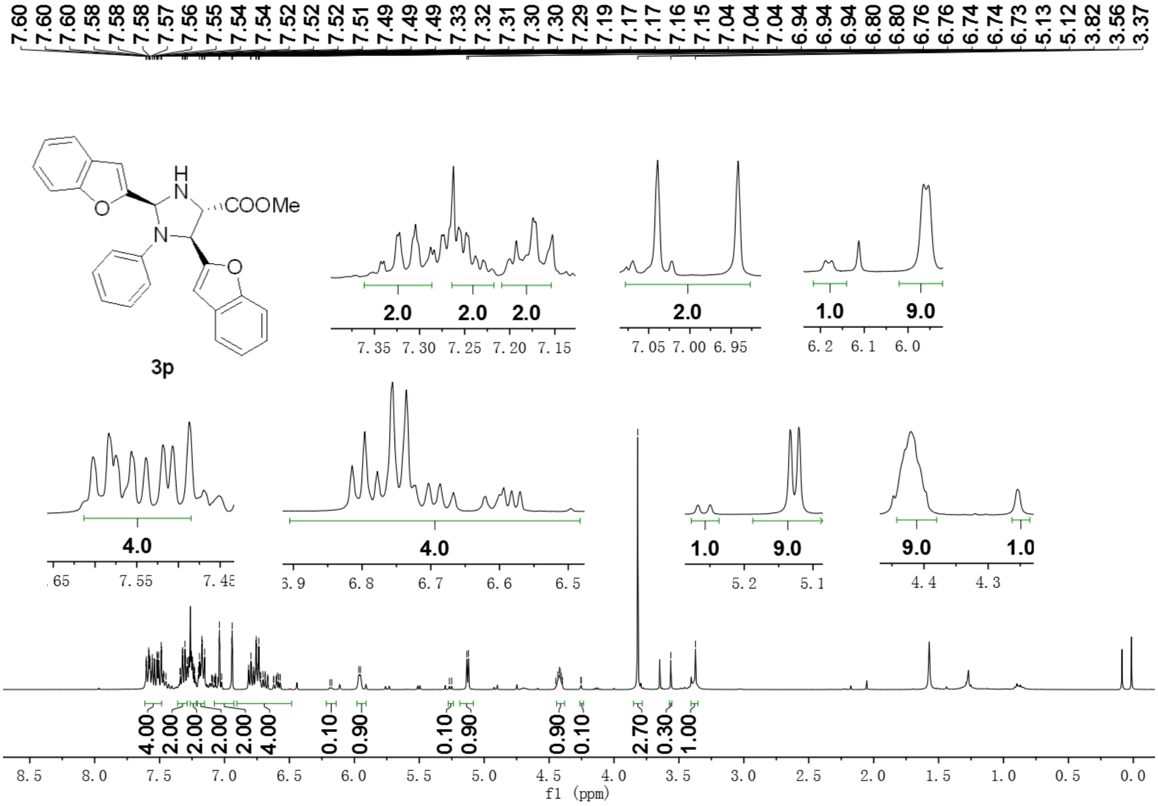

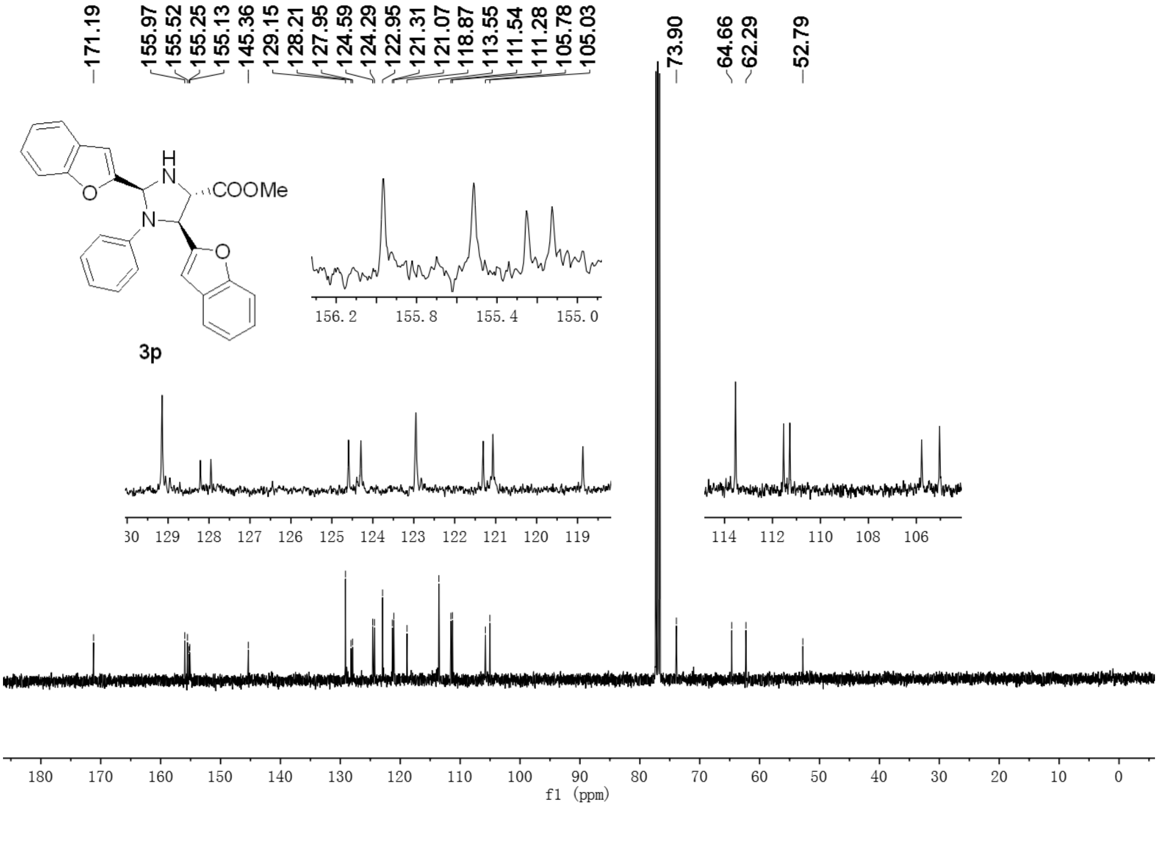

^{1}H NMR and ^{13}C{^{1}H} NMR spectra of compound of **3q** (procedure A) in $CDCl_3$

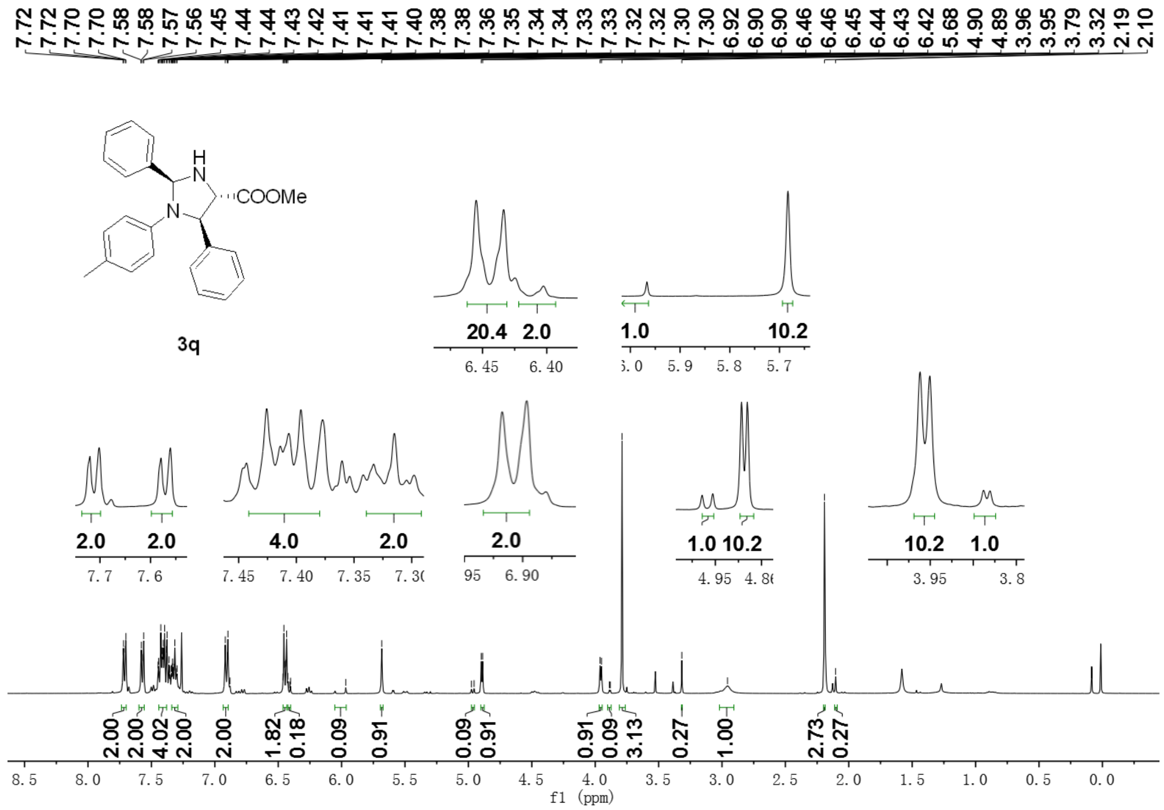

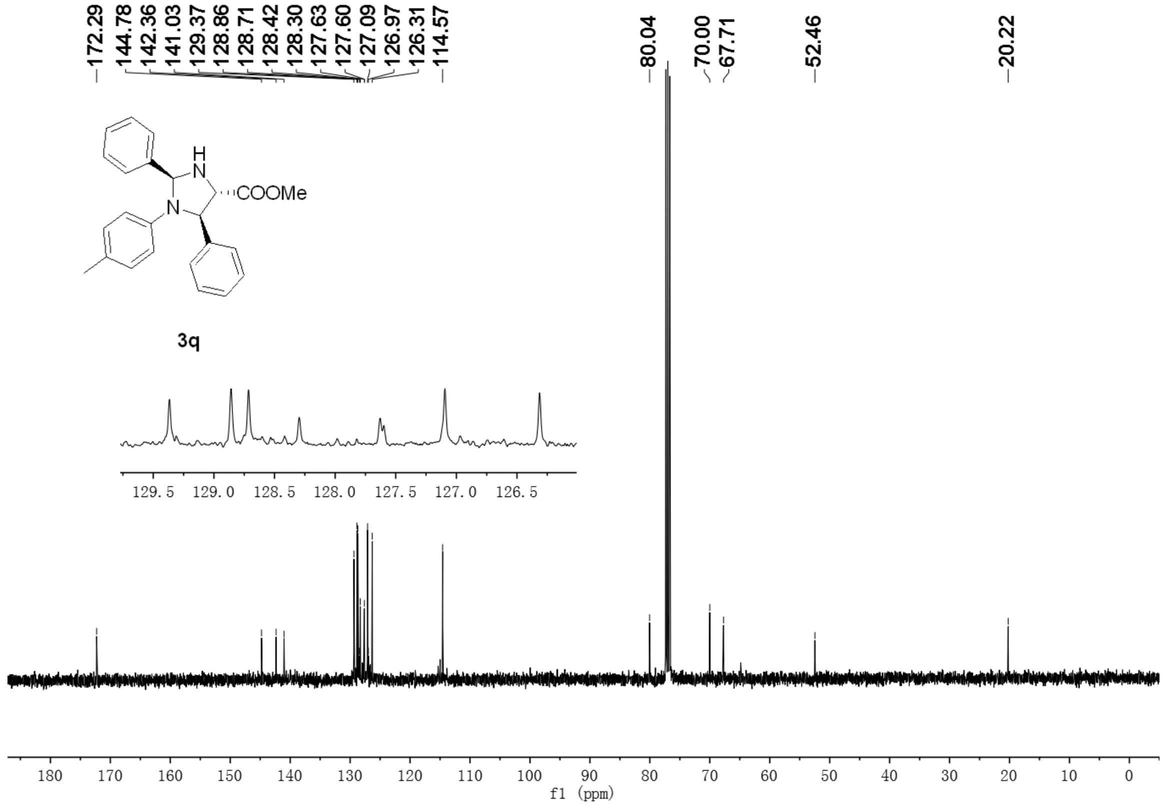

^{1}H NMR and ^{13}C{^{1}H} NMR spectra of compound of **3q** (procedure B) in $CDCl_3$

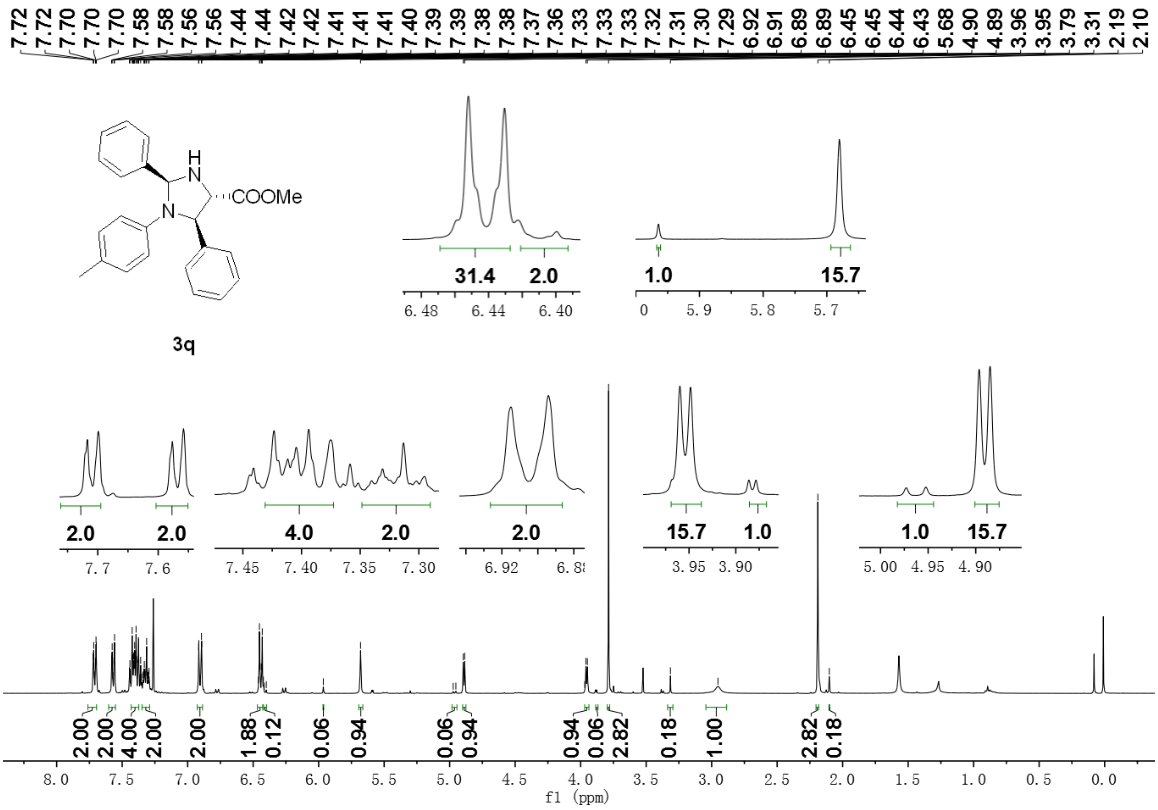

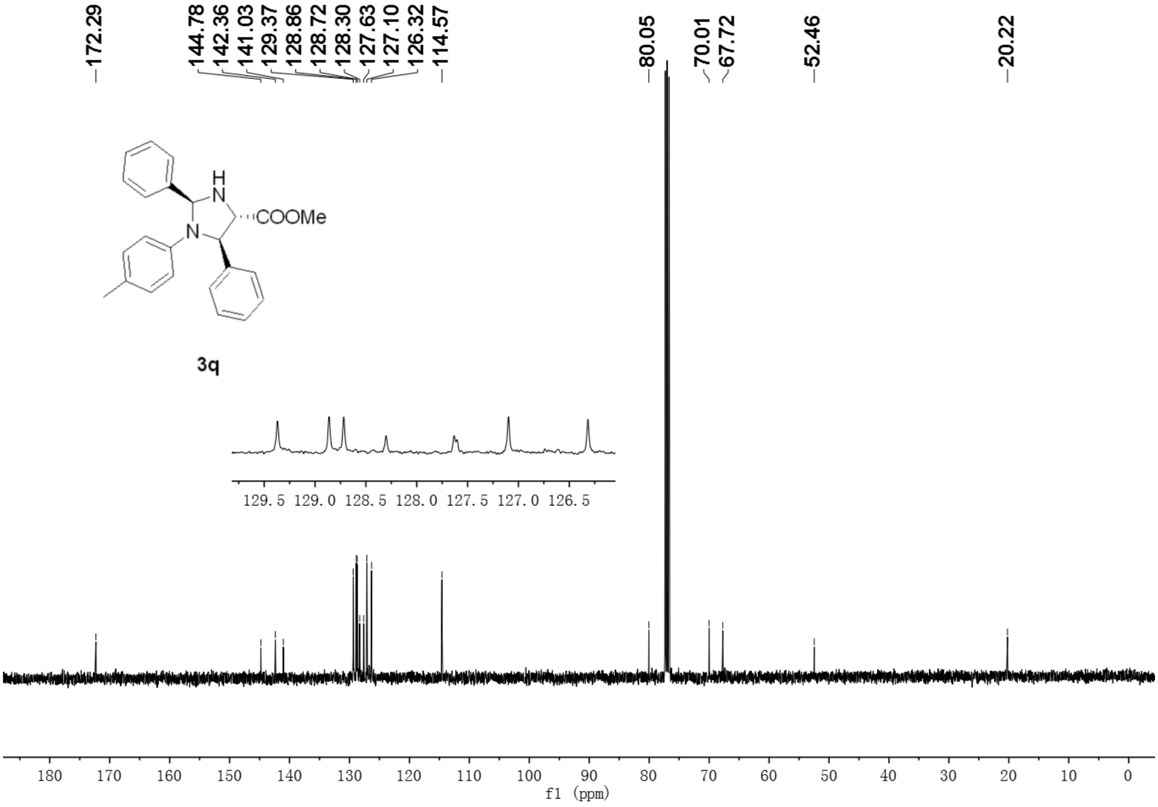

^{1}H NMR and $^{13}C\{^{1}H\}$ NMR spectra of compound of **3r** (procedure A) in d_6-Acetone

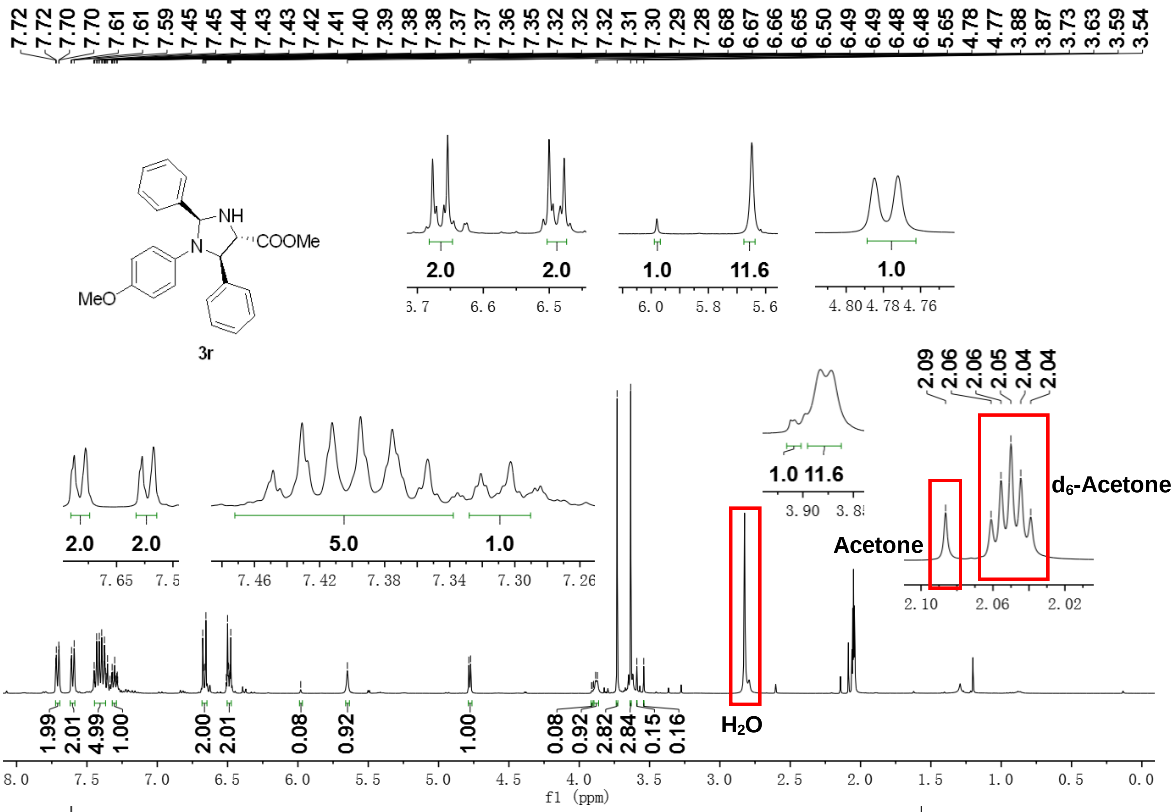

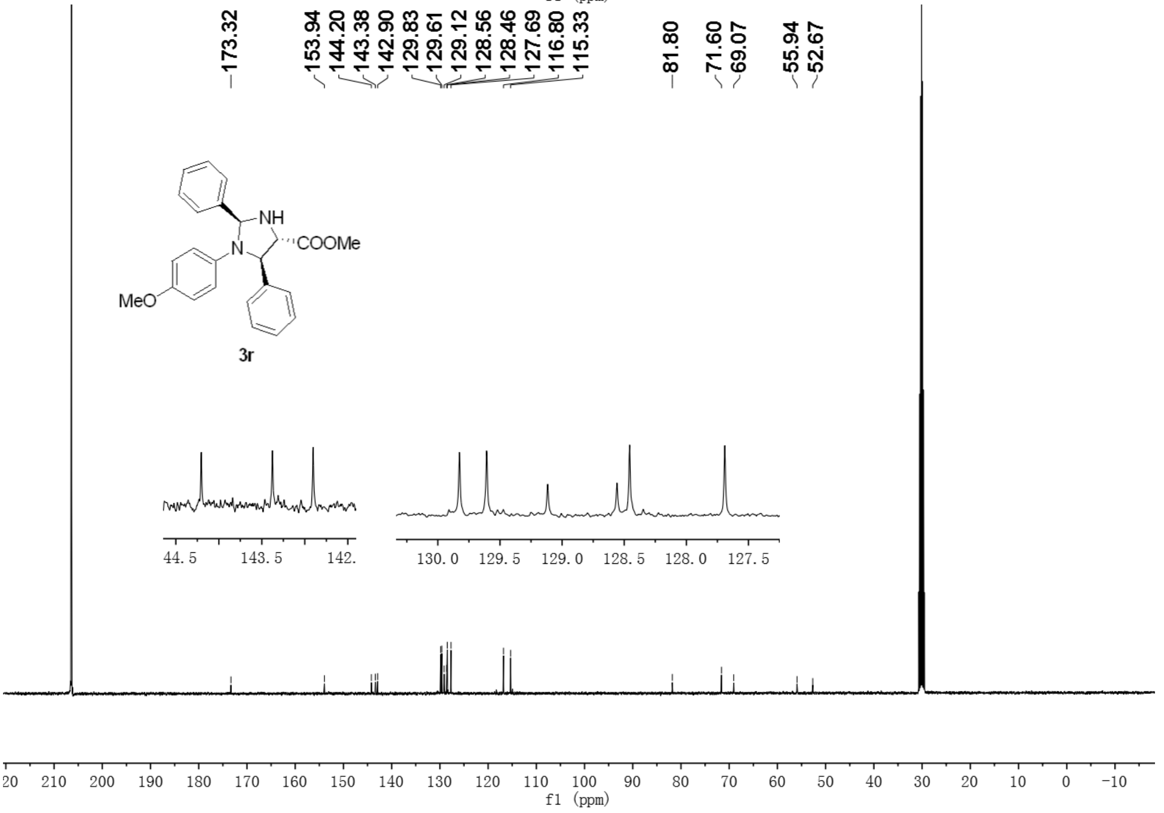

^{1}H NMR and $^{13}C\{^{1}H\}$ NMR spectra of compound of **3s** (procedure A) in $CDCl_3$

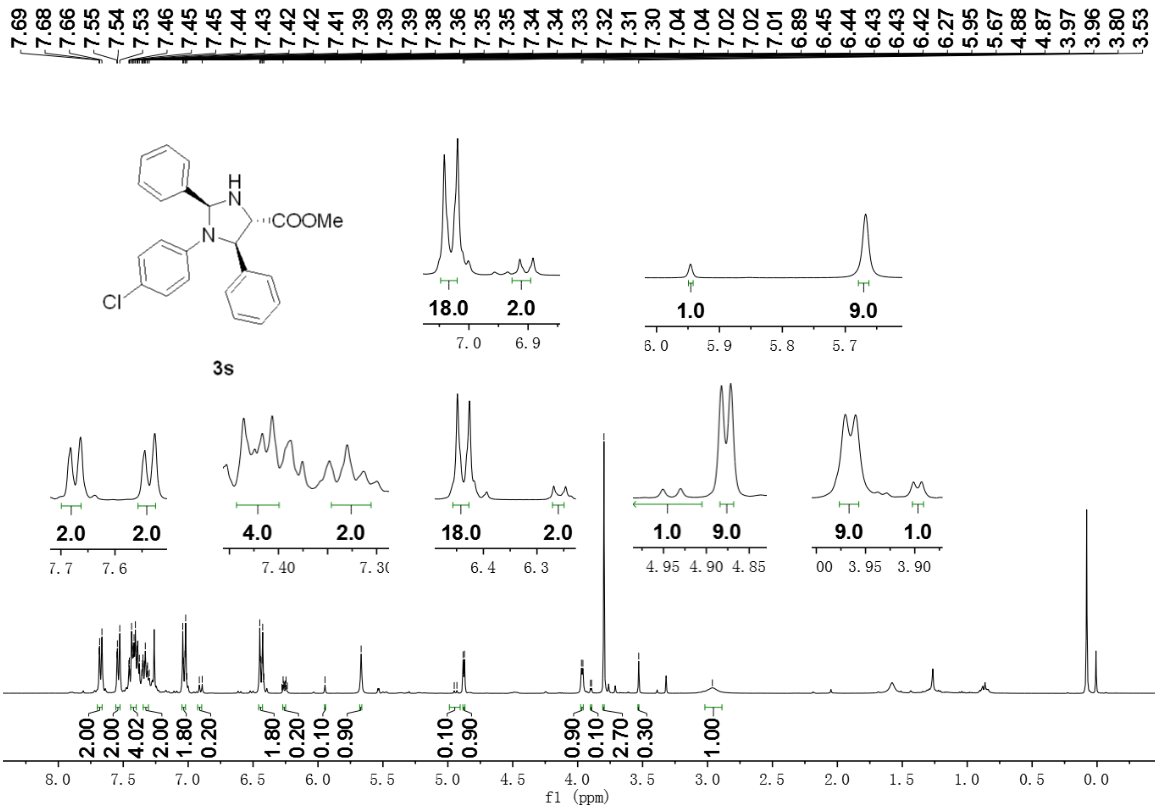

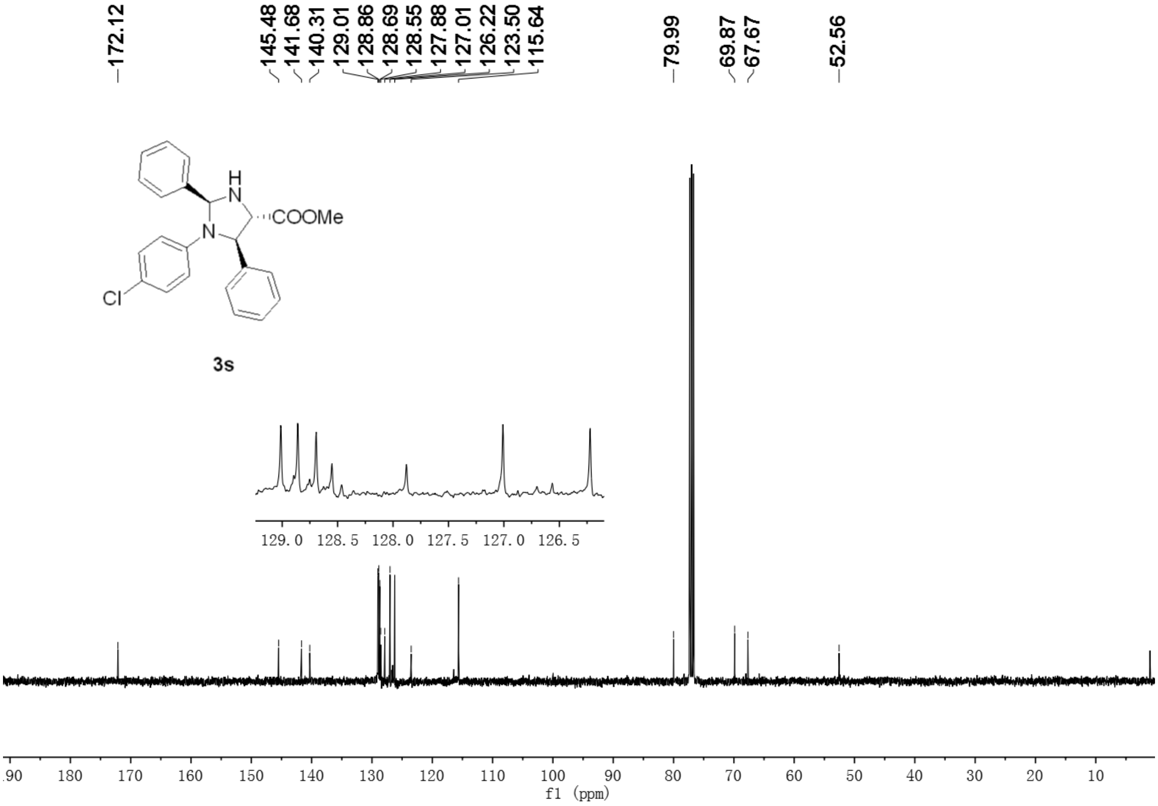

^{1}H NMR and $^{13}C\{^{1}H\}$ NMR spectra of compound of **3s** (procedure B) in $CDCl_3$

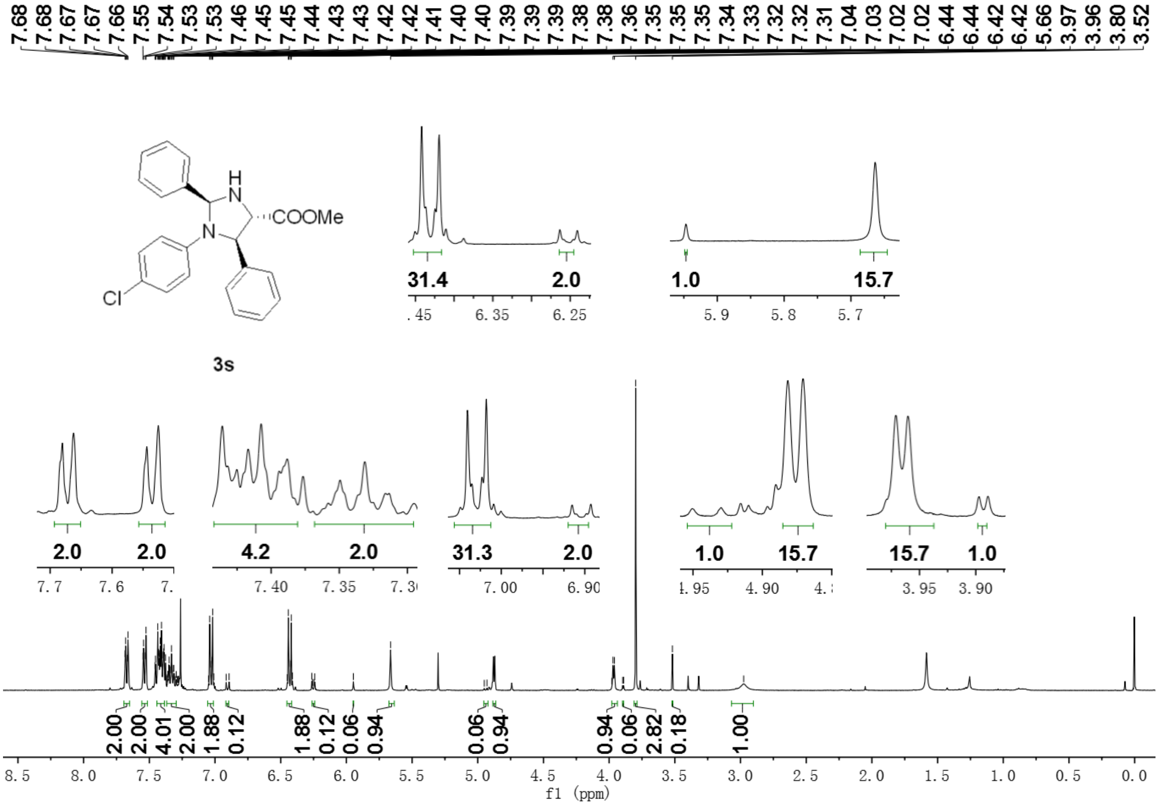

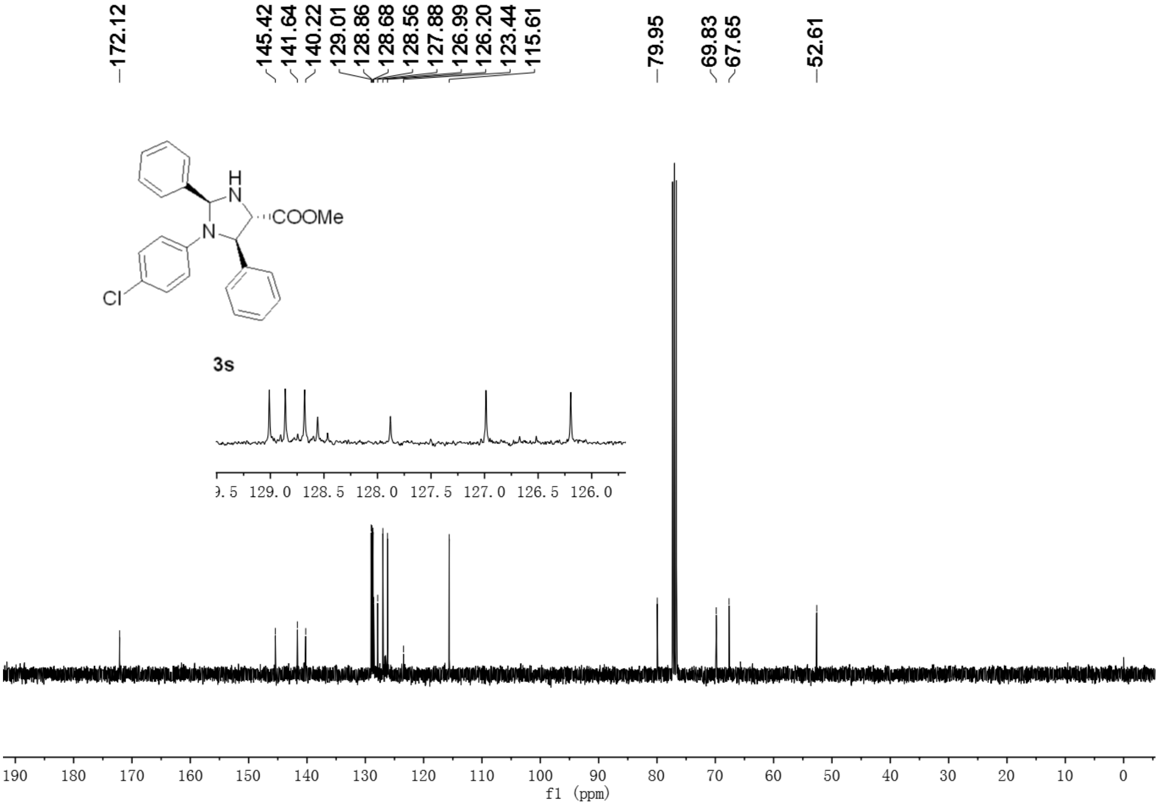

^{1}H NMR and ^{13}C{^{1}H} NMR spectra of compound of **3t** (procedure A) in $CDCl_3$

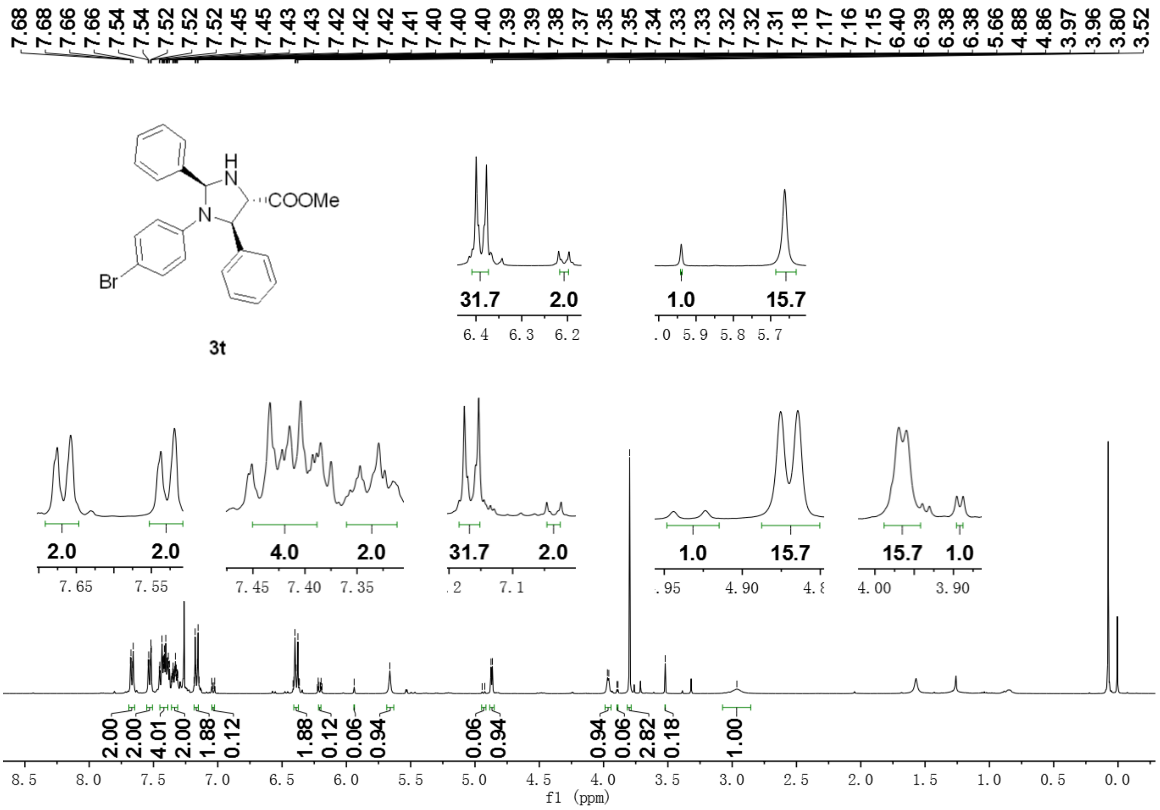

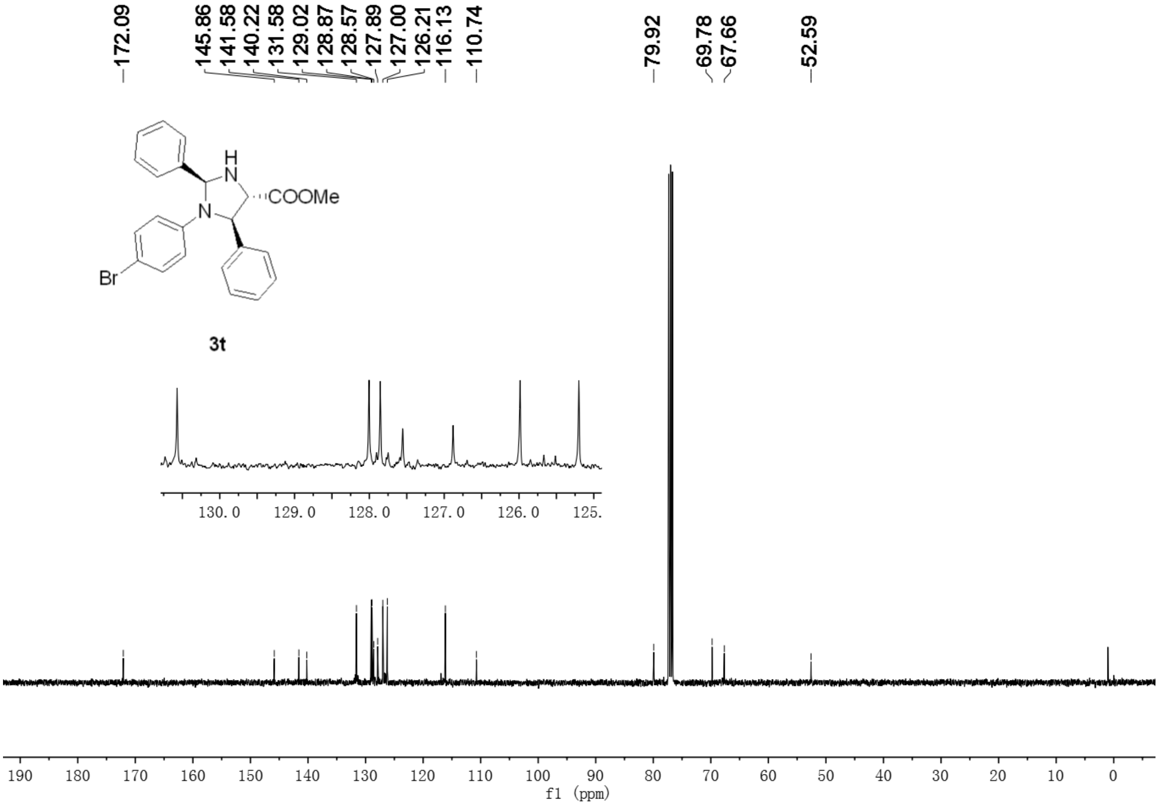

^{1}H NMR and $^{13}C\{^{1}H\}$ NMR spectra of compound of (2*R*,4*R*,5*S*)-**3t** (procedure A) in $CDCl_3$

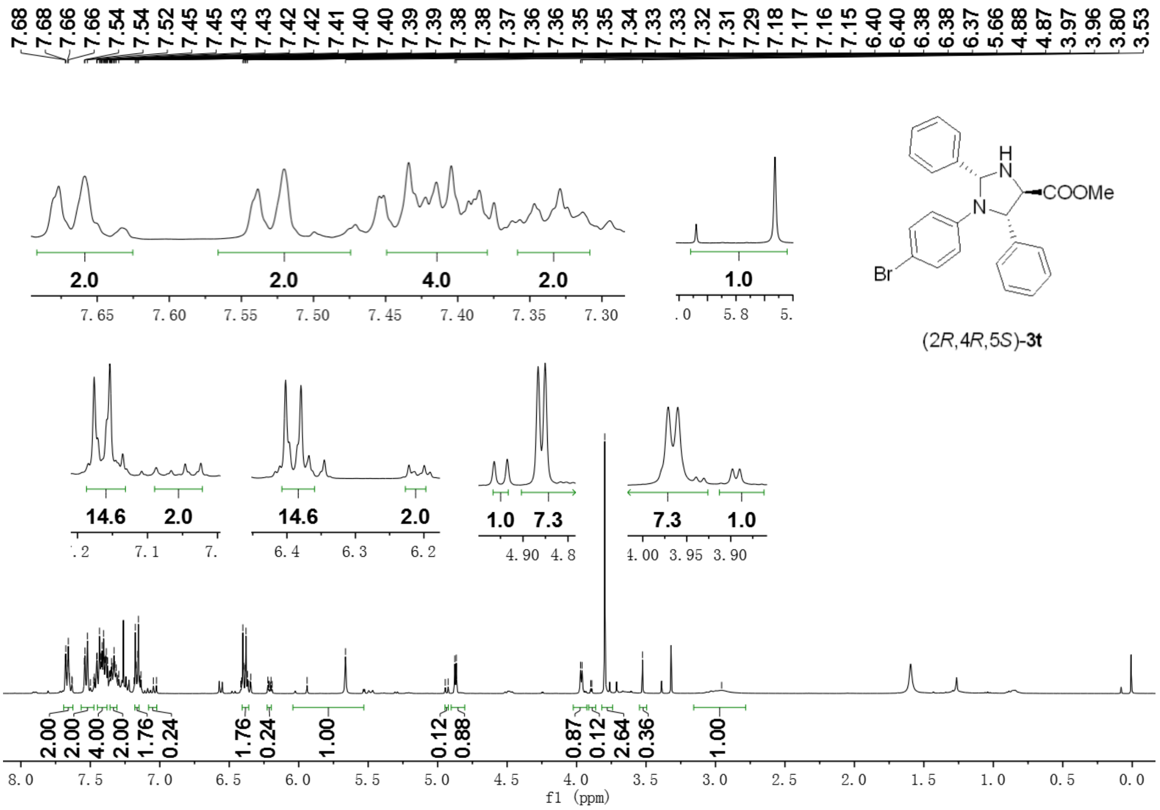

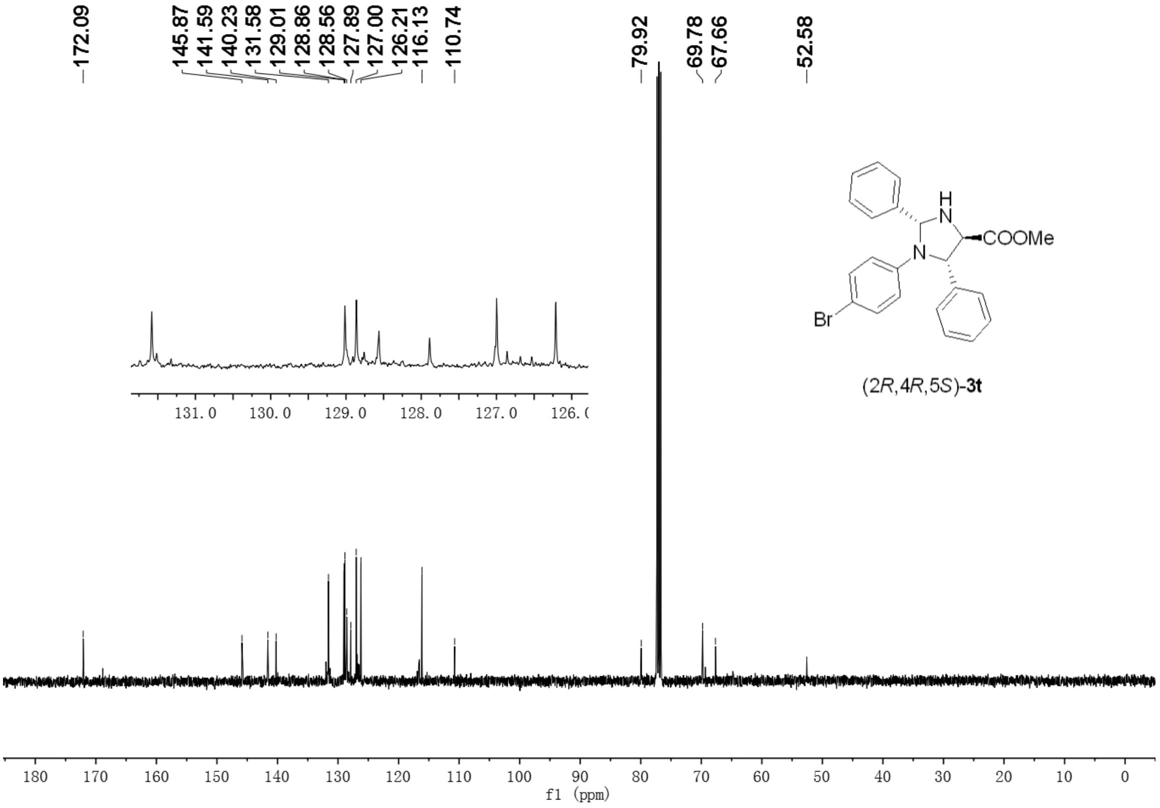

^{1}H NMR and ^{13}C{^{1}H} NMR spectra of compound of **3u** (procedure A) in $CDCl_3$

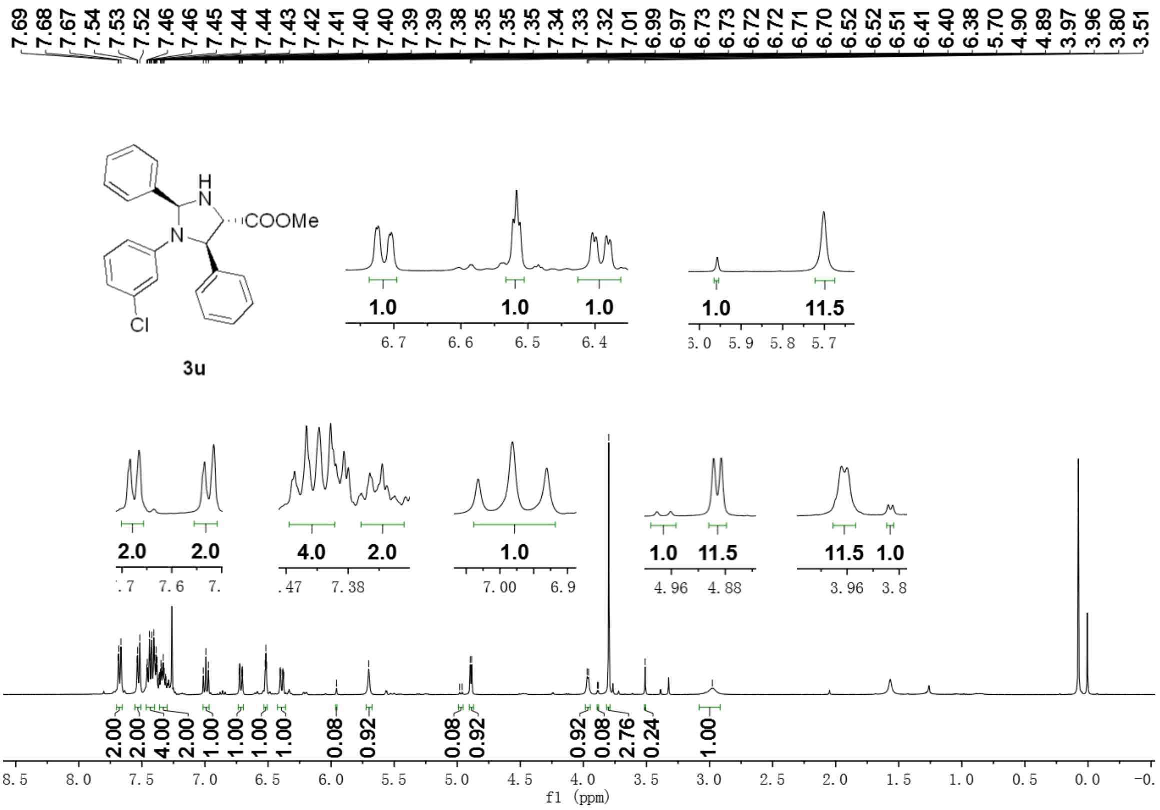

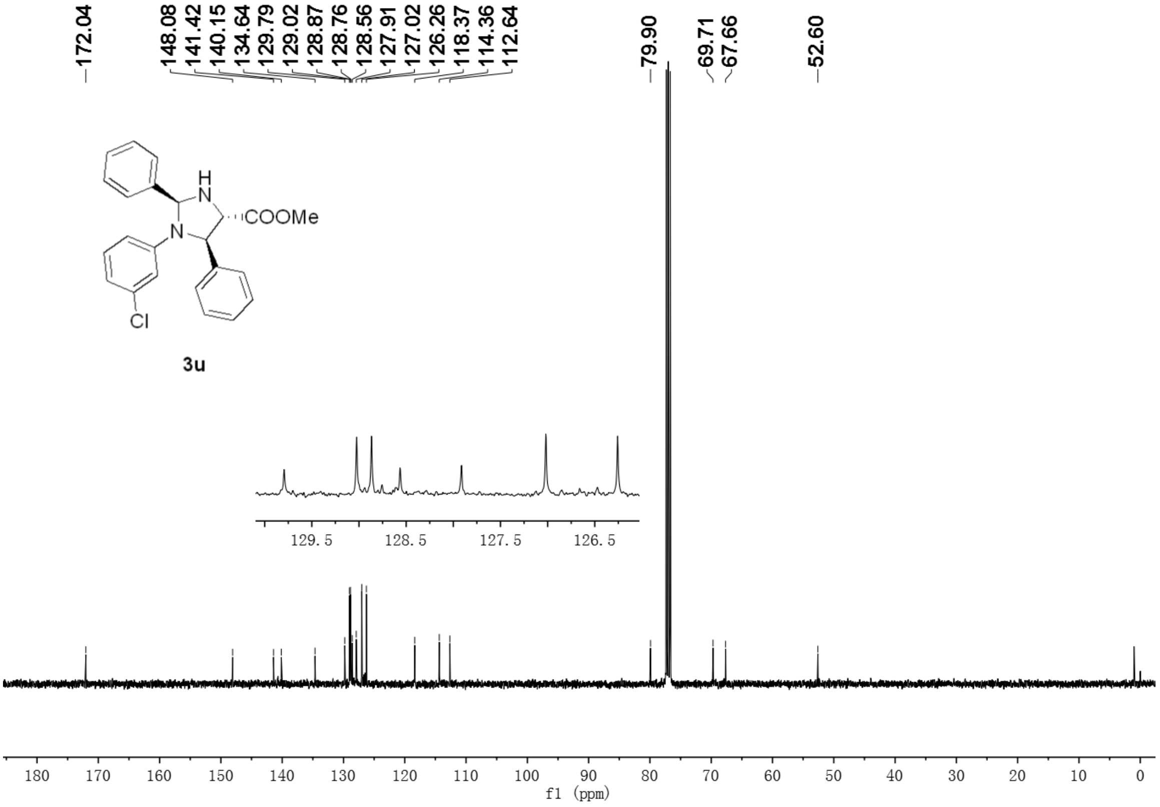

^{1}H NMR and $^{13}C\{^{1}H\}$ NMR spectra of compound of **3v** (procedure B) in $CDCl_3$

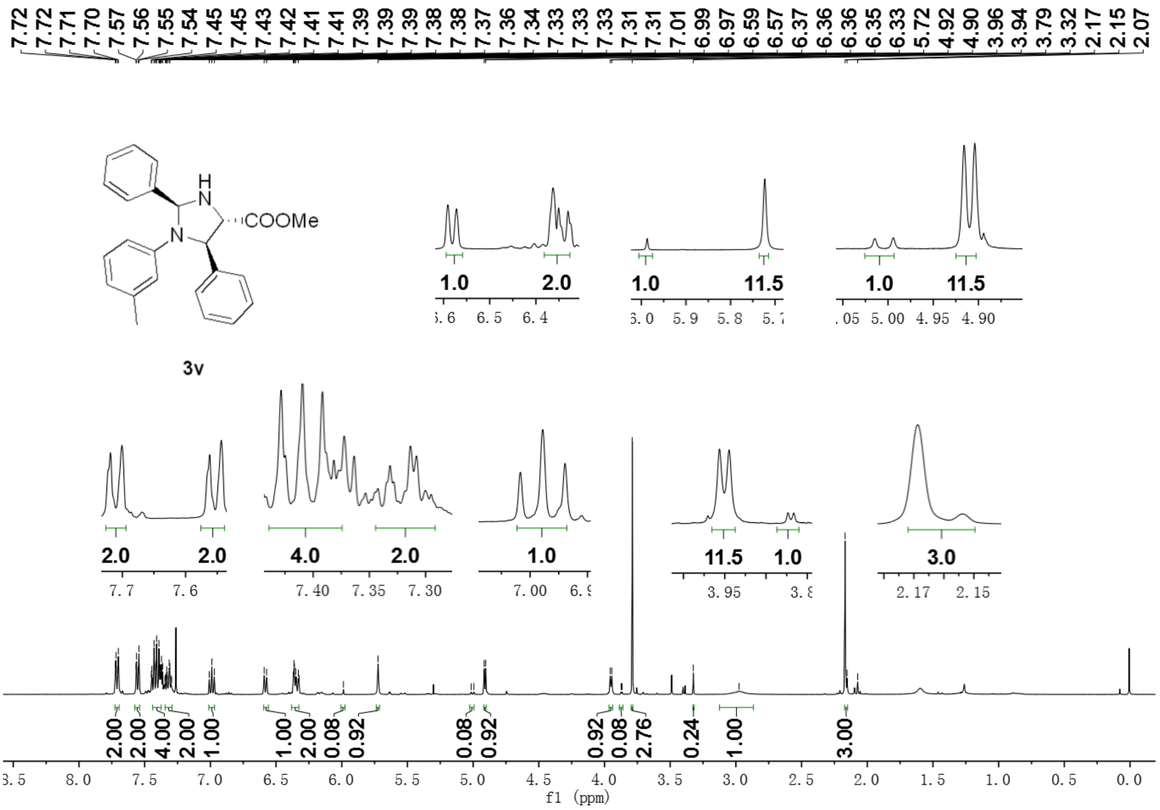

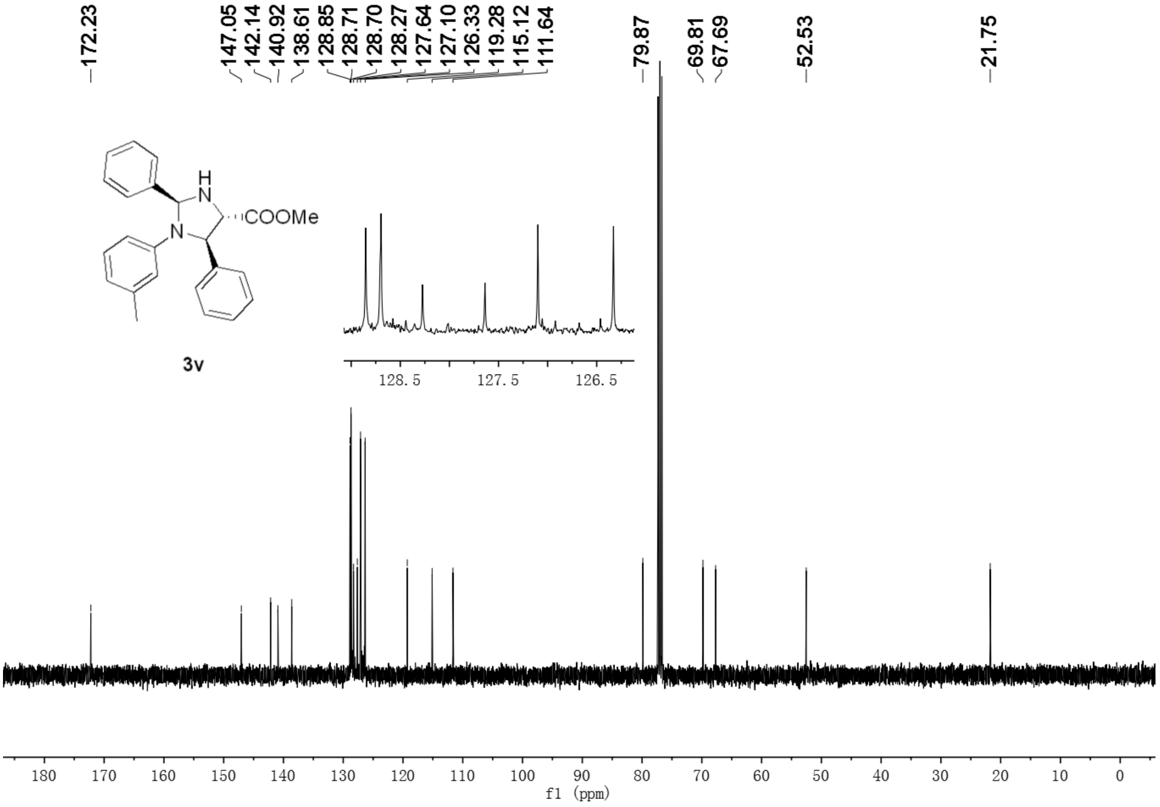

^{1}H NMR and ^{13}C{^{1}H} NMR spectra of compound of **3da** (procedure A) in $CDCl_3$

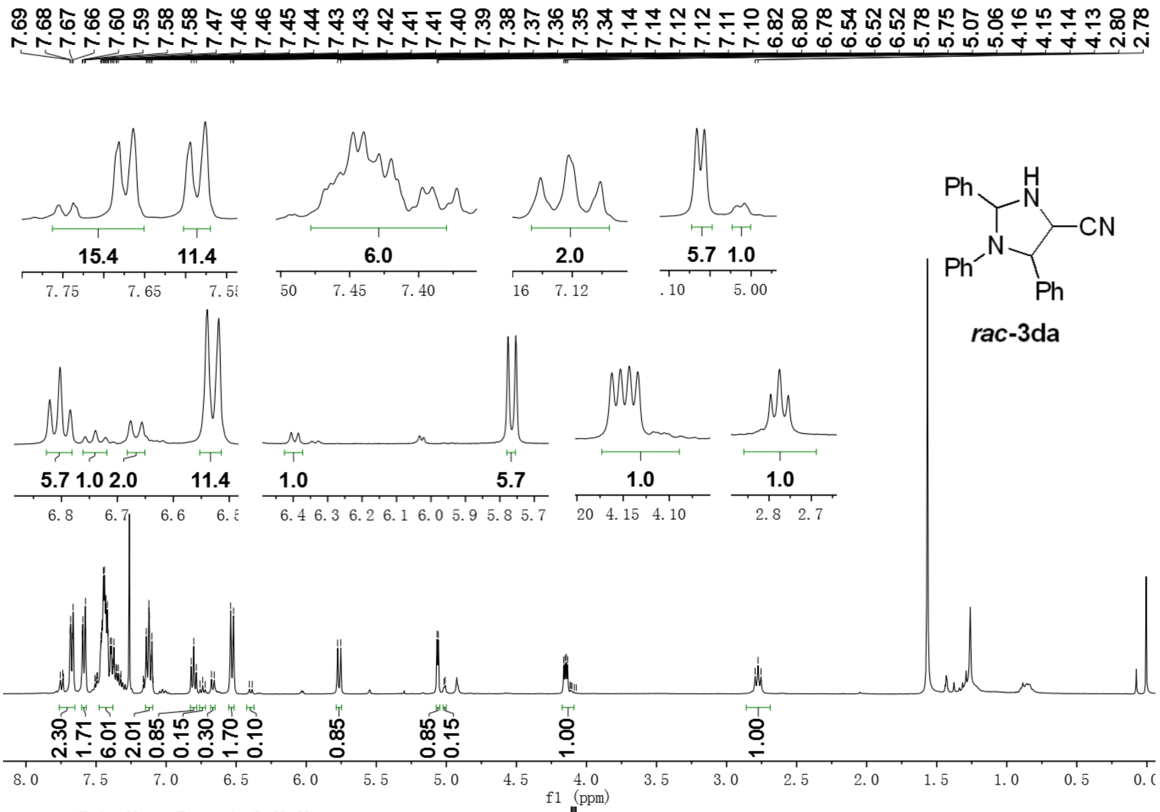

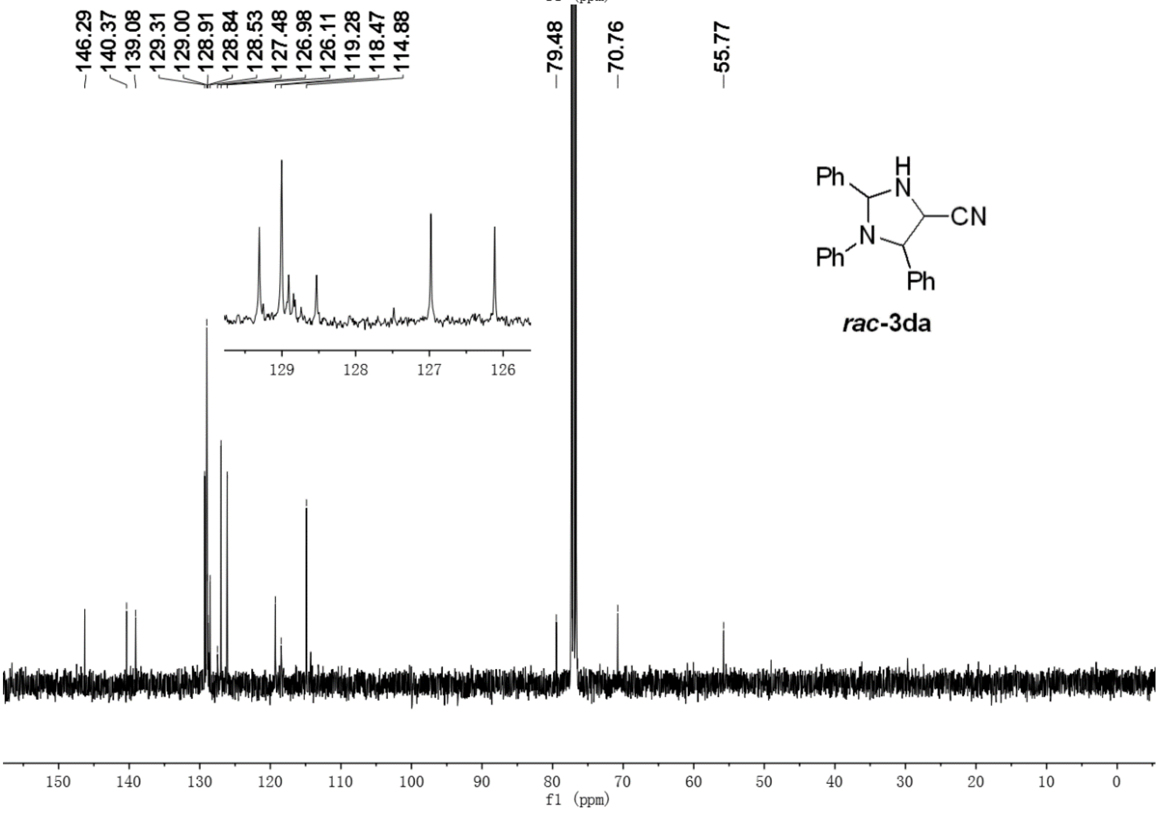

^{1}H NMR and ^{13}C{^{1}H} NMR spectra of compound of **3ala** (procedure A) in $CDCl_3$

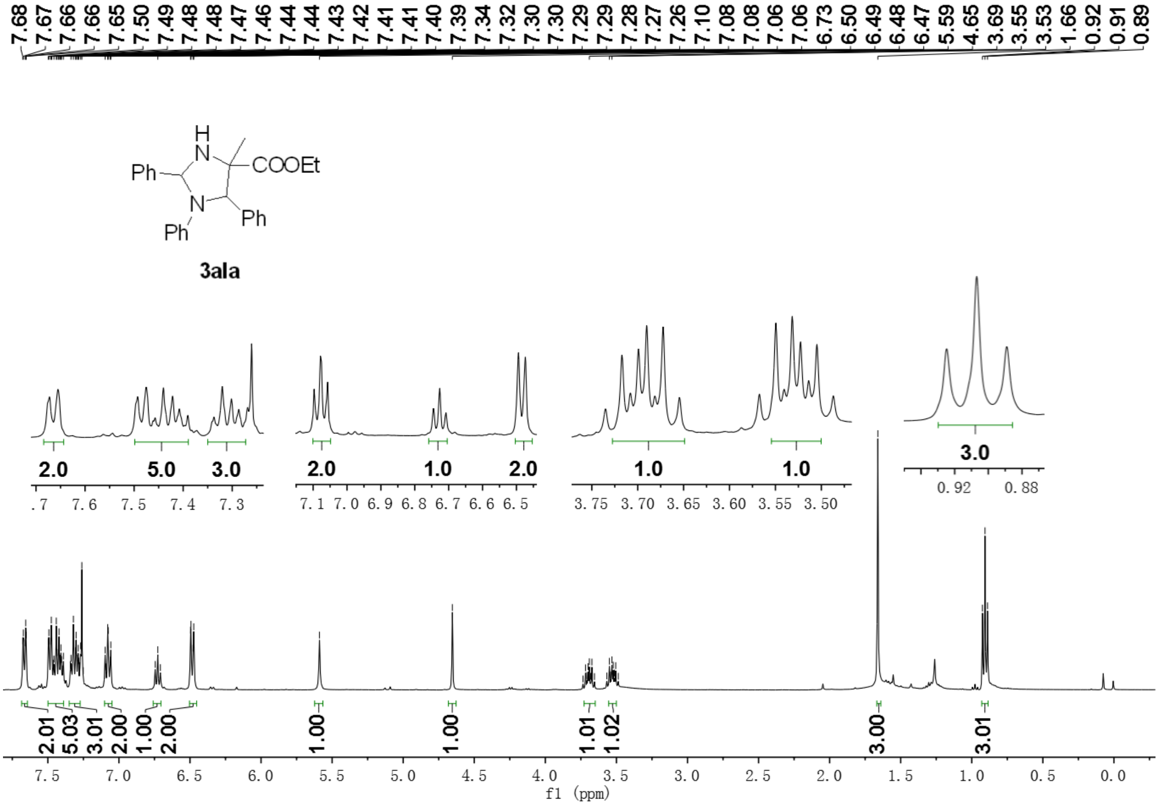

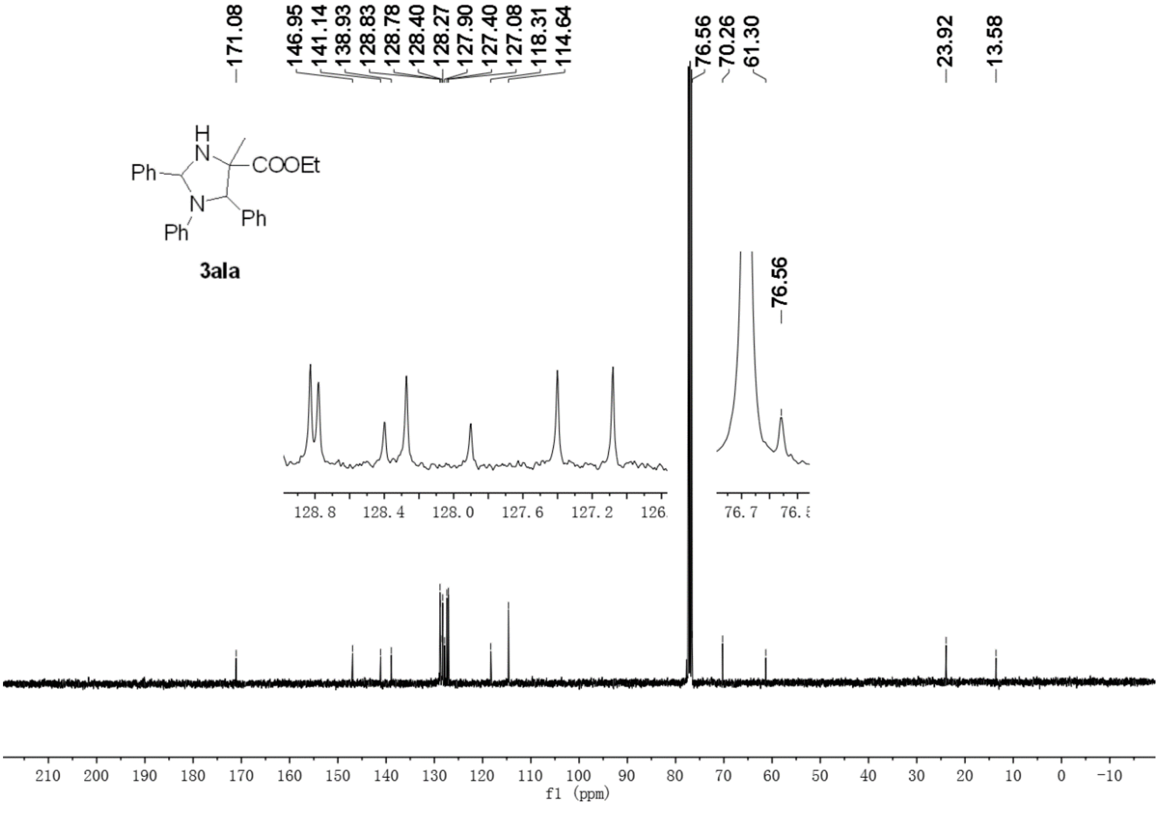

^{1}H NMR and ^{13}C{^{1}H} NMR spectra of compound of **3ser** (procedure A) in $CDCl_3$

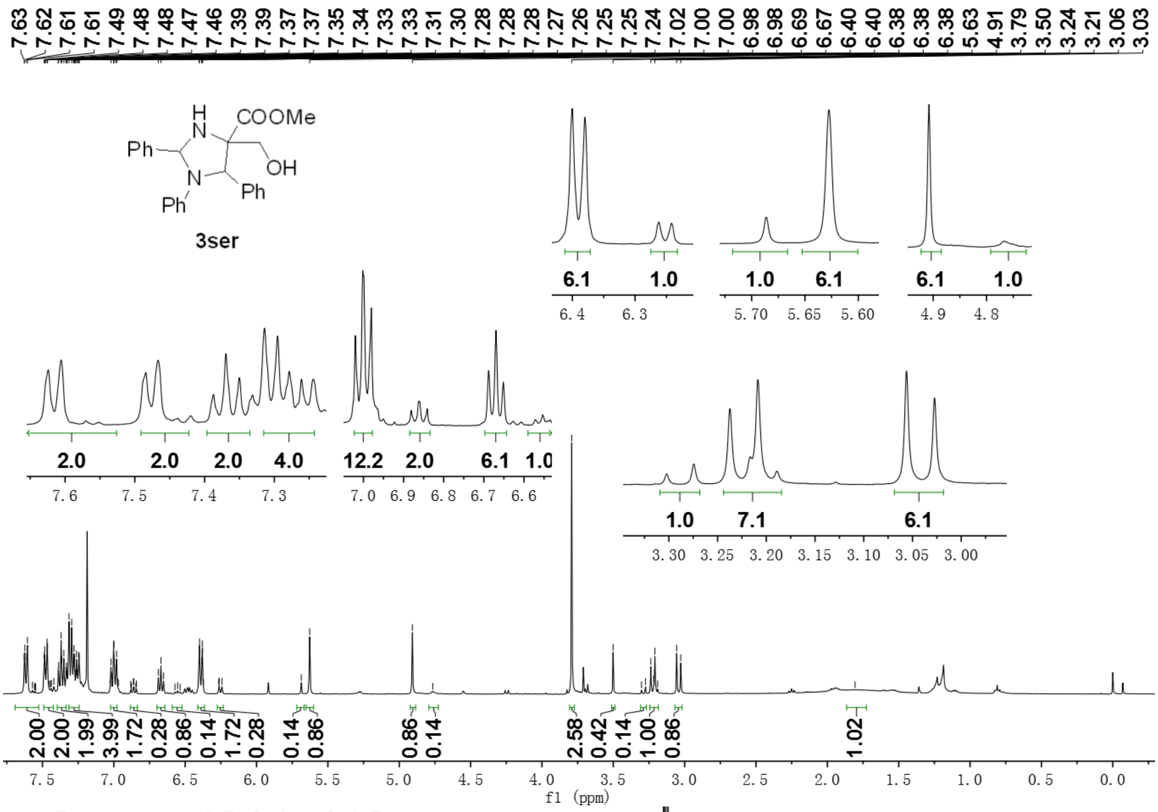

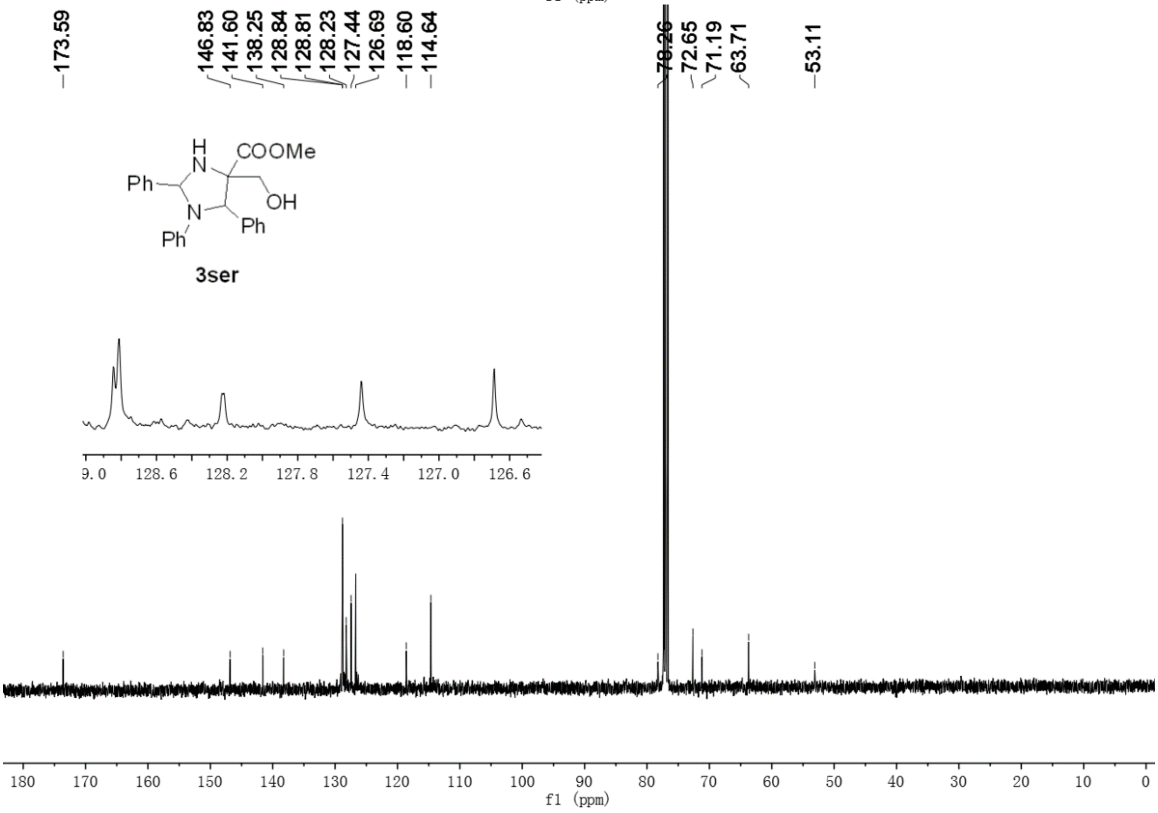

^{1}H NMR and ^{13}C{^{1}H} NMR spectra of compound of **3asp** (procedure A) in $CDCl_3$

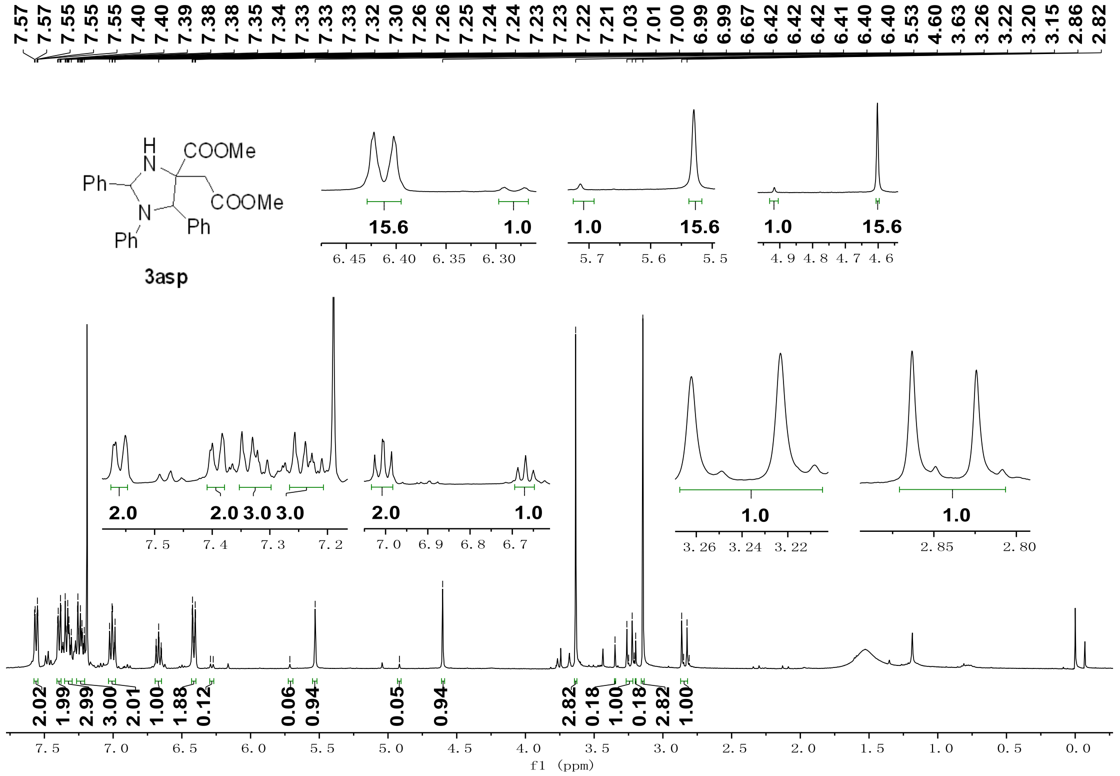

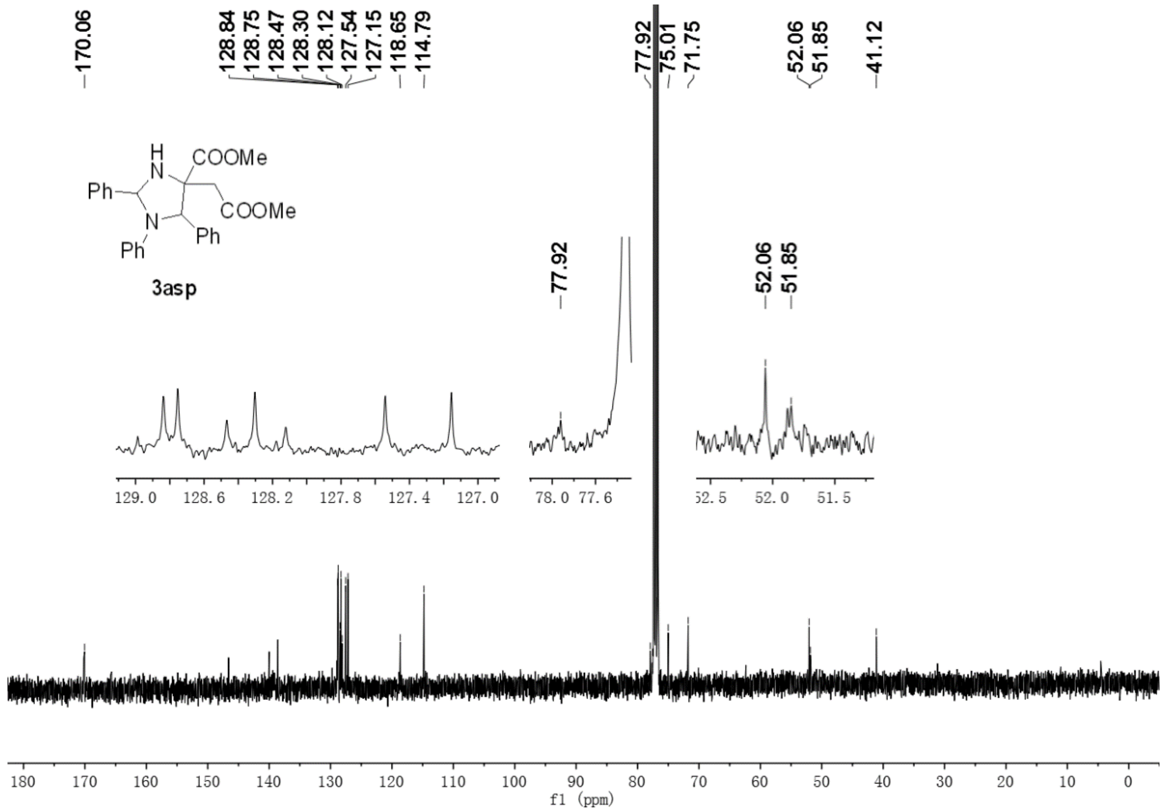

(O) Copies of CD Spectra in CH_2Cl_2

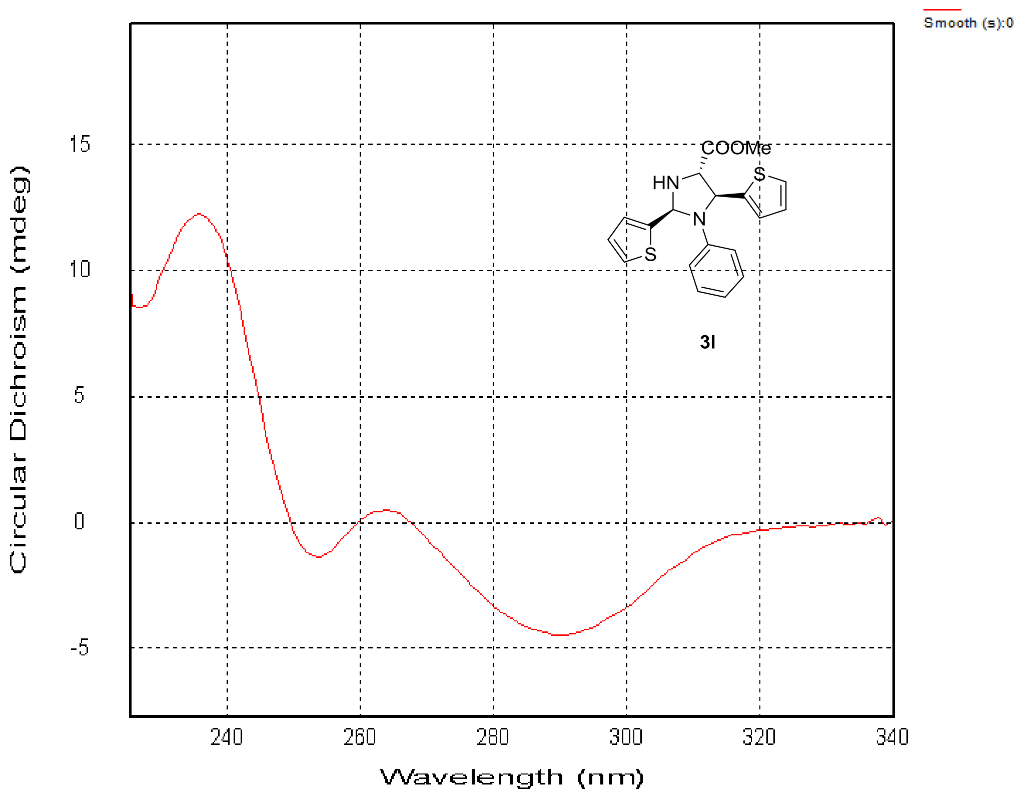

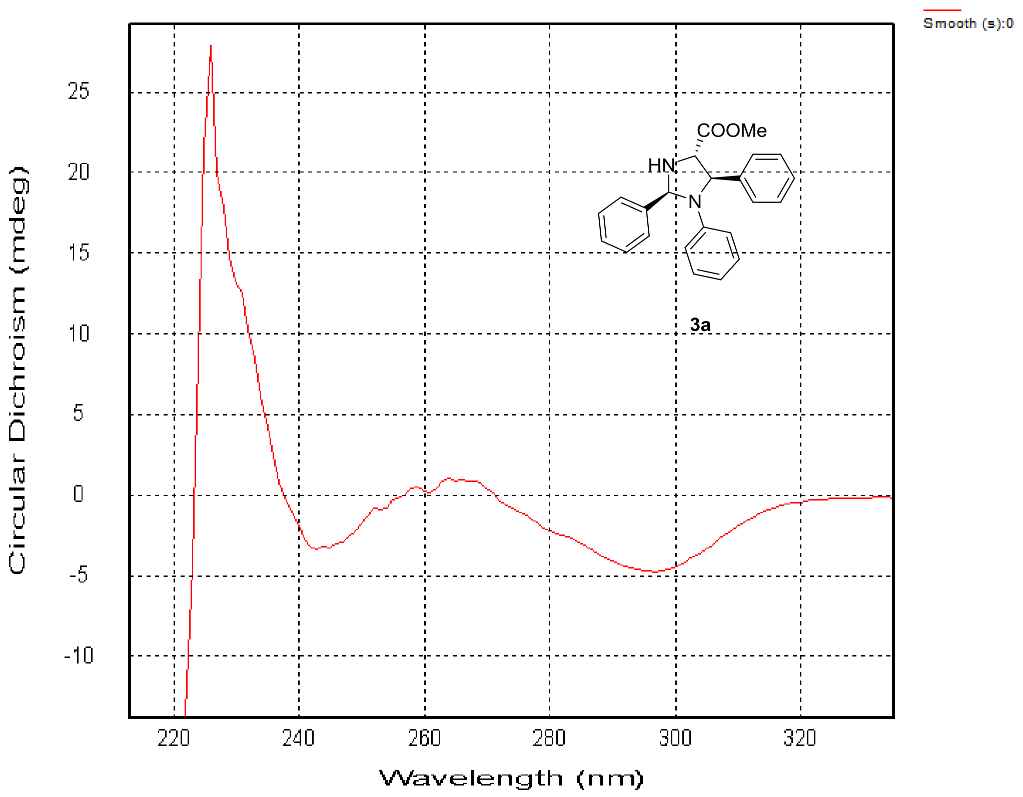

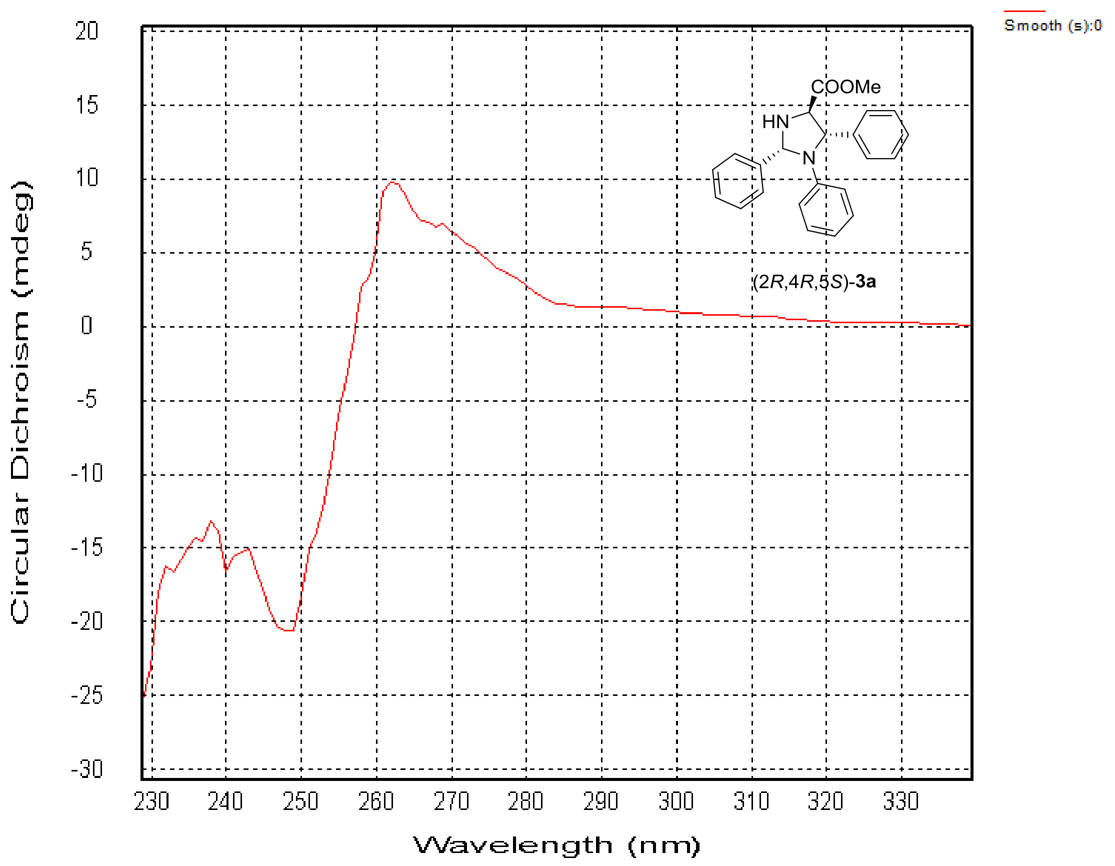
Smooth (s):0
COOMe
HN
N
(2R,4R,5S)-3a
Circular Dichroism (mdeg)
Wavelength (nm)
20
15
10
5
0
-5
-10
-15
-20
-25
-30
230
240
250
260
270
280
290
300
310
320
330

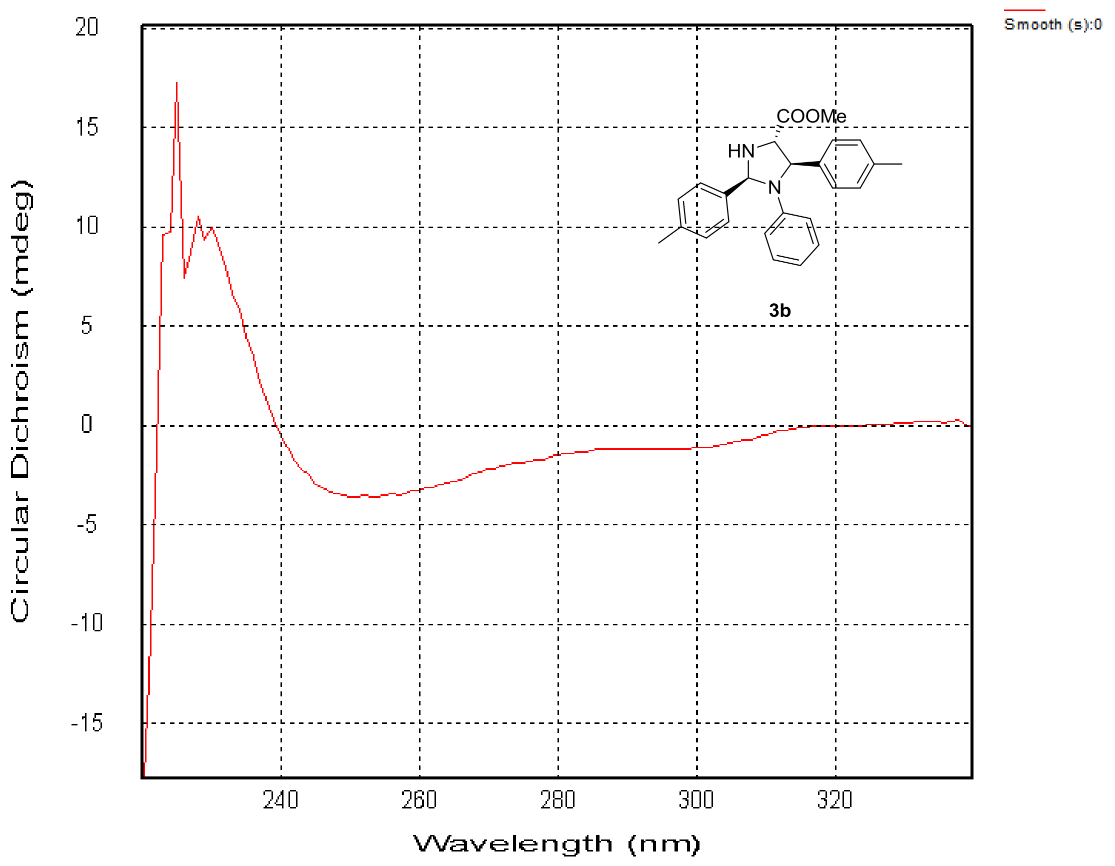
Smooth (s):0
COOMe
HN
N
3b
Circular Dichroism (mdeg)
Wavelength (nm)
20
15
10
5
0
-5
-10
-15
240
260
280
300
320

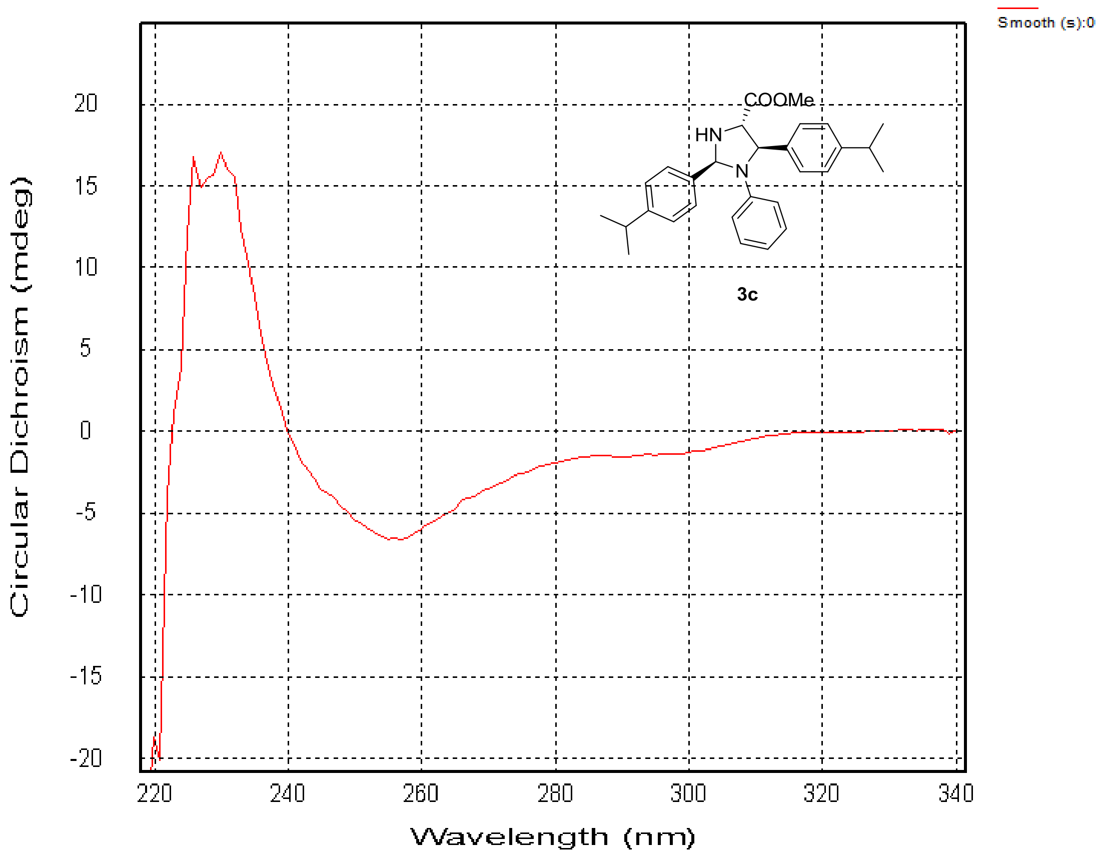
Smooth (s):0
Circular Dichroism (mdeg)
20
15
10
5
0
-5
-10
-15
-20
220
240
260
280
300
320
340
Wavelength (nm)
COOMe
HN
N
3c

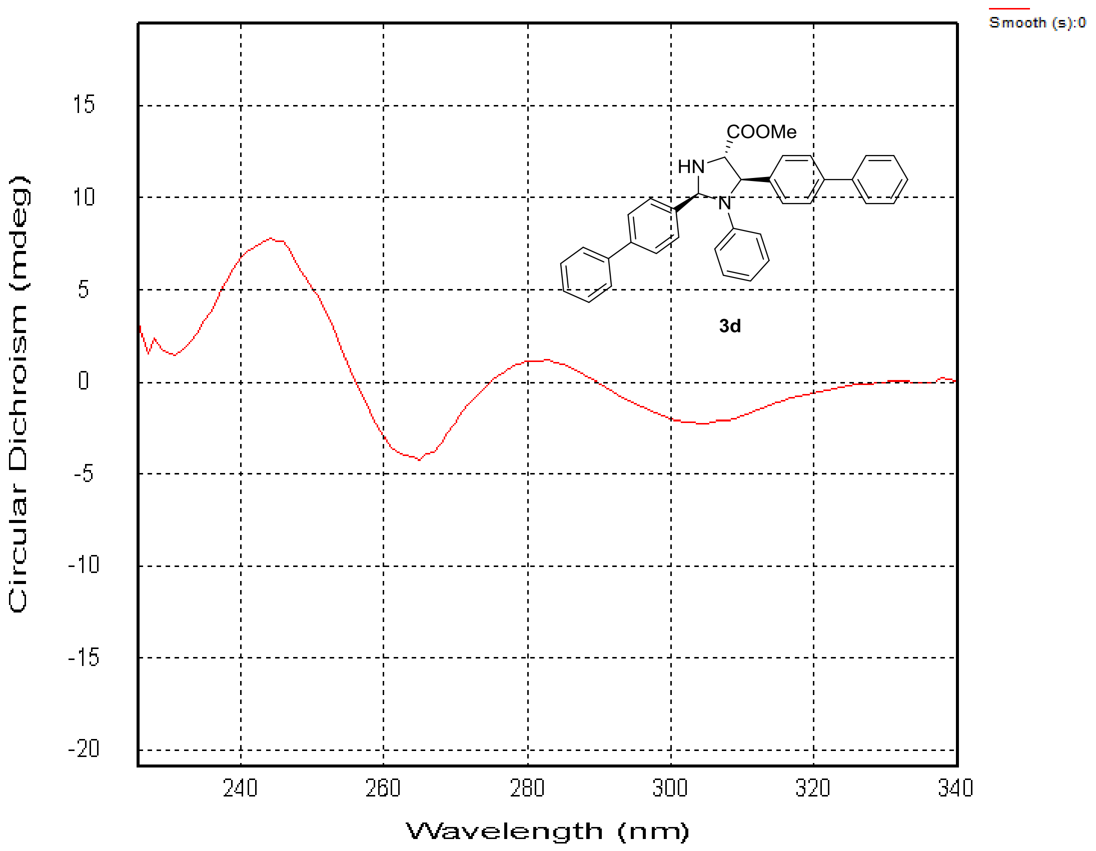
Smooth (s):0
Circular Dichroism (mdeg)
15
10
5
0
-5
-10
-15
-20
240
260
280
300
320
340
Wavelength (nm)
COOMe
HN
N
3d

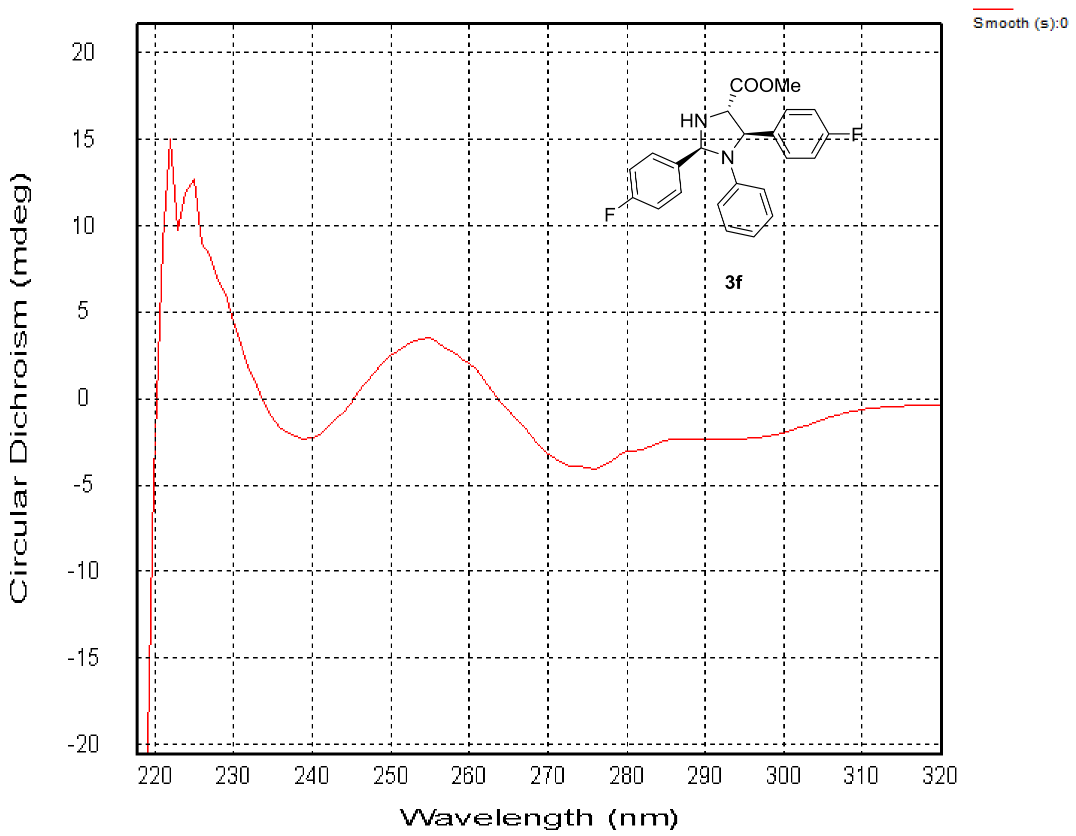
Smooth (s):0
20
15
10
5
0
-5
-10
-15
-20
Circular Dichroism (mdeg)
220
230
240
250
260
270
280
290
300
310
320
Wavelength (nm)
COOMe
HN
N
F
F
3f

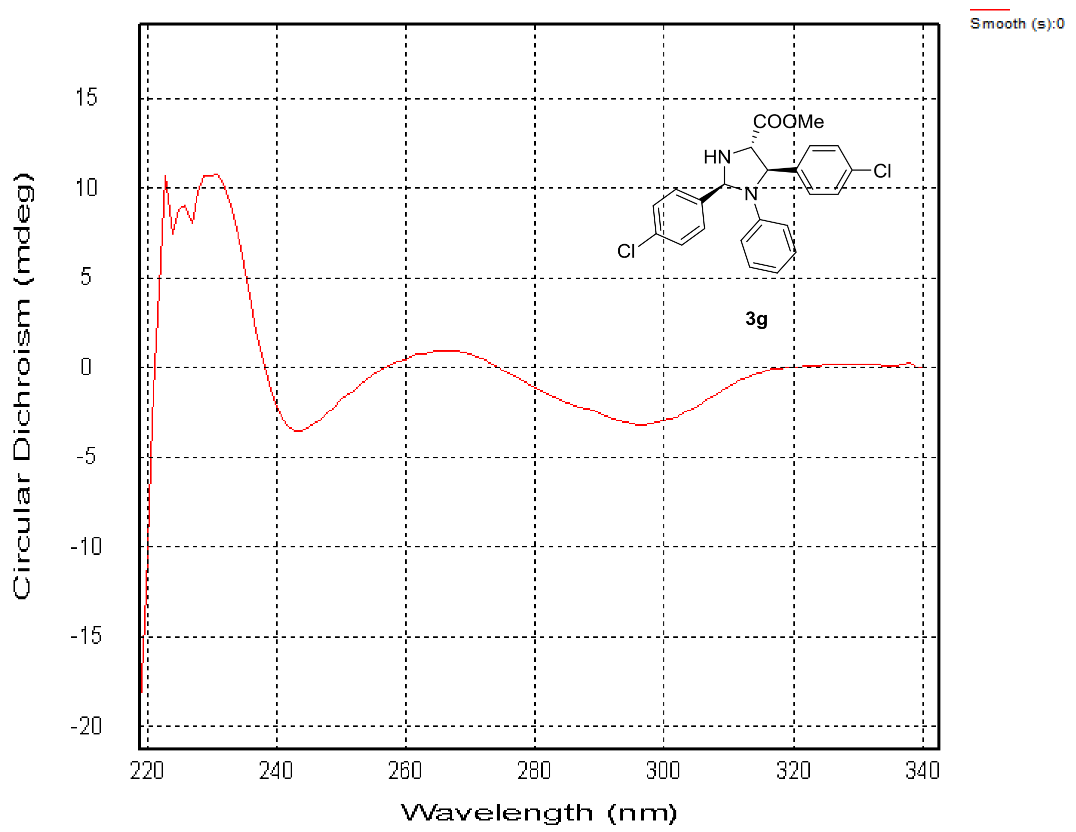
Smooth (s):0
15
10
5
0
-5
-10
-15
-20
Circular Dichroism (mdeg)
220
240
260
280
300
320
340
Wavelength (nm)
COOMe
HN
N
Cl
Cl
3g

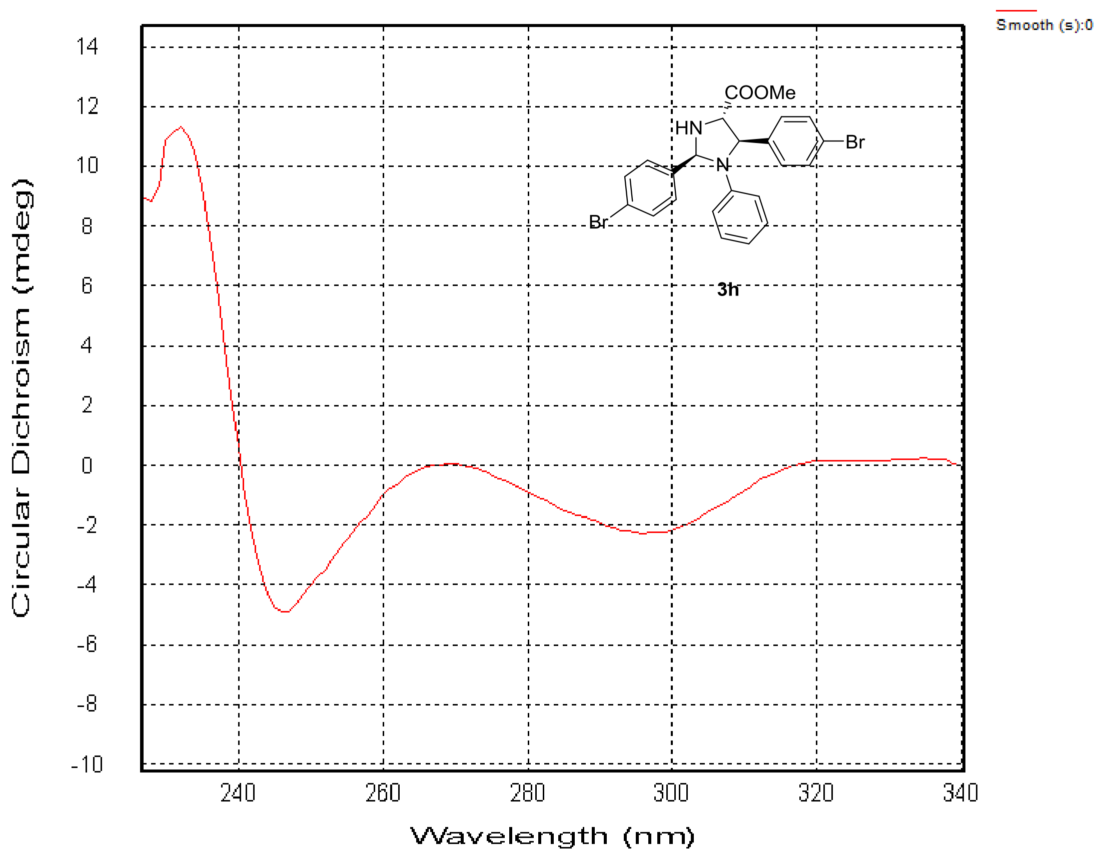
Smooth (s):0
14
12
10
8
6
4
2
0
-2
-4
-6
-8
-10
Circular Dichroism (mdeg)
240
260
280
300
320
340
Wavelength (nm)
COOMe
HN
N
Br
Br
3h

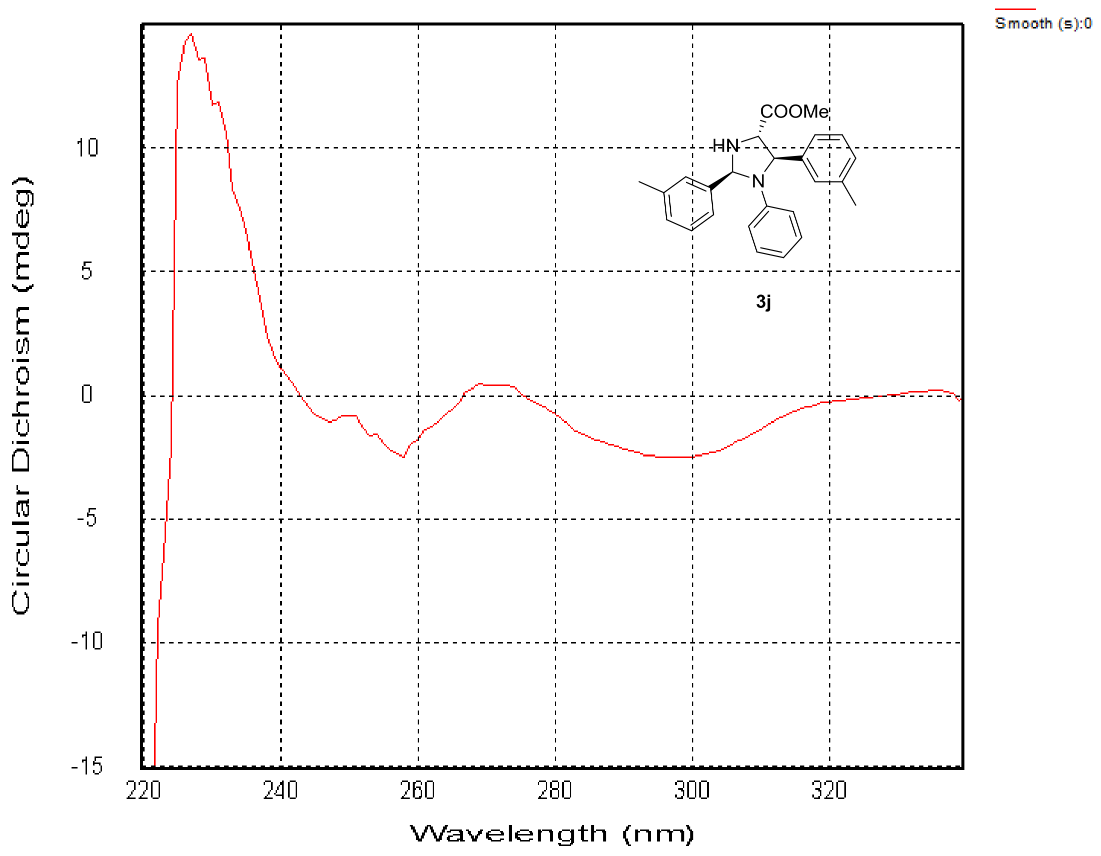
Smooth (s):0
10
5
0
-5
-10
-15
Circular Dichroism (mdeg)
220
240
260
280
300
320
Wavelength (nm)
COOMe
HN
N
3j

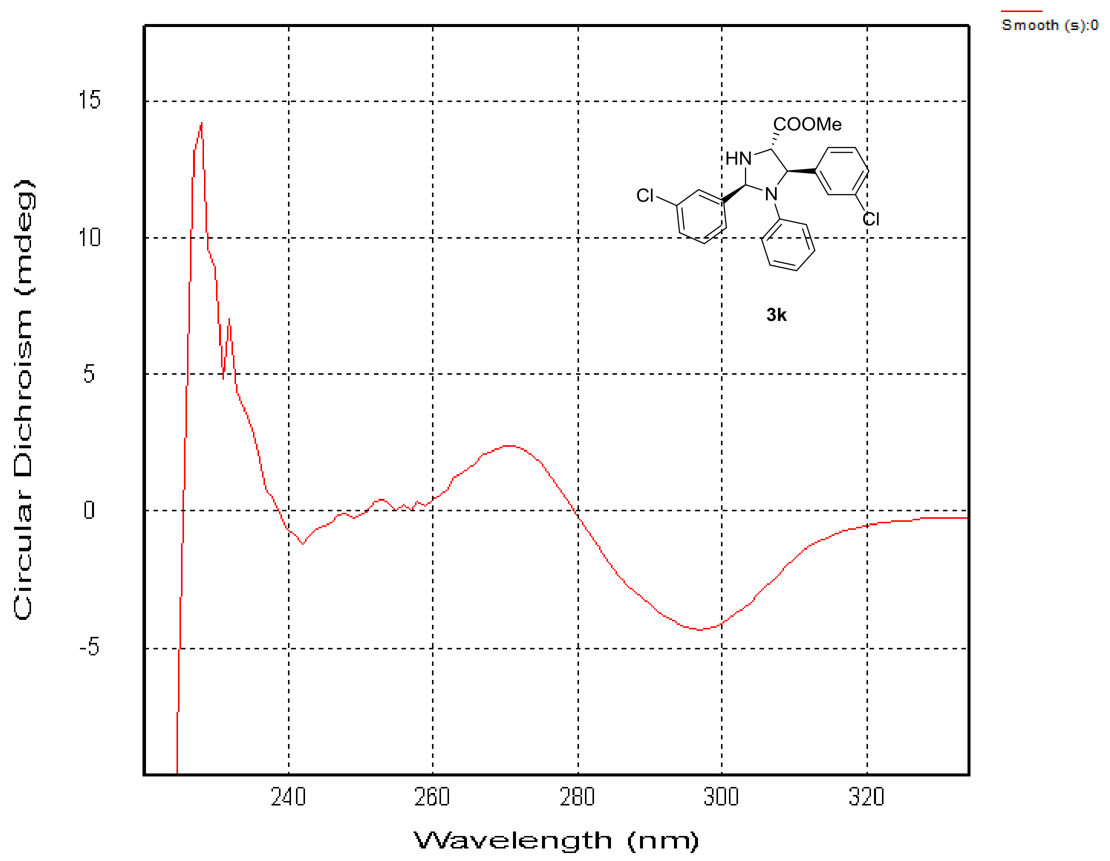
Smooth (s):0
COOMe
HN
N
Cl
Cl
3k
15
10
5
0
-5
240
260
280
300
320
Circular Dichroism (mdeg)
Wavelength (nm)

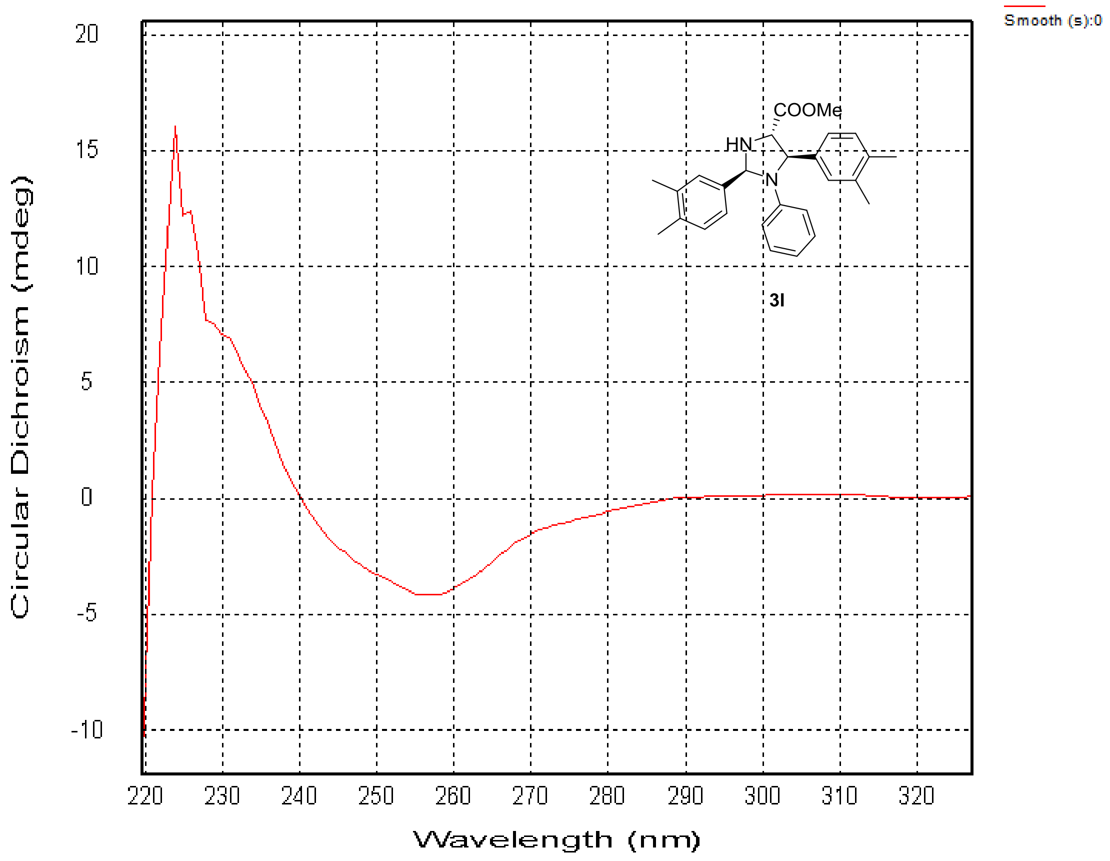
Smooth (s):0
COOMe
HN
N
3l
20
15
10
5
0
-5
-10
220
230
240
250
260
270
280
290
300
310
320
Circular Dichroism (mdeg)
Wavelength (nm)

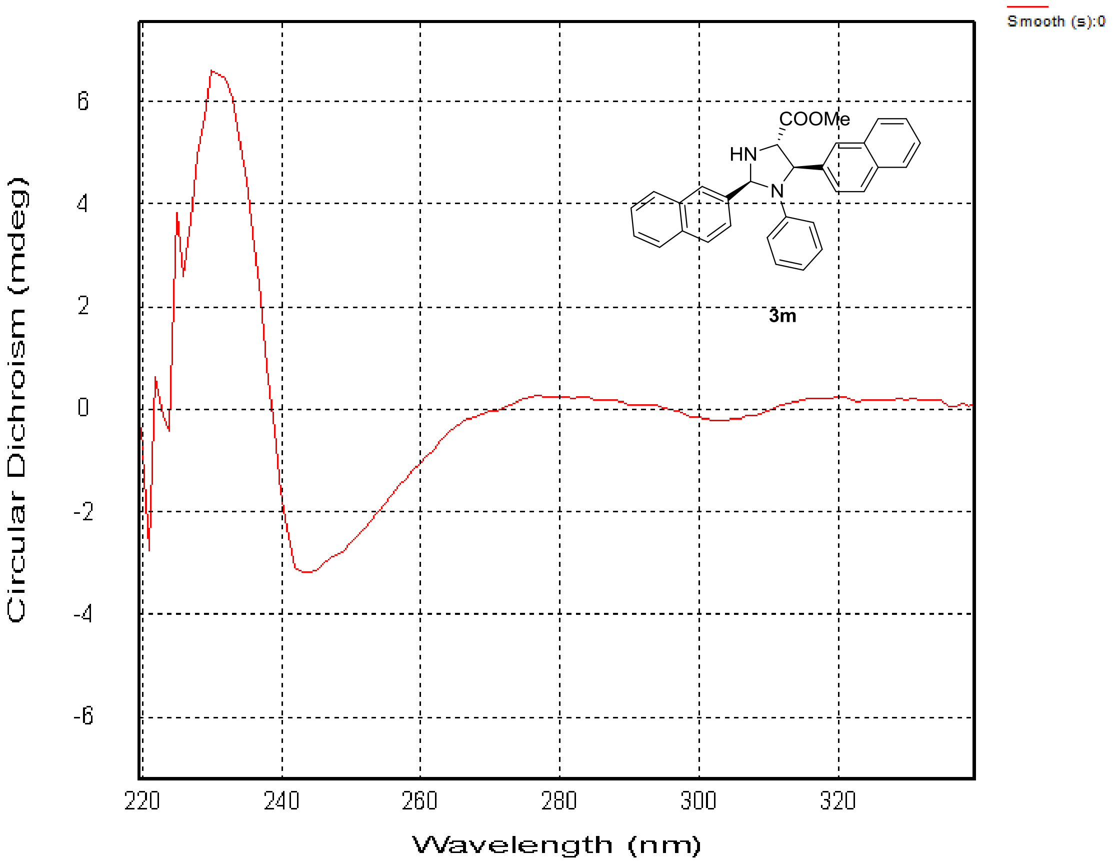
Smooth (s):0
6
4
2
0
-2
-4
-6
Circular Dichroism (mdeg)
220
240
260
280
300
320
Wavelength (nm)
COOMe
HN
N
3m

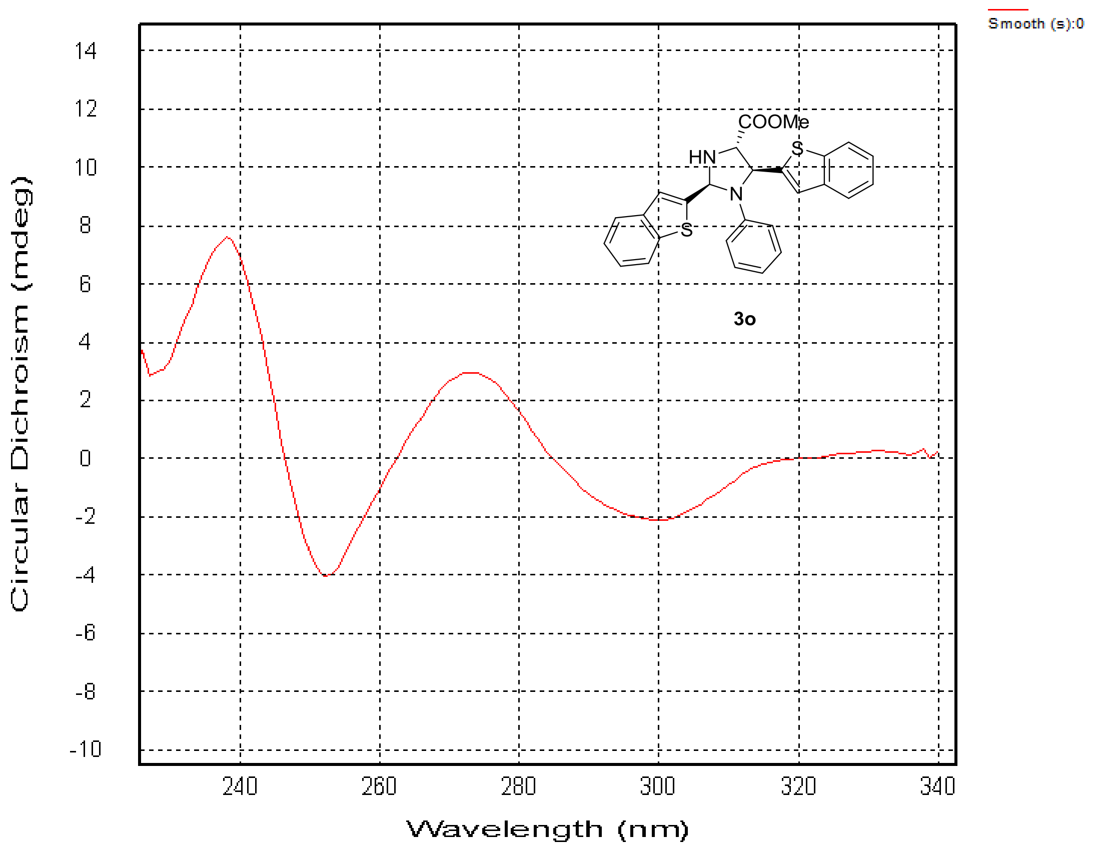
Smooth (s):0
14
12
10
8
6
4
2
0
-2
-4
-6
-8
-10
Circular Dichroism (mdeg)
240
260
280
300
320
340
Wavelength (nm)
COOMe
HN
N
S
S
3o

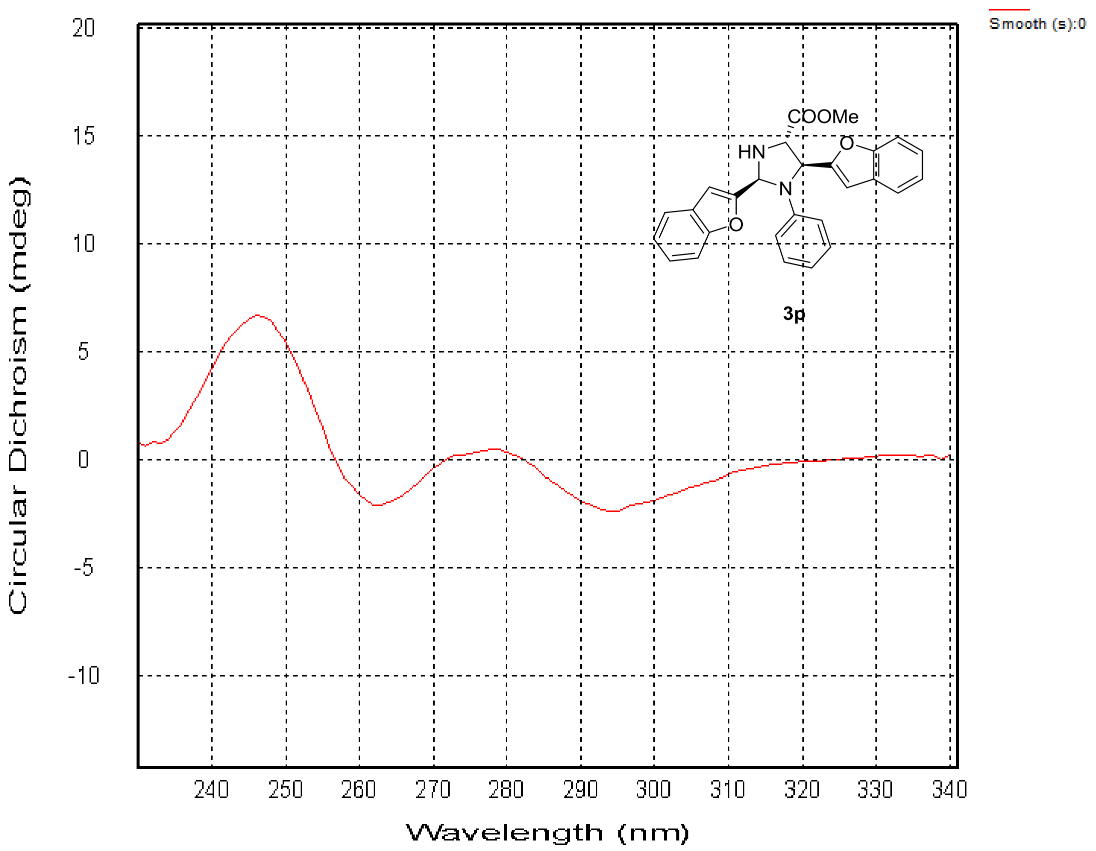
Smooth (s):0
20
15
10
5
0
-5
-10
Circular Dichroism (mdeg)
240
250
260
270
280
290
300
310
320
330
340
Wavelength (nm)
COOMe
HN
N
O
O
3p

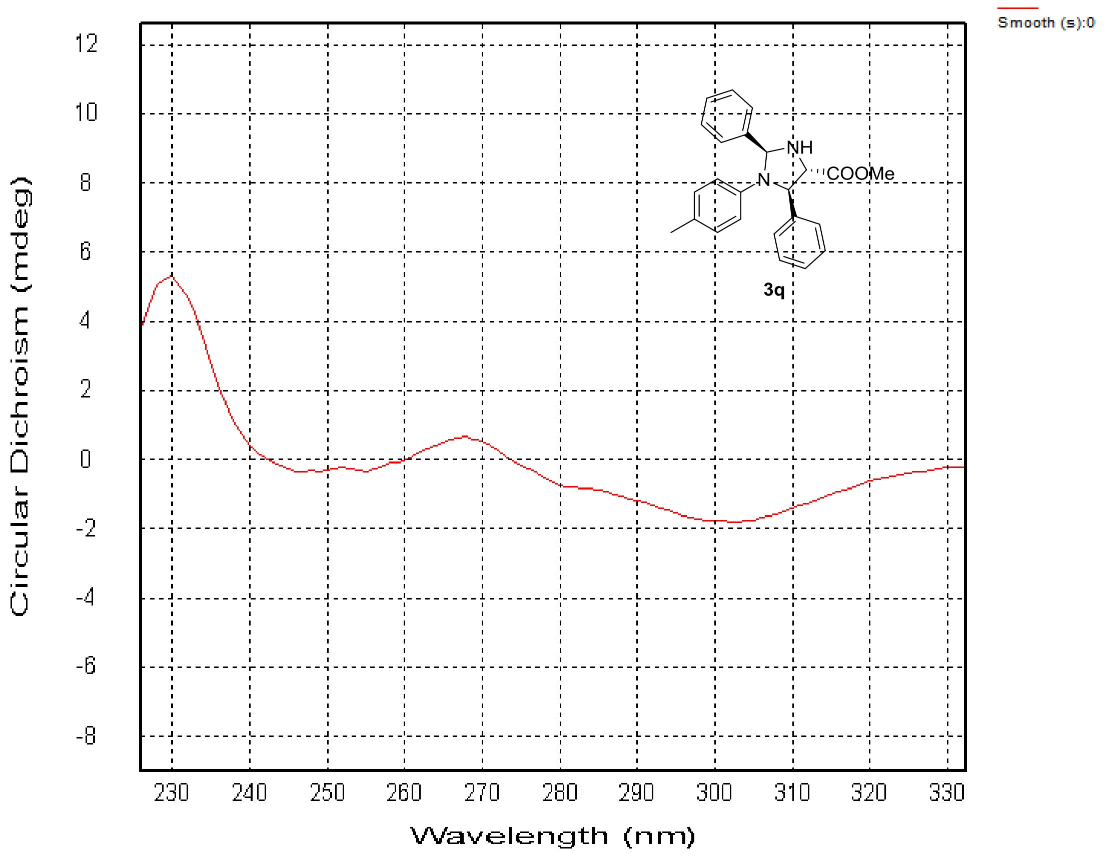
Smooth (s):0
12
10
8
6
4
2
0
-2
-4
-6
-8
Circular Dichroism (mdeg)
230
240
250
260
270
280
290
300
310
320
330
Wavelength (nm)
NH
N
COOMe
3q

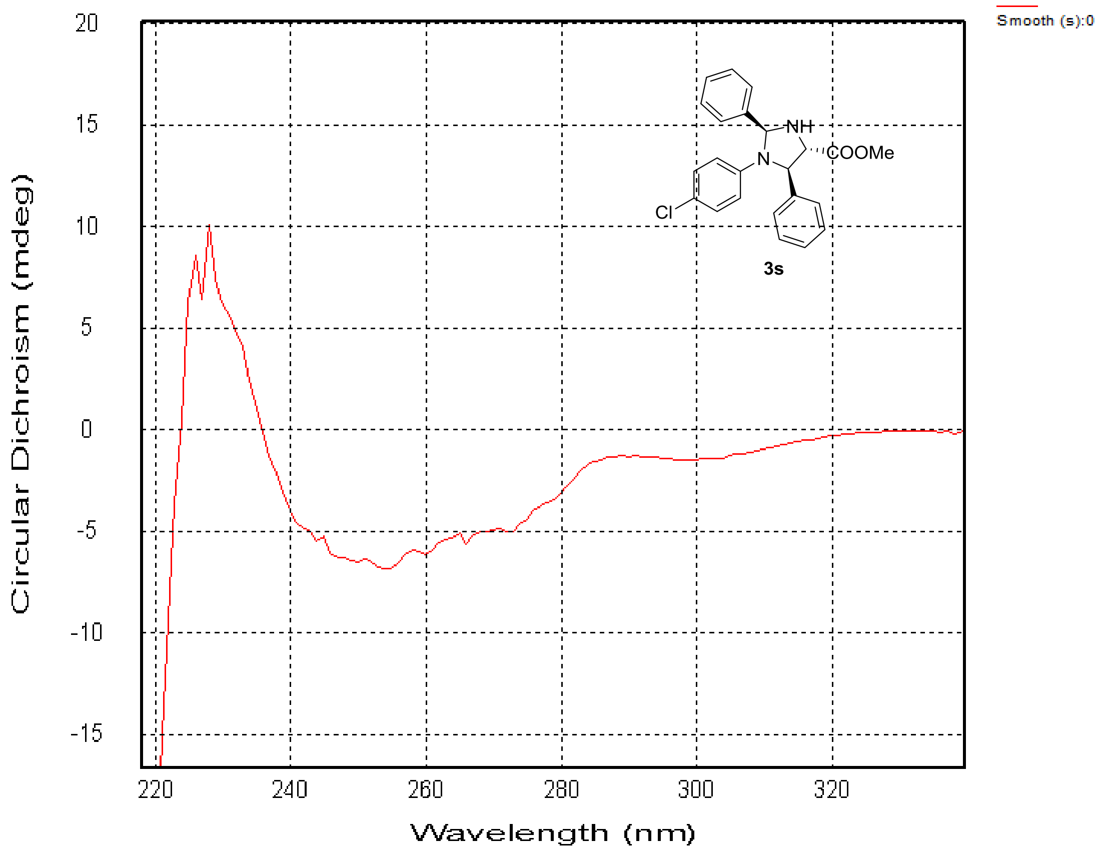

Smooth (s):0
20
15
10
5
0
-5
-10
-15
Circular Dichroism (mdeg)
220
240
260
280
300
320
Wavelength (nm)
NH
N
COOMe
Cl
3s

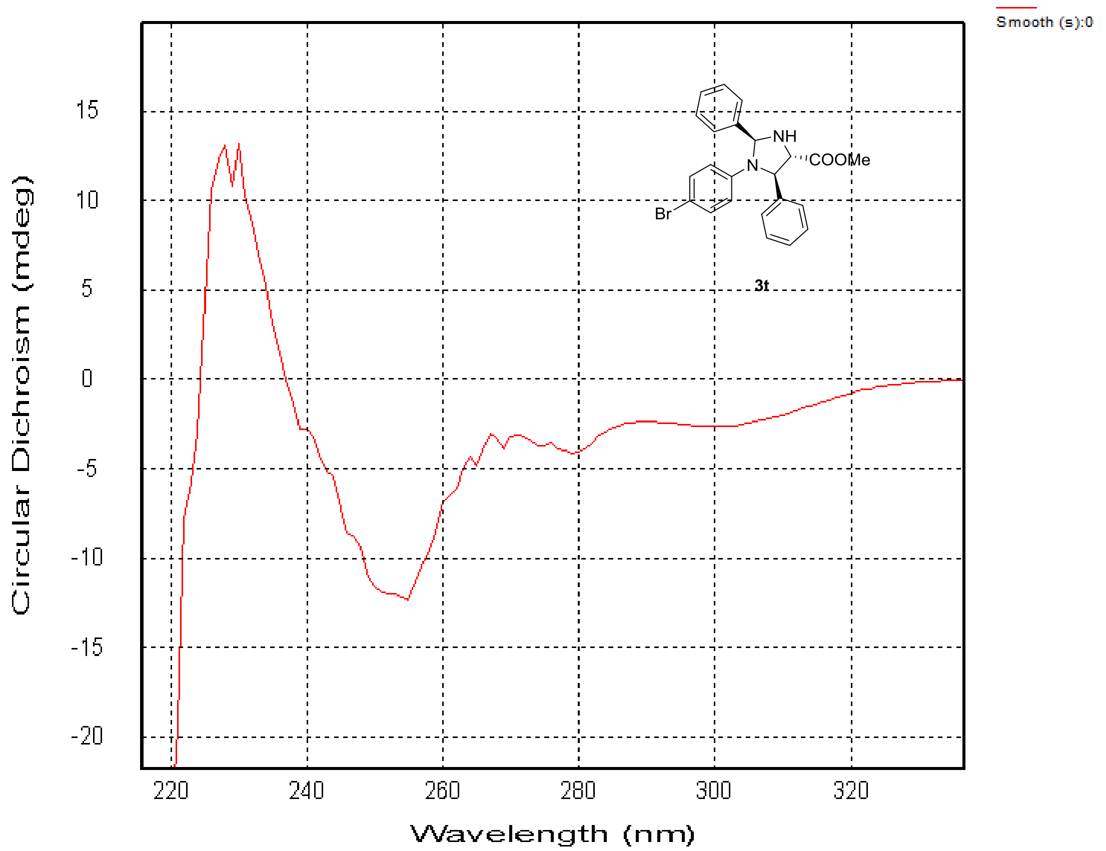

Smooth (s):0
15
10
5
0
-5
-10
-15
-20
Circular Dichroism (mdeg)
220
240
260
280
300
320
Wavelength (nm)
NH
N
COOMe
Br
3t

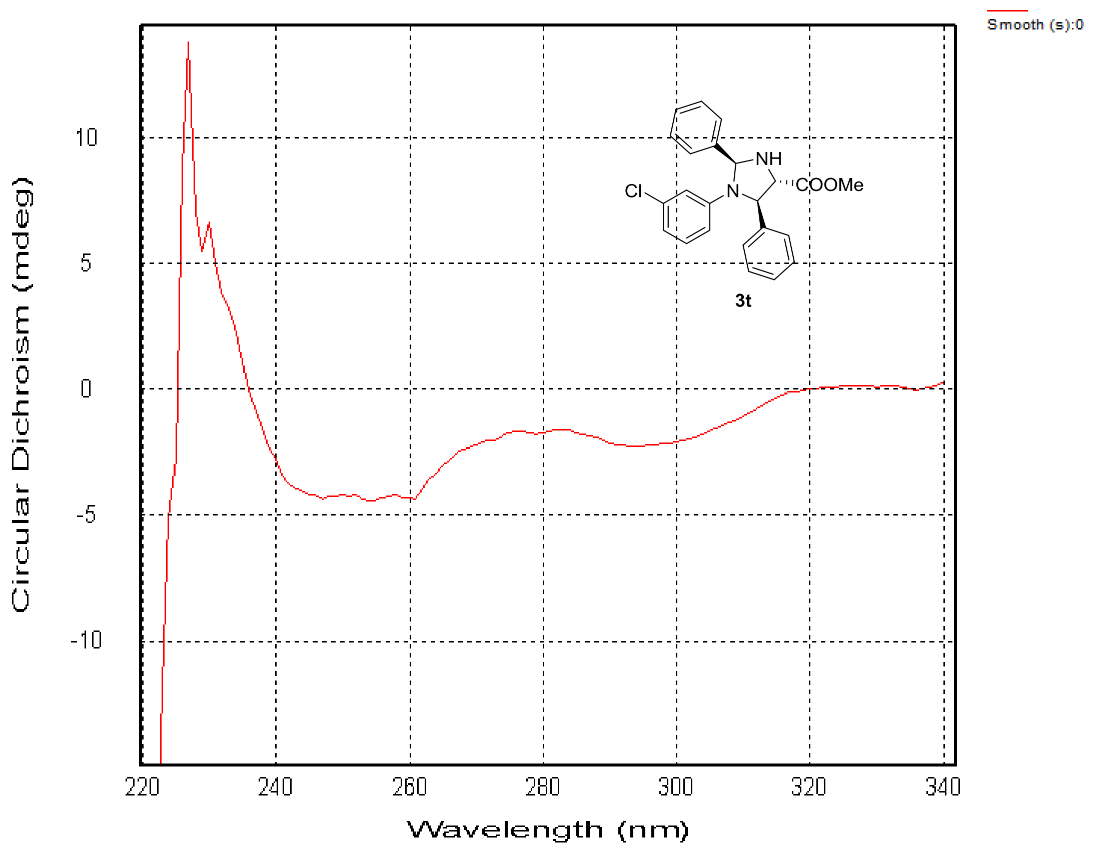
Smooth (s):0
Circular Dichroism (mdeg)
Wavelength (nm)
NH
COOMe
Cl
N
3t
10
5
0
-5
-10
220
240
260
280
300
320
340

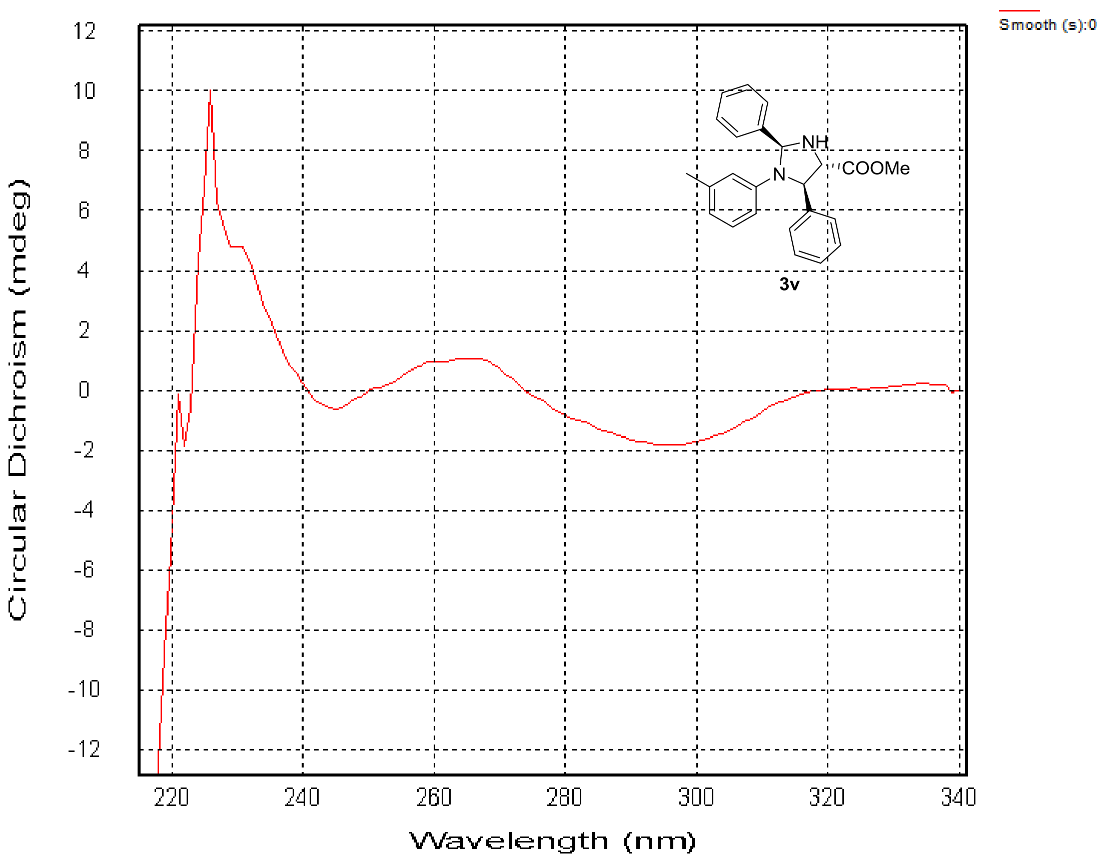
Smooth (s):0
Circular Dichroism (mdeg)
Wavelength (nm)
NH
COOMe
N
3v
12
10
8
6
4
2
0
-2
-4
-6
-8
-10
-12
220
240
260
280
300
320
340
